# Photoinduced Olefin Diamination with Alkylamines

**DOI:** 10.1002/anie.202005652

**Published:** 2020-06-09

**Authors:** Sebastian Govaerts, Lucrezia Angelini, Charlotte Hampton, Laia Malet‐Sanz, Alessandro Ruffoni, Daniele Leonori

**Affiliations:** ^1^ Department of Chemistry University of Manchester Oxford Road Manchester M13 9PL UK; ^2^ Eli Lilly and Company Limited Erl Wood Manor Windlesham Surrey GU20 6PH UK

**Keywords:** alkylamines, aminochlorination, diamination, nitrogen radicals, olefin functionalization

## Abstract

Vicinal diamines are ubiquitous materials in organic and medicinal chemistry. The direct coupling of olefins and amines would be an ideal approach to construct these motifs. However, alkene diamination remains a long‐standing challenge in organic synthesis, especially when using two different amine components. We report a general strategy for the direct and selective assembly of vicinal 1,2‐diamines using readily available olefin and amine building blocks. This mild and straightforward approach involves in situ formation and photoinduced activation of N‐chloroamines to give aminium radicals that enable efficient alkene aminochlorination. Owing to the ambiphilic nature of the β‐chloroamines produced, conversion into tetra‐alkyl aziridinium ions was possible, thus enabling diamination by regioselective ring‐opening with primary or secondary amines. This strategy streamlines the preparation of vicinal diamines from multistep sequences to a single chemical transformation.

## Graphical abstract

**All in one**: Direct olefin diamination with alkylamines was achieved by using a photoinduced radical‐to‐polar crossover strategy. This approach enables the programmable and sequential addition of complex amine building blocks across olefins in a single chemical transformation. This operationally simple process has broad functional‐group compatibility and enables the use of advanced building blocks for the preparation of drug‐like small molecules.

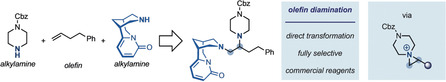

## Introduction

**Scheme 1:**
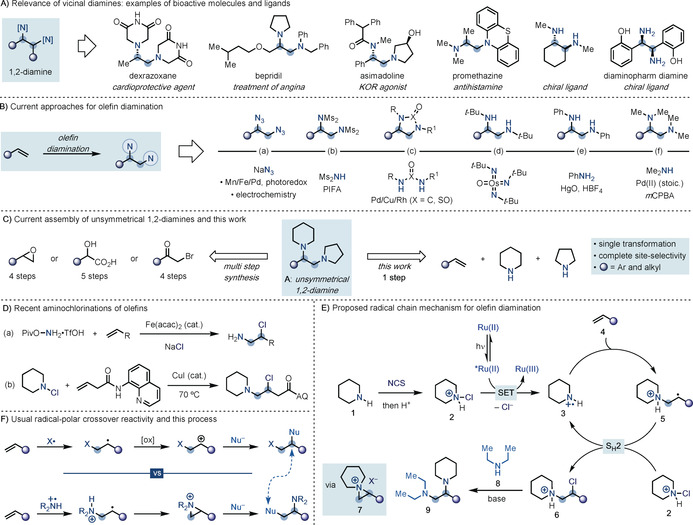
A) Vicinal diamines are high‐value materials in chemical sciences. B) Current strategies for olefin diamination do not enable the direct use of alkylamines. C) The construction of unsymmetrical diamines requires multistep syntheses while the strategy reported here assembles them in a single step. D) Recent developments for olefin aminochlorination. E) The proposed mechanism for olefin diamination involves the generation of an aminium radical to achieve an aminochlorination reaction, which is followed by aziridinium formation and regioselective ring‐opening. F) This radical‐polar crossover strategy offers a different disconnection to classical approaches.

Vicinal diamines are important building blocks in organic chemistry with applications as medicines and ligands in transition‐metal catalysis, as well as organocatalysts (Scheme 1 A).^1^ Despite their importance, the preparation of these high‐value materials is still very challenging.

Olefin diamination would represent a very attractive approach for their assembly, especially owing to the broad availability of alkenes as commercial materials from petroleum feedstocks.^2^ Indeed, several strategies have been developed to directly introduce two N‐containing functionalities across an olefin. The most general approach involves olefin diazidation, which can be promoted by a broad range of systems spanning Mn/Fe/Pd/photoredox catalysis as well as electrochemistry (Scheme 1 B, path a).^3^ Alternatively, hypervalent iodine reagents have been applied to the preparation of 1,2‐bis‐sulfonamides^4^ while Pd/Rh/Cu‐catalytic systems^5^ and electrochemistry^6^ have enabled the reaction of sulfonamides and symmetrical ureas with olefins (Scheme 1 B, path b and c, respectively).

While successful, these strategies do not enable the direct introduction of alkylamine substituents, which are a prominent class of nitrogenated motifs frequently found in the high‐value materials mentioned above. As a result, multistep synthetic sequences are still required to elaborate any of these nitrogenated intermediates into the corresponding alkylamine targets. Pioneering studies that attempted to solve this challenge involved the use of stoichiometric Os^VIII^‐imido complexes for the introduction of *t*‐BuNH_2_ units (Scheme 1 B, path d),^7^ while a restricted number of anilines have been added across alkenes with the aid of equimolar HgO^8^ or Tl(OAc)_3_ 9 under acidic conditions (Scheme 1 B, path e). In an isolated example, Bäckvall demonstrated a stoichiometric reaction between olefins, Me_2_NH, and a Pd^II^ species to produce dimethylamino palladate complexes that, upon oxidation, yielded the diaminated products (Scheme 1 B, path f).^10^

Overall, despite the success and applicability of all these methods, the construction of vicinal diamines is still synthetically very demanding. The development of a general strategy able to directly engage amines and olefins would be very desirable, especially considering that the direct construction of unsymmetrically substituted substrates has thus far been elusive (e.g. **A**, Scheme 1 C). Currently, the preparation of this class of substrates is still based on lengthy synthetic sequences, relying on functional‐group manipulation around epoxides,^11^ α‐Br‐ketones,^12^ or α‐hydroxy‐acids.^13^ Herein, we report the development of a general process for the direct and selective construction of unsymmetrical diamines wherein readily available primary and secondary amines are sequentially added with complete site‐selectivity across olefin building blocks.

### Design Plan

We have recently demonstrated how primary and secondary amines can be converted into the corresponding aminium radicals by in situ activation with *N*‐chlorosuccinimide (NCS), protonation, and photoinduced SET (single‐electron transfer) reduction.^14^

We have explored the ability of these highly electrophilic open‐shell intermediates to react selectively with a wide range of aromatic coupling partners, thus offering a platform for direct aromatic C−H amination. We recently wondered whether this reactivity profile for amine activation could be exploited in order to enable intermolecular reaction of aminium radicals with alkenes to give an olefin aminochlorination that would serve as a stepping stone to achieve a general and direct strategy leading to olefin diamination. Pioneering work by Minisci et al.^15^ and Neale et al.^16^ demonstrated the ability of preformed N‐chloroamines to engage in aminochlorination reactions with olefins. However, the requirement for harsh reaction conditions (sulfuric acid as solvent and high‐energy UV light) in addition to the known chemical instability of these reagents have limited the adoption of this approach, as well as its applicability in complex molecular assembly.

More recently Morandi has developed an Fe‐catalysed aminochlorination of olefins using *O*‐Piv hydroxylamine and NaCl as the chlorine source (Scheme 1 D, reaction a).^17^ This strategy enables the addition of a free amino group across the olefin, which requires further multistep manipulation to access any desired alkylamine. During the execution of this project, Fu has used preformed secondary *N*‐chloroamines under Cu‐catalysis to enable aminochlorination of 3‐butenoic derivatives containing an 8‐aminoquinoline (AQ) directing group required for binding and activation of the metal catalyst (Scheme 1 C, reaction b).^18^ In both reports, the isolated β‐chloroamines have been modified to yield various β‐functionalized amines. Overall, despite these advances, the direct construction of β‐chloroamines using unfunctionalized alkylamines and alkenes is still not possible and these compounds are still prepared by multistep sequences from the corresponding epoxides.^19^ We were hopeful that addressing this synthetic gap might provide a powerful entry for olefin diamination where complex alkylamines can be sequentially introduced across non‐activated olefins in a programmable manner.

As shown in Scheme 1 E, we envisioned a process where initial chlorination of an alkylamine (e.g. piperidine **1**) with NCS, followed by the addition of a strong Brønsted acid would form a highly activated *N*‐chloroammonium salt **2**. This species has a very high reduction potential (*E*_red_=+0.43 V vs. SCE)^14^ and should undergo exothermic SET reduction from the photoexcited state of a Ru(bpy)_3_^2+^ photocatalyst (*E_ox_=−0.81 V vs. SCE).^20^ This event ought to trigger a radical chain propagation where the incipient aminium radical **3** would react with the olefin coupling partner **4** with complete anti‐Markovnikov selectivity.^21^ At this point S_H_2 functionalization of the β‐ammonium radical **5** with another molecule of **2** would regenerate the chain‐carrying aminium radical **3** and provide the protonated β‐chloroamine **6**, that can undergo further ionic reactions. Indeed, since β‐chloroamines are ambiphilic building blocks, we speculated that upon addition of a base, transient aziridinium ion **7** could be accessed.19a The electrophilicity of this species should ensure a facile ring‐opening reaction by a second alkylamine (e.g., Et_2_NH, **8**) to give the desired product **9**. The high selectivity of this reaction for the less substituted site^22^ should ensure full regioselectivity in our proposal for vicinal dialkylamine synthesis. Mechanistically, it is interesting to note that we would explore umpolung radical chemistry to assemble the first C−N bond by atom‐transfer radical reaction (ATRA) reactivity and natural ionic polarity to forge the second one. Furthermore, radical chemistry would initially deliver the first amine at the most reactive terminal carbon of the olefin, to then rearrange it to the internal one upon addition of the second nucleophilic building block. This strategy therefore offers the opposite disconnectivity to what is normally observed in olefin difunctionalization through radical‐polar crossover strategies (Scheme 1 F).^23^

## Results and Discussion

### Development and Scope of Olefin Aminochlorination

**Scheme 2:**
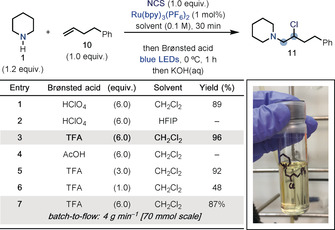
Development of the aminochlorination reaction using piperidine **1** and olefin **10**.

Optimization of the aminochlorination process started with piperidine (**1**) as the amine, 4‐phenylbutene (**10**) as the olefin building block, and NCS (Scheme 2). We were pleased to find that this reactivity could be immediately achieved in high yield by using HClO_4_ (p*K*_a_=−10) (6.0 equiv) as the Brønsted acid and Ru(bpy)_3_(PF_6_)_2_ (1 mol %) as the photocatalyst in CH_2_Cl_2_ solvent under blue‐light irradiation at 0 °C (entry 1). Interestingly, while HFIP proved to be a powerful solvent to achieve aromatic C−H amination, its use in these settings completely suppressed the desired reactivity and led to complex reaction mixtures (entry 2). Weaker acids were evaluated and while TFA (p*K*_a_=−0.25) was found to be optimum (96 % yield, entry 3), AcOH (p*K*_a_=4.76) did not lead to product formation (entry 4). In this case, since AcOH is not strong enough to promote N‐chloroamine protonation, we believe that the radical chain propagation cannot be initiated due to an endothermic SET between *Ru^II^ (*E_ox_=−0.81 V vs. SCE) and *N*‐chloropiperidine (*E*_red_=−1.80 V vs. SCE).^24^ The equivalents of TFA could be reduced to 3.0 without affecting the overall productivity of the reaction (entry 5 and 6). However, during the substrate scope evaluation, better and more consistent results were obtained with 6.0 equiv of the acid. Evidence for the efficiency of the radical chain propagation was obtained by measuring the quantum yield for the reaction, which gave *Φ*>200.

We then decided to make use of the fast nature of the aminochlorination radical chain to scale‐up the process. Using a custom‐made batch‐to‐flow reactor developed at Eli Lilly, **11** was prepared on a multigram scale by slightly changing the reaction conditions (higher concentration: 0.6 m, reduced photocatalyst loading: 0.05 mol %). The flow process proved to be a very effective solution for the scale‐up of the original reaction (0.1 mmol, 96 %, 1 hour), since it provided similar yield and excellent productivity in a much shorter time (70 mmol, 87 %, 3.75 min; productivity=16.3 mmol min^−1^; residence time=8.7 s), which could not be achieved in batch.^24^

**Scheme 3:**
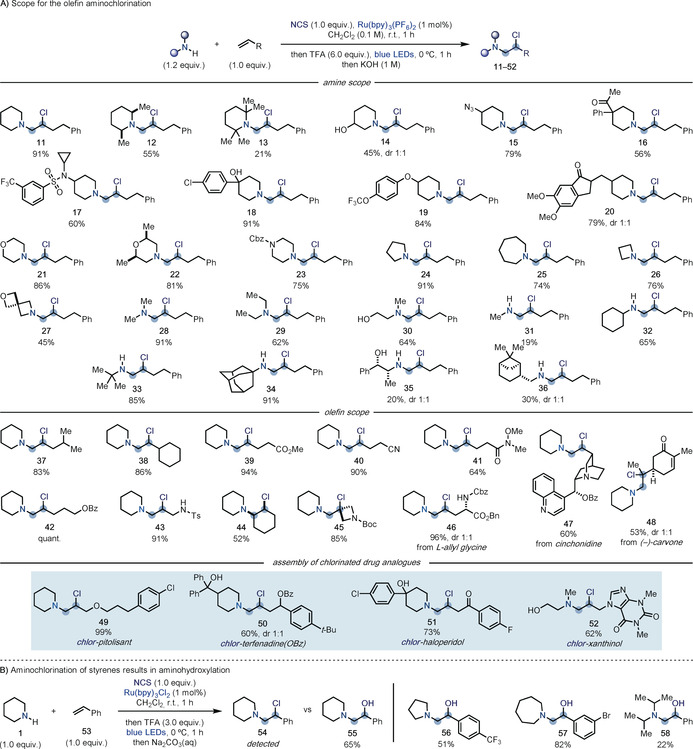
A) Substrate scope for the aminochlorination of olefins with different amine and olefin building blocks and its use to assemble chlorinated drug analogues. B) Aminochlorination of styrenes results in aminohydroxylation products.

The scope of the aminochlorination reaction proved to be general and enabled the direct installation of a broad range of secondary as well as primary amines across **10** (Scheme 3 A). We started by evaluating the use of differentially substituted piperidines and found that a variety of substituents across the N‐heterocycle core were tolerated. This included sterically encumbering methyl groups at C‐2 and C‐6 (**12** and **13**), and a C‐3 free alcohol (**14**). C‐4‐substituted piperidines are commonly found in the core structure of many opioid analgesics and antipsychotic agents such as pethidine and haloperidol. Pleasingly, we were able to engage advanced building blocks containing a C‐4 azide (**15**) which is useful in click chemistry ligation, and a benzylic quaternary centre (**16**), as well as sulfonamide (**17**), tertiary benzylic alcohol (**18**), and O‐aryl ether (**19**) units. The amine leading to product **20** is the direct precursor in the synthesis of the Alzheimer's disease drug donepezil.

Other saturated N‐heterocycles were tolerated and we successfully extended this chemistry to the introduction of morpholines (**21** and **22**), *N*‐Cbz‐piperazine (**23**), pyrrolidine (**24**), and azepane (**25**), as well as azetidine (**26**) and a spirocyclic derivative that has application as morpholine bioisostere (**27**).

Non‐cyclic secondary amines were tested next and they efficiently delivered the desired products in high yields (**28**–**30**). Finally, we evaluated the use of primary amines. While MeNH_2_ gave the desired product **31** in low yield, sterically more demanding substrates reacted smoothly providing **32**–**34** in high conversions. The chemistry was also extended to olefin functionalization using (+)‐norephedrine (**35**) and (−)‐*cis*‐myrtanylamine (**36**).

Evaluation of the olefin scope was performed using piperidine as the amine, which revealed that a wide range of alkyl substituents could be present. Alkyl groups containing activated C−H bonds for H‐atom abstraction (HAT) by aminium radicals were tolerated (**37** and **38**), as well as substrates containing inductively electron withdrawing ester (**39**), nitrile (**40**), and Weinreb amide (**41**) groups. Protected amine and alcohol functionalities were also compatible and provided the desired products **42** and **43** in high yields. Disubstituted olefins may be employed as demonstrated by the formation of **44** and **45**.

The number of functional groups tolerated by this process meant that we were able to successfully use it for the late‐stage aminochlorination of a protected l‐allyl glycine residue (**46**), a protected cinchonidine alkaloid (**47**), and the terpene (−)‐carvone (**48**). In this last example, the two olefins are electronically different, which ensured complete chemoselectivity for the functionalization of the electron‐rich one.

To further demonstrate the utility of this amination strategy, we sought to adapt it to the direct assembly of sp^3^‐chlorinated drug analogues. By using readily available and inexpensive amines and olefin building blocks, we were able to rapidly access a representative selection of drug analogues of pitolisant (**49,** treatment of narcolepsy), terfenadine (**50,** anti‐allergic), haloperidol (**51,** antipsychotic), and xanthinol (**52**, vasodilator). This reactivity enabled the direct construction of the drug cores in one step and also the installation of a chlorine atom at a specific sp^3^ site, which can then be used as handle for further modification.

When evaluating the olefin scope, we realized that styrene **53** displayed a slightly different reactivity profile (Scheme 3 B). While aminochlorination (**54**) could be achieved as demonstrated by crude LC–MS analysis, the more activated nature of the benzylic chloride meant that, upon basic work up, full hydrolysis to the amino alcohol **55** took place. The process was extended to both cyclic and acyclic amines with differentially substituted styrene partners to give amino alcohols **56**–**58**. Overall, this reactivity provides direct access to aminohydroxylated building blocks that are otherwise still prepared through olefin epoxidation followed by nucleophilic substitution.^25^

### Development and Scope of Olefin Diamination

**Scheme 4:**
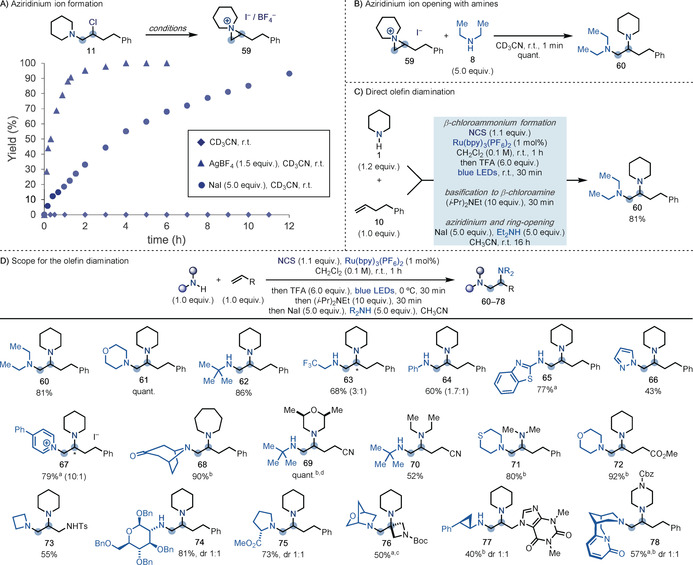
A) Reaction profiles for the formation of aziridinium **59** from **11**. B) Aziridinium ring opening with Et_2_NH **8** is immediate. C) Development of a procedure for direct olefin diamination. D) Substrate scope for the direct olefin diamination using a range of different amine and olefin building blocks. [a] A basic work‐up was performed before addition of NaI and the amine. [b] Upon addition of NaI and the amine, the mixture was warmed to 60 °C. [c] In this case the aziridinium was formed using Ag(BF_4_). [d] In this case the N‐chloroamine was purified before addition of NaI.* Denotes the minor constitutional isomer.

Having developed a versatile and general process for olefin aminochlorination using a broad range of alkylamines, we decided to validate its implementation as part of a strategy leading to an overall diamination. Key to the success of this proposal is the ability of the β‐chloroamine intermediates to undergo aziridinium ion formation followed by regioselective nucleophilic ring‐opening. Since the β‐chloroamine substrates were found to be stable at room temperature, we performed initial experiments to identify conditions that could then be adapted for in situ aziridinium ion formation. As shown in Scheme 4 A, while **11** was stable in CD_3_CN at room temperature, efficient formation of aziridinium **59** was achieved by addition of either AgBF_4_ 26 (1.5 equiv) or NaI (5.0 equiv). In the case of NaI, the reaction presented a slightly slower profile but complete conversion could be achieved in an 18 h time window. With **59** in hand, we initially evaluated the ability of a secondary amine to promote regioselective ring‐opening at the less substituted carbon (Scheme 4 B). Pleasingly, upon addition of Et_2_NH **8** (5.0 equiv), immediate formation of the desired product **60** as a single regioisomer was observed.^24^

Having validated the conversion of the β‐chloroamine **11** into the corresponding aziridinium ion **59** and subsequent nucleophilic ring‐opening with a secondary amine (**59**+**8**→**60**), we were keen to adapt this approach to a one‐pot procedure. As shown in Scheme 4 C, we identified an optimum procedure starting with the initial aminochlorination step (see Scheme 2, entry 3), followed by basification with (*i*‐Pr)_2_NEt (10 equiv) and final in situ aziridinium formation/ring‐opening reaction by addition of NaI (5.0 equiv) and Et_2_NH (5.0 equiv) as a solution in CH_3_CN. This procedure provided **60** in excellent yield, and importantly, streamlined its preparation from the current 5‐step synthesis to a single chemical operation that took 19 h overall.^27^

With this optimized procedure in hand, we focused our efforts on establishing the scope for the olefin diamination reaction (Scheme 4 D). Using piperidine as the aminium radical precursor and **10** as the olefin, we then evaluated several other N‐nucleophiles. Pleasingly, we were able to use secondary cyclic (**61**) as well as primary amines of both high steric hindrance (**62**) and decreased nucleophilicity (**63** and **64**). In just these two cases over the entire scope, the products were respectively obtained as 3:1 and 1.7:1 mixtures of isomers resulting from an unselective aziridinium ring‐opening, while benzothiazole‐2‐amine gave selectively **65**. Pleasingly, we were also able introduce aromatic N‐heterocycles like pyrazole and 4‐Ph‐pyridine that gave the desired product **66** and **67** in good yield. In the case of **65** and **67**, a basic work‐up at the end of the aminochlorination step before aziridinium formation was crucial in obtaining high reaction yield.

Other unsymmetrical 1,2‐diamines were assembled by generating the aminium radical from various N‐heterocycles like azepane and *syn*‐2,6‐dimethylmorpholine that, following aziridinium ring‐opening with nortropinone and *t*‐BuNH_2_, gave **68** and **69** in excellent yields. Acyclic amines were also amenable to this reactivity as demonstrated by the sequential introduction of Et_2_NH and *t*‐BuNH_2_ on 4‐pentenenitrile (**70**), whilst Me_2_NH and thiomorpholine also gave **71** in high yields. Ester‐containing olefins were compatible with the reactivity (**72**), and by using *N*‐Ts‐allylamine as the alkene, we prepared the completely differentiated 1,2,3‐triamine **73** in high yield.

More complex building blocks were accessed by using a protected glucosamine and l‐proline methyl ester in the ring‐opening event that gave **74** and **75** respectively (1:1 mixture of epimers). Implementation of 3‐methylene‐*N*‐Boc‐azetidine as the olefin, piperidine as the aminium radical precursor, and 2‐oxa‐5‐azobicyclo[2.2.1]heptane as the final nucleophile gave **76** in good yield, thus demonstrating that aziridinium formation at tertiary centres is possible. A theophylline‐containing olefin was reacted with piperidine and the MAO inhibitor tranylcypromine to give **77** in good yield (1:1 mixture of epimers). Finally, by sequentially using *N*‐Cbz‐piperazine (aminium radical) and the biologically active alkaloid cytisine (nucleophilic amine), we prepared **78** in high yield (1:1 mixture of epimers).

**Scheme 5:**
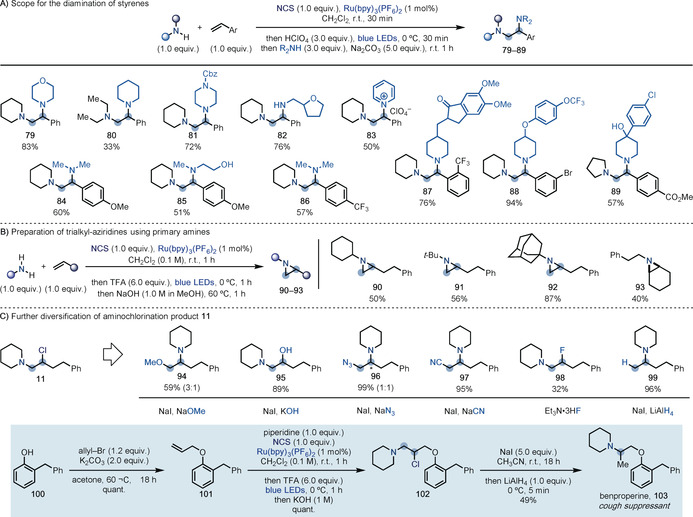
A) Styrene diamination provides a different regioselectivity. B) The use of primary amines enables the preparation of trialkyl aziridines. C) Further diversification of β‐chloroamine and use of the reduction process for the 3‐step synthesis of benproperine. [a] A basic work‐up was performed before addition of NaI and the amine. [b] Upon addition of NaI and the amine the mixture was warmed to 60 °C. [c] In this case the aziridinium was formed using Ag(BF_4_). * Denotes the minor constitutional isomer.

We also found that the diamination could be applied to a broad range of styrene building blocks (Scheme 5 A). In this case however, addition of NaI to aid aziridinium ion formation was not required due to the more activated nature of the analogous benzylic β‐chloroamine.^28^ In line with the observed aminohydroxylation reactivity, we found that the addition of the second amine nucleophile in the presence of Na_2_CO_3_ at the end of the aminochlorination step immediately provided the corresponding 1,2‐diamines. Importantly, in this case the substitution occurred at the more activated benzylic position, which is in line with the known reactivity of aryl‐substituted aziridiniums. This process was compatible with the use of secondary (**79**–**81**) and primary amines (**82**), as well as pyridine, which led to the isolation of the corresponding salt **83** in moderate yield. More advanced amine building blocks were easily introduced and the chemistry was also expanded to both electron‐rich (**84** and **85**) and electron‐poor (**86** and **89**) styrenes and tolerated *ortho* (**87**) as well as *meta* (**88**) substituents.

When primary amines were used, the aminochlorination occurred smoothly, and upon basification a cyclization occurred to give the corresponding and stable aziridine, which could not be ring‐opened in situ (Scheme 5 B). This procedure allowed the introduction of amines of increasing steric hindrance (**90**–**92**) and also worked well on disubstituted olefins (**93**). Overall, this reactivity enabled one‐step access to all‐alkyl substituted aziridines, which are an underrepresented class of building blocks that are difficult to prepare using nitrene‐based approaches and still require multistep sequences.^29^

The initial aminochlorination process represents an interesting gateway to expand the number of potential products of aminofunctionalization. Since aziridinium ions support a rich array of chemistry, we have been able to engage **11** in several other processes (Scheme 5 C). Oxygen nucleophiles were competent partners, as demonstrated by the use of NaOMe and NaOH, which led to aminohydroxylated products **94** and **95** with opposite selectivity in the ring‐opening step. NaN_3_ could be used to access the vicinal amino azide **96**, albeit as a mixture of isomers (unselective aziridinium ion ring‐opening). NaCN enabled selective ring‐opening, resulting in the formation of a C−C bond to deliver **97**, which has potential for application to the synthesis of β‐aminoacids. Vicinal fluoroamines are important and highly sought‐after building blocks in medicinal chemistry19b, 30 but we did not succeed in identifying reaction conditions for aziridinium ring‐opening with fluoride sources. Nevertheless, we found that direct addition of Et_3_N⋅3 HF to **11** provided **98** in moderate yield and as a single isomer. Finally, addition of LiAlH_4_ at 0 °C enabled efficient reduction (**99**), which gives direct access to challenging products of Markovnikov hydroamination that are currently prepared by reductive amination on ketones.^31^ We showcased the potential utilization of this reactivity in the preparation of the cough suppressant benproperine (**103**). In this case, the commercial phenol **100** was allylated to give **101**, which underwent efficient aminochlorination with piperidine (**102**, quant.). Aziridinium ion formation and selective LiAlH_4_ reduction gave benproperine in just 3 steps.

## Conclusion

Direct synthesis of vicinal alkyl diamines from olefins has been a long‐standing challenge and still requires multistep synthesis. In this work, we developed a photoinduced method for their efficient and selective assembly. This strategy exploits the generation of aminium radicals from in situ generated N‐chloroamines and their ability to react with a broad range of olefins with *anti*‐Markovnikov selectivity. The resulting β‐chloroamines are powerful ambiphilic building blocks for further elaboration, as demonstrated by their direct conversion into the corresponding aziridinium ions. The following in situ ring‐opening reaction with primary, secondary, and aromatic amine nucleophiles allowed regioselective diamination.

Overall this reactivity enables, for the first time, the direct and selective introduction of advanced amine building blocks across olefins in a single chemical operation. The process tolerates a broad range of functionalities, does not require the presence of any directing group, and is scalable using batch‐to‐flow technology. We believe that these results could substantially broaden the construction of valuable nitrogenated small‐molecule building blocks that are central to pharmaceutical, agrochemical, and chemical sciences.

## Conflict of interest

The authors declare no conflict of interest.

## Supporting information

As a service to our authors and readers, this journal provides supporting information supplied by the authors. Such materials are peer reviewed and may be re‐organized for online delivery, but are not copy‐edited or typeset. Technical support issues arising from supporting information (other than missing files) should be addressed to the authors.

Supplementary
