# Supplementary material for: Photoinduced Olefin Diamination with Alkylamines
Source: Angew Chem Int Ed Engl. 2020 Jun 9;59(35):15021–8. doi: 10.1002/anie.202005652 (PMC7497254; doi:10.1002/anie.202005652)
Supplement: Supplementary file 1 — Supplementary [file ANIE-59-15021-s001.pdf]

Angewandte Chemie
Eine Zeitschrift der Gesellschaft Deutscher Chemiker

Supporting Information

Photoinduced Olefin Diamination with Alkylamines

Sebastian Govaerts[+], Lucrezia Angelini[+], Charlotte Hampton, Laia Malet-Sanz, Alessandro Ruffoni, and Daniele Leonori**

anie_202005652_sm_miscellaneous_information.pdf

1 Table of Contents

2 General Experimental Details 3

3 Starting Material Synthesis 4

4 Olefin Aminochlorination 10

4.1 Reaction Optimization 10
4.2 Substrate Scope 11
4.3 Mechanistic Considerations 31
4.3.1 Protonation of *N*-Chloropiperidine 31
4.3.2 Quantum Yield Determination 32

5 Aziridinium Formation and Ring-Opening Studies 33

5.1 Aziridinium Formation 33
5.2 Aziridinium Ring-Opening 35

6 Olefin Diamination 36

6.1 Reaction Optimization 36
6.2 Reaction Scope 37

7 Aminohydroxylation and Diamination of Styrenes 49

7.1 Aminohydroxylation – Substrate Scope 49
7.2 Diamination – Substrate Scope 51

8 Olefin Aziridination 58
8.1 Substrate Scope 58
9 Diversification of β-Chloroamines 61
9.1 Substrate Scope 61
10 Olefin Aminochlorination Scale-Up by Batch-to-Flow 66
10.1 General Experimental Details 66
10.2 General Flow Procedure 67
11 NMR Spectra 69
12 References 167

2 General Experimental Details

All required fine chemicals were used directly without purification unless stated otherwise. All air and moisture sensitive reactions were carried out under nitrogen atmosphere using standard Schlenk manifold technique. THF was distilled from sodium/benzophenone, CH_2Cl_2 and was distilled from CaH_2, CH_3CN was distilled from activated 4Å molecular sieves, Et_3N was distilled over KOH. ^1H and ^{13}C Nuclear Magnetic Resonance (NMR) spectra were acquired at various field strengths as indicated and were referenced to $CHCl_3$ (7.27 and 77.0 ppm for ^1H and ^{13}C respectively). ^1H NMR coupling constants are reported in Hertz and refer to apparent multiplicities and not true coupling constants. Data are reported as follows: chemical shift, integration, multiplicity (s = singlet, br s = broad singlet, d = doublet, t = triplet, q = quartet, p = pentet, sx = sextet, sp = septet, m = multiplet, dd = doublet of doublets, etc.), proton assignment (determined by 2D NMR experiments: COSY, HSQC and HMBC) where possible. High-resolution mass spectra were obtained using a JEOL JMS-700 spectrometer or a Fissions VG Trio 2000 quadrupole mass spectrometer. Spectra were obtained using electron impact ionization (EI) and chemical ionization (CI) techniques, or positive electrospray (ES). Infra-red spectra were recorded using a JASCO FT/IR 410 spectrometer, ATI Mattson Genesis Seris FTIR or Bruker Alpha-P spectrometer as evaporated films or liquid films. Analytical TLC: aluminum backed plates pre-coated (0.25 mm) with Merck Silica Gel 60 F254. Compounds were visualized by exposure to UV-light or by dipping the plates in ninhydrin stain followed by heating. Flash column chromatography was performed using Merck Silica Gel 60 (40–63 μm). All mixed solvent eluents are reported as v/v solutions. UV/Vis spectra were obtained using an Agilent 6453 spectrometer and 1 mm High Precision Cell made of quartz from Hellma Analytics. The LEDs used are Kessil H150-blue. All the reactions were conducted in CEM 10 mL glass microwave tubes.

3 Starting Material Synthesis

N-Methoxy-*N*-methylpent-4-enamide (S1)

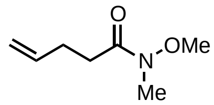

A mixture of 100 mL *N*,*O*-dimethylhydroxylamine hydrochloride (0.90 g, 9.27 mmol, 1.1 equiv.), CH_2Cl_2 (20 mL) and pyridine (1.70 mL, 21.0 mmol, 2.5 equiv.) was cooled to 0 °C, treated with pent-4-enoyl chloride (1.0 g, 8.43 mmol, 1.0 equiv.), warmed to room temperature and stirred for 4 h. The mixture was diluted with EtOAc (30 mL), and washed with 2 N aqueous HCl (20 mL) and brine (20 mL). The organic layer was dried ($MgSO_4$), filtered and evaporated to give **S1** (1.15 g, 95%) as an oil. ^{1}H NMR (400 MHz, $CDCl_3$) δ 5.82 (1H, ddt, *J* = 17.2, 10.0, 6.4 Hz), 5.03 (1H, ddt, *J* = 17.2, 1.7, 1.6 Hz), 4.95 (1H, ddt, *J* = 10.0, 1.6, 1.4 Hz), 3.65 (3H, s), 3.14 (3H, s), 2.49 (2H, t, *J* = 7.6 Hz), 2.40–2.29 (2H, m). Data in accordance with literature.[1]

Pent-4-en-1-yl Benzoate (S2)

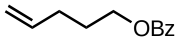

A solution of 4-pentenol (0.6 g, 7.0 mmol, 1.0 equiv.) and TMEDA (0.6 mL, 4.17 mmol, 0.6 equiv.) in CH_2Cl_2 (20 mL) was cooled to –78 °C, treated with BzCl (0.90 mL, 7.65 mmol, 1.1 equiv.) and allowed to warm to room temperature overnight. Aqueous 1 M KOH (20 mL) was added, the layers were separated and the aqueous layer was extracted with CH_2Cl_2 (10 mL x 3). The combined organic layers were dried ($MgSO_4$), filtered and evaporated. Purification by column chromatography on silica gel eluting petrol–EtOAc (99:1) gave **S2** (0.96 g, 75%) as an oil. ^{1}H NMR (400 MHz, $CDCl_3$) δ 8.14–7.96 (2H, m), 7.63–7.50 (1H, m), 7.49–7.40 (2H, m), 5.85 (1H, ddt, *J* = 16.9, 10.2, 6.6 Hz), 5.08 (1H, ddt, *J* = 17.1, 1.6, 1.4 Hz), 5.02 (1H, ddt, *J* = 10.2, 1.6, 1.4 Hz), 4.34 (2H, t, *J* = 6.6 Hz), 2.32–2.15 (2H, m), 1.94–1.80 (2H, m). Data in accordance with literature.[2]

N-Allyl-4-methylbenzenesulfonamide (S3)

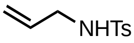

A solution of TsCl (1.05 g, 5.5 mmol, 1.1 equiv.) in CH_2Cl_2 (5 mL) was cooled to 0 °C and treated with Et_3N (0.74 mL, 5.3 mmol, 1.05 equiv.) and allylamine (0.37 mL, 5.0 mmol, 1.0 equiv.). The mixture was allowed to warm to room temperature overnight.

Aqueous 1 M HCl (5 mL) was added and the layers were separated. The aqueous layer was extracted with CH_2Cl_2 (10 mL x 3). The combined organic layers were dried ($MgSO_4$), filtered and evaporated to give **S3** (1.1 g, quantitative) as a solid. ^{1}H NMR (400 MHz, $CDCl_3$) δ 7.85–7.68 (2H, m), 7.41–7.20 (2H, m), 5.85–5.64 (1H, m), 5.26–5.05 (2H, m), 4.62 (1H, br s), 3.67–3.58 (2H, m), 2.61–2.22 (3H, m). Data in accordance with literature.[3]

Benzyl (*S*)-2-(((benzyloxy)carbonyl)amino)pent-4-enoate (S4)

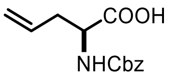

A solution of L-allyl glycine (0.50 g, 4.34 mmol, 1.0 equiv.), and $NaHCO_3$ (1.02 g, 12.1 mmol, 2.8 equiv.) in H_2O (10 mL) was treated with CbzCl (0.93 mL, 6.51 mmol, 1.5 equiv.) and stirred for 4 h. Et_2O (10 mL) was added and the layers were separated. The aqueous layer was acidified with 1 N aqueous HCl (20 mL) and extracted with Et_2O (30 mL x 3). The combined organic layers were dried ($MgSO_4$), filtered and evaporated to give **S4** (0.62 g, 56%) as an oil. ^{1}H NMR (400 MHz, $CDCl_3$): δ 9.72 (1H, br s), 7.44–7.27 (5H, m), 5.72 (1H, ddt, *J* = 16.9, 10.3, 7.2 Hz), 5.36 (1H, d, *J* = 8.2 Hz), 5.23–5.05 (4H, m), 4.50 (1H, dt, *J* = 8.0, 5.8 Hz), 2.71–2.38 (2H, m). Data in accordance with literature.[4]

Benzyl (*S*)-2-(((benzyloxy)carbonyl)amino)pent-4-enoate (S5)

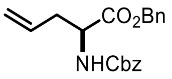

A solution of **S4** (0.62 g, 2.49 mmol, 1.0 equiv.) and K_2CO_3 (0.516 g, 3.75 mmol, 1.5 equiv.) in DMF (5 mL) was treated with BnBr (0.44 mL, 3.75 mmol, 1.5 equiv.) and heated under reflux for 18 h. The mixture was cooled to room temperature and diluted with H_2O (10 mL) and EtOAc (10 mL). The layers were separated and the aqueous layer was extracted with EtOAc (10 mL x 3). The combined organic layers were washed with brine (10 mL), dried ($MgSO_4$), filtered and evapoarted. Purification by column chromatography on silica gel, eluting with petrol–EtOAc (9:1), gave **S5** (0.66 g, 79%) as an oil. ^{1}H NMR (400 MHz, $CDCl_3$) δ 7.47–7.27 (10H, m), 5.64 (1H, ddt, *J* = 17.5, 10.6, 7.2 Hz), 5.32 (1H, d, *J* = 8.2 Hz), 5.25–5.12 (2H, m), 5.13–5.02 (4H, m), 4.51 (1H, dt, *J* = 8.2, 5.8 Hz), 2.67–2.41 (2H, m). Data in accordance with literature.[5]

(*R*)-Quinolin-4-yl((*1S,2R,4S,5R*)-5-vinylquinuclidin-2-yl)methyl Benzoate (S6)

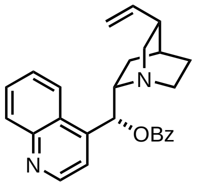

A solution of cinchonidine (2.0 g, 6.8 mmol, 1.0 equiv.) and TMEDA (0.61 mL, 4.10 mmol, 0.6 equiv.) in CH_2Cl_2 (40 mL) was cooled to –78 °C, treated with BzCl (0.87 mL, 7.5 mmol, 1.1 equiv.) and allowed to warm to room temperature overnight. The mixture was diluted with aqueous 1 M KOH (20 mL), the layers were separated and the aqueous layer was extracted with CH_2Cl_2 (3 x 20 mL). The combined organic layers were dried ($MgSO_4$), filtered and evaporated. Purification by column chromatography on silica gel, eluting with CH_2Cl_2–MeOH–37% aq. NH_3 (97:3:0.5), gave **S6** (2.2 g, 81%) as a foam. R_f 0.40 [CH_2Cl_2:MeOH 97:3]; FT-IR: ν_{max} (film)/cm^{-1} 2940, 2862, 1718, 1266; ^{1}H NMR (500 MHz, $CDCl_3$) δ 8.87 (1H, d, *J* = 4.5 Hz), 8.33 (1H, d, *J* = 8.5 Hz), 8.14 (1H, d, *J* = 8.4 Hz), 8.12–8.06 (2H, m), 7.72 (1H, ddd, *J* = 8.4, 6.7, 1.3 Hz), 7.63 (1H, ddd, *J* =8.4, 6.7, 1.4 Hz), 7.60–7.55 (1H, m), 7.47 (3H, dd, *J* = 8.7, 6.4 Hz), 6.80 (1H, d, *J* = 6.8 Hz), 5.84 (1H, ddd, *J* = 17.5, 10.3, 7.4 Hz), 5.05–4.95 (2H, m), 3.50 (1H, q, *J* = 7.5 Hz), 3.22 (1H, dddd, *J* = 13.4, 10.3, 5.5, 2.5 Hz), 3.07 (1H, dd, *J* = 13.9, 10.1 Hz), 2.72–2.59 (2H, m), 2.35–2.22 (1H, m), 2.00–1.92 (1H, m), 1.91–1.86 (1H, m), 1.81–1.66 (2H, m), 1.57 (1H, dddt, *J* = 13.4, 11.0, 5.6, 2.9 Hz); ^{13}C NMR (126 MHz, $CDCl_3$) δ 165.6, 150.1, 148.8, 145.5, 141.8, 133.5, 130.6, 129.9, 129.8, 129.3, 128.7, 127.0, 126.1, 123.4, 118.6, 114.6, 74.8, 60.1, 56.9, 42.6, 39.8, 28.0, 27.8, 24.4; HRMS (ESI$^+$): Found M$^+$ 398.1986, $[C_{26}H_{26}N_2O_2]^+$ requires 398.1994.

1-(3-(Allyloxy)propyl)-4-chlorobenzene (S7)

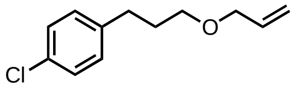

A suspension of NaH (0.47 g, 11.7 mmol, 2.0 equiv., 60% wt. in mineral oil) in THF (10 mL) was cooled to 0 °C and treated with 3-(4-chlorophenyl)propanol (1.0 g, 5.86 mmol, 1.0 equiv.). The mixture was stirred for 1 h and, treated with allyl bromide (0.60 mL, 7.0 mmol, 1.2 equiv.) and allowed to warm to room temperature overnight. The mixture was cooled to 0 °C and diluted with saturated aqueous NH_4Cl (10 mL). Et_2O (10 mL) was added, the layers were separated and the aqueous layer was extracted with Et_2O (10 mL x 3). The combined organic layers were dried ($MgSO_4$), filtered and

evaporated **S7** (1.14 g, 93%) as an oil. FT-IR: ν_{max} (film)/cm^{-1} 2935, 2857, 1491, 1090; ^{1}H NMR (400 MHz, $CDCl_3$) δ 7.29–7.21 (2H, m), 7.16–7.07 (2H, m), 5.93 (1H, ddt, *J* = 17.2, 10.3, 5.6 Hz), 5.28 (1H, ddt, *J* = 17.2, 1.7, 1.6 Hz), 5.18 (1H, ddt, *J* = 10.4, 1.6, 1.5 Hz), 3.96 (2H, dt, *J* = 5.6, 1.4 Hz), 3.43 (2H, t, *J* = 6.3 Hz), 2.68 (2H, t, *J* = 7.5 Hz), 1.96–1.79 (2H, m); ^{13}C NMR (101 MHz, $CDCl_3$) δ 140.5, 135.0, 131.6, 130.0, 128.5, 117.0, 72.0, 69.3, 31.8, 31.4; HRMS (ESI$^+$): found MH$^+$ 211.0882, $[C_{12}H_{16}ClO]^+$ requires 211.0982.

1-(4-(*tert*-Butyl)phenyl)but-3-en-1-ol (S8)

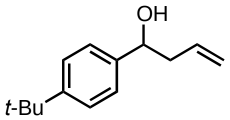

A solution of *tert*-butyl benzaldehyde (1.67 mL, 10.0 mmol, 1.0 equiv.) in THF (25 mL) was cooled to 0 °C and treated with allyl–MgBr (12.0 mL, 12.0 mmol, 1.2 equiv., 1 M in Et_2O). The mixture was allowed to warm to room temperature overnight, quenched with saturated aqueous NH_4Cl solution (20 mL) and extracted with Et_2O (30 mL x 3). The organic phase was dried ($MgSO_4$), filtered and evaporated to give **S8** (2.1 g, quantitative) as an oil. ^{1}H NMR (400 MHz, $CDCl_3$) δ 7.43–7.35 (2H, m), 7.33–7.28 (2H, m), 5.84 (1H, ddt, *J* = 17.2, 10.2, 7.1 Hz), 5.25–5.04 (2H, m), 4.78–4.62 (1H, m), 2.59–2.45 (2H, m), 2.04 (1H, br s), 1.33 (9H, s). Data in accordance with literature.[6]

1-(4-(*tert*-Butyl)phenyl)but-3-en-1-yl Benzoate (S9)

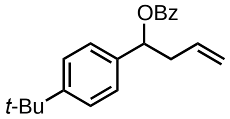

A solution of **S8** (719 mg, 3.52 mmol, 1.0 equiv.) and TMEDA (0.32 mL, 2.11 mmol, 0.6 equiv.) in CH_2Cl_2 (5 mL) was cooled to –78 °C and treated with BzCl (0.60 mL, 3.87 mmol, 1.1 equiv.). The mixture was allowed to warm to room temperature overnight. Aqueous 1 M KOH (5 mL) was added, the layers were separated, and the aqueous layer was extracted with CH_2Cl_2 (5 mL x 3). The combined organic layers were dried ($MgSO_4$), filtered and evaporated. Purification by column chromatography on silica gel eluting with petrol–EtOAc (98:2) gave **S9** (0.80 g, 74%) as an oil. FT-IR: ν_{max} (film)/cm^{-1} 2961, 1716, 1266; ^{1}H NMR (400 MHz, $CDCl_3$) δ 8.10 (2H, d, *J* = 7.5 Hz), 7.56 (1H, t, *J* = 7.3 Hz), 7.43 (2H, t, *J* = 7.6 Hz), 7.42–7.35 (4H, m), 6.06 (1H, dd, *J* = 7.9, 5.6 Hz), 5.81 (1H, ddt, *J* = 17.1, 10.2, 6.9 Hz), 5.15 (1H, dd, *J* = 17.4, 1.5 Hz),

5.07 (1H, dd, *J* = 10.3 Hz, 1.1 Hz), 2.89–2.76 (1H, m), 2.75–2.66 (1H, m), 1.31 (9H, s); ^{13}C NMR (126 MHz, $CDCl_3$) δ 165.8, 150.9, 137.2, 133.6, 133.0, 130.6, 129.7, 128.4, 126.3, 125.5, 118.1, 75.7, 41.0, 34.6, 31.4. HRMS (ESI$^+$): found M^+ 308.1757, $[C_{21}H_{24}O_2]^+$ requires 308.1776.

1-(4-Fluorophenyl)but-3-en-1-ol (S10)

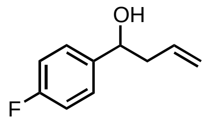

A solution of 4-fluorobenzaldehyde (0.87 mL, 8.0 mmol, 1.0 equiv.) in THF (10 mL) was cooled to 0 °C and treated with allyl–MgCl (5.0 mL, 10.0 mmol, 1.25 equiv., 2 M in THF). The mixture was allowed to warm to room temperature and monitored by TLC until completion (2 h). Saturated aqueous NH_4Cl solution (10 mL) and Et_2O (20 mL) were added. The layers were separated and the aqueous layer was extracted with Et_2O (3 x 20 mL). The combined organic layers were dried ($MgSO_4$), filtered and evaporated. Purification by column chromatography on silica gel eluting with petrol–EtOAc (97:3) gave **S10** (1.2 g, 86%) as an oil. ^{1}H NMR (400 MHz, $CDCl_3$) δ 7.44–7.29 (2H, m), 7.19–6.97 (2H, m), 5.79 (1H, dddd, *J* = 17.0, 10.5, 7.6, 6.6 Hz), 5.21–5.15 (1H, m), 5.14 (1H, d, *J* = 1.2 Hz), 4.73 (1H, dd, *J* = 7.7, 5.4 Hz), 2.61–2.39 (3H, m). Data in accordance with literature.[7]

1-(4-Fluorophenyl)but-3-en-1-one (S11)

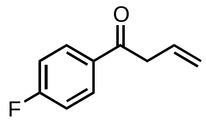

To a solution of **S10** (482 mg, 2.9 mmol) in CH_2Cl_2 (15 mL) was added Celite (0.8 g) followed by PCC (935 mg, 4.35 mmol, 1.5 equiv.). The mixture was stirred at room temperature for 4 h, and then filtered through a short pad Celite washing with CH_2Cl_2 (20 mL). The combined filtrates were evaporated and the residue was purified by column chromatography on silica gel eluting petrol–EtOAc (90:10) to give **S11** (341 mg, 73%) as an oil. ^{1}H NMR (400 MHz, $CDCl_3$) δ 8.07–7.93 (2H, m), 7.21–7.05 (2H, m), 6.07 (1H, ddt, *J* = 17.0, 10.3, 6.7 Hz), 5.32–5.15 (2H, m), 3.82–3.65 (2H, m). Data in accordance with literature. [7]

7-Allyl-1,3-dimethyl-3,7-dihydro-*1H*-purine-2,6-dione (S12)

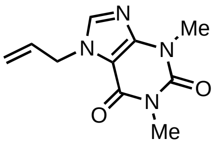

A suspension of theophylline (810 mg, 4.5 mmol, 1.0 equiv.) and K_2CO_3 (700 mg, 5.0 mmol, 1.1 equiv.) in DMF (8 mL, 0.6 M) was treated with allyl bromide (430 µL, 5.0 mmol, 1.1 equiv.) at room temperature. The mixture was warmed to 40 °C, stirred for 4 h and then cooled to room temperature. The crude was diluted with EtOAc (20 mL) and H_2O (20 mL). The layers were separated and the aqueous layer was washed with EtOAc (3 x 20 mL). The combined organic layers were dried ($MgSO_4$), filtered and evaporated to give **S12** (600 mg, 60%) as a solid. ^{1}H NMR (400 MHz, $CDCl_3$) δ 7.56 (1H, s), 6.05 (1H, ddt, *J* = 16.4, 11.0, 5.8 Hz), 5.42–5.15 (2H, m), 4.95 (2H, d, *J* = 5.9 Hz), 3.60 (3H, s), 3.41 (3H, s). Data in accordance with literature.[8]

1-(Allyloxy)-2-benzylbenzene (101)

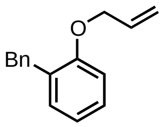

A suspension of 2-benzylphenol (1.00 g, 5.43 mmol, 1.0 equiv.) and K_2CO_3 (2.25 g, 16.28 mmol, 3.0 equiv.) in acetone (40 mL) was stirred at room temperature for 30 min, treated with allyl bromide (0.70 mL, 8.15 mmol, 1.5 equiv.) and heated under reflux for 12 h. The mixture was evaporated, diluted with EtOAc (20 mL) and aqueous 1 M KOH (20 mL). The layers were separated and the aqueous layer was extracted with EtOAc (3 x 20 mL). The combined organic layers were washed with brine (10 mL), dried ($MgSO_4$), filtered and evaporated to give **101** (1.22 g, quantitative) as an oil. ^{1}H NMR (400 MHz, $CDCl_3$) δ 7.31–7.21 (4H, m), 7.29–7.20 (2H, m), 7.08 (1H, dd, *J* = 7.5, 1.7 Hz), 6.92–6.79 (2H, m), 6.01 (1H, ddt, *J* = 17.3, 10.3, 5.0 Hz), 5.36 (1H, dd, *J* = 17.3, 1.8 Hz), 5.23 (1H, dd, *J* = 10.5, 1.7 Hz), 4.52 (2H, d, *J* = 5.1), 4.00 (2H, s). Data in accordance with literature.[9]

4 Olefin Aminochlorination

4.1 Reaction Optimization

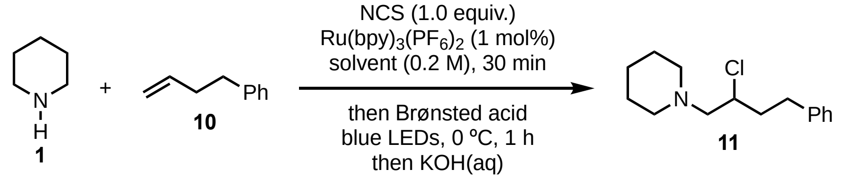

A tube equipped with a stirring bar was charged with $Ru(bpy)_3(PF_6)_2$ (1.0 mg, 1.0 μmol, 1 mol%) and *N*-chlorosuccinimide (NCS). The tube was capped with a Supelco aluminium crimp seal with septum (PTFE/butyl), evacuated and refilled with N_2 (x 3). Piperidine **1** (12 μL, 0.12 mmol, 1.2 equiv.) and the solvent (0.5 mL, 0.2 M, dry and degassed by bubbling through with N_2 for 20 min) were added and the mixture was stirred in the dark for 1 h at room temperature. 4-Phenyl-1-butene **10** was added along with an additional 0.5 mL of the same solvent, followed by the acid. The blue LEDs were immediately switched on and the mixture was stirred under irradiation for 1 h. KOH (1.0 M, 3 mL) and EtOAc (3 mL) were added and the mixture was shaken vigorously. 1,3,5-Trimethoxybenzene (17 mg, 0.1 mmol, 1.0 equiv.) was added and the layers were separated. The aqueous layer was extracted with EOAc (x3), the combined organic layers were dried ($MgSO_4$), filtered and evaporated. $CDCl_3$ (0.4 mL) was added and the mixture was analysed by ^{1}H NMR spectroscopy to determine the NMR yield. Table **S1** reports all the experiments performed.

Table S1.

Entry	Bronsted acid (equiv.)	Solvent	Yield (%)
**1**	$HClO_4$	CH_2Cl_2	89
**2**	$HClO_4$	HFIP	–
**3**	TFA	CH_2Cl_2	96
**4**	AcOH	CH_2Cl_2	–
**5**	TFA	CH_2Cl_2	92
**6**	TFA	CH_2Cl_2	48
**7**	–	CH_2Cl_2	–
**8**[a]	TFA	CH_2Cl_2	37
**9**[b]	TFA	CH_2Cl_2	–

a) Reaction was run without $Ru(bpy)_3(PF_6)$. b) Reaction was run in the dark

4.2 Substrate Scope

General Procedure for the Olefin Aminochlorination Using Free Amines – GP1

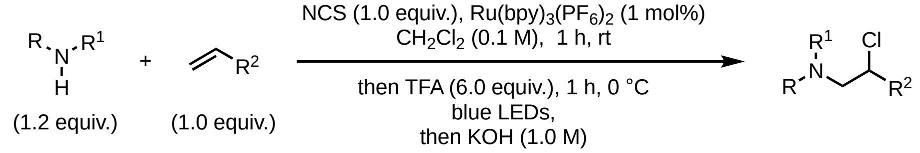

A dry tube equipped with a stirring bar was charged with NCS (1.0 equiv.), $Ru(bpy)_3(PF_6)_2$ (1 mol%) and the amine if solid (1.2 equiv.). The tube was capped with a Supelco aluminium crimp seal with septum (PTFE/butyl), evacuated and refilled with N_2 (x 3). CH_2Cl_2 (0.2 M) (dry and degassed by bubbling through with N_2 for 20 min) and the amine (1.2 equiv.) if liquid were added and the mixture was stirred for 1 h at room temperature. The mixture was cooled to 0 °C and a solution of the olefin (1.0 equiv.) in CH_2Cl_2 (0.2 M) and TFA (6 equiv.) were added. The LEDs were immediately switched on. The mixture was stirred under irradiation for 1 h at 0 °C. Aqueous 1 M KOH and EtOAc were added and the mixture was shaken vigorously. The aqueous layer was extracted with EOAc (x 3), the combined organic layers were dried ($MgSO_4$), filtered and evaporated. Purification by flash column or preparative thin-layer chromatography on silica gel gave the products.

General Procedure for the Olefin Aminochlorination Using Ammonium Salts – GP2

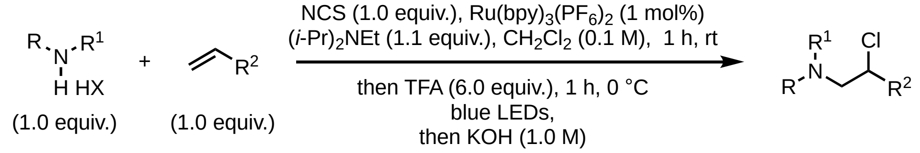

A dry tube equipped with a stirring bar was charged with NCS (1.0 equiv.), $Ru(bpy)_3(PF_6)_2$ (1 mol%) and the ammonium salt (1.0 equiv.). The tube was capped with a Supelco aluminium crimp seal with septum (PTFE/butyl), evacuated and refilled with N_2 (x 3). CH_2Cl_2 (0.2 M) (dry and degassed by bubbling through with N_2 for 20 min) and (*i*-Pr)$_2$NEt (1.1 equiv.) were added and the mixture was stirred for 60 min in the dark. The mixture was cooled to 0 °C and a solution of the olefin (1.0 equiv.) in CH_2Cl_2 (0.2 M) and TFA (6 equiv.) were added. The LEDs were immediately switched on. The mixture was stirred under irradiation for 1 h at 0 °C. Aqueous 1 M KOH and EtOAc were added and the mixture was shaken vigorously. The aqueous layer was extracted with EOAc (x 3), the combined organic layers were dried ($MgSO_4$), filtered

and evaporated. Purification by flash column or preparative thin-layer chromatography on silica gel gave the products.

1-(2-Chloro-4-phenylbutyl)piperidine (11)

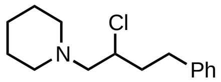

Following **GP1**, **1** (12 µL, 0.12 mmol) and **10** (10 µL, 0.1 mmol) gave **11** (23 mg, 91%) as an oil. R_f 0.60 [petrol:EtOAc (8:2)]; FT-IR ν_{max} (film)/cm^{-1} 2938, 2859, 2651, 2341, 1495, 1454, 1303, 1259, 1156, 1029; ^{1}H NMR (500 MHz, $CDCl_3$) δ 7.33–7.27 (2H, m), 7.24–7.18 (3H, m), 3.95 (1H, dddd, *J* = 11.0, 9.6, 4.0, 3.5 Hz), 2.93 (1H, ddd, *J* = 14.1, 9.5, 4.8 Hz), 2.75 (1H, ddd, *J* = 13.8, 9.3, 7.2 Hz), 2.64 (1H, dd, *J* = 13.1, 6.5 Hz), 2.52 (1H, dd, *J* = 13.1, 7.3 Hz), 2.38 (4H, br s), 2.33–2.17 (1H, m), 1.92 (1H, dtd, *J* = 14.3, 9.4, 4.9 Hz), 1.55 (4H, p, *J* = 5.6 Hz), 1.47–1.34 (2H, m); ^{13}C NMR (126 MHz, $CDCl_3$) δ 141.4, 128.7, 128.5, 126.1, 66.0, 59.5, 55.1, 38.0, 32.5, 26.0, 24.4; HRMS (HESI): found MH^+ 252.1512, $[C_{15}H_{23}NCl]^+$ requires 252.1514.

1-(2-Chloro-4-phenylbutyl)-2,6-*syn*-dimethylpiperidine (12)

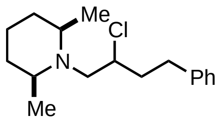

Following **GP1**, (2*S*,6*R*)-2,6-dimethylpiperidine (16 µL, 0.12 mmol) and **10** (10 µL, 0.1 mmol) gave **12** (15 mg, 55%) as an oil. R_f 0.40 [petrol:EtOAc (8:2)]; FT-IR ν_{max} (film)/cm^{-1} 2359, 2341, 1455, 1258, 1086, 1019; ^{1}H NMR (500 MHz, $CDCl_3$) δ 7.28 (2H, t, *J* = 7.3 Hz), 7.20 (3H, dd, *J* = 18.1, 7.6 Hz), 3.89–3.82 (1H, m), 2.96 (1H, dd, *J* = 14.0, 9.3, 4.4 Hz), 2.87 (1H, dd, *J* = 15.1, 5.8 Hz), 2.82–2.66 (2H, m), 2.57–2.49 (1H, m), 2.50–2.42 (1H, m), 2.38–2.28 (1H, m), 1.79 (1H, dtd, *J* = 14.4, 9.9, 4.3 Hz), 1.67–1.60 (1H, m), 1.50–1.42 (2H, m), 1.38–1.28 (1H, m), 1.28–1.17 (2H, m), 1.06 (3H, d, *J* = 6.2 Hz), 1.01 (3H, d, *J* = 6.3 Hz); ^{13}C NMR (126 MHz, $CDCl_3$) δ 141.4, 128.7, 128.5, 126.1, 62.8, 58.5, 57.9, 38.0, 33.1, 24.6, 22.2, 22.1; HRMS (ASAP): Found MH^+ 280.1822, $[C_{17}H_{27}NCl]^+$ requires 280.1827.

1-(2-Chloro-4-phenylbutyl)-2,2,6,6-tetramethylpiperidine (13)

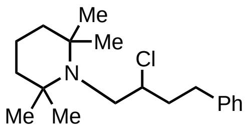

Following **GP1**, 2,2,6,6-tetramethylpiperidine (20 µL, 0.12 mmol) and **10** (10 µL, 0.1 mmol) gave **13** (6.5 mg, 21%) as an oil. R_f 0.35 [petrol:EtOAc (8:2)]; FT-IR ν_{max} (film)/cm^{-1} 2926, 2359, 2341, 1455, 1381, 1258, 1174, 1089, 1021; ^{1}H NMR (500 MHz, $CDCl_3$) δ 7.32–7.27 (2H, m), 7.24–7.15 (3H, m), 3.93–3.84 (1H, m), 2.99 (1H, ddd, *J* = 13.7, 9.2, 4.3 Hz), 2.89 (1H, dd, *J* = 15.6, 5.4), 2.79–2.61 (2H, m), 2.58–2.43 (1H, m), 1.82–1.65 (1H, m), 1.55 (1H, br s), 1.51 (1H, br s), 1.38 (4H, t, *J* = 6.0 Hz), 1.00 (6H, s), 0.92 (6H, s); ^{13}C NMR (126 MHz, $CDCl_3$) δ 141.6, 128.7, 128.5, 126.0, 65.7, 55.0, 52.5, 41.4, 37.2, 33.5, 25.8, 17.9; HRMS (ASAP): Found MH^+ 308.2133 $C_{19}H_{31}NCl$ requires 308.2140.

1-(2-Chloro-4-phenylbutyl)piperidin-3-ol (14)

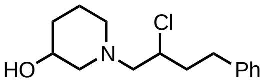

Following **GP2**, adding NaCl (6 mg, 0.1 mmol) to the reaction mixture, 3-hydroxypiperidine hydrochloride (16 mg, 0.12 mmol) and **10** (10 µL, 0.1 mmol) gave **14** (12 mg, 45%) as an oil. dr: 1:1. R_f 0.20 [petrol:EtOAc (8:2)]; FT-IR ν_{max} (film)/cm^{-1} 3334, 2359, 2341, 1634, 1260, 1017; ^{1}H NMR (500 MHz, $CDCl_3$, diastereomers) δ 7.30 (2H, t, *J* = 7.5 Hz), 7.23–7.19 (3H, m), 3.96–3.87 (1H, m), 3.85–3.72 (1H, m), 2.92 (1H, ddd, *J* = 14.0, 9.2, 4.9 Hz), 2.75 (1H, dt, *J* = 13.9, 8.1 Hz), 2.71–2.61 (1H, m), 2.61–2.54 (1H, m), 2.54–2.44 (3H, m), 2.29 (1H, q, *J* = 10.0, 9.5 Hz), 2.24–2.15 (1H, m), 1.99–1.85 (1H, m), 1.83–1.72 (1H, m), 1.61–1.53 (2H, m), 1.63–1.45 (1H, m); ^{13}C NMR (126 MHz, $CDCl_3$, diastereomers) δ 141.2 (x 2), 128.6 (x 2), 126.2 (x 2), 66.2, 66.1, 65.0, 64.8, 61.1, 60.7, 59.5, 59.2, 54.3, 54.1, 37.9 (x 2), 32.5 (x 2), 31.5 (x 2), 21.7, 21.6; HRMS (ASAP): Found MH^+ 268.1460 $C_{15}H_{23}NOCl$ requires 268.1463.

4-Azido-1-(2-chloro-4-phenylbutyl)piperidine (15)

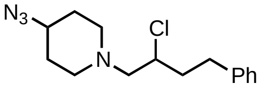

Following **GP2**, 4-azidopiperidine hydrochloride (15 mg, 0.12 mmol) and **10** (10 µL, 0.1 mmol) gave **15** (23 mg, 79%) as an oil. R_f 0.50 [petrol:EtOAc (8:2)]; FT-IR ν_{max}

(film)/cm^{-1} 2847, 2360, 1644, 1259, 1015; ^{1}H NMR (500 MHz, $CDCl_3$) δ 7.30 (2H, t, *J* = 7.6 Hz), 7.25–7.18 (3H, m), 3.90 (1H, dtd, *J* = 9.7, 7.0, 3.0 Hz), 3.42–3.34 (1H, m), 2.93 (1H, ddd, *J* = 13.9, 9.2, 4.8 Hz), 2.80–2.70 (3H, m), 2.67 (1H, dd, *J* = 13.3, 6.5 Hz), 2.56 (1H, dd, *J* = 13.2, 7.2 Hz), 2.32–2.13 (3H, m), 1.97–1.89 (1H, m), 1.90–1.82 (2H, m), 1.65 (2H, dtt, *J* = 15.8, 9.6, 4.9 Hz); ^{13}C NMR (126 MHz, $CDCl_3$) δ 141.1, 128.5, 128.5, 126.1, 64.8, 59.2, 57.5, 51.9, 51.4, 37.8, 32.4, 30.8, 30.7; HRMS (ASAP): Found MH^+ 293.1522 $C_{15}H_{22}N_4Cl$ requires 293.1528.

1-(1-(2-Chloro-4-phenylbutyl)-4-phenylpiperidin-4-yl)ethan-1-one (16)

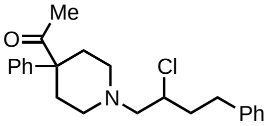

Following **GP2**, 4-acetyl-4-phenylpiperidine hydrochloride (29 mg, 0.12 mmol) and **10** (10 μL, 0.1 mmol) gave **16** (21 mg, 56%) as an oil. R_f 0.60 [petrol:EtOAc (8:2)]; FT-IR ν_{max} (film)/cm^{-1} 2925, 2341, 1702, 1599, 1494, 1446, 1353, 1259, 1202, 1028; ^{1}H NMR (500 MHz, $CDCl_3$) δ 7.39–7.34 (2H, m), 7.33–7.27 (5H, m), 7.25–7.18 (3H, m), 3.98–3.87 (1H, m), 2.93 (1H, ddd, *J* = 14.0, 9.3, 4.9 Hz), 2.82–2.73 (1H, m), 2.73–2.62 (3H, m), 2.54 (1H, dd, *J* = 13.1, 7.1 Hz), 2.48–2.39 (2H, m), 2.37–2.18 (3H, m), 2.14–2.01 (2H, m), 1.99–1.86 (1H, m), 1.91 (3H, s); ^{13}C NMR (126 MHz, $CDCl_3$) δ 209.6, 141.7, 141.3, 129.0, 128.7, 128.6, 127.3, 126.5, 126.2, 65.2, 59.3, 54.6, 51.4, 51.3, 38.0, 32.9, 32.8, 32.5, 25.8; HRMS (HESI): Found MH^+ 370.1926 $C_{23}H_{29}NOCl$ requires 370.1932.

***N*-(1-(2-Chloro-4-phenylbutyl)piperidin-4-yl)-*N*-cyclopropyl-3-(trifluoromethyl)benzene-sulfonamide (17)**

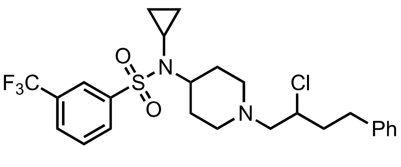

Following **GP1**, *N*-cyclopropyl-*N*-(piperidin-4-yl)-3-(trifluoromethyl)benzenesulfonamide (42 mg, 0.12 mmol) and **10** (10 μL, 0.1 mmol) gave **17** (31 mg, 60%) as an oil. R_f 0.40 [petrol:EtOAc (8:2)]; FT-IR ν_{max} (film)/cm^{-1} 2951, 2838, 2359, 2341, 1651, 1404, 1260, 1104, 1013; ^{1}H NMR (500 MHz, $CDCl_3$) δ 8.12 (1H, s), 8.05 (1H, d, *J* = 7.9 Hz), 7.84 (1H, d, *J* = 7.9 Hz), 7.67 (1H, t, *J* = 7.8 Hz), 7.29 (2H, t, *J* = 7.5 Hz), 7.20 (3H, d, *J* = 7.3 Hz), 4.15–3.64 (2H, m), 2.91 (1H, ddd, *J* = 14.1, 9.3, 4.9 Hz), 2.87 –

2.78 (2H, m), 2.73 (1H, dt, J = 14.0, 8.2 Hz), 2.64 (1H, dd, J = 13.2, 6.8 Hz), 2.52 (1H, dd, J = 13.2, 6.8 Hz), 2.25–2.14 (2H, m), 2.13–2.02 (1H, m), 2.00–1.85 (4H, m), 1.48 (2H, d, J = 11.5 Hz), 1.03–0.92 (2H, m), 0.78 (2H, q, J = 6.3 Hz); ^{13}C NMR (126 MHz, $CDCl_3$) δ 141.3, 141.2, 131.8 (q, J = 33.4 Hz), 130.6, 129.9, 129.2 (q, J = 3.3 Hz), 128.6, 128.5, 126.2, 124.6 (q, J = 3.6 Hz), 123.3 (q, J = 272.9 Hz), 64.9, 59.3, 59.0, 54.1, 53.8, 38.0, 32.5, 31.0, 30.9, 26.2, 7.8; ^{19}F NMR (471 MHz, $CDCl_3$) δ 62.81; HRMS (ASAP): Found MH^+ 515.1738 $C_{25}H_{31}N_2O_2ClSF_3$ requires 515.1741.

1-(2-Chloro-4-phenylbutyl)-4-(4-chlorophenyl)piperidin-4-ol (18)

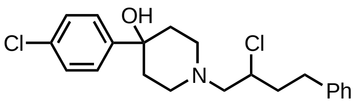

Following **GP1**, 4-(4-chlorophenyl)-4-hydroxypiperidine (25 mg, 0.12 mmol) and **10** (10 μL, 0.1 mmol) gave **18** (34 mg, 91%) as an oil. R_f 0.40 [petrol:EtOAc (8:2)]; FT-IR ν_{max} (film)/cm^{-1} 3322, 2960, 2918, 2849, 1637, 1493, 1454, 1398, 1258, 1090, 1012; ^{1}H NMR (500 MHz, $CDCl_3$) δ 7.43 (2H, d, J = 8.6 Hz), 7.36–7.28 (4H, m), 7.25–7.18 (3H, m), 4.02–3.94 (1H, m), 2.95 (1H, ddd, J = 14.0, 9.4, 4.9 Hz), 2.84–2.67 (4H, m), 2.63 (1H, dd, J = 13.2, 6.8 Hz), 2.58–2.43 (2H, m), 2.31–2.20 (1H, m), 2.15–2.04 (2H, m), 1.96 (1H, dtd, J = 14.2, 9.3, 4.9 Hz), 1.73–1.63 (2H, m); , 1.56 (1H, br s); ^{13}C NMR (126 MHz, $CDCl_3$) δ 146.9, 141.3, 132.9, 128.7, 128.6, 128.5, 126.2, 126.2, 71.0, 65.3, 59.4, 50.0, 49.8, 38.5, 38.4, 38.0, 32.5; HRMS (ASAP): Found MH^+ 378.1382 $C_{21}H_{26}NOCl_2$ requires 378.1386.

1-(2-Chloro-4-phenylbutyl)-4-(4-(trifluoromethoxy)phenoxy)piperidine (19)

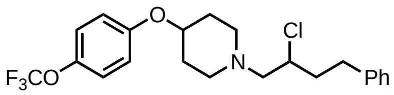

Following **GP1**, 4-[4-(trifluoromethoxy)phenoxy]piperidine (31 mg, 0.12 mmol) and **10** (10 μL, 0.1 mmol) gave **19** (36 mg, 84%) as an oil. R_f 0.40 [petrol:EtOAc (8:2)]; FT-IR ν_{max} (film)/cm^{-1} 2951, 2359, 2341, 1503, 1454, 1261, 1238, 1194, 1159, 1042; ^{1}H NMR (500 MHz, $CDCl_3$) δ 7.30 (2H, t, J = 7.4 Hz), 7.24–7.19 (3H, m), 7.12 (2H, d, J = 8.5 Hz), 6.87 (2H, d, J = 8.5 Hz), 4.31–4.21 (1H, m), 4.01–3.87 (1H, m), 2.94 (1H, ddd, J = 14.1, 9.3, 4.8 Hz), 2.77 (1H, dd, J = 14.4, 7.7 Hz), 2.74–2.66 (3H, m), 2.59 (1H, dd, J = 13.2, 7.2 Hz), 2.42–2.19 (3H, m), 2.05–1.85 (3H, m), 1.84–1.74 (2H, m); ^{13}C NMR (126 MHz, $CDCl_3$) δ ^{13}C NMR (126 MHz, $CDCl_3$) δ 156.1, 142.8 (q, J

= 2.1 Hz), 141.3, 128.7, 128.6, 126.2, 122.6, 120.7 (q, *J* = 256.1 Hz), 116.9, 73.1, 65.1, 59.4, 51.2, 50.8, 37.9, 32.5, 30.9, 30.8; ^{19}F NMR (471 MHz, $CDCl_3$) δ –58.4; HRMS (ASAP): Found MH^+ 428.1588 $C_{22}H_{26}NO_2ClF_3$ requires 428.1599.

2-((1-(2-Chloro-4-phenylbutyl)piperidin-4-yl)methyl)-5,6-dimethoxy-2,3-dihydro-*1H*-inden-1-one (20)

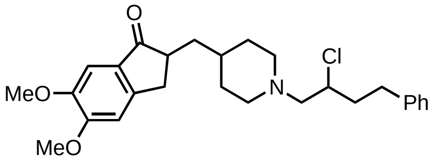

Following **GP2**, desbenzyl donepezil hydrochloride (39 mg, 0.12 mmol) and **10** (10 μL, 0.1 mmol) gave **20** (36 mg, 79%) as an oil. d.r. 1:1. R_f 0.20 [petrol:EtOAc (8:2)]; FT-IR ν_{max} (film)/cm^{-1} 2960, 2359, 2341, 1693, 1590, 1499, 1455, 1313, 1258, 1016; ^{1}H NMR (500 MHz, $CDCl_3$, diasteromers) δ 7.29 (2H, t, *J* = 7.5 Hz), 7.24–7.18 (3H, m), 7.17 (1H, s), 6.85 (1H, s), 3.96 (3H, s), 4.00–3.92 (1H, m), 3.90 (3H, s), 3.23 (1H, dd, *J* = 17.5, 8.0 Hz), 2.92 (1H, ddd, *J* = 14.0, 9.4, 4.8 Hz), 2.88–2.79 (2H, m), 2.78–2.72 (1H, m), 2.72–2.62 (3H, m), 2.59–2.52 (1H, m), 2.32–2.20 (1H, m), 2.12–1.84 (4H, m), 1.75–1.57 (2H, m), 1.52–1.43 (1H, m), 1.41–1.22 (3H, m); ^{13}C NMR (126 MHz, $CDCl_3$, diasteromers) δ 207.7 (x 2), 155.5 (x 2), 149.4 (x 2), 148.7 (x 2), 141.2 (x 2), 129.3 (x 2), 128.5 (x 2), 128.4 (x 2), 126.0 (x 2), 107.3 (x 2), 104.4 (x 2), 65.4 (x 2), 59.3 (x 2), 56.3, 56.2, 56.1, 56.0, 54.6 (x 2), 54.0 (x 2), 45.4 (x 2), 38.7 (x 2), 37.9 (x 2), 34.3 (x 2), 33.3 (x 2), 33.1, 32.9, 32.4 (x 2), 31.8, 31.7; HRMS (ASAP): Found MH^+ 456.2293 $C_{27}H_{35}NO_3NCl$ requires 456.2300.

4-(2-Chloro-4-phenylbutyl)morpholine (21)

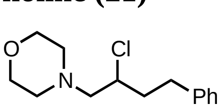

Following **GP1**, morpholine (10 μL, 0.12 mmol) and **10** (10 μL, 0.1 mmol) gave **21** (22 mg, 86%) as an oil. R_f 0.20 [petrol:EtOAc (8:2)]; FT-IR ν_{max} (film)/cm^{-1} 2957, 2853, 2810, 2359, 2341, 1495, 1454, 1360, 1259, 1207, 121116, 1069, 1008; ^{1}H NMR (500 MHz, $CDCl_3$) δ 7.34–7.27 (2H, m), 7.24–7.17 (3H, m), 4.01–3.87 (1H, m), 3.75–3.63 (4H, m), 2.93 (1H, ddd, *J* = 14.0, 9.2, 4.9 Hz), 2.81–2.71 (1H, m), 2.68 (1H, dd, *J* = 13.1, 6.8 Hz), 2.56 (1H, dd, *J* = 13.1, 7.0 Hz), 2.48–2.40 (4H, m), 2.30–2.19 (1H, m), 1.94 (1H, dtd, *J* = 14.2, 9.2, 4.9 Hz); ^{13}C NMR (126 MHz, $CDCl_3$) δ 141.2, 128.7,

128.6, 126.2, 67.0, 65.6, 58.7, 54.0, 37.9, 32.5; HRMS (ASAP): Found MH$^+$ 254.1302 $C_{14}H_{21}NOCl$ requires 254.1306.

4-(2-Chloro-4-phenylbutyl)-2,6-*syn*-dimethylmorpholine (22)

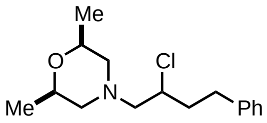

Following **GP1**, *cis*-2,6-dimethylmorpholine (15 µL, 0.12 mmol) and **10** (10 µL, 0.1 mmol) gave **22** (31 mg, 81%) as an oil. R_f 0.20 [petrol:EtOAc (8:2)]; FT-IR ν_{max} (film)/cm^{-1} 2931, 2858, 2815, 2360, 2342, 1495, 1454, 1374, 1322, 1225, 1178, 1143, 1085, 1049, 1030; ^{1}H NMR (500 MHz, $CDCl_3$) δ 7.34–7.25 (2H, m), 7.24–7.17 (3H, m), 3.93 (1H, dtd, *J* = 9.8, 6.8, 3.1 Hz), 3.65 (2H, dqd, *J* = 16.4, 8.7, 4.4 Hz), 2.93 (1H, ddd, *J* = 13.9, 9.2, 4.9 Hz), 2.75 (1H, dt, *J* = 13.8, 8.2 Hz), 2.69–2.48 (4H, m), 2.30–2.19 (1H, m), 1.99–1.87 (1H, m), 1.79 (2H, dt, *J* = 20.5, 10.5 Hz), 1.13 (3H, t, *J* = 6.7 Hz), 1.12 (3H, t, *J* = 6.4 Hz); ^{13}C NMR (126 MHz, $CDCl_3$) δ 141.1, 128.5, 128.5, 126.1, 71.6, 71.5, 65.0, 59.8, 59.6, 58.6, 37.8, 32.3, 19.1 (x 2); HRMS (ASAP): Found MH$^+$ 282.1617 $C_{16}H_{25}NOCl$ requires 282.1619.

Benzyl 4-(2-Chloro-4-phenylbutyl)piperazine-1-carboxylate (23)

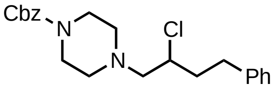

Following **GP1**, 1-Cbz-piperazine (23 µL, 0.12 mmol) and **10** (10 µL, 0.1 mmol) gave **23** (29 mg, 75%) as an oil. R_f 0.60 [petrol:EtOAc (8:2)]; FT-IR ν_{max} (film)/cm^{-1} 2960, 2359, 2341, 1699, 1496, 1454, 1428, 1361, 1257, 1237, 1079, 1015; ^{1}H NMR (500 MHz, $CDCl_3$) δ 7.39–7.27 (7H, m), 7.23–7.18 (3H, m), 5.13 (2H, s), 3.92 (1H, dtd, *J* = 9.8, 6.7, 3.1 Hz), 3.49 (4H, t, *J* = 5.0 Hz), 2.92 (1H, ddd, *J* = 13.9, 9.1, 4.9 Hz), 2.81–2.72 (1H, m), 2.70 (1H, dd, *J* = 13.4, 7.0 Hz), 2.59 (1H, dd, *J* = 13.2, 6.8 Hz), 2.42 (4H, br s), 2.30–2.18 (1H, m), 2.01–1.86 (1H, m); ^{13}C NMR (126 MHz, $CDCl_3$) δ 155.1, 141.0, 136.7, 128.55 (x 2), 128.5, 128.0, 127.9, 126.1, 67.2, 64.9, 58.6, 53.1, 43.7, 37.7, 32.3; HRMS (ASAP): Found MH$^+$ 387.1826 $C_{22}H_{28}ClN_2O_2$ requires 387.1834.

1-(2-Chloro-4-phenylbutyl)pyrrolidine (24)

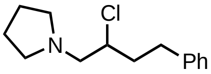

Following **GP1** but adding NaCl (6 mg, 0.1 mmol) to the reaction mixture, pyrrolidine (10 μL, 0.12 mmol) and **10** (10 μL, 0.1 mmol) gave **24** (22 mg, 91%) as an oil. R_f 0.40 [petrol:EtOAc (8:2)]; ^{1}H NMR (500 MHz, $CDCl_3$) δ 7.34–7.24 (2H, m), 7.23–7.17 (3H, m), 4.01–3.85 (1H, m), 2.93 (1H, ddd, *J* = 14.2, 9.4, 4.9 Hz), 2.86–2.65 (3H, m), 2.52 (4H, br s), 2.29–2.17 (1H, m), 1.95 (1H, dtd, *J* = 10.7, 9.2, 8.6, 4.8 Hz), 1.76 (4H, br s); ^{13}C NMR (126 MHz, $CDCl_3$) δ 141.1, 128.5, 128.4, 126.0, 63.3, 60.5, 54.4, 37.9, 32.4, 23.5. Data in accordance with the literature.[10]

1-(2-Chloro-4-phenylbutyl)azepane (25)

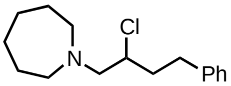

Following **GP1**, hexamethyleneimine (13.5 μL, 0.12 mmol) and **10** (10 μL, 0.1 mmol) gave **25** (19 mg, 74%) as an oil. R_f 0.60 [petrol:EtOAc (8:2)]; FT-IR ν_{max} (film)/cm^{-1} 2359, 2341, 1652, 1459, 1454, 1258, 1017; ^{1}H NMR (500 MHz, $CDCl_3$) δ 7.34–7.27 (2H, m), 7.24–7.16 (3H, m), 3.90–3.80 (1H, m), 2.93 (1H, ddd, *J* = 14.1, 9.5, 4.8 Hz), 2.85 (1H, dd, *J* = 13.4, 5.8 Hz), 2.75 (1H, dd, *J* = 14.9, 7.3 Hz), 2.72–2.67 (1H, m), 2.66 (4H, br s), 2.37–2.25 (1H, m), 1.90 (1H, dtd, *J* = 14.2, 9.3, 4.6 Hz), 1.65–1.50 (8H, m); ^{13}C NMR (126 MHz, $CDCl_3$) δ 141.5, 128.7, 128.5, 126.1, 64.5, 60.7, 55.9, 37.7, 32.6, 28.5, 27.3; HRMS (ASAP): Found MH^+ 266.1668 $C_{16}H_{25}NCl$ requires 266.1670.

1-(2-Chloro-4-phenylbutyl)azetidine (26)

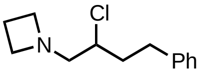

Following **GP2** but using 2.0 equiv. of the olefin, azetidine hydrochloride (11 mg, 0.12 mmol) and **10** (20 μL, 0.2 mmol) gave **26** (17 mg, 76%) as an oil. R_f 0.50 [petrol:EtOAc (8:2)]; FT-IR ν_{max} (film)/cm^{-1} 2959, 2359, 2341, 1715, 1455, 1259, 1179, 1028; ^{1}H NMR (500 MHz, $CDCl_3$) δ 7.32–7.25 (2H, m), 7.23–7.14 (3H, m), 3.84–3.73 (1H, m), 3.26 (4H, t, *J* = 7.1 Hz), 2.89 (1H, ddd, *J* = 14.0, 9.3, 4.9 Hz), 2.79–2.61 (3H, m), 2.19–2.01 (3H, m), 1.92 (1H, dtd, *J* = 14.2, 9.3, 4.9 Hz); ^{13}C NMR (126 MHz, $CDCl_3$) δ 141.2, 128.7, 128.6, 126.2, 66.6, 60.5, 56.4, 38.0, 32.6, 18.2; HRMS (ASAP): Found MH^+ 224.1199 $C_{13}H_{19}NCl$ requires 224.1201.

6-(2-Chloro-4-phenylbutyl)-2-oxa-6-azaspiro[3.3]heptane (27)

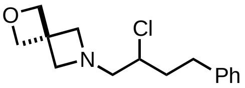

Following **GP2** but using 3.0 equiv. of the olefin, 2-oxa-6-azaspiro[3.3]heptane oxalate (23 mg, 0.12 mmol) and **10** (30 μL, 0.3 mmol) gave **27** (12 mg, 45%) as an oil. R_f 0.20 [petrol:EtOAc (8:2)]; FT-IR ν_{max} (film)/cm^{-1} 2961, 2360, 2341, 1259, 1028; ^{1}H NMR (500 MHz, $CDCl_3$) δ 7.33–7.26 (2H, m), 7.24–7.12 (3H, m), 4.72 (4H, s), 3.78–3.70 (1H, m), 3.41 (4H, s), 2.87 (1H, ddd, *J* = 14.0, 9.0, 5.0 Hz), 2.72 (1H, dd, *J* = 15.0, 6.5 Hz), 2.66 (1H, br s), 2.65 (1H, br s), 2.14–1.99 (1H, m), 1.92 (1H, dtd, *J* = 14.3, 9.2, 5.0 Hz); ^{13}C NMR (126 MHz, $CDCl_3$) δ 141.0, 128.6, 128.6, 126.2, 81.4, 66.1, 65.0, 60.5, 39.7, 37.9, 32.5; HRMS (HESI): Found MH$^+$ 266.1295 $C_{15}H_{21}NOCl$ requires 266.1293.

2-Chloro-*N,N*-dimethyl-4-phenylbutan-1-amine (28)

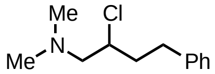

Following **GP2**, Me_2NH•HCl (10 mg, 0.12 mmol) and **10** (10 μL, 0.1 mmol) gave **29** (19 mg, 91%) as an oil. R_f 0.60 [petrol:EtOAc (8:2)]; FT-IR ν_{max} (film)/cm^{-1} 3362, 2960, 2919, 2849, 2360, 1634, 1455, 1258, 1018; ^{1}H NMR (500 MHz, $CDCl_3$) δ 7.34–7.25 (2H, m), 7.25–7.17 (3H, m), 3.97–3.85 (1H, m), 2.93 (1H, ddd, *J* = 14.1, 9.4, 4.9 Hz), 2.76 (1H, ddd, *J* = 13.7, 9.2, 7.2 Hz), 2.64 (1H, dd, *J* = 12.9, 7.5 Hz), 2.51 (1H, dd, *J* = 12.9, 6.2 Hz), 2.26 (6H, s), 2.23–2.15 (1H, m), 1.93 (1H, dtd, *J* = 14.3, 9.4, 4.9 Hz); ^{13}C NMR (101 MHz, $CDCl_3$) δ 141.2, 128.7, 128.6, 126.2, 66.5, 59.4, 45.8, 38.0, 32.5; HRMS (ASAP): Found MH$^+$ 212.1203 $C_{12}H_{19}NCl$ requires 212.1201.

2-Chloro-*N,N*-diethyl-4-phenylbutan-1-amine (29)

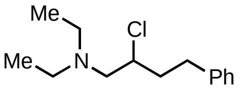

Following **GP1**, **8** (46 μL, 0.12 mmol) and **10** (10 μL, 0.1 mmol) gave **29** (15 mg, 62%) as an oil. R_f 0.20 [petrol:EtOAc (8:2)]; FT-IR ν_{max} (film)/cm^{-1} 2359, 2341, 1635, 1495, 1454, 1258, 1015; ^{1}H NMR (500 MHz, $CDCl_3$) δ 7.35–7.27 (2H, m), 7.24–7.16 (3H, m), 4.05–3.71 (1H, m), 2.94 (1H, ddd, *J* = 14.1, 9.6, 4.7 Hz), 2.80–2.68 (2H, m), 2.62 (1H, dd, *J* = 13.6, 7.7 Hz), 2.58–2.44 (4H, m), 2.36–2.18 (1H, m), 1.87 (1H, dtd, *J* = 14.2, 9.5, 4.7 Hz), 0.98 (6H, t, *J* = 7.1 Hz); ^{13}C NMR (126 MHz, $CDCl_3$) δ 141.5, 128.7,

128.5, 126.1, 61.1, 60.7, 48.1, 37.8, 32.7, 12.1; HRMS (HESI): Found MH^+ 240.1509 $C_{14}H_{23}NCl$ requires 240.1514.

2-((2-Chloro-4-phenylbutyl)(methyl)amino)ethan-1-ol (30)

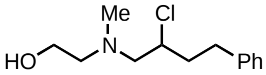

Following **GP1**, 2-(methylamino)ethanol (10 μL, 0.12 mmol) and **10** (10 μL, 0.1 mmol) gave **30** (15 mg, 64%) as an oil. R_f 0.20 [petrol:EtOAc (8:2)]; FT-IR ν_{max} (film)/cm^{-1} 3378, 2922, 2852, 2358, 2342, 2170, 2343, 2170, 1973, 1454, 1199, 1031; ^1H NMR (500 MHz, $CDCl_3$) δ 7.32–7.28 (2H, m), 7.24–7.17 (3H, m), 3.98–3.90 (1H, m), 3.62–3.53 (2H, m), 2.93 (1H, ddd, *J* = 14.0, 9.3, 5.0 Hz), 2.80–2.69 (2H, m), 2.68–2.59 (2H, m), 2.58–2.52 (1H, m), 2.28 (3H, s), 2.18–2.07 (1H, m), 1.94 (1H, dtd, *J* = 14.2, 9.3, 5.0 Hz); ^{13}C NMR (126 MHz, $CDCl_3$) δ 141.0, 128.7, 128.6, 126.3, 64.5, 60.1, 59.5, 58.6, 42.3, 37.9, 32.5; HRMS (ASAP): Found MH^+ 242.1305 $C_{13}H_{21}NOCl$ requires 242.1306.

2-Chloro-*N*-methyl-4-phenylbutan-1-amine (31)

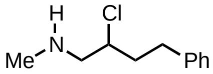

Following **GP1**, Me_2NH (11 μL, 0.12 mmol, 33 wt.% in ethanol) and **10** (10 μL, 0.1 mmol) gave **31** (4 mg, 19%) as an oil. R_f 0.45 [petrol:EtOAc (8:2)]; FT-IR ν_{max} (film)/cm^{-1} 2961, 2909, 2852, 2359, 2341, 1456, 1259, 1029; ^1H NMR (500 MHz, $CDCl_3$) δ 7.30 (2H, t, *J* = 7.5 Hz), 7.25–7.17 (3H, m), 4.20 (1H, tt, *J* = 8.9, 4.0 Hz), 3.93 (1H, dd, *J* = 14.0, 4.2 Hz), 3.42 (1H, dd, *J* = 14.0, 8.9 Hz), 3.24 (3H, s), 2.94 (1H, ddd, *J* = 14.1, 9.3, 5.1 Hz), 2.80–2.70 (1H, m), 2.13–2.03 (1H, m), 2.03–1.91 (1H, m), 1.56 (1H, br s); ^{13}C NMR (126 MHz, $CDCl_3$) δ 140.4, 128.6, 128.5, 126.3, 58.7, 56.6, 37.4, 37.2, 32.3; HRMS (ASAP): Found MH^+ 197.0972 $C_{11}H_{17}NCl$ requires 197.0971.

***N*-(2-Chloro-4-phenylbutyl)cyclohexanamine (32)**

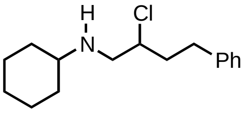

Following **GP1** but adding KPF_6 (18 mg, 0.1 mmol) to the reaction mixture, cyclohexylamine (14 μL, 0.12 mmol) and **10** (10 μL, 0.1 mmol) gave **32** (17 mg, 65%)

as an oil. R_f 0.40 [petrol:EtOAc (8:2)]; FT-IR ν_{max} (film)/cm^{-1} 2961, 2926, 2854, 1410, 1258, 1018; ^{1}H NMR (500 MHz, $CDCl_3$) δ 7.28 (2H, t, *J* = 7.9 Hz), 7.23–7.16 (3H, m), 4.04–3.97 (1H, m), 2.96–2.81 (3H, m), 2.75 (1H, dt, *J* = 13.2, 8.2 Hz), 2.46–2.37 (1H, m), 2.13–1.98 (2H, m), 1.85 (2H, d, *J* = 11.8 Hz), 1.72 (2H, d, *J* = 12.7 Hz), 1.61 (1H, d, *J* = 12.6 Hz), 1.27–1.01 (6H, m); ^{13}C NMR (126 MHz, $CDCl_3$) δ 140.9, 128.5, 128.5, 126.1, 63.2, 56.3, 53.4, 38.0, 33.6, 33.4, 32.6, 26.1, 25.0, 24.9; HRMS (ASAP): Found MH$^+$ 266.1666 $C_{16}H_{25}NCl$ requires 266.1670.

_N_-(_tert_-Butyl)-2-chloro-4-phenylbutan-1-amine (33)

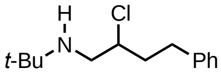

Following **GP1**, *t*-BuNH_2 (13 µL, 0.12 mmol) and **10** (10 µL, 0.1 mmol) gave **34** (20 mg, 85%) as an oil. R_f 0.50 [petrol:EtOAc (8:2)]; FT-IR ν_{max} (film)/cm^{-1} 2839, 2360, 2341, 1645, 1404, 1260, 1014; ^{1}H NMR (500 MHz, $CDCl_3$) δ 7.27 (2H, t, *J* = 7.1 Hz), 7.22–7.14 (3H, m), 4.04–3.87 (1H, m), 2.94–2.85 (1H, m), 2.83–2.76 (2H, m), 2.79–2.70 (1H, m), 2.18–1.97 (2H, m), 1.31 (1H, br s), 1.09 (9H, s); ^{13}C NMR (126 MHz, $CDCl_3$) δ 141.1, 128.7, 128.6, 126.2, 64.0, 50.5, 49.7, 38.2, 32.7, 29.1; HRMS (ASAP): Found MH$^+$ 240.1512 $C_{14}H_{23}NCl$ requires 240.1514.

_N_-(2-chloro-4-phenylbutyl)adamantan-1-amine (34)

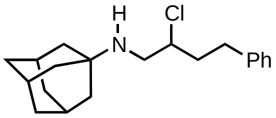

Following **GP1**, 1-adamantylamine (12 mg, 0.12 mmol) and **10** (10 µL, 0.1 mmol) gave **34** (29 mg, 91%) as an oil. R_f 0.40 [petrol:EtOAc (8:2)]; FT-IR ν_{max} (film)/cm^{-1} 2961, 2909, 2852, 2359, 2341, 1456, 1259, 1029; ^{1}H NMR (500 MHz, $CDCl_3$) δ 7.32–7.26 (2H, m), 7.23–7.16 (3H, m), 4.00–3.90 (1H, m), 3.00–2.80 (3H, m), 2.80–2.70 (1H, m), 2.17–1.97 (5H, m), 1.73–1.49 (12H, m); ^{13}C NMR (126 MHz, $CDCl_3$) δ 141.1, 128.7, 128.6, 126.2, 64.3, 50.5, 47.7, 42.9, 38.2, 36.8, 32.7, 29.7; HRMS (ASAP): Found MH$^+$ 318.1969 $C_{20}H_{29}NCl$ requires 318.1983.

(*1S,2R*)-2-((2-Chloro-4-phenylbutyl)amino)-1-phenylpropan-1-ol (35)

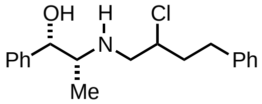

Following **GP1** but using 10.0 equiv. of TFA, (*1S,2R*)-(+)-norephedrine (18 mg, 0.12 mmol) and **10** (10 μL, 0.1 mmol) gave **35** (6 mg, 20%) as an oil. d.r. 1:1. R_f 0.70 [petrol:EtOAc (8:2)]; FT-IR ν_{max} (film)/cm^{-1} 2960, 2924, 2360, 2341, 1455, 1257, 1016; ^{1}H NMR (500 MHz, $CDCl_3$, diastereomers) δ 7.38–7.27 (6.5H, m), 7.24–7.18 (3.5H, m), 4.72 (0.5H, d, *J* = 3.8 Hz), 4.70 (0.5H, d, *J* = 3.9 Hz), 4.06–3.94 (1H, m), 3.06 (0.5H, dd, *J* = 13.0, 3.9 Hz), 2.97–2.89 (2.5H, m), 2.82–2.71 (1H, m), 2.15–2.03 (2H, m), 1.31 (1H, br s), 1.29–1.22 (1H, m), 0.85 (1.5H, d, *J* = 3.3 Hz), 0.83 (1.5H, d, *J* = 3.4 Hz); ^{13}C NMR (126 MHz, $CDCl_3$, diastereomers) δ 141.2, 141.1, 140.9, 140.8, 128.7 (x 2), 128.6 (x 2), 128.3, 128.2, 127.2 (x 2), 126.4 (x 2), 126.2 (x 2), 73.5, 73.4, 63.1, 62.5, 58.4, 58.0, 54.0, 53.4, 38.0, 37.7, 32.8, 32.6, 15.0, 14.8; HRMS (ASAP): Found MH$^+$ 318.1612 $C_{19}H_{25}NOCl$ requires 318.1619.

2-Chloro-*N*-(((*1S,2R,5S*)-6,6-dimethylbicyclo[3.1.1]heptan-2-yl)methyl)-4-phenylbutan-1-amine (36)

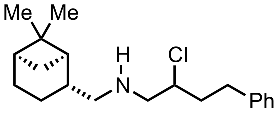

Following **GP1**, (–)-*cis*-myrtanylamine (20 μL, 0.12 mmol) and **10** (10 μL, 0.1 mmol) gave **36** (10 mg, 30%) as an oil. d.r.1:1. R_f 0.50 [petrol:EtOAc (8:2)]; FT-IR ν_{max} (film)/cm^{-1} 2959, 2359, 2341, 1455, 1258, 1020; ^{1}H NMR (500 MHz, $CDCl_3$) δ 7.34–7.24 (2H, m), 7.24–7.14 (3H, m), 4.09–3.96 (1H, m), 2.90 (1H, ddd, *J* = 14.2, 8.6, 5.7 Hz), 2.86–2.78 (1H, m), 2.80–2.69 (1H, m), 2.67–2.47 (2H, m), 2.39–2.29 (1H, m), 2.22–2.13 (1H, m), 2.09–1.99 (2H, m), 1.95–1.86 (4H, m), 1.77–1.63 (1H, m), 1.56 (1H, br s), 1.50–1.38 (1H, m), 1.34–1.27 (1H, m), 1.18 (3H, s), 0.97 (3H, s), 0.93–0.87 (1H, m); ^{13}C NMR (126 MHz, $CDCl_3$, diastereomers) δ 142.1, 141.1, 128.7, 128.6, 128.6, 128.4, 126.2, 125.9, 62.9, 62.8, 56.5, 56.4, 56.0, 55.9, 44.6, 44.5, 41.8, 41.7, 41.7, 41.6, 38.8 (x 2), 38.0 (x 2), 33.6 (x 2), 32.7 (x 2), 28.3, 28.2, 26.4, 26.3, 23.5(x2), 20.8, 20.7; HRMS (ASAP): Found MH$^+$ 320.2136 $C_{20}H_{31}NCl$ requires 320.2140.

1-(2-Chloro-4-methylpentyl)piperidine (37)

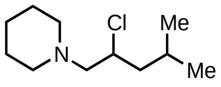

Following **GP1**, **1** (48 μL, 0.48 mmol) and 4-methylpent-1-ene (34 mg, 0.4 mmol) gave **37** (68 mg, 83%) as an oil. R_f 0.30 [petrol:EtOAc (95:5)]; ^{1}H NMR (500 MHz, $CDCl_3$) δ 4.02 (1H, dtd, *J* = 10.3, 6.7, 3.7 Hz), 2.62 (1H, dd, *J* = 13.1, 6.8 Hz), 2.47 (1H, dd, *J* = 13.2, 6.5 Hz), 2.45–2.33 (4H, m), 1.99–1.81 (1H, m), 1.67–1.51 (6H, m), 1.41 (2H, p, *J* = 5.7 Hz), 0.94 (3H, d, *J* = 6.7 Hz), 0.89 (3H, d, *J* = 6.6 Hz); ^{13}C NMR (125 MHz, $CDCl_3$) δ 66.8, 58.6, 55.1, 45.7, 26.0, 25.3, 24.4, 23.5, 21.2. HRMS (ESI$^+$): found MH$^+$ 204.1516, $[C_{11}H_{23}ClN]^+$ requires 204.1520.

1-(2-Chloro-2-cyclohexylethyl)piperidine (38)

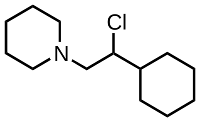

Following **GP1, 1** (48 μL, 0.48 mmol) and vinylcyclohexane (56 μL, 0.4 mmol) gave **38** (79 mg, 86%) as an oil. R_f 0.30 [petrol:EtOAc (95:5)]; FT-IR ν_{max} (film)/cm^{-1}: 2960, 2926, 1259, 800; ^{1}H NMR (400 MHz, $CDCl_3$) δ 3.96 (1H, td, *J* = 6.7, 3.0 Hz), 2.60 (2H, d, *J* = 6.7 Hz), 2.41 (4H, t, *J* = 5.4 Hz), 1.83–1.62 (6H, m), 1.57 (4H, p, *J* = 5.7 Hz), 1.44–1.38 (2H, m), 1.37–1.26 (2H, m); 1.24–1.07 (3H, m). ^{13}C NMR (101 MHz, $CDCl_3$) δ 65.5, 63.7, 54.9, 42.2, 30.5, 27.0, 26.4, 26.3, 26.1, 25.8, 24.2; HRMS (ESI$^+$): found MH$^+$ 230.1666, $[C_{13}H_{25}ClN]^+$ requires 230.1676.

Methyl 3-chloro-4-(piperidin-1-yl)pentanoate (39)

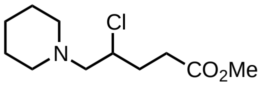

Following **GP1**, **1** (12 μL, 0.12 mmol) and methyl 4-pentenoate (12 μL, 0.1 mmol) gave **39** (20 mg, 93%) as an oil. R_f 0.30 [petrol:EtOAc (90:10)]; FT-IR ν_{max} (film)/cm^{-1} 2361, 2341, 1733; ^{1}H NMR (500 MHz, $CDCl_3$) δ 4.03 (1H, dddd, *J* = 9.5, 7.7, 6.2, 3.2 Hz), 3.68 (3H, s), 2.66–2.27 (9H, m), 1.92–1.80 (1H, m), 1.63–1.50 (4H, m), 1.41 (2H, p, *J* = 6.0 Hz); ^{13}C NMR (126 MHz, $CDCl_3$) δ 173.7, 66.0, 59.2, 55.1, 51.8, 31.5, 30.9, 26.1, 24.4; HRMS (ESI+): found MH$^+$ 234.1246, $[C_{11}H_{21}ClNO_2]^+$ requires 234.1262.

4-Chloro-5-(piperidin-1-yl)pentanenitrile (40)

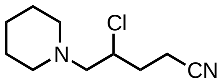

Following **GP1**, **1** (12 µL, 0.12 mmol) and 4-pentenenitrile (10 µL, 0.1 mmol) gave **40** (18 mg, 90%) as an oil. R_f 0.30 [petrol:EtOAc (95:5)]; ^{1}H NMR (500 MHz, $CDCl_3$) δ 4.02–4.03 (1H, m), 2.69–2.32 (9H, m), 1.90 (1H, m), 1.60–1.49 (4H, m), 1.41 (2H, p, *J* = 6.0 Hz); ^{13}C NMR (126 MHz, $CDCl_3$) δ 119.2, 65.3, 57.6, 55.2, 32.1, 26.1, 24.2, 14.7; HRMS (ESI$^+$): found MH$^+$ 201.1150, $[C_{10}H_{18}ClN_2]^+$ requires 201.1159.

4-Chloro-*N*-methoxy-*N*-methyl-5-(piperidin-1-yl)pentanamide (41)

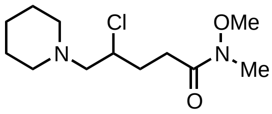

Following **GP1**, **1** (24 µL, 0.24 mmol) and **S1** (18 µL, 0.2 mmol) gave **41** (14 mg, 64%) as an oil. R_f 0.30 [petrol:EtOAc:NH_3 aq. (60:40:0.5)]; FT-IR ν_{max} (film)/cm^{-1}: 2957, 1659, 1172, 802; ^{1}H NMR (400 MHz, $CDCl_3$) δ 4.10 (1H, dtd, *J* = 9.9, 6.8, 2.8 Hz), 3.69 (3H, s), 3.17 (3H, s), 2.75–2.60 (3H, m), 2.59–2.28 (6H, m), 1.87–1.75 (1H, m), 1.56 (4H, p, *J* = 5.6 Hz), 1.47–1.34 (2H, m); ^{13}C NMR (101 MHz, $CDCl_3$) δ 173.8, 66.1, 61.4, 59.6, 55.0, 32.3, 31.2, 28.7, 25.9, 24.3; HRMS (ESI$^+$): found MH$^+$ 263.1509, $[C_{12}H_{24}ClN_2O_2]^+$ requires 263.1529.

4-Chloro-5-(piperidin-1-yl)pentyl benzoate (42)

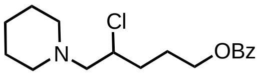

Following **GP1**, **1** (48 µL, 0.48 mmol) and **S2** (76 mg, 0.4 mmol) gave **42** as an oil (124 mg, quantitative). R_f 0.30 [petrol:EtOAc (85:15)]; ^{1}H NMR (500 MHz, $CDCl_3$) δ 8.09–8.01 (2H, m), 7.58–7.52 (1H, m), 7.48–7.40 (2H, m), 4.36 (2H, t, *J* = 6.2 Hz), 4.12–3.97 (1H, m), 2.65 (1H, dd, *J* = 13.1, 6.4 Hz), 2.52 (1H, dd, *J* = 13.1, 7.5 Hz), 2.48–2.33 (4H, m), 2.21–2.03 (2H, m), 1.98–1.85 (1H, m), 1.82–1.71 (1H, m), 1.55 (2H, p, *J* = 5.8 Hz), 1.40 (2H, p, *J* = 6.0 Hz); ^{13}C NMR (126 MHz, $CDCl_3$) δ 166.7, 133.0, 130.4, 129.7, 128.5, 66.0, 64.5, 59.6, 55.1, 32.9, 26.0, 25.7, 24.3; HRMS (ESI): found M$^+$ 309.1490, $[C_{17}H_{24}ClNO_2]^+$ requires 309.1496.

N-(2-Chloro-3-(piperidin-1-yl)propyl)-4-methylbenzenesulfonamide (43)

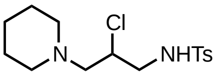

Following **GP1**, **1** (48 μL, 0.48 mmol) and **S3** (62 mg, 0.4 mmol) gave **43** (120 mg, 91%) as an oil. R$_f$ 0.30 [petrol:EtOAc (8:2)]; ^{1}H NMR (400 MHz, $CDCl_3$) δ 7.75 (2H, d, J = 8.2 Hz), 7.32 (2H, d, J = 7.9 Hz), 6.93 (1H, br s), 3.96 (1H, tt, J = 9.1, 4.5 Hz), 3.42 (1H, dd, J = 12.8, 4.9 Hz), 3.21 (1H, ddd, J = 12.9, 8.2, 1.3 Hz), 2.67 (1H, dd, J = 12.9, 4.3 Hz), 2.54 (1H, d, J = 11.3 Hz), 2.51–2.42 (2H, m), 2.44 (3H, s), 2.35–2.23 (2H, m), 1.58 (4H, p, J = 5.7 Hz), 1.48–1.38 (2H, m); ^{13}C NMR (126 MHz, $CDCl_3$) δ 143.6, 137.3, 129.9, 127.1, 65.7, 55.1, 53.8, 50.2, 26.1, 24.0, 21.7; HRMS (ESI+): found MH$^+$ 331.1233, $[C_{15}H_{24}ClN_2O_2S]^+$ requires 331.1248.

***syn*-1-(2-Chlorocyclohexyl)piperidine (44) and *anti*-1-(2-Chlorocyclohexyl)piperidine (44')**

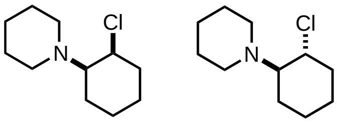

Following **GP1**, **1** (48 μL, 0.48 mmol) and cyclohexene (41 μL, 0.4 mmol, 1.0 equiv.) gave **44** and **44'** (70% crude NMR yield). **44**:**44'** = 1.2:1. **44'** decomposes on silica. Following **GP1** but using 1.5 equiv. of piperidine and 1.6 equiv. of NCS gave **44** as an oil (42 mg, 52%). Data for **44**: R$_f$ 0.40 [petrol:EtOAc:aq. NH_3 37% (40:60:0.5)]; ^{1}H NMR (500 MHz, $CDCl_3$) δ 4.65–4.56 (1H, m), 2.71–2.57 (4H, m), 2.42 (1H, d J = 11.6), 2.04–2.00 (1H, m), 1.84–1.77 (2H, m), 1.77–1.53 (8H, m), 1.48–1.41 (3H, m); ^{13}C NMR (126 MHz, $CDCl_3$) δ 66.9, 60.5, 50.8, 34.4, 26.0, 25.5, 24.5, 24.0, 19.8; HRMS (ESI$^+$): Found MH$^+$ 202.1357, $[C_{11}H_{21}ClN]^+$ requires 202.1363

***Tert*-butyl 3-chloro-3-(piperidin-1-ylmethyl)azetidine-1-carboxylate (45)**

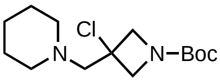

Following **GP1**, **1** (14 μL, 0.14 mmol) and *tert*-butyl 3-methyleneazetidine-1-carboxylate (19 μL, 0.12 mmol) gave **45** (85%) as an oil. R$_f$ 0.60 [petrol:EtOAc (80:20)]; FT-IR ν_{max} (film)/cm^{-1} 2361, 2339, 1554, 1260, 1080; ^{1}H NMR (500 MHz, $CDCl_3$) δ 4.13 (2H, d, J = 9.3 Hz), 4.04 (2H, d, J = 9.3 Hz), 2.69 (2H, s), 2.50 (4H, t, J = 5.2 Hz), 1.59–1.50 (4H, m), 1.42 (9H, s), 1.41–1.35 (2H, m); ^{13}C NMR (126 MHz,

$CDCl_3$) δ 156.2, 80.0, 65.8, 62.8, 61.7, 55.9, 28.4, 26.1, 24.0; HRMS (ESI): Found MH^+ 289.1681, $C_{14}H_{26}N_2O_2Cl$ requires 289.1683.

Benzyl (*2S*)-2-(((benzyloxy)carbonyl)amino)-4-chloro-5-(piperidin-1-yl)pentanoate (46)

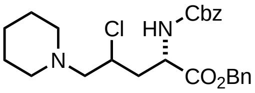

Following **GP1**, **1** (48 μL, 0.48 mmol) and **S5** (184 mg, 0.4 mmol, 1.00 equiv.) gave **46** as an oil (130 mg, 96%). d.r. 1:1.

Data for the first eluting isomer: R_f 0.4 [petrol:EtOAc:NH_3 aq. 37% (70:30:05)]; ^{1}H NMR (400 MHz, $CDCl_3$) 7.47–7.27 (10H, m), δ 5.96 (1H, d, *J* = 8.1 Hz), 5.30–4.98 (4H, m), 4.53 (1H, m), 3.95 (1H, m), 2.73–2.43 (2H, m), 2.43–2.15 (6H, m), 1.70–1.45 (4H, m), 1.45–1.28 (2H, m; ^{13}C NMR (101 MHz, $CDCl_3$) δ 171.9, 156.3, 136.6, 135.5, 128.7, 128.5, 128.5, 128.3, 128.2, 128.2, 67.3, 67.0, 65.9, 54.9, 54.1, 51.9, 40.0, 25.3, 24.0; HRMS (ESI^+): found MH^+ 459.2024, $[C_{25}H_{32}ClN_2O_4]^+$ requires 459.2045.

Data for the second eluting isomer: R_f 0.35 [petrol:EtOAc:NH_3 aq. 37% (70:30:0.5)]; ^{1}H NMR (400 MHz, $CDCl_3$) δ 7.40–7.27 (10H, m), 6.00 (1H, br s), 5.28–5.02 (4H, m), 4.74–4.58 (1H, m), 4.05–4.96 (1H, m), 2.64 (1H, dd, *J* = 13.1, 5.4 Hz), 2.51 (1H, dd, *J* = 13.1, 8.7 Hz), 2.53–2.24 (5H, m), 2.23–2.05 (1H, m), 1.64–1.47 (4H, m), 1.46–1.33 (2H, m); ^{13}C NMR (126 MHz, $CDCl_3$) δ 172.1, 156.3, 136.3, 135.4, 128.7, 128.6, 128.6, 128.5, 128.4, 128.3, 67.4, 67.4, 67.2, 65.7, 65.7, 55.4, 55.1, 52.4, 39.1, 25.8, 24.2; HRMS (ESI^-): found M^- 458.1972, $[C_{25}H_{31}ClN_2O_4]^-$ requires 458.1972.

(*R*)-((*1S,2R,4S,5R*)-5-(1-Chloro-2-(piperidin-1-yl)ethyl)quinuclidin-2-yl)(quinolin-4-yl)methyl Benzoate (47)

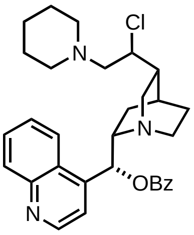

Following **GP1** but using 8.0 equivalents of TFA, **1** (48 μL, 0.48 mmol) and **S6** (159 mg, 0.4 mmol) gave **47** (124 mg, 60%) as an oil. R_f 0.60 [CH_2Cl_2:*i*-PrOH (97:3)]; ^{1}H NMR (500 MHz, $CDCl_3$) δ 8.87 (1H, d, *J* = 4.5 Hz), 8.31 (1H, d, *J* = 8.5 Hz), 8.14 (1H, dd, *J* = 8.5, 1.3 Hz), 8.12–8.06 (2H, m), 7.74 (1H, ddd, *J* = 8.4, 6.8, 1.3 Hz), 7.65 (1H,

ddd, J = 8.3, 6.8, 1.4 Hz), 7.62–7.55 (1H, m), 7.51–7.44 (3H, m), 6.82 (1H, d, J = 6.3 Hz), 3.95 (1H, dt, J = 9.6, 6.1 Hz), 3.53 (1H, dt, J = 9.8, 7.0 Hz), 3.19 (1H, dddd, J = 13.2, 10.3, 5.1, 2.4 Hz), 3.07 (1H, dd, J = 14.2, 9.8 Hz), 2.78 (1H, ddd, J = 14.2, 5.4, 2.5 Hz), 2.70–2.60 (1H, m), 2.58 (2H, d, J = 6.2 Hz), 2.49–2.31 (4H, m), 2.12–2.06 (1H, m), 1.97–1.84 (2H, m), 1.83–1.66 (2H, m), 1.59–1.46 (5H, m), 1.44–1.34 (2H, m); ^{13}C NMR (126 MHz, $CDCl_3$) δ 165.6, 150.1, 148.8, 145.3, 133.6, 130.7, 129.8, 129.8, 129.4, 128.8, 127.2, 126.0, 123.4, 118.5, 74.8, 65.3, 64.8, 60.1, 60.1, 56.7, 55.3, 42.6, 42.2, 28.7, 26.0, 25.8, 24.4, 24.3; HRMS (ESI$^+$): found MH$^+$ 518.2562, $[C_{31}H_{37}ClN_3O_2]^+$ requires 518.2575.

(5*R*)-5-(2-Chloro-1-(piperidin-1-yl)propan-2-yl)-2-methylcyclohex-2-en-1-one (48)

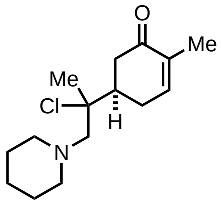

Following **GP1, 1** (24 µL, 0.24 mmol) and (*R*)-carvone (18 µL, 0.2 mmol) gave **48** (45 mg, 83%) as an oil. d.r. 1:1. R_f 0.20 [petrol:EtOAc (9:1)]; FT-IR ν_{max} (film)/cm^{-1}: 2933, 1669, 800; ^{1}H NMR (400 MHz, $CDCl_3$) δ 6.79–6.67 (1H, m), 2.79–2.31 (11H, m), 1.80–1.74 (3H, m), 1.62–1.43 (7H, m), 1.41–1.31 (2H, m); ^{13}C NMR (126 MHz, $CDCl_3$, diastereomers) δ 199.7 (A), 199.6 (B), 144.9 (A), 144.7 (B), 135.2 (A), 135.2 (B), 76.8 (A), 76.7 (B), 67.6 (A), 67.3 (B), 56.9 (A), 56.8 (B), 43.1 (A), 42.7 (B), 40.4 (AB), 39.9 (AB), 39.9, 28.2 (AB), 27.7 (AB), 26.8 (A), 26.6 (B), 26.4 (AB), 24.1 (A), 24.0 (B), 15.7 (AB); HRMS (ESI+): found MH$^+$ 270.1612, $[C_{15}H_{25}ClNO]^+$ requires 270.1625.

1-(2-Chloro-3-(3-(4-chlorophenyl)propoxy)propyl)piperidine (49)

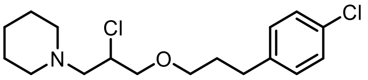

Following **GP1**, **1** (48 µL, 0.48 mmol) and **S7** (84 mg, 0.4 mmol) gave **49** (131 mg, 99%) as an oil. R_f 0.30 [petrol:EtOAc 9:1]; FT-IR ν_{max} (film)/cm^{-1}: 2933, 2854, 1116, 1091; ^{1}H NMR (500 MHz, $CDCl_3$) δ 7.26–7.22 (2H, m), 7.15–7.10 (2H, m), 4.13–4.03 (1H, m), 3.72 (1H, dd, J = 10.5, 4.4 Hz), 3.59 (1H, dd, J = 10.5, 6.2 Hz), 3.54–3.41 (2H, m), 2.71–2.64 (3H, m), 2.59 (1H, dd, J = 13.3, 6.5 Hz), 2.52–2.33 (4H, m), 1.88

(2H, dq, *J* = 9.3, 6.9 Hz), 1.56 (4H, p, *J* = 5.6 Hz), 1.47–1.37 (2H, m); ^{13}C NMR (126 MHz, $CDCl_3$) δ 140.4, 131.6, 130.0, 128.5, 73.4, 70.2, 62.7, 57.8, 55.2, 31.7, 31.2, 26.1, 24.3; HRMS (ESI$^+$): found MH$^+$ 330.1370, $[C_{17}H_{26}Cl_2NO]^+$ requires 330.1392.

1-(4-(*tert*-Butyl)phenyl)-3-chloro-4-(4-(hydroxydiphenylmethyl)piperidin-1-yl)butyl benzoate (50)

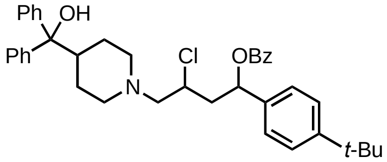

Following **GP2**, α,α-diphenyl-4-piperidinomethanol hydrochloride (36 mg, 0.12 mmol) and **S9** (61 mg, 0.1 mmol) gave **50** (37 mg, 60%) as an oil. d.r. 1:1. R_f 0.40 [petrol:EtOAc (8:2)]; FT-IR ν_{max} (film)/cm^{-1} 2961, 2359, 2341, 1716, 1489, 1447, 1314, 1258, 1093, 1066, 1024; ^{1}H NMR (500 MHz, $CDCl_3$, diastereomers) δ 8.14–7.95 (2H, m), 7.55 (1H, t, *J* = 7.5 Hz), 7.49 (4H, t, *J* = 7.0 Hz), 7.46–7.40 (2.5H, m), 7.40–7.35 (3H, m), 7.35–7.27 (4H, m), 7.23–7.16 (2.5H, m), 6.31 (0.5H, dd, *J* = 10.3, 2.9 Hz), 6.26 (0.5H, t, *J* = 7.4 Hz), 4.35–4.01 (0.5H, m), 3.83–3.63 (0.5H, m), 3.03–2.83 (1.5H, m), 2.83–2.72 (0.5H, m), 2.72–2.58 (3H, m), 2.50–2.33 (1.5H, m), 2.31–2.13 (2H, m), 2.13–2.00 (1H, m), 1.95 (0.5H, td, *J* = 11.0, 3.9 Hz), 1.65–1.41 (4H, m), 1.30 (4.5H, s), 1.29 (4.5H, s); ^{13}C NMR (126 MHz, $CDCl_3$, diastereomers) δ 165.6, 165.5, 151.3, 151.0, 145.9 (x 2), 137.3 (x 2), 136.1 (x 2), 133.0, 132.9, 130.4, 130.3, 129.7, 129.6, 128.4, 128.3, 128.2 (x 2), 128.2, 128.1, 126.7 (x 2), 126.6, 126.5, 126.5, 126.4, 126.0 (x 2), 125.8, 125.7, 125.6, 125.5, 79.6, 79.5, 77.3, 74.6, 73.5, 65.1, 55.7, 55.5, 55.1, 53.8, 53.6, 44.0, 43.9, 43.5, 42.6, 34.6, 34.6, 31.3, 31.3, 29.7, 26.5, 26.5, 26.3, 26.2; HRMS (ASAP): Found MH$^+$ 610.3057 $C_{39}H_{45}NO_3Cl$ requires 610.3082.

3-Chloro-4-(4-(4-chlorophenyl)-4-hydroxypiperidin-1-yl)-1-(4-fluorophenyl)butan-1-one (51)

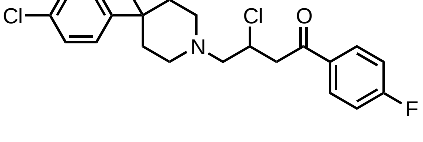

Following **GP1** in CD_2Cl_2 without adding KOH at the end of the reaction, 4-(4-chlorophenyl)-4-hydroxypiperidine (25 mg, 0.12 mmol) and **S11** (16 mg, 0.1 mmol) and gave **51** (30 mg, 73%) as an oil. ^{1}H NMR (500 MHz, CD_2Cl_2) δ 7.99 (2H, dd, *J* =

8.7, 5.4 Hz), 7.44 (2H, d, *J* = 8.9 Hz), 7.37 (2H, d, *J* = 8.5 Hz), 7.25–7.15 (2H, m), 4.94 (1H, p, *J* = 6.4 Hz), 3.89–3.77 (2H, m), 3.75 (1H, dd, *J* = 18.3, 5.4 Hz), 3.70 (2H, t, *J* = 5.9 Hz), 3.67–3.58 (2H, m), 3.56 (1H, dd, *J* = 18.3, 7.3 Hz), 2.64–2.56 (2H, m), 2.18–2.05 (2H, m); ^{13}C NMR (126 MHz, CD_2Cl_2) δ 195.3, 167.14 (d, *J* = 256.5 Hz), 144.1, 132.5 (d, *J* = 2.9 Hz), 131.65 (d, *J* = 9.8 Hz), 129.4, 126.5, 126.4, 116.73 (d, *J* = 22.0 Hz), 69.7, 63.2, 51.9, 49.9, 49.7, 45.2, 35.7, 35.1; ^{19}F NMR (471 MHz, CD_2Cl_2) δ –76.31. LC-MS expected: 410,31; found: 410.1.

7-(2-Chloro-3-((2-hydroxyethyl)(methyl)amino)propyl)-1,3-dimethyl-3,7-dihydro-*1H*-purine-2,6-dione (52)

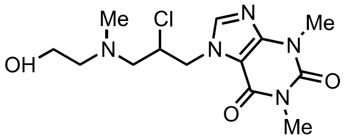

Following **GP1** but adding KPF_6 (18 mg, 0.1 mmol) to the reaction mixture and using $HClO_4$ instead of TFA, 2-(methylamino)ethanol (10 µL, 0.12 mmol) and **S12** (22 mg, 0.1 mmol) and gave **52** (20 mg, 62%) as an oil. R_f 0.50 [petrol:EtOAc (8:2)]; FT-IR ν_{max} (film)/cm^{-1} 3444, 2952, 2360, 1699, 1651, 1604, 1547, 1437, 1456, 1428, 1408, 1377, 1259, 1234, 1191, 1027; ^{1}H NMR (500 MHz, $CDCl_3$,) δ 7.68 (1H, s), 5.17 (1H, dd, *J* = 14.3, 2.5 Hz), 4.42–4.33 (1H, m), 4.05 (1H, dd, *J* = 14.2, 10.0 Hz), 3.65 (2H, br s), 3.60 (3H, s), 3.39 (3H, s), 3.12 (1H, br s), 2.86 (1H, dd, *J* = 13.4, 5.9 Hz), 2.81–2.68 (2H, m), 2.60 (1H, dt, *J* = 13.0, 4.9 Hz), 2.40 (3H, s) 1.83 (1H, br s); ^{13}C NMR (126 MHz, $CDCl_3$) δ 155.4, 151.5, 149.3, 142.6, 106.4, 62.4, 59.8, 59.1, 58.6, 51.9, 42.6, 29.9, 28.1; HRMS (HESI): Found MNa^+ 352.1135 $C_{13}H_{20}N_5O_3ClNa$ requires 352.1147.

1-(3-(2-Benzylphenoxy)-2-chloropropyl)piperidine (102)

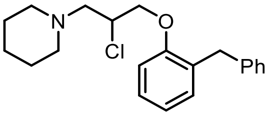

Following **GP1**, **1** (12 µL, 0.12 mmol) and 1-(allyloxy)-2-benzylbenzene **100** (22 mg, 0.1 mmol, 1.00 equiv.) gave **102** as an oil (34 mg, quantitative). R_f = 0.8 [petrol:EtOAc (8:2)]; ^{1}H NMR (500 MHz, $CDCl_3$) δ 7.27–7.20 (4H, m), 7.20–7.13 (2H, m), 7.11 (1H, d, *J* = 7.5 Hz), 6.90 (1H, t, *J* = 7.4 Hz), 6.85 (1H, d, *J* = 8.2 Hz), 4.29–4.13 (3H, m), 4.01 (2H, s), 2.71 (1H, dd, *J* = 13.3, 6.8 Hz), 2.61 (1H, dd, *J* = 13.3, 5.5 Hz), 2.47–2.28

(4H, m), 1.53 (4H, p, *J* = 5.6 Hz), 1.40 (2H, p, *J* = 6.0 Hz); ^{13}C NMR (126 MHz, $CDCl_3$) δ 156.2, 141.1, 130.8, 130.1, 129.1, 128.4, 127.6, 125.9, 121.1, 111.6, 70.3, 62.4, 56.8, 55.2, 36.3, 26.1, 24.3. HRMS (ESI$^+$): found MH$^+$ 344.1775, $[C_{21}H_{27}ClNO]^+$ requires 344.1775.

4.3 Mechanistic Considerations

4.3.1 Protonation of *N*-Chloropiperidine

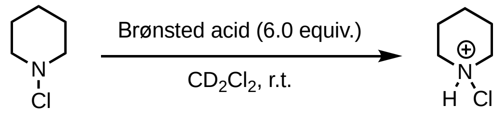

A solution on *N*-chloropiperidine (12 mg, 0.1 mmol, 1.0 equiv.) in CD_2Cl_2 in a dry NMR tube was treated with the acid (0.6 mmol, 6.0 equiv.) and the sample was immediately analysed by ^{1}H NMR spectroscopy.

This study demonstrated that AcOH is not able to protonate *N*-chloropiperidine.

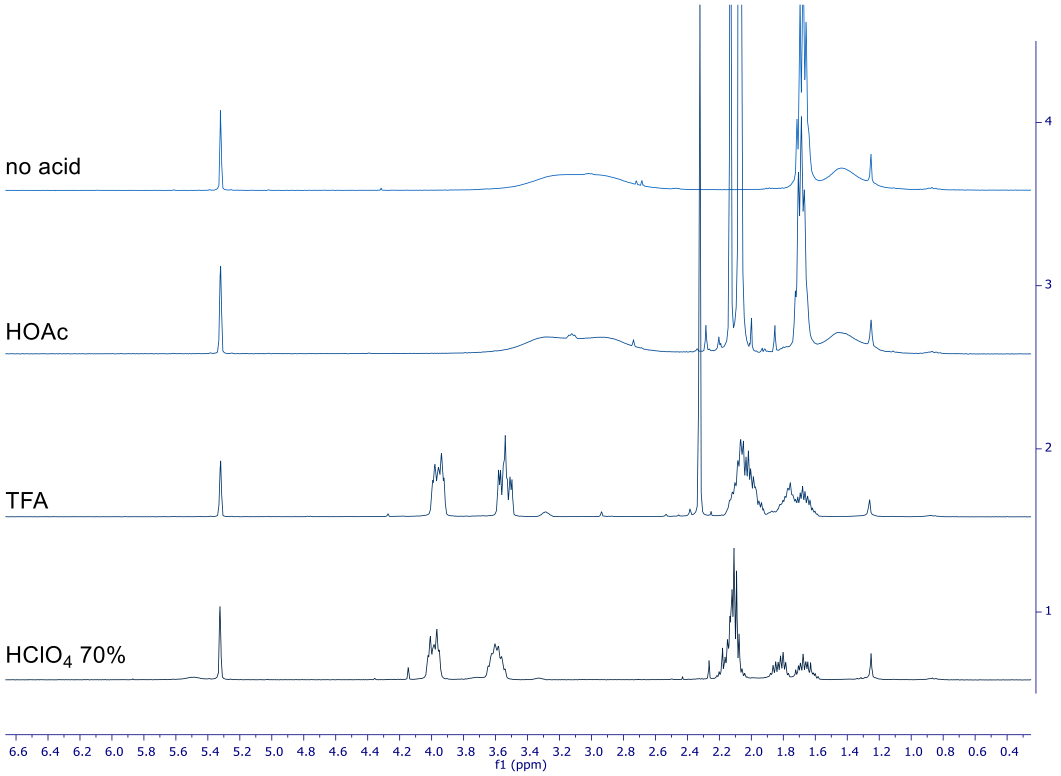

Figure 1.

4.3.2 Quantum Yield Determination

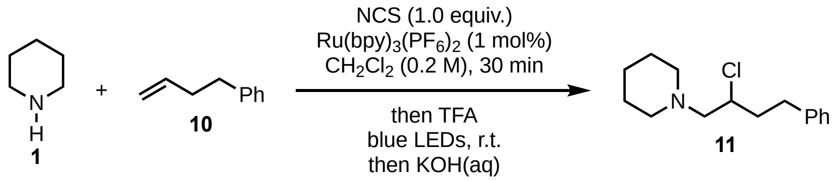

The quantum yield was determined using the method reported by Yoon[11] at three different times. Since the reaction is fully complete after 10 s, this indicates Φ > 200 in CH_2Cl_2 and supports a radical chain mechanism. All yields were determined by ^1H NMR spectroscopy with 1,3,5-trimethoxybenzene as the internal standard.

Table S2.

Entry	Reaction time (s)	11 (%)	10 (%)	Φ
**1**	5	3	95	>200
**2**	10	100	–	>200
**3**	30	100	–	>200

5 Aziridinium Formation and Ring-Opening Studies

5.1 Aziridinium Formation

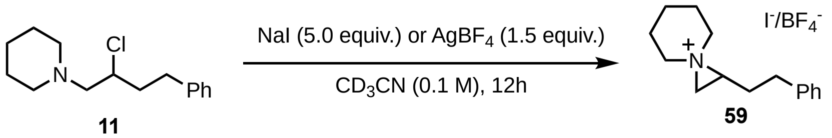

A stock solution of **11** (126 mg, 0.5 mmol, 1.0 equiv.) and 1,3-dinitrobenzene (84 mg, 0.5 mmol, 1.0 equiv.) as the internal standard in CD_3CN (2.5 mL) was prepared and was added to five NMR tubes (3 x 500 µL):

- **A.** Tube 1: nothing else added.
- **B.** Tube 2: 1.5 equiv. of NaI (23 mg, 0.15 mmol, 1 M solution in CD_3CN)
- **C.** Tube 3: 3.0 equiv. of NaI (45 mg, 0.30 mmol, 1 M solution in CD_3CN)
- **D.** Tube 4: 5.0 equiv. of NaI (75 mg, 0.50 mmol, 1 M solution in CD_3CN)
- **E.** Tube 5: 1.5 equiv. of $AgBF_4$ (29 mg, 0.15 mmol, 1.5 equiv.)

^{1}H NMR spectra were acquired at regular intervals (Figure 2).

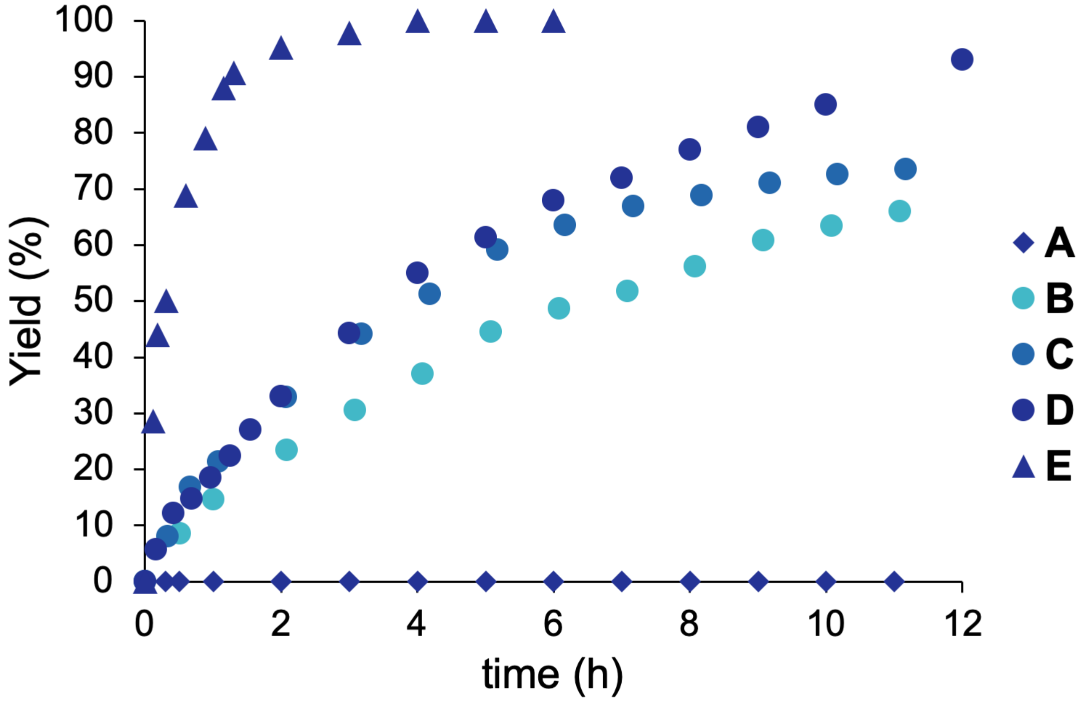

Figure 2.

Data for **59**: ^{1}H NMR (400 MHz, CD_3CN) δ 7.34 (2H, dd, *J* = 8.0, 6.7 Hz), 7.31–7.21 (3H, m), 3.22 (1H, dt, *J* = 12.9, 6.4 Hz), 3.13–2.99 (3H, m), 2.98–2.79 (4H, m), 2.64 (1H, dd, *J* = 7.4, 3.7 Hz), 2.40–2.22 (1H, m), 2.07–1.97 (1H, m), 1.86–1.65 (5H, m); 1.64–1.49 (1H, m); ^{13}C NMR (101 MHz, CD_3CN) δ 141.0, 129.7, 129.5, 127.6, 61.5, 53.8, 53.2, 43.6, 33.0, 28.1, 23.9, 23.7, 22.2.

The putative β-iodoamine intermediate **S13** involved in the formation of **59** was detected by positive ESI MS analysis of the mixture in the NMR tube in the presence of 1% HCOOH (Figure 3).

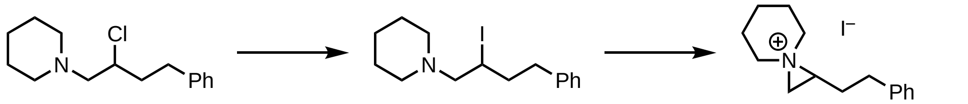

11 Chemical Formula: $C_{15}H_{22}ClN$ Exact Mass: 251,14

S13 Chemical Formula: $C_{15}H_{22}IN$ Exact Mass: 343,08

59 Chemical Formula: $C_{15}H_{22}N^+$ Exact Mass: 216,17

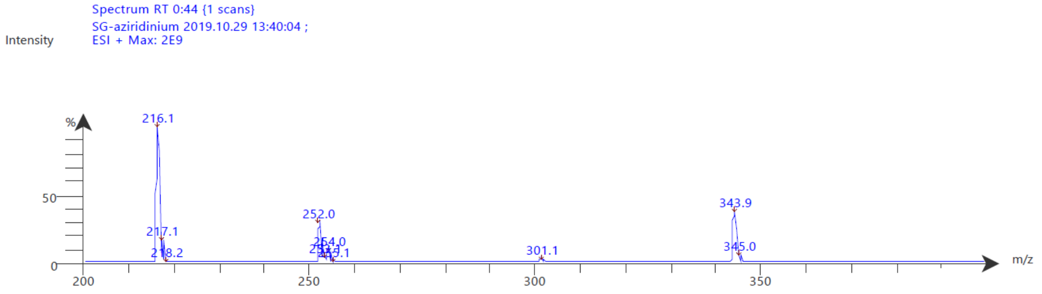

Figure 3.

5.2 Aziridinium Ring-Opening

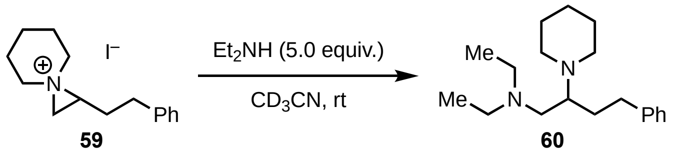

A CD_3CN solution of **59** was treated with Et_2NH (5.0 equiv.) and an ^{1}H NMR spectrum was recorded after 5 minutes showing complete conversion into the diamine **60** (Figure 4).

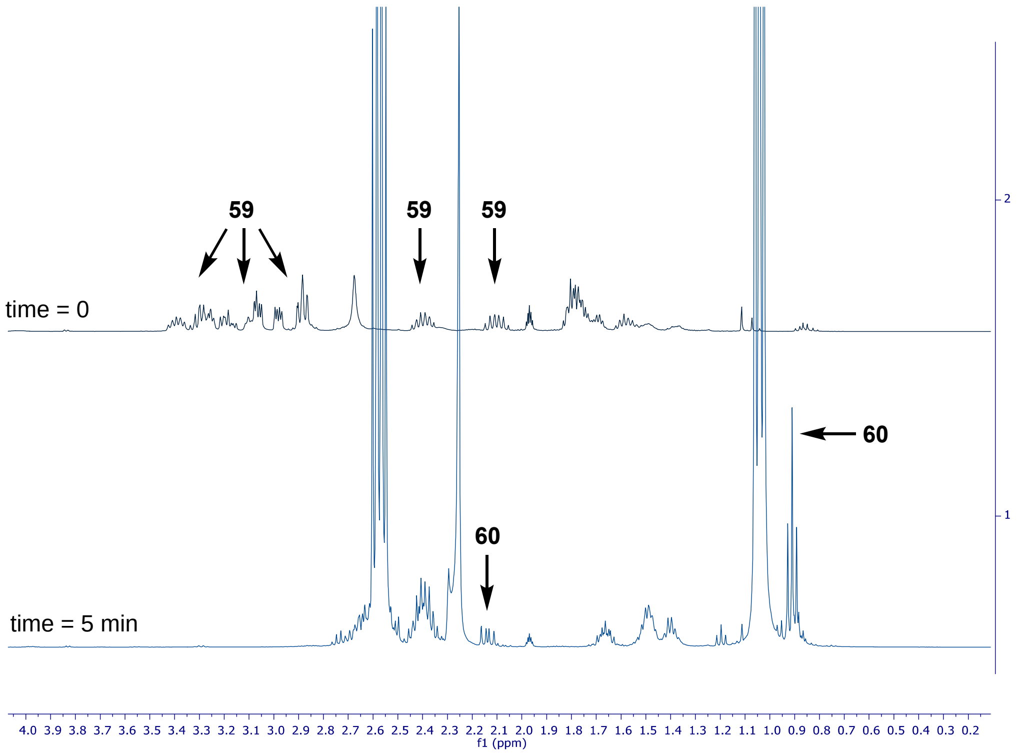

Figure 4.

6 Olefin Diamination

6.1 Reaction Optimization

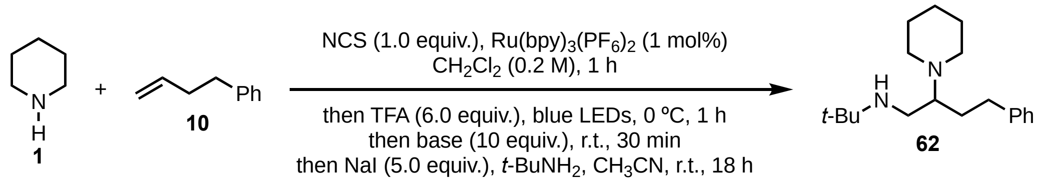

An oven-dried tube equipped with a stirring bar was capped with a Supelco aluminium crimp seal with septum (PTFE/butyl), evacuated and refilled with N_2 (x3) and charged with piperidine **1** (10 μL, 0.1 mmol, 1.0 equiv.). $Ru(bpy)_3(PF_6)_2$ (0.4 mg, 0.5 μmol, 0.5 mol%) and NCS (15 mg, 0.11 mmol, 1.1 equiv.) as a stock solution in CH_2Cl_2 (0.5 mL, dry and degassed by bubbling through with N_2 for 20 min). The mixture was stirred for 1 h at room temperature. The mixture was cooled to 0 ºC and 4-Phenylbutene **10** (15 μL, 0.1 mmol, 1.0 equiv.) was added as a solution in CH_2Cl_2 (0.5 mL) followed by TFA (46 μL, 0.6 mmol, 6 equiv.). The blue LEDs were switched on and the mixture was stirred under irradiation for 1 h at 0 °C. The base (10.0 equiv.) was added and the reaction was warmed to room temperature and stirred for 30 minutes. A solution of NaI (75 mg, 0.5 mmol, 5.0 equiv) and *tert*-butylamine in CH_3CN (0.5 mL) was added and the mixture was stirred for 18 h at room temperature. Aqueous 1 M KOH (3 mL), EtOAc (3 mL) and 1,3,5-trimethoxybenzene (17 mg, 0.1 mmol, 1.0 equiv.) were added and the layers were separated. The aqueous layer was extracted with EOAc (3 x 3 mL), the combined organic layers were dried ($MgSO_4$), filtered and evaporated. $CDCl_3$ (0.4 mL) was added and the mixture was analysed by ^1H NMR spectroscopy to determine the NMR yield.

Table **S3** reports all the experiments performed. The conditions of entry 8 were chosen to run all one-pot diamination reactions.

Table S3.

Entry	base	*t*-BuNH2 (equiv.)	Yield (%)
**1**	–	2.5	–
**2**	–	10	87
**3**	HMDS	2.5	–
**5**	DIPEA	2.5	78
**6**	DIPEA	5.0	86

6.2 Reaction Scope

General Procedure for the Olefin Diamination Using Free Amines – GP3

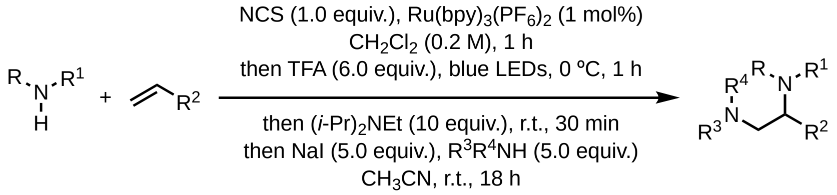

A dry tube equipped with a stirring bar was charged with NCS (1.0 equiv.), $Ru(bpy)_3(PF_6)_2$ (1 mol%) and the amine if solid (1.2 equiv.). The tube was capped with a Supelco aluminium crimp seal with septum (PTFE/butyl), evacuated and refilled with N_2 (x 3). CH_2Cl_2 (0.2 M) (dry and degassed by bubbling through with N_2 for 20 min) and the amine (1.2 equiv.) if liquid were added and the mixture was stirred for 1 h at room temperature. The mixture was cooled to 0 °C and a solution of the olefin (1.0 equiv.) in CH_2Cl_2 (0.2 M) and TFA (6 equiv.) were added. The LEDs were immediately switched on. The mixture was stirred under irradiation for 1 h at 0 °C. (*i*-Pr)$_2$NEt (10.0 equiv.) was added and the mixture stirred at room temperature for 30 min. As solution of the second amine (5.0 equiv.) and NaI (5.0 equiv.)[1] in CH_3CN (1 M) was added and the mixture was stirred at room temperature for 18 h. Aqueous 1 M KOH (3 mL) and EtOAc (3 mL) were added. The layers were separated, and the aqueous layer was extracted with EtOAc (3 x 3 mL). The combined organic layers were dried ($MgSO_4$), filtered and evaporated. Purification by flash column or preparative TLC chromatography on silica gel gave the products.

While this procedure afforded the desired products in all instances, in a few cases we have slightly modified the elaboration of the intermediate *N*-chloroamine to improve the reaction yield.

Alternative General Procedure for the Olefin Diamination Using Free Amines – GP3'

A dry tube equipped with a stirring bar was charged with NCS (1.0 equiv.), $Ru(bpy)_3(PF_6)_2$ (1 mol%) and the amine if solid (1.2 equiv.). The tube was capped with a Supelco aluminium crimp seal with septum (PTFE/butyl), evacuated and refilled with

[1] If the amine is available as a hydrochloride salt, the amount of NaI should be adjusted to account for the precipitation of insoluble NaCl.

N_2 (x 3). CH_2Cl_2 (0.2 M) (dry and degassed by bubbling through with N_2 for 20 min) and the amine (1.2 equiv.) if liquid were added and the mixture was stirred for 1 h at room temperature. The mixture was cooled to 0 °C and a solution of the olefin (1.0 equiv.) in CH_2Cl_2 (0.2 M) and TFA (6 equiv.) were added. The LEDs were immediately switched on. The mixture was stirred under irradiation for 1 h at 0 °C. Aqueous 1 M KOH (3 mL) and EtOAc (3 mL) were added. The layers were separated, and the aqueous layer was extracted with EtOAc (3 x 3 mL). The combined organic layers were dried ($MgSO_4$), filtered and evaporated. The crude was dissolved in CH_3CN (0.1 M, 1.0 mL), the second amine (5.0 equiv.) and NaI (5.0 equiv.) were added and the mixture was stirred at room temperature for 18 h. Aqueous 1 M KOH (3 mL) and EtOAc (3 mL) were added. The layers were separated, and the aqueous layer was extracted with EtOAc (3 x 3 mL). Purification by flash column or preparative TLC chromatography on silica gel gave the products.

_N,N_-Diethyl-4-phenyl-2-(piperidin-1-yl)butan-1-amine (60)

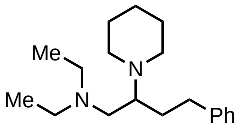

Following **GP3**, **1** (10 μL, 0.1 mmol, 1.0 equiv.), **10** (15 μL, 0.1 mmol, 1.0 equiv.) and Et_2NH (52 μL, 0.5 mmol, 5.0 equiv.) gave **60** (23 mg, 78%) as an oil. R_f 0.15 [CH_2Cl_2:$MeNO_2$:MeOH (3:1:1)]; FT-IR ν_{max} (film)/cm^{-1}: 2928, 2852, 2794, 1453, 1381; ^{1}H NMR (400 MHz, $CDCl_3$) δ 7.26 (2H, t, *J* = 7.4 Hz), 7.22–7.18 (2H, m), 7.18–7.11 (1H, m), 2.82–2.37 (12H, m), 2.22 (1H, dd, *J* = 12.6, 7.2 Hz), 1.80–1.66 (2H, m), 1.62–1.48 (4H, m), 1.47–1.36 (2H, m), 0.99 (6H, t, *J* = 7.1 Hz); ^{13}C NMR (101 MHz, $CDCl_3$) δ 143.3, 128.6, 128.3, 125.6, 62.1, 53.0, 49.8, 47.7, 33.6, 31.9, 26.8, 25.3, 11.8.; HRMS (ESI$^+$): found MH+ 289.2628, $[C_{19}H_{33}N_2]^+$ requires 289.2644.

4-(4-Phenyl-2-(piperidin-1-yl)butyl)morpholine (61)

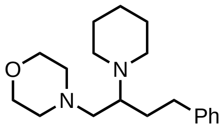

Following **GP3**, **1** (10 μL, 0.1 mmol, 1.0 equiv.), **10** (15 μL, 0.1 mmol, 1.0 equiv.) and morpholine (44 μL, 0.5 mmol, 5.0 equiv.) gave **61** (29 mg, quantitative) as an oil. R_f 0.63 [CH_2Cl_2:$MeNO_2$:MeOH (8:1:1)]; FT-IR ν_{max} (film)/cm^{-1} 2929, 2850, 1453, 1116;

^{1}H NMR (400 MHz, $CDCl_3$) δ 7.30–7.24 (2H, m), 7.24–7.13 (3H, m), 3.66 (4H, t, *J* = 4.7 Hz), 2.82–2.61 (5H, m), 2.59–2.41 (5H, m), 2.40–2.32 (2H, m), 2.19 (1H, dd, *J* = 12.5, 6.9 Hz), 1.92–1.77 (1H, m), 1.74–1.65 (1H, m), 1.65–1.51 (4H, m), 1.46 (2H, p, *J* = 5.8 Hz); ^{13}C NMR (126 MHz, $CDCl_3$) δ 142.8, 128.7, 128.4, 125.8, 67.3, 60.6, 59.2, 54.3, 50.0, 33.4, 32.2, 26.6, 25.0; HRMS (ESI$^+$): found MNa+ 325.2239, $[C_{19}H_{30}N_2ONa]^+$ requires 325.2250.

N-(tert-Butyl)-4-phenyl-2-(piperidin-1-yl)butan-1-amine (62)

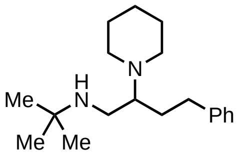

Following **GP3**, **1** (10 μL, 0.1 mmol, 1.0 equiv.), **10** (15 μL, 0.1 mmol, 1.0 equiv.) and *tert*-butylamine (53 μL, 0.5 mmol, 5.0 equiv.) gave **62** as an oil (25 mg, 86%). R_f 0.3 [CH_2Cl_2:$MeNO_2$:MeOH (3:1:1)]; FT-IR ν_{max} (film)/cm^{-1}: 2929, 2852, 2799, 1495, 1452, 1441; ^{1}H NMR (500 MHz, $CDCl_3$) δ 7.27 (2H, d, *J* = 6.3 Hz), 7.21–7.11 (3H, m), 2.72–2.45 (7H, m), 2.39–2.27 (2H, m), 2.26–1.98 (1H, br s), 1.92 (1H, ddd, *J* = 16.8, 9.4, 5.1 Hz), 1.62–1.51 (2H, m), 1.51–1.35 (5H, m), 1.12 (9H, s); ^{13}C NMR (126 MHz, $CDCl_3$) δ 142.7, 128.5, 128.4, 125.9, 64.0, 50.2, 49.3, 42.6, 34.1, 29.2, 29.0, 27.0, 25.2; HRMS (ESI$^+$): found MH+ 289.2628, $[C_{19}H_{33}N_2]^+$ requires 289.2644.

4-Phenyl-2-(piperidin-1-yl)-N-(2,2,2-trifluoroethyl)butan-1-amine (63) and 4-phenyl-1-(piperidin-1-yl)-N-(2,2,2-trifluoroethyl)butan-2-amine (63')

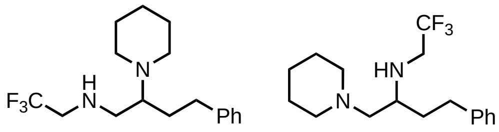

Following **GP3**, **1** (10 μL, 0.1 mmol, 1.0 equiv.), **10** (15 μL, 0.1 mmol, 1.0 equiv.) and 2,2,2-trifluoroethan-1-amine (39 μL, 0.5 mmol, 5.0 equiv.) gave a mixture of **63** and **63** (21 mg, 68%) as an oil. **63**:**63'** = 3:1.

Data for **63**: R_f 0.50 [CH_2Cl_2:$MeNO_2$:MeOH (10:1:1)]; FT-IR ν_{max} (film)/cm^{-1}: 3335, 2929, 2853, 2802, 1142; ^{1}H NMR (400 MHz, $CDCl_3$) δ 7.32–7.26 (2H, m), 7.22–7.14 (3H, m), 3.18 (2H, q, *J* = 11.1, 10.0 Hz), 2.74–2.66 (2H, m), 2.66–2.47 (6H, m), 2.38–2.28 (2H, m), 1.97–1.85 (1H, m), 1.61–1.47 (3H, m), 1.47–1.35 (4H, m); ^{13}C NMR (126 MHz, $CDCl_3$) δ 142.4, 128.5, 128.4, 126.0 (q, *J* = 279.8 Hz), 126.0, 63.7, 50.9 (q, *J* =

30.7 Hz), 49.6, 49.5, 33.9, 28.7, 26.9, 25.1; ^{19}F NMR (376 MHz, $CDCl_3$) δ –71.4; HRMS (ESI$^+$): found MH+ 315.2035, $[C_{17}H_{26}F_3N_2]^+$ requires 315.2043.

N-(4-phenyl-2-(piperidin-1-yl)butyl)aniline (64) and *N*-(4-phenyl-1-(piperidin-1-yl)butan-2-yl)aniline (64')

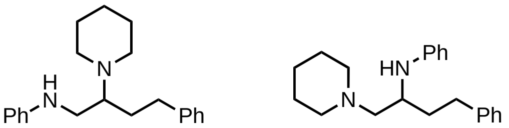

Following **GP3**, **1** (10 μL, 0.1 mmol, 1.0 equiv.), **10** (15 μL, 0.1 mmol, 1.0 equiv.) and aniline (48 μL, 0.5 mmol, 5.0 equiv.) gave a mixture of **64** and **64'** (1.7:1) (19 mg, 60%) as oils. **64**:**64'** = 1.7:1.

Data for **64**: R_f 0.56 [CH_2Cl_2:$MeNO_2$:MeOH (10:1:1)]; FT-IR ν_{max} (film)/cm^{-1}: 3354, 2926, 2850, 1602, 1505; ^{1}H NMR (500 MHz, $CDCl_3$) δ 7.33–7.26 (2H, m), 7.23–7.15 (5H, m), 6.69 (1H, t, *J* = 7.3 Hz), 6.64 (2H, d, *J* = 7.9 Hz), 3.20 (1H, dd, *J* = 11.2, 4.7 Hz), 2.86 (1H, t, *J* = 10.7 Hz), 2.77–2.66 (2H, m), 2.63–2.53 (3H, m), 2.42–2.30, (2H, m), 2.05–1.94 (1H, m), 1.64–1.48 (6H, m), 1.46–1.40 (2H, m); ^{13}C NMR (126 MHz, $CDCl_3$) δ 149.0, 142.3, 129.4, 128.6, 128.4, 126.1, 117.1, 113.2, 63.0, 49.2, 44.0, 38.8, 28.7, 27.0, 25.1; HRMS (ESI$^+$): found MH+ 309.2320, $[C_{21}H_{29}N_2]^+$ requires 309.2325.

Data for **64'**: R_f 0.40 [CH_2Cl_2:$MeNO_2$:MeOH (10:1:1)]; FT-IR ν_{max} (film)/cm^{-1}: 3354, 2926, 2850, 1602, 1505; ^{1}H NMR (400 MHz, $CDCl_3$) δ 7.25–7.18 (2H, m), 7.15–7.09 (3H, m), 7.10–7.04 (2H, m), 6.65–6.58 (1H, m), 6.55–6.47 (2H, m), 4.48–3.91 (1H, br s), 3.49–3.28 (1H, m), 2.65 (2H, dd, *J* = 9.4 Hz, 7.1 Hz), 2.53–2.43 (1H, m), 2.42–2.23 (5H, m), 1.89–1.79 (1H, m), 1.60–1.41 (4H, m), 1.41–1.31 (2H, m); ^{13}C NMR (126 MHz, $CDCl_3$) δ 148.3, 142.1, 129.2, 128.5, 128.4, 125.9, 117.2, 113.4, 62.2, 54.7, 49.7, 35.4, 31.9, 25.9, 24.2; HRMS (ESI$^+$): found MH$^+$ 309.2320, $[C_{21}H_{29}N_2]^+$ requires 309.2325.

N-(4-phenyl-2-(piperidin-1-yl)butyl)-2,3-dihydrobenzo[*d*]thiazol-2-amine (65)

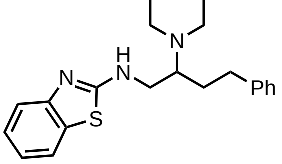

Following **GP3'**, **1** (10 μL, 0.1 mmol, 1.0 equiv.), **10** (15 μL, 0.1 mmol, 1.0 equiv.) and 2-aminobenzothiazole (75 mg, 0.5 mmol, 5.0 equiv.) gave **65** (28 mg, 77%) as an oil.

R_f 0.25 [CH_2Cl_2:$MeNO_2$:MeOH (10:1:1)]; FT-IR ν_{max} (film)/cm^{-1} 3331, 2929, 2851, 1605, 1117; ^{1}H NMR (500 MHz, $CDCl_3$) δ 7.25–7.18 (3H, m), 7.18–7.10 (4H, m), 6.96 (1H, t, J = 7.6 Hz), 6.80 (1H, d, J = 8.1 Hz), 4.05–3.94 (2H, m), 3.08–2.99 (1H, m), 2.78–2.69 (1H, m), 2.69–2.56 (5H, m), 1.98–1.87 (1H, m), 1.73–1.62 (1H, m), 1.60–1.46 (5H, m), 1.46–1.38 (2H, m); ^{13}C NMR (126 MHz, $CDCl_3$) δ 162.4, 142.6, 141.2, 128.5, 128.4, 126.1, 125.7, 122.7, 121.5, 121.4, 109.9, 60.9, 49.9, 33.4, 31.1, 30.9, 26.9, 25.1; HRMS (ESI$^+$): found MH+ 366.2005, [$C_{22}H_{28}N_3S$]$^+$ requires 366.1998.

1-(4-phenyl-1-(1*H*-pyrazol-1-yl)butan-2-yl)piperidine (**66**)

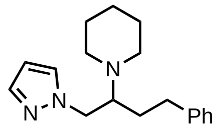

Following **GP3**, **1** (10 µL, 0.1 mmol, 1.0 equiv.), **10** (15 µL, 0.1 mmol, 1.0 equiv.) and pyrazole (34.0 mg, 0.5 mmol, 5.0 equiv.) gave **66** (12 mg, 43%) as an oil. R_f 0.62 [CH_2Cl_2:$MeNO_2$:MeOH (10:1:1)]; FT-IR ν_{max} (film)/cm^{-1}: 2931, 2852, 2801, 1512, 749; ^{1}H NMR (500 MHz, $CDCl_3$) δ 7.49 (1H, d, J = 1.8 Hz), 7.39 (1H, d, J = 2.2 Hz), 7.28–7.22 (2H, m), 7.18–7.10 (3H, m), 6.22 (1H, t, J = 2.1 Hz), 4.28 (1H, dd, J = 13.7, 6.9 Hz), 4.00 (1H, dd, J = 13.7, 6.8 Hz), 2.98–2.92 (1H, m), 2.73–2.63 (1H, m), 2.57–2.44 (5H, m), 1.89–1.76 (1H, m), 1.57–1.46 (5H, m), 1.46–1.38 (2H, m); ^{13}C NMR (126 MHz, $CDCl_3$) δ 142.5, 139.1, 130.0, 128.6, 128.4, 125.8, 105.4, 64.1, 52.2, 49.5, 33.1, 31.0, 26.9, 25.1; HRMS (ESI$^+$): found MH+ 284.2116, [$C_{18}H_{26}N_3$]$^+$ requires 284.2120.

4-Phenyl-1-(4-phenyl-2-(piperidin-1-yl)butyl)pyridin-1-ium iodide (67)

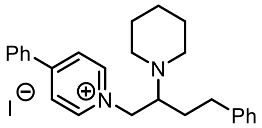

Following **GP3'**, **1** (10 µL, 0.1 mmol, 1.0 equiv.), **10** (15 µL, 0.1 mmol, 1.0 equiv.) and 4-phenylpyridine (78 mg, 0.5 mmol, 5.0 equiv.) gave **67** (29 mg, 79%) as a solid. R_f 0.73 [CH_2Cl_2:$MeNO_2$:MeOH (10:1:1)]; FT-IR ν_{max} (film)/cm^{-1} 2930, 2852, 1636, 1452; ^{1}H NMR (500 MHz, $CDCl_3$) δ 9.12 (2H, d, J = 6.6 Hz), 8.05 (2H, d, J = 6.6 Hz), 7.73 (2H, d, J = 6.9 Hz), 7.60–7.51 (3H, m), 7.28–7.22 (5H, m), 5.03 (1H, dd, J = 13.3, 3.5 Hz), 4.64–4.55 (1H, m), 2.91–2.84 (1H, m), 2.85–2.74 (4H, m), 2.24–2.18 (2H, m), 2.07–1.97 (1H, m), 1.70–1.65 (1H, m), 1.39–1.35 (6H, m); ^{13}C NMR (126 MHz, $CDCl_3$) δ 156.2, 145.5, 141.3, 133.8, 132.5, 130.1, 128.7, 128.6, 127.9, 126.3, 123.7,

66.0, 60.7, 49.9, 33.5, 28.6, 26.7, 24.6; HRMS (ESI$^+$): found M+ 371.2499, $[C_{26}H_{31}N_2]^+$ requires 371.2482.

8-(2-(Azepan-1-yl)-4-phenylbutyl)-8-azabicyclo[3.2.1]octan-3-one (68)

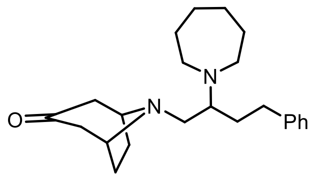

Following **GP3** but warming the reaction to 60 ºC after the addition of NaI (10.0 equiv.) and the second amine, azepane (11 µL, 0.1 mmol, 1.0 equiv.), **10** (15 µL, 0.1 mmol, 1.0 equiv.) and nortropinone hydrochloride (81 mg, 0.5 mmol, 5.0 equiv.) gave **68** (32 mg, 90%) as an oil. R_f 0.41 [CH_2Cl_2:MeNO2:MeOH (10:1:1)]; FT-IR ν_{max} (film)/cm^{-1} 2923, 2852, 1713, 1452; ^{1}H NMR (500 MHz, $CDCl_3$) δ 7.30–7.26 (2H, m), 7.24–7.20 (2H, m), 7.17 (1H, t, *J* = 7.2 Hz), 3.43 (2H, d, *J* = 16.8 Hz), 2.93–2.77 (3H, m), 2.74–2.66 (2H, m), 2.67–2.48 (5H, m), 2.36–2.28 (1H, m), 2.36–2.28 (2H, m), 2.05–1.81 (4H, m), 1.71–1.54 (10H, m); ^{13}C NMR (126 MHz, $CDCl_3$) δ 207.3, 143.1, 128.6, 128.4, 125.7, 64.3, 59.6, 51.7, 48.2, 47.9, 33.5, 30.5, 28.4, 27.8, 27.2 (the following signals were missing in the ^{13}C NMR and were identified by analysing the ^{1}H–^{13}C HMBC (500 MHz, $CDCl_3$) δ 207.3, 143.1, 30.5); HRMS (ESI$^+$): found MH$^+$ 355.2731, $[C_{23}H_{35}N_2O]^+$ requires 355.2744.

5-(*tert*-Butylamino)-4-(2,6-*syn*-dimethylmorpholino)pentanenitrile (69)

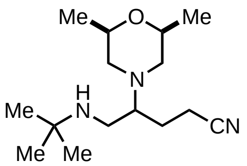

Following **GP3’** but on the compound purified by flash chromatography and warming the reaction to 60 ºC, 2,6-*syn*-dimethylmorpholine (12.5 µL, 0.1 mmol, 1.0 equiv.), pent-4-enenitrile (10 µL, 0.1 mmol, 1.0 equiv.) and *tert*-butylamine (53 µL, 0.5 mmol, 5.0 equiv.) gave **69** (27 mg, quant.) as an oil. dr 1:1. R_f 0.4 [CH_2Cl_2:acetone (1:1)]; FT-IR ν_{max} (film)/cm^{-1}: 2965, 2866, 1453, 1362, 1322, 1259, 1230, 1143, 1084, 1028; ^{1}H NMR (500 MHz, $CDCl_3$) δ 3.64–3.54 (2H, m), 2.70 (1H, dd, *J* = 11.1, 7.3 Hz), 2.64–2.43 (4H, m), 2.42 (2H, t, *J* = 7.4 Hz), 2.14 (2H, q, *J* = 10.0 Hz), 2.10 (1H, br s), 1.88–1.78 (1H, m), 1.78–1.68 (1H, m), 1.14 (6H, d, *J* = 6.2 Hz), 1.09 (9H, s); ^{13}C NMR (126

MHz, $CDCl_3$) δ 120.0, 72.4, 72.4, 63.2, 54.7, 54.6, 50.7, 41.5, 29.0, 24.5, 19.2, 19.2, 15.1; HRMS (ASAP): found MH^+ 268.2383, $C_{15}H_{30}N_3O$ requires 268.2383.

5-(*tert*-Butylamino)-4-(diethylamino)pentanenitrile (70)

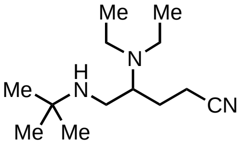

Following **GP3** at 60 °C, diethylamine (10.5 µL, 0.1 mmol, 1.0 equiv.), pent-4-enenitrile (10 µL, 0.1 mmol, 1.0 equiv.) and *tert*-butylamine (53 µL, 0.5 mmol, 5.0 eq.) gave **70** (12 mg, 52%) as an oil. R_f 0.6 [CH_2Cl_2:acetone (1:1)]; FT-IR ν_{max} (film)/cm^{-1}: 2870, 2243, 1702, 1474, 1447, 1381, 1299, 1259, 1232, 1205, 1059; ^{1}H NMR (500 MHz, $CDCl_3$) δ 2.81 (1H, p, *J* = 6.8 Hz), 2.63 (1H, dd, *J* = 11.1, 8.1 Hz), 2.59–2.53 (1H, m), 2.51 (2H, q, *J* = 7.1 Hz), 2.47 (2H, q, *J* = 7.0 Hz), 2.45–2.38 (2H. m), 1.89–1.77 (1H, m), 1.77–1.65 (1H, m), 1.15 (9H, s), 1.04 (6H, t, *J* = 7.1 Hz); ^{13}C NMR (126 MHz, $CDCl_3$) δ 120.1, 58.9, 51.6, 43.3, 42.1, 28.7, 25.1, 15.2, 15.0; HRMS (ASAP): found MH^+ 226.2279 $C_{13}H_{28}N_3$ requires 226.2278.

***N,N*-Dimethyl-4-phenyl-1-thiomorpholinobutan-2-amine** (**71**)

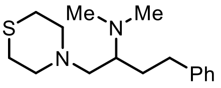

Following **GP3** but warming the reaction to 60 ºC after the addition of NaI (6.0 equiv.) and the second amine, dimethylamine hydrochloride (8 mg, 0.1 mmol, 1.0 equiv.), **10** (15 µL, 0.1 mmol, 1.0 equiv.) and thiomorpholine (50 µL, 0.5 mmol, 5.0 equiv.) gave **71** (22 mg, 80%) as an oil. R_f 0.34 [CH_2Cl_2:$MeNO_2$:MeOH (10:1:1)]; FT-IR ν_{max} (film)/cm^{-1} 2924, 2852, 1670, 1454; ^{1}H NMR (500 MHz, $CDCl_3$) δ 7.31–7.26 (2H, m), 7.23–7.15 (3H, m), 2.73–2.61 (10H, m), 2.61–2.50 (2H, m), 2.31 (6H, s), 2.21–2.14 (1H, m), 1.82–1.71 (1H, m), 1.66 (1H, ddt, *J* = 13.7, 9.5, 6.3 Hz); ^{13}C NMR (126 MHz, $CDCl_3$) δ 142.2, 128.6, 128.5, 126.0, 60.3, 59.3, 55.8, 40.8, 33.3, 31.5, 28.1 (the following signal was missing in the ^{13}C NMR and were identified by analysing the ^{1}H–^{13}C HMBC (500 MHz, $CDCl_3$) 142.2; HRMS (ESI$^+$): found MH^+ 279.1879, $[C_{16}H_{27}N_2S]^+$ requires 279.1889.

Methyl 5-morpholino-4-(piperidin-1-yl)pentanoate (**72**)

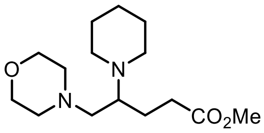

Following **GP3** but warming the reaction to 60 ºC after the addition of NaI and the second amine, **1** (10 µL, 0.1 mmol, 1.0 equiv.), methyl pent-4-enoate (12 µL, 0.1 mmol, 1.0 equiv.) and morpholine (45 µL, 0.5 mmol, 5.0 equiv.) gave **72** (26 mg, 92 %) as an oil. R$_f$ 0.50 [CH_2Cl_2:$MeNO_2$:MeOH (10:1:1)]; FT-IR ν_{max} (film)/cm^{-1} 2930, 2851, 1737, 1440, 1118; ^{1}H NMR (500 MHz, $CDCl_3$) δ ^{1}H NMR ($CDCl_3$, 500 MHz) δ 3.73–3.62 (7H, m), 2.72–2.62 (2H, m), 2.61–2.54 (1H, m), 2.53–2.43 (4H, m), 2.43–2.31 (5H, m), 2.13 (1H, dd, *J* = 12.3, 7.9 Hz), 1.80–1.67 (2H, m), 1.55–1.44 (4H, m), 1.44–1.37 (2H, m); ^{13}C NMR (126 MHz, $CDCl_3$) δ 174.9, 67.2, 60.8, 59.1, 54.4, 51.5, 49.8, 31.9, 26.8, 25.7, 25.1; HRMS (ESI$^+$): found MH+ 285.2161, [$C_{15}H_{29}N_2O_3$]$^+$ requires 285.2173.

***N*-(3-(Azetidin-1-yl)-2-(piperidin-1-yl)propyl)-4-methylbenzenesulfonamide (73)**

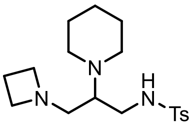

Following **GP3** but using 10 equiv. of NaI and 15 equiv. of (*i*-Pr)$_2$NEt, **1** (10 µL, 0.1 mmol, 1.0 equiv.), **S3** (33 mg, 0.1 mmol, 1.0 equiv.) and azetidine hydrochloride (47 mg, 0.5 mmol, 5.0 equiv.) gave **73** (19 mg, 55%) as an oil. R$_f$ 0.15 [CH_2Cl_2:MeOH:$MeNO_2$ (3:1:1)]; ^{1}H NMR (500 MHz, $CDCl_3$, NH missing) δ 7.76 (2H, d, *J* = 8.2 Hz), 7.30 (2H, d, *J* = 8.1 Hz), 3.21 (4H, t, *J* = 6.9 Hz), 2.92 (1H, m), 2.81 (1H, dd, *J* = 12.4, 4.0 Hz), 2.53 (1H, dd, *J* = 12.4, 7.2 Hz), 2.41 (3H, s), 2.31–2.23 (2H, m), 2.13–2.00 (4H, m), 1.96 (2H, dd, *J* = 11.1, 5.8 Hz), 1.34 (6H, br s); ^{13}C NMR (126 MHz, $CDCl_3$) δ 143.4, 136.6, 129.7, 127.5, 62.0, 60.3, 56.5, 54.3, 49.4, 26.1, 24.2, 21.7, 18.1. HRMS (ESI$^+$): found MH$^+$ 352.2053, [$C_{18}H_{30}N_3O_2S$]$^+$ requires 352.2053.

2,4,5-Tris(benzyloxy)-6-((benzyloxy)methyl)-*N*-(4-phenyl-2-(piperidin-1-yl)butyl)tetrahydro-*2H*-pyran-3-amine (74)

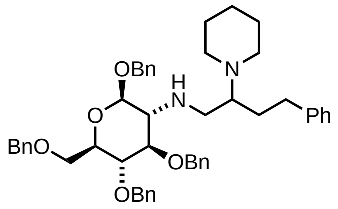

Following **GP3** but using 7.5 equiv. NaI, **1** (10 μL, 0.1 mmol, 1.0 equiv.), **10** (15 μL, 0.1 mmol, 1.0 equiv.) and 2-amino-2-deoxy-3,4,6-tri-*O*-benzyl-β-D-glucopyranoside hydrochloride (144 mg, 0.25 mmol, 2.5 equiv.) gave **74** (61 mg, 81%) as an oil. d.r. 1:1. R_f 0.50 [CH_2Cl_2:$MeNO_2$:MeOH (10:1:1)]; FT-IR ν_{max} (film)/cm^{-1} 2930, 2853, 1453, 1095, 697; ^{1}H NMR (500 MHz, $CDCl_3$, diastereomers) δ 7.44–7.27 (15H, m), 7.25–7.19 (5H, m), 7.19–7.14 (2H, m), 7.13–7.10 (1H, m), 7.07 (1H, d, *J* = 7.6 Hz), 7.02 (1H, d, *J* = 7.5 Hz), 5.00 (1H, d, *J* = 13.6 Hz), 4.95 (1H, d, *J* = 13.6 Hz), 4.87–4.72 (2H, m), 4.67 (1H, dd, *J* = 12.2, 4.4 Hz), 4.64–4.60 (2H, m), 4.57 (1H, dd, *J* = 10.5, 5.3 Hz), 4.45 (0.5H, d, *J* = 7.8 Hz), 4.40 (0.5H, d, *J* = 7.8 Hz), 3.81–3.65 (3H, m), 3.58–3.47 (2H, m), 3.21–3.08 (0.5H, d, *J* = 7.8 Hz), 2.96 (0.5H, dd, *J* = 11.9, 4.4 Hz), 2.71–2.62 (1.5H, m), 2.56–2.42 (4.5H, m), 2.42–2.30 (2H, m), 2.27–2.19 (2.5H, m), 1.86–1.73 (1.5H, m), 1.41–1.29 (4H, m); ^{13}C NMR (126 MHz, $CDCl_3$, diastereomers) δ 142.8, 142.7, 138.7, 138.5, 138.4 (x 2), 138.2, 138.0, 137.8, 137.4, 128.7–128.5(16C), 128.4–128.3(10C), 128.2 (x 2), 128.1 (x 2), 128.0 (x 2), 127.9(7C), 127.8(3C), 127.7, 127.6, 127.5 (x 2), 127.3 (x 2), 125.8, 125.7, 104.6, 102.5, 86.1, 83.7, 79.1 (x 2), 75.4 (x 2), 75.2, 75.1, 75.0, 74.9, 73.7 (x 2), 71.4, 71.1, 69.1 (x 2), 64.4 (x 2), 64.1, 63.9, 49.9, 49.6, 49.2(4C), 33.9, 33.8, 28.8, 28.5, 26.5(4C), 25.2, 25.1; HRMS (ESI$^+$): found MH+ 755.4437, [$C_{49}H_{59}N_2O_5$]$^+$ requires 755.4418.

Methyl (4-Phenyl-2-(piperidin-1-yl)butyl)prolinate (75)

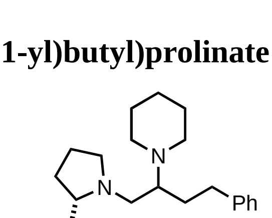

Following **GP3** but using 10 equiv. of NaI, **1** (10 μL, 0.1 mmol, 1.0 equiv.), **10** (15 μL, 0.1 mmol, 1.0 equiv.) and L-Pro-OMe hydrochloride (83 mg, 0.5 mmol, 5.0 equiv.) gave **75** (25 mg, 73%) as an oil. d.r. 1:1. R_f 0.30 [CH_2Cl_2:$MeNO_2$:MeOH (10:1:1)]; FT-IR ν_{max} (film)/cm^{-1}: 2926, 2852, 2803, 1664, 1496; ^{1}H NMR (400 MHz, $CDCl_3$,

diastereomers) δ 7.29–7.23 (2H, m), 7.23–7.11 (3H, m), 3.68 (3H, s), 3.29–3.19 (1H, m), 3.15–3.06 (1H, m), 2.75–2.67 (1.5H, m), 2.67–2.48 (5H, m), 2.47–2.36 (2H, m), 2.13–1.97 (1.5H, m), 1.95–1.80 (3H, m), 1.80–1.70 (3H, m), 1.60–1.47 (4H, m), 1.47–1.36 (2H, m); ^{13}C NMR (126 MHz, $CDCl_3$, diastereomers) δ 175.3, 174.9, 143.3, 143.1, 128.7, 128.6, 128.4, 128.3, 125.7, 125.6, 66.3, 66.2, 63.1, 62.5, 54.9, 54.7, 54.1, 53.4, 51.8, 51.7, 49.7, 49.5, 33.7, 33.4, 31.5, 31.0, 30.0, 29.9, 29.1, 26.8, 26.8, 25.2, 23.6, 23.3; HRMS (ESI$^+$): found MH+ 345.2534, $[C_{21}H_{33}N_2O_2]^+$ requires 345.2537.

***tert*-Butyl-3-(((*1S,4S*)-2-oxa-5-azabicyclo[2.2.1]heptan-5-yl)methyl)-3-(piperidin-1-yl)azetidine-1-carboxylate (76)**

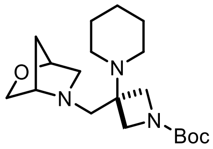

Following **GP3'** but using $AgBF_4$ (1.5 equiv.) in place of NaI, **1** (10 µL, 0.1 mmol, 1.0 equiv.), **10** (15 µL, 0.1 mmol, 1.0 equiv.) and (*1S,4S*)-2-oxa-5-azabicyclo[2.2.1]heptane hydrochloride (68 mg, 0.5 mmol, 5.0 equiv.) gave **76** (17.5 mg, 50%) as an oil. R_f 0.40 [CH_2Cl_2:acetone (7:3)]; FT-IR ν_{max} (film)/cm^{-1} 2933, 1700, 1399, 1366, 1258, 1031; ^{1}H NMR (500 MHz, $CDCl_3$) δ 4.32 (1H, s), 3.91 (1H, d, *J* = 7.6 Hz), 3.68–3.61 (3H, m), 3.57 (2H, d, *J* = 7.5 Hz), 3.38 (1H, s), 2.99 (1H, d, *J* = 9.5 Hz), 2.81 (1H, d, *J* = 13.8 Hz), 2.75 (1H, d, *J* = 13.8 Hz), 2.56 (1H, d, *J* = 9.6 Hz), 2.50 – 2.40 (4H, m), 1.72 (1H, d, *J* = 9.3 Hz), 1.64 (1H, d, *J* = 9.5 Hz), 1.47 (4H, p, *J* = 5.4 Hz), 1.42–1.33 (2H, m), 1.36 (9H, s); ^{13}C NMR (126 MHz, $CDCl_3$ conformers) δ 156.7, 79.2, 77.5, 70.1, 64.1, 63.6, 60.2, 57.1, 56.7, 55.8, 55.2, 55.0, 46.9, 36.4, 28.5, 26.6, 24.8; HRMS (HESI): found MNa$^+$ 374.2400, $C_{19}H_{33}N_3O_3Na$ requires 374.2414.

1,3-Dimethyl-7-(3-((*trans*-2-phenylcyclopropyl)amino)-2-(piperidin-1-yl)propyl)-3,7-dihydro-*1H*-purine-2,6-dione (77)

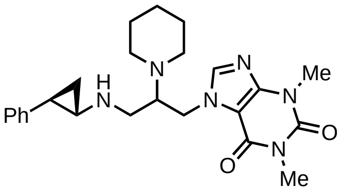

Following **GP3** but warming the reaction to 60 ºC after the addition of NaI (10.0 equiv.) and the second amine, **1** (10 µL, 0.1 mmol, 1.0 equiv.), **S12** (22 mg, 0.1 mmol, 1.0 equiv.) and *trans*-2-phenylcyclopropan-1-amine hydrochloride (75 mg, 0.5 mmol, 5.0

equiv.) gave **77** (17.5 mg, 40%) as an oil. d.r. 1:1. R_f 0.50 [acetone:CH_2Cl_2 (7:3)]; FT-IR ν_{max} (film)/cm^{-1} 2930, 1700, 1650, 1551, 1434, 1259, 1030; ^{1}H NMR (500 MHz, $CDCl_3$) δ 7.58 (1H, br s), 7.27–7.19 (2H, m), 7.16–7.11 (1H, m), 7.07–6.94 (2H, m), 4.47–4.22 (2H, m), 3.59 (1.5H, s), 3.58 (1.5H, s), 3.40 (1.5H, s), 3.38 (1.5H, s), 3.14–3.00 (1H, m), 2.92–2.84 (1H, m), 2.74–2.62 (2H, m), 2.63–2.46 (3H, m), 2.41–2.28 (1H, m), 1.95–1.81 (1H, m), 1.52 (4H, br s), 1.44 (2H, br s), 1.09 (0.5H, dt, J = 9.5, 4.8 Hz), 1.05 (0.5H, dt, J = 9.5, 4.8 Hz), 1.00–0.91 (1H, m); ^{13}C NMR (126 MHz, $CDCl_3$) δ 155.3, 155.2, 151.8, 151.7, 148.9 (x2), 142.4, 142.3, 142.2, 142.1, 128.4, 128.3, 125.9, 125.8, 125.6 (x 2), 106.9 (x 2), 64.3, 64.1, 53.9, 50.0, 49.9, 46.9, 46.7, 45.6 (x 2), 41.8, 41.7, 29.9, 29.8, 29.4, 28.1, 26.8, 25.1, 25.0, 24.8 (x 2), 17.4 (x 2); HRMS (HESI): found MH^+ 437.2641, $C_{24}H_{33}N_6O_2$ requires 437.2660.

Benzyl 4-(1-((*1R,5S*)-8-Oxo-1,5,6,8-tetrahydro-*2H*-1,5-methanopyrido[1,2-a][1,5]diazocin-3(*4H*)-yl)-4-phenylbutan-2-yl)piperazine-1-carboxylate (78)

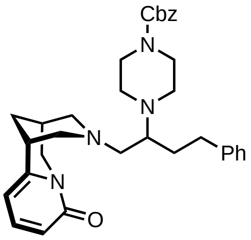

Following **GP3’** but warming the reaction to 60 ºC after the addition of NaI and the second amine, benzyl piperazine-1-carboxylate (19 µL, 0.1 mmol, 1.0 equiv.), **10** (15 µL, 0.1 mmol, 1.0 equiv.) and cytisine (95 mg, 0.5 mmol, 5.0 equiv.) gave **78** (31 mg, 57%) as an oil. d.r. 1:1. R_f 0.50 [CH_2Cl_2:acetone (1:1)]; FT-IR ν_{max} (film)/cm^{-1} 29339, 2805, 2360, 1697, 1651, 1565, 1545, 1495, 1492, 1356, 1309, 1257, 1240, 1139, 1058, 1026; ^{1}H NMR (500 MHz, $CDCl_3$, diasteromers) δ 7.43–7.26 (5H, m), 7.23 (2H, t, J = 8.4 Hz), 7.19–7.00 (4H, m), 6.34 (0.5H, d, J = 9.0 Hz), 6.30 (0.5H, d, J = 9.0 Hz), 5.90 (0.5H, d, J = 7.6 Hz), 5.89 (0.5H, d, J = 7.5 Hz), 5.13 (1H, s), 5.12 (1H, s), 4.06–3.93 (1H, m), 3.91–3.77 (1H, m), 3.52–3.15 (4H, m), 2.96–2.70 (3H, m), 2.60–2.45 (2H, m), 2.45–2.31 (4H, m), 2.32–2.21 (3H, m), 2.21–2.11 (2H, m), 2.05–1.94 (1H, m), 1.85 (1H, d, J = 12.9 Hz), 1.73 (1H, d, J = 12.9 Hz), 1.63–1.45 (1H, m), 1.45–1.32 (1H, m); ^{13}C NMR (126 MHz, $CDCl_3$, diasteromers) δ 163.5, 163.4, 155.3, 155.2, 151.4, 151.3, 142.5, 142.4, 138.6, 138.4, 137.0, 136.9, 128.6, 128.6, 128.5, 128.4, 128.3, 128.2, 128.1, 128.0, 128.0, 127.9, 127.8, 125.7, 125.6, 116.7, 116.6, 116.5, 104.5, 104.4, 67.1, 67.0, 61.3, 61.2, 61.0, 60.1, 60.0, 58.4, 58.0, 50.1, 50.0, 49.2, 48.4, 48.1, 47.4, 44.6,

35.7, 32.8, 32.7, 32.0, 31.9, 28.2, 28.1, 26.0; HRMS (HESI): found MNa^+ 563.2976, $C_{33}H_{40}N_4O_3Na$ requires 563.2993.

7 Aminohydroxylation and Diamination of Styrenes

7.1 Aminohydroxylation – Substrate Scope

General Procedure for the Aminohydroxylation of Styrenes – GP4

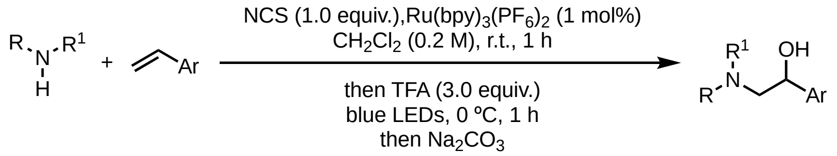

A tube equipped with a stirring bar was charged with NCS (1.0 equiv.) and $Ru(bpy)_3(PF_6)_2$ (1 mol%). The tube was capped with a Supelco aluminium crimp seal with septum (PTFE/butyl), evacuated and refilled with N_2 (x 3). The amine (1.0 equiv.) and CH_2Cl_2 (0.1 M, dry and degassed by bubbling through with N_2 for 20 min) were added and the mixture was stirred for 1 h in the dark. The mixture was cooled to 0 ºC then the styrene (1.0 equiv.) and TFA (3.0 equiv.) were added, and the blue LEDs were immediately switched on. The mixture was stirred under irradiation for 1 h at 0 ºC. Na_2CO_3 (10.0 equiv.) and H_2O (1 mL) were added and the resulting heterogeneous micture was stirred vigorously for 30 minutes. The layers were separated and the aqueous layer was extracted with CH_2Cl_2 (x 2). The combined organic layers were dried ($MgSO_4$), filtered and evaporated. Purification by preparative TLC chromatography on silica gave the products.

1-Phenyl-2-(piperidin-1-yl)ethan-1-ol (55)

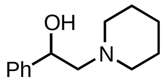

Following **GP4**, **1** (10 μL, 0.1 mmol, 1.0 equiv.) and **53** (13 μL, 0.1 mmol, 1.0 equiv.) gave **55** (13 mg, 65%) as an oil. ^{1}H NMR (400 MHz, $CDCl_3$) δ 7.37–7.24 (5H, m), 4.69 (1H, dd, J = 10.6 Hz, 3.5 Hz), 2.66 (2H, br s), 2.45 (1H, dd, J = 12.4 Hz, 3.6 Hz), 2.40–2.34 (3H, m), 1.67–1.45 (6H, m); ^{13}C NMR (101 MHz, $CDCl_3$) δ 142.6, 128.4, 127.5,126.0, 68.9, 67.2, 54.6, 26.3, 24.4. Data in accordance with the literature.[12]

2-(pyrrolidin-1-yl)-1-(4-(trifluoromethyl)phenyl)ethan-1-ol (56)

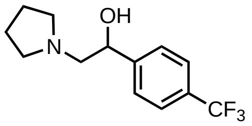

Following **GP4**, pyrrolidine (8.5 μL, 0.1 mmol, 1.0 equiv.) and 1-(trifluoromethyl)-4-vinylbenzene (15 μL, 0.1 mmol, 1.0 equiv.) gave **56** (13 mg, 51%) as an oil. R_f 0.50

[acetone:CH_2Cl_2 (8:2)]; FT-IR ν_{max} (film)/cm^{-1} 3500, 2961, 1619, 1412, 1324, 1259, 1164, 1066, 1019; ^{1}H NMR (400 MHz, $CDCl_3$) δ 7.60 (2H, d, *J* = 8.1 Hz), 7.50 (2H, d, *J* = 8.1 Hz), 4.75 (1H, dd, *J* = 10.6, 3.5 Hz), 2.82–2.73 (2H, m), 2.73 (1H, dd, *J* = 12.0, 10.7 Hz), 2.59–2.52 (2H, m), 2.52 (1H, dd, *J* = 12.1, 3.5 Hz), 1.91–1.72 (4H, m); ^{13}C NMR (101 MHz, $CDCl_3$) δ 146.7, 129.7 (q, *J* = 32.3 Hz), 126.2, 125.4 (q, *J* = 3.8 Hz), 124.4 (q, *J* = 272.0 Hz), 70.2, 63.9, 54.0, 23.8; ^{19}F NMR (376 MHz, $CDCl_3$) δ –62.4; HRMS (HESI): Found MH$^+$ 260.1267 $C_{13}H_{17}NOF_3$ requires 260.1257.

2-(Azepan-1-yl)-1-(3-bromophenyl)ethan-1-ol (57)

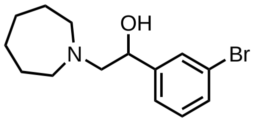

Following **GP4**, azepane (11.5 μL, 0.1 mmol, 1.0 equiv.) and 1-bromo-3-vinylbenzene (13 μL, 0.1 mmol, 1.0 equiv.) gave **57** (24 mg, 82%) as an oil. R_f 0.50 [Et_2O:CH_2Cl_2 (6:4)]; FT-IR ν_{max} (film)/cm^{-1} 3400, 2924, 2853, 1595, 1568, 1471, 1426, 1400, 1323, 1258, 1195, 1066; ^{1}H NMR (400 MHz, $CDCl_3$) δ 7.54 (1H, s), 7.38 (1H, d, *J* = 7.8 Hz), 7.28 (1H, d, *J* = 7.8 Hz), 7.19 (1H, t, *J* = 7.8 Hz), 4.57 (1H, dd, *J* = 10.6, 3.6 Hz), 4.39 (1H, br s), 2.88–2.78 (2H, m), 2.78 (1H, dd, *J* = 12.6, 3.6 Hz), 2.67 (1H, dd, *J* = 13.1, 7.2 Hz), 2.72–2.63 (1H, m), 2.37 (1H, dd, *J* = 12.5, 10.5 Hz, 1H), 1.79–1.55 (8H, m); ^{13}C NMR (126 MHz, $CDCl_3$) δ 145.1, 130.5, 130.0, 129.0, 124.6, 122.6, 68.9, 66.2, 55.7, 28.7, 27.1; HRMS (ASAP): Found MH$^+$ 298.0809 $C_{14}H_{21}NOBr$ requires 298.0801.

2-(Diisopropylamino)-1-phenylethan-1-ol (58)

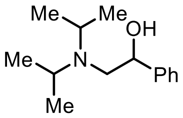

Following **GP4**, *i*-$PrNH_2$ (14 μL, 0.1 mmol, 1.0 equiv.) and **53** (13 μL, 0.1 mmol, 1.0 equiv.) gave **58** (5 mg, 22%) as an oil. ^{1}H NMR (400 MHz, $CDCl_3$) δ 7.40–7.23 (5H, m), 4.56 (1H, dd, *J* = 10.5, 3.9 Hz), 3.15 (2H, hept, *J* = 6.6 Hz), 2.74 (1H, dd, *J* = 13.4, 3.9 Hz), 2.36 (1H, dd, *J* = 13.4, 10.6 Hz), 1.14 (6H, d, *J* = 6.7 Hz), 1.03 (6H, d, *J* = 6.6 Hz). Data in accordance with the literature.[13]

7.2 Diamination – Substrate Scope

General Procedure for the Diamination of Styrenes – GP5

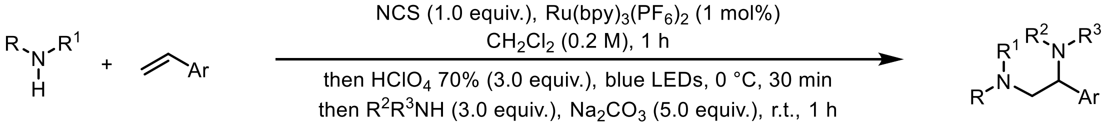

A tube equipped with a stirring bar was charged with NCS (1.0 equiv.) and Ru(bpy)$_3$(PF$_6$)$_2$ (1 mol%). The tube was capped with a Supelco aluminium crimp seal with septum (PTFE/butyl), evacuated and refilled with N_2 (x 3). The amine (1.0 equiv.) and CH_2Cl_2 (0.1 M, dry and degassed by bubbling through with N_2 for 20 min) were added and the mixture was stirred for 1 h in the dark. The mixture was cooled to 0 ºC then the styrene (1.0 equiv.) and $HClO_4$ 70% (3.0 equiv.) were added, and the blue LEDs were immediately switched on. The mixture wans stirred under irradiation for 1 h at 0 ºC. The second amine (3.0 equiv.) was added, followed by Na_2CO_3 (5.0 equiv.). The mixture was stirred for 1 h at room temperature, then H_2O (2 mL) was added and the mixture was shaken vigorously. The layers were separated and the aqueous layer was extracted with CH_2Cl_2 (x 2). The combined organic layers were dried ($MgSO_4$), filtered and evaporated. Purification by preparative TLC chromatography on silica gave the products.

4-(1-Phenyl-2-(piperidin-1-yl)ethyl)morpholine (79)

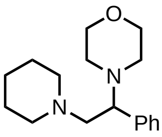

Following **GP5**, **1** (9 µL, 0.1 mmol, 1.0 equiv.), **53** (13 µL, 0.1 mmol, 1.0 equiv.) and morpholine (26 µL, 0.3 mmol, 3.0 equiv.) gave **79** (23 mg, 83%) as an oil. R$_f$ 0.50 [CH_2Cl_2:acetone (6:4)]; ^{1}H NMR (500 MHz, $CDCl_3$) δ 7.33–7.26 (2H, m), 7.26–7.19 (3H, m), 3.65 (4H, t, J = 4.7 Hz), 3.52 (1H, t, J = 6.1 Hz), 2.87 (1H, dd, J = 13.1, 6.4 Hz), 2.57 (1H, dd, J = 13.1, 5.7 Hz), 2.61–2.51 (2H, m), 2.47–2.33 (6H, m), 1.49 (4H, p, J = 5.6 Hz), 141–1.32 (2H, m). Data in accordance with literature.[14]

N,N-Diethyl-1-phenyl-2-(piperidin-1-yl)ethan-1-amine (80)

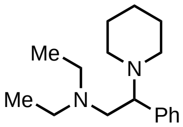

Following **GP5**, Et_2NH (10.5 µL, 0.1 mmol, 1.0 equiv.), **53** (13 µL, 0.1 mmol, 1.0 equiv.) and **1** (30 µL, 0.3 mmol, 3.0 equiv.) gave **80** (8.5 mg, 33%) as an oil. ^{1}H NMR (500 MHz, $CDCl_3$) δ 7.15–7.55 (5H, m), 3.54 (1H, t, J = 6.4 Hz), 2.96 (1H, dd, J = 13.1, 5.4 Hz), 2.82 (1H, dd, J = 13.1, 5.5 Hz), 2.46–2.70 (4H, m), 2.39 (4H, br s), 1.42–1.75 (4H, m), 1.22–1.42(2H, m), 0.95 (6H, t, J = 7.1 Hz). Data in accordance with literature.[15]

Benzyl 4-(1-Phenyl-2-(piperidin-1-yl)ethyl)piperazine-1-carboxylate (81)

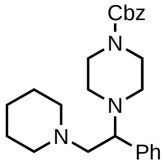

Following **GP5**, **1** (9 µL, 0.1 mmol, 1.0 equiv.), **53** (13 µL, 0.1 mmol, 1.0 equiv.) and 1-Cbz-piperazine (58 µL, 0.3 mmol, 3.0 equiv.) gave **81** (29 mg, 72%) as an oil. R_f 0.50 [CH_2Cl_2:acetone (6:4)]; FT-IR ν_{max} (film)/cm^{-1} 2341, 2256, 1635, 1463, 1374, 1258, 1037, 920; ^{1}H NMR (500 MHz, $CDCl_3$,) δ 7.39–7.24 (7H, m), 7.24–7.09 (3H, m), 5.05 (2H, s), 3.58 (1H, t, J = 6.1 Hz), 3.50–3.38 (4H, m), 2.82 (1H, dd, J = 13.2, 6.6 Hz), 2.56 (1H, dd, J = 13.2, 5.5 Hz), 2.51–2.43 (2H, m), 2.36 (6H, br s), 1.47 (4H, p, J = 5.6 Hz), 1.39–1.29 (2H, m); ^{13}C NMR (126 MHz, $CDCl_3$) δ 155.3, 140.3, 136.9, 128.6, 128.5, 128.2, 128.0, 127.9, 127.2, 67.2, 67.1, 62.6, 55.2, 50.4, 44.3, 26.1, 24.4; HRMS (ASAP): Found MH$^+$ 408.2636 $C_{25}H_{34}N_3O_2$ requires 408.2646.

1-Phenyl-2-(piperidin-1-yl)-N-((tetrahydrofuran-3-yl)methyl)ethan-1-amine (82)

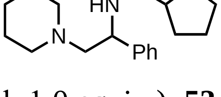

Following **GP5**, **1** (9 µL, 0.1 mmol, 1.0 equiv.), **53** (13 µL, 0.1 mmol, 1.0 equiv.) and 3-(aminomethyl)tetrahydrofuran (30 µL, 0.3 mmol, 3.0 equiv.) gave **82** (22 mg, 76%) as an oil. d.r. 1:1. R_f 0.50 [CH_2Cl_2:acetone (6:4)]; FT-IR ν_{max} (film)/cm^{-1} 2934, 2359, 1504, 1260, 1242, 1197, 1163, 1096, 1040; ^{1}H NMR (500 MHz, $CDCl_3$) δ 7.39–7.27 (4H, m), 7.23 (1H, t, J = 7.4 Hz), 3.88 (1H, dt, J = 15.9, 7.8 Hz), 3.84–3.74 (1H, m), 3.77–3.66 (2H, m), 3.48–3.40 (1H, m), 2.54 (2H, br s), 2.50–2.43 (2H, m), 2.40 (2H,

td), 2.33–2.18 (3H, m), 2.02 (1H, tdd, J = 13.3, 7.6, 5.5 Hz), 1.68–1.51 (5H, m), 1.50–1.32 (2H, m); ^{13}C NMR (126 MHz, $CDCl_3$, diastereomers) δ 143.2 (x 2), 128.3 (x 2), 127.3 (x 2), 127.1 (x 2), 72.3, 72.0, 67.9, 67.8, 66.5 (x 2), 60.3 (x 2), 54.6 (x 2), 51.7, 51.4, 39.6 (x 2), 30.8 (x 2), 26.2 (x 2), 24.5 (x 2); HRMS (ASAP): Found MH^+ 289.2268 $C_{18}H_{29}N_2O$ requires 289.2274.

1-(1-Phenyl-2-(piperidin-1-yl)ethyl)pyridin-1-ium perchlorate (83)

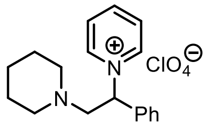

Following **GP5** but letting the reaction stir overnight upon addition of the second amine, **1** (9 µL, 0.1 mmol, 1.0 equiv.), **53** (13 µL, 0.1 mmol, 1.0 equiv.) and pyridine (24 µL, 0.3 mmol) gave **83** (13 mg, 50%) as a solid. R_f 0.51 [CH_2Cl_2:$MeNO_2$:MeOH (20:1:1)]; FT-IR ν_{max} (film)/cm^{-1} 2934, 2852, 1710, 1092; ^{1}H NMR (500 MHz, $CDCl_3$,) δ 8.90 (2H, d, J = 5.7 Hz), 8.42 (1H, t, J = 7.7 Hz), 7.99 (2H, t, J = 7.1 Hz), 7.57 (2H, dd, J = 6.5, 2.9 Hz), 7.47 (3H, dd, J = 5.1, 1.9 Hz), 6.06 (1H, dd, J = 11.3, 3.4 Hz), 3.33–3.22 (1H, m), 3.14 (1H, dd, J = 14.2, 3.3 Hz), 2.82–2.68 (2H, m), 2.27 (2H, dt, J = 10.7, 4.4 Hz), 1.55–1.34 (6H, m) ^{13}C NMR (126 MHz, $CDCl_3$) δ 145.6, 144.2, 133.5, 130.7, 130.0, 128.9, 127.8, 72.6, 61.5, 54.7, 26.1, 24.0; HRMS (ESI$^+$): found M+ 267.1844, $[C_{18}H_{23}N_2]^+$ requires 267.1856.

1-(4-Methoxyphenyl)-*N*,*N*-dimethyl-2-(piperidin-1-yl)ethan-1-amine (84)

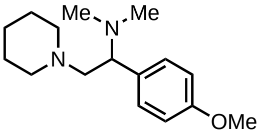

Following **GP5**, **1** (9 µL, 0.1 mmol, 1.0 equiv.) and 1-methoxy-4-vinylbenzene (13 µL, 0.1 mmol, 1.0 equiv.) and Et_2NH (38 µL, 0.3 mmol, 40% wt solution in H_2O) gave **84** (16 mg, 60%) as an oil. R_f 0.50 [CH_2Cl_2:acetone (6:4)]; FT-IR ν_{max} (film)/cm^{-1} 1610, 1465, 1456, 1245, 1038; ^{1}H NMR (500 MHz, $CDCl_3$) δ 7.13 (2H, d, J = 8.6 Hz), 6.85 (2H, d, J = 8.7 Hz), 3.80 (3H, s), 3.49 (1H, t, J = 6.3 Hz), 2.85 (1H, dd, J = 13.0, 6.3 Hz), 2.56 (1H, dd, J = 13.0, 6.3 Hz), 2.37 (4H, br s), 2.17 (6H, s), 1.52-1.47 (4H, m), 1.36 (2H, m); ^{13}C NMR (126 MHz, $CDCl_3$) δ 158.6, 131.8, 129.8, 113.3, 66.8, 62.7, 55.3, 55.3, 42.6, 26.1, 24.5; HRMS (HESI): Found MH^+ 263.2212 $[C_{16}H_{27}N_2O]^+$ requires 263.2210.

2-((1-(4-Methoxyphenyl)-2-(piperidin-1-yl)ethyl)(methyl)amino)ethan-1-ol (85)

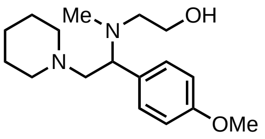

Following **GP5**, **1** (9 μL, 0.1 mmol, 1.0 equiv.), 1-methoxy-4-vinylbenzene (13 μL, 0.1 mmol, 1.0 equiv.) and 2-(methylamino)ethanol (30.5 μL, 0.3 mmol, 3.0 equiv.) gave **85** (15 mg, 51%) as an oil. R_f 0.40 [CH_2Cl_2:acetone (6:4)]; FT-IR ν_{max} (film)/cm^{-1} 3345, 1498, 1442, 1440, 1258, 1025, 862; ^{1}H NMR (500 MHz, $CDCl_3$) δ 7.13 (2H, d, *J* = 8.6 Hz), 6.86 (2H, d, *J* = 8.6 Hz), 3.87 (1H, dd, *J* = 11.9, 4.6 Hz), 3.80 (3H, s), 3.71–3.60 (1H, m), 3.48 (1H, dt, *J* = 10.7, 3.0 Hz), 3.14–2.94 (2H, m), 2.63 (2H, br s), 2.35 (2H, br s), 2.29–2.18 (2H, m), 2.24 (3H, s), 1.71–1.57 (4H, m), 1.51–1.40 (2H, m); ^{13}C NMR (126 MHz, $CDCl_3$) δ 159.0, 130.7, 129.3, 113.7, 62.7, 60.6, 60.0, 55.3, 54.1, 52.4, 40.4, 25.7, 24.5; HRMS (HESI): Found MH$^+$ 293.2212 $C_{17}H_{29}N_2O_2$ requires 293.2224.

***N,N*-dimethyl-2-(piperidin-1-yl)-1-(4-(trifluoromethyl)phenyl)ethan-1-amine (86)**

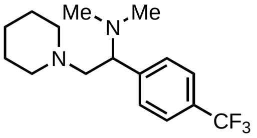

Following **GP5**, **1** (10 μL, 0.1 mmol, 1.0 equiv.), 1-(trifluoromethyl)-4-vinylbenzene (15 μL, 0.1 mmol) and Me_2NH (38 μL, 0.3 mmol, 3.0 equiv., 40% wt solution in H_2O) gave **86** (17 mg, 57%) as an oil. R_f 0.50 [CH_2Cl_2:acetone (6:4)]; FT-IR ν_{max} (film)/cm^{-1} 2359, 2341, 1325, 1259, 1032, 860; ^{1}H NMR (500 MHz, $CDCl_3$) δ 7.57 (2H, d, *J* = 8.1 Hz), 7.35 (2H, d, *J* = 8.0 Hz), 3.55 (1H, t, *J* = 6.4 Hz), 2.84 (1H, dd, *J* = 13.0, 6.1 Hz), 2.57 (1H, dd, *J* = 13.0, 6.8 Hz), 2.46–2.30 (4H, m), 2.19 (6H, s), 1.48 (4H, p, *J* = 5.5 Hz), 1.42–1.31 (2H, m); ^{13}C NMR (126 MHz, $CDCl_3$) δ 144.5, 129.2 (q, *J* = 32.4 Hz), 128.8, 124.9 (q, *J* = 3.9 Hz), 124.4 (q, *J* = 271.9 Hz), 67.4, 62.2, 55.3, 42.9, 26.0, 24.4; ^{19}F NMR (376 MHz, $CDCl_3$) δ –62.3; HRMS (ASAP): Found MH$^+$ 301.1881 $C_{16}H_{24}N_2F_3$ requires 301.1886.

5,6-Dimethoxy-2-((1-(2-(piperidin-1-yl)-1-(2-(trifluoromethyl)phenyl)ethyl) piperidin-4-yl)methyl)-2,3-dihydro-*1H*-inden-1-one (87)

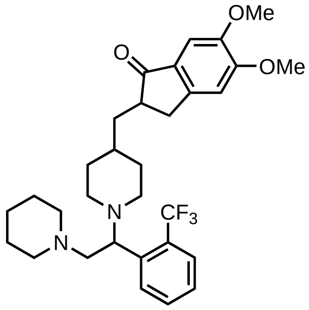

Following **GP5**, **1** (10 μL, 0.1 mmol, 1.0 equiv.), 1-(trifluoromethyl)-2-vinylbenzene (15 μL, 0.1 mmol, 1.0 equiv.) and desbenzyl donepezil hydrochloride (98 mg, 0.3 mmol, 3.0 equiv.) gave **87** (41 mg, 76%) as an oil. dr 1:1. R_f 0.50 [CH_2Cl_2:acetone (6:4)]; FT-IR ν_{max} (film)/cm^{-1} 2923, 2850, 2359, 2341, 1698, 1591, 1500, 1456, 1361, 1311, 1260, 1223, 1154, 1118, 1034; ^{1}H NMR (500 MHz, $CDCl_3$) δ 7.74 (1H, d, *J* = 7.9 Hz), 7.59 (1H, d, *J* = 7.9 Hz), 7.49 (1H, t, *J* = 7.6 Hz), 7.30 (1H, t, *J* = 7.6 Hz), 7.16 (1H, s), 6.84 (1H, d, *J* = 1.6 Hz), 3.95 (3H, s), 3.90 (3H, s), 3.87 (1H br s), 3.51 (1H, t, *J* = 11.7 Hz), 3.21 (1H, dd, *J* = 17.3, 7.8 Hz), 2.74 (1H, dd, *J* = 13.9, 8.0 Hz), 2.73–2.61 (2H, m), 2.56 (1H, t, *J* = 11.6 Hz), 2.40 (2H, br s), 2.31 (1H, dd, *J* = 13.5, 3.8 Hz), 2.25 (2H, br s), 2.12 (1H, tt, *J* = 12.1, 2.9 Hz), 1.98 (1H, t, *J* = 11.4 Hz), 1.94–1.74 (2H, m), 1.72–1.54 (1H, m), 1.52–1.41 (6H, m), 1.39–1.28 (4H, m); ^{13}C NMR (126 MHz, $CDCl_3$, diastereomers) δ 208.0, 207.9, 155.4 (x 2), 149.4 (x 2), 148.8 (x 2), 143.5 (x 2), 131.6 (x 2), 129.3 (x 2), 128.2 (x 2, q, *J* = 29.4 Hz), 126.4 (x 2), 125.31(2C, q, *J* = 5.6 Hz), 124.6(2C, q, *J* = 274.0 Hz), 107.3 (x 2), 104.4 (x 2), 64.8 (x 2), 61.6 (x 2), 56.2 (x 2), 56.1 (x 2), 55.0 (x 2), 52.7 (x 2), 51.7 (x 2), 45.5 (x 2), 38.7 (x 2), 34.8 (x 2), 33.7, 33.6, 33.2 (x 2), 32.3, 32.1, 26.1 (x 2), 24.4 (x 2); ^{19}F NMR (376 MHz, $CDCl_3$, diastereomers) δ –57.2, –57.25; HRMS (HESI): Found MH^+ 545.2983 $C_{31}H_{40}N_2O_3F_3$ requires 545.2986.

1-(1-(3-Bromophenyl)-2-(piperidin-1-yl)ethyl)-4-(4-(trifluoromethoxy)phenoxy) piperidine (88)

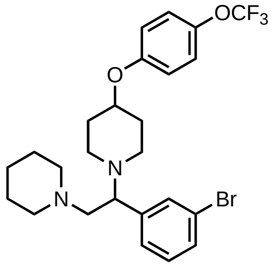

Following **GP5**, **1** (10 μL, 0.1 mmol, 1.0 equiv.), 1-bromo-3-vinylbenzene (13 μL, 0.1 mmol, 1.0 equiv.) and 4-[4-(trifluoromethoxy)phenoxy]piperidine (78 mg, 0.3 mmol, 3.0 equiv.) gave **88** (49 mg, 94%) as an oil. R_f 0.50 [CH_2Cl_2:acetone (6:4)]; FT-IR ν_{max} (film)/cm^{-1} 2934, 2359, 1504, 1260, 1242, 1197, 1163, 1096, 1040; ^{1}H NMR (500 MHz, $CDCl_3$) δ 7.42 (1H, s), 7.37 (1H, dt, *J* = 6.0, 2.4 Hz), 7.22–7.16 (2H, m), 7.09 (2H, d, *J* = 8.7 Hz), 6.83 (2H, d, *J* = 9.1 Hz), 4.15 (1H, tt, *J* = 8.2, 3.9 Hz), 3.61 (1H, t, *J* = 6.2 Hz), 2.79 (1H, dd, *J* = 13.2, 6.2 Hz), 2.88–2.69 (2H, m), 2.57 (1H, dd, *J* = 13.1, 6.3 Hz), 2.49–2.32 (4H, m), 2.31–2.21 (2H, m), 2.05–1.86 (2H, m), 1.75 (2H, ddp, *J* = 13.2, 8.8, 4.9, 4.3 Hz), 1.56–1.42 (4H, m), 1.42–1.34 (2H, m); ^{13}C NMR (126 MHz, $CDCl_3$) δ 156.1, 143.4, 142.7, 131.4, 130.0, 129.6, 127.1, 122.5, 122.4, 120.69 (q, *J* = 256.1 Hz), 116.9, 74.0, 66.6, 62.1, 55.3, 48.2, 47.4, 31.4, 31.3, 26.2, 24.5; ^{19}F NMR (376 MHz, $CDCl_3$) δ –58.4; HRMS (HESI): Found MH^+ 527.1513 $C_{25}H_{31}N_2O_2BrF_3$ requires 527.1521.

Methyl-4-(1-(4-(4-chlorophenyl)-4-hydroxypiperidin-1-yl)-2-(pyrrolidin-1-yl)ethyl) benzoate (89)

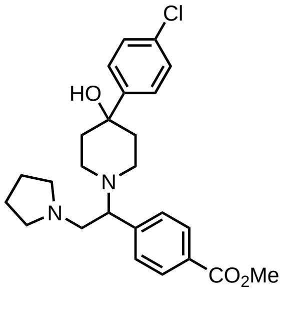

Following **GP5**, pyrrolidine (10 μL, 0.1 mmol, 1.0 equiv.), methyl 4-vinylbenzoate (16 mg, 0.1 mmol, 1.0 equiv.) and 4-(4-chlorophenyl)piperidin-4-ol (63 mg, 0.3 mmol, 3.0 equiv.) gave **89** (25 mg, 57%) as an oil. R_f 0.50 [CH_2Cl_2:acetone (1:1)]; FT-IR ν_{max} (film)/cm^{-1} 3381, 2952, 2821, 2358, 1720, 1609, 1489, 1435, 1387, 1280, 1185, 1109, 1042, 1012; ^{1}H NMR (500 MHz, $CDCl_3$ and MeOH) δ 7.90 (2H, d, *J* = 8.1 Hz), 7.37–

7.25 (4H, m), 7.16 (2H, d, *J* = 8.5 Hz), 3.81 (3H, s), 3.68–3.62 (1H, m), 3.01 (1H, dd, *J* = 12.4, 5.3 Hz), 2.87 (1H, dd, *J* = 12.5, 7.7 Hz), 2.80–2.72 (1H, m), 2.59–2.50 (1H, m), 2.50–2.40 (2H, m), 2.40–2.31 (2H, m), 2.31–2.16 (2H, m), 2.00 (1H, td, *J* = 12.8, 4.5 Hz), 1.89 (1H, td, *J* = 12.8, 4.4 Hz), 1.68–1.53 (4H, m), 1.58–1.50 (2H, m); ^{13}C NMR (126 MHz, $CDCl_3$ and MeOH) δ 167.3, 147.2, 144.0, 132.2, 129.2, 128.9, 128.8, 128.0, 126.0, 70.4, 68.6, 58.5, 54.9, 52.0, 47.9, 44.5, 38.1, 38.0, 23.0; HRMS (APCI): Found MH^+ 443.2089 $C_{25}H_{32}N_2O_3Cl$ requires 443.2096.

8 Olefin Aziridination

8.1 Substrate Scope

General Procedure for the Olefin Aziridination – GP6

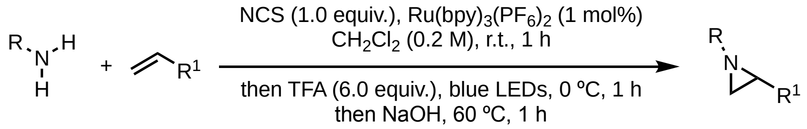

A dry tube equipped with a stirring bar was charged with NCS (13 mg, 0.1 mmol, 1.0 equiv.) and $Ru(bpy)_3(PF_6)_2$ (0.7 mg, 0.01 mmol, 1 mol%). The tube was capped with a Supelco aluminium crimp seal with septum (PTFE/butyl), evacuated and refilled with N_2 (x 3). CH_2Cl_2 (0.5 mL) (dry and degassed by bubbling through with N_2 for 20 min) and the amine (0.1 mmol, 1.2 equiv.), were added and the mixture was stirred for 1 h in the dark. The mixture was cooled to 0 ºC and then a solution of the olefin (0.1 mmol, 1.0 equiv.) in CH_2Cl_2 (0.5 mL) and TFA (46 μL, 0.6 mmol, 6.0 equiv.) were added. The blue LEDs were immediately switched on and the mixture was stirred under irradiation at 0 ºC for 1 h. NaOH (1.0 M in MeOH) was added and the mixture was stirred for 1 h at 60 ºC. The mixture was allowed to cool to room temperature, diluted with H_2O (10 mL) and the layers were separated. The aqueous layer was extracted with CH_2Cl_2 (3 x 10 mL). The combined organic layers were dried ($MgSO_4$), filtered and evaporated. Purification by flash column chromatography or preparative TLC chromatography on silica gel gave the products.

1-Cyclohexyl-2-phenethylaziridine (90)

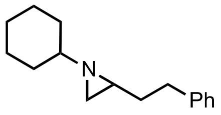

Following **GP6** but adding adding KPF_6 (18 mg, 0.1 mmol, 1.0 equiv.) to the reaction mixture, cyclohexylamine (14 μL, 0.12 mmol, 1.2 equiv.) and **10** (15 μL, 0.1 mmol, 1.0 equiv.) gave **90** (11.5 mg, 50%) as an oil. R_f 0.50 [petrol:EtOAc (8:2)]; FT-IR ν_{max} (film)/cm^{-1} 2362, 1259, 1029, 861; ^{1}H NMR (500 MHz, $CDCl_3$) δ 7.27 (2H, t, *J* = 7.5 Hz), 7.24–7.13 (3H, m), 2.83 (1H, ddd, *J* = 13.8, 10.2, 5.8 Hz), 2.70 (1H, ddd, *J* = 13.8, 10.1, 6.0 Hz), 1.90–1.77 (3H, m), 1.81–1.70 (2H, m), 1.66–1.52 (2H, m), 1.51 (1H, d, *J* = 3.5 Hz), 1.45–1.28 (3H, m), 1.25 (1H, d, *J* = 6.3 Hz), 1.21–1.10 (3H, m), 1.03 (1H, tt, *J* = 10.7, 3.9 Hz); ^{13}C NMR (126 MHz, $CDCl_3$) δ ^{13}C NMR (126 MHz, $CDCl_3$) δ

142.2, 128.5, 128.4, 125.9, 69.1, 38.2, 35.3, 34.3, 33.3, 32.7, 26.3, 25.2; HRMS (HESI): Found MH$^+$ 230.1893 $C_{16}H_{24}N$ requires 230.1903.

1-(*tert*-Butyl)-2-phenethylaziridine (91)

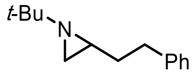

Following **GP6,** *tert*-butylamine (13 µL, 0.12 mmol, 1.2 equiv.) and **10** (15 µL, 0.1 mmol) gave **91** (11 mg, 56%) as an oil. R$_f$ 0.50 [petrol:EtOAc (8:2)]; FT-IR ν_{max} (film)/cm^{-1} 2360, 2341, 2156, 1260, 1089, 1030; ^{1}H NMR (500 MHz, $CDCl_3$) δ 7.32–7.23 (2H, m), 7.23–7.14 (3H, m), 2.89–2.76 (1H, m), 2.74–2.61 (1H, m), 1.86–1.73 (1H, m), 1.70–1.54 (2H, m), 1.48 (1H, d, *J* = 5.7 Hz), 1.29 (1H, s), 0.98 (9H, s); ^{13}C NMR (126 MHz, $CDCl_3$) δ 142.3, 128.5, 128.4, 125.8, 52.6, 35.6, 34.4, 32.0, 26.9, 26.8; HRMS (HESI): Found MH$^+$ 204.1741 $C_{14}H_{22}N$ requires 204.1747.

1-(Adamantan-1-yl)-2-phenethylaziridine (92)

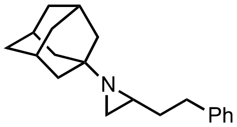

Following **GP6**, 1-adamantylamine (12 mg, 0.12 mmol, 1.2 equiv.) and **10** (15 µL, 0.1 mmol, 1.0 equiv.) gave **92** (24 mg, 87%) as an oil. R$_f$ 0.50 [petrol:EtOAc (8:2)]; FT-IR ν_{max} (film)/cm^{-1} 2851, 2360, 2342, 1258, 1088, 1027; ^{1}H NMR (500 MHz, $CDCl_3$) δ 7.28 (2H, t, *J* = 7.4 Hz), 7.25–7.13 (3H, m), 2.86–2.79 (1H, m), 2.72–2.64 (1H, m), 2.07 (3H, br s), 1.86–1.73 (2H, m), 1.74–1.63 (4H, m), 1.62–1.56 (4H, m), 1.54 (7H, s); ^{13}C NMR (126 MHz, $CDCl_3$) δ 142.3, 128.5, 128.4, 125.8, 52.2, 40.7, 36.9, 35.8, 34.4, 29.9, 29.6, 24.9; HRMS (HESI): Found MH$^+$ 282.2207 $C_{20}H_{28}N$ requires 282.2216.

***cis*-7-phenethyl-7-azabicyclo[4.1.0]heptane (93)**

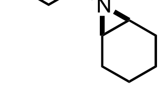

Following **GP6**, 2-phenylethan-1-amine (15 µL, 0.12 mmol, 1.2 equiv.) and cyclohexene (10 µL, 0.10 mmol, 1.0 equiv.) and **10** (15 µL, 0.1 mmol) gave **93** (8 mg, 40%) as an oil. R$_f$ 0.60 [petrol:EtOAc (8:2)]; FT-IR ν_{max} (film)/cm^{-1} 2359, 2341, 2257, 1635, 1373, 1260, 1035; ^{1}H NMR (500 MHz, $CDCl_3$) δ 7.33–7.22 (2H, m), 7.22–7.16

(3H, m), 2.87 (2H, t, *J* = 7.8 Hz), 2.47 (2H, t, *J* = 7.9 Hz), 1.83–1.66 (2H, m), 1.74–1.66 (2H, m), 1.43 (2H, br s), 1.38–1.29 (2H, m), 1.21–1.09 (2H, m); ^{13}C NMR (126 MHz, $CDCl_3$) δ 140.5, 129.0, 128.4, 126.1, 63.3, 38.5, 36.6, 24.6, 20.7; HRMS (HESI): Found MH^+ 202.1583 $C_{14}H_{20}N$ requires 202.1590. Data in accordance with the literature.[16]

9 Diversification of β-Chloroamines

9.1 Substrate Scope

General Procedure for the Derivatization of β-Chloroamines – GP7

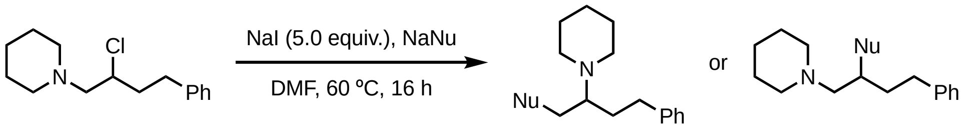

A tube equipped with a stirring bar was charged with NaI (5.0 equiv.), and the appropriate nucleophile as the sodium salt (5.0 equiv). The tube was capped with a Supelco aluminium crimp seal with septum (PTFE/butyl), evacuated and refilled with N_2 (x 3). A solution of **11** (1.0 equiv.) in DMF (0.25 M) was added and the mixture was stirred at 60 °C for 16 h. After cooling the reaction at room temperature, H_2O was added and the layer was separated. The aqueous layer was extracted with CH_2Cl_2 (x 3). The combined organic layers were dried ($MgSO_4$), filtered and evaporated. Purification by flash column chromatography on silica gel gave the products.

1-(1-Methoxy-4-phenylbutan-2-yl)piperidine (94) and 1-(2-methoxy-4-phenylbutyl)piperidine (94')

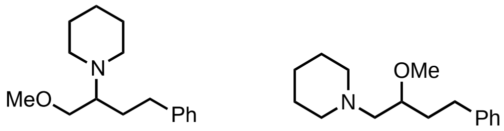

Following **GP7**, **11** (25 mg, 0.1 mmol) and NaOMe (solution 24% in MeOH, 118 μL, 0.5 mmol, 5.0 equiv.) gave **94** and **94'** (15 mg, 59%) as an oil. **94**:**94'** = 3:1.

Data for **94**: R_f 0.60 [petrol:EtOAc (8:2)]; FT-IR ν_{max} (film)/cm^{-1} 2961, 2359, 2341, 1734, 1652, 1558, 1539, 1456, 1259, 1035; ^{1}H NMR (500 MHz, $CDCl_3$) δ 7.43–7.32 (2H, m), 7.33–7.20 (3H, m), 3.72–3.59 (1H, m), 3.45–3.28 (1H, m), 3.41 (3H, s), 2.90–2.62 (5H, m), 2.61–2.48 (2H, m), 1.97–1.72 (2H, m), 1.73–1.59 (4H, m), 1.57–1.48 (2H, m); ^{13}C NMR (126 MHz, $CDCl_3$) δ 142.9, 128.6, 128.4, 125.8, 72.8, 63.4, 59.0, 50.5, 33.3, 33.2, 30.6, 25.1; HRMS (ASAP): Found MH^+ 248.2007 $C_{16}H_{26}NO$ requires 248.2009.

4-Phenyl-1-(piperidin-1-yl)butan-2-ol (95)

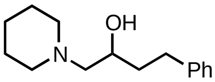

Following **GP7**, **11** (25 mg, 0.1 mmol, 1.0 equiv.) and KOH (0.5 mL, 1M, 5.0 equiv.) gave **95** (21 mg, 89 %) as an oil. R*f* 0.53 [CH_2Cl_2:$MeNO_2$:MeOH (4:1:1)]; FT-IR ν_{max} (film)/cm^{-1} 3349, 2932, 1453, 698; ^{1}H NMR (400 MHz, $CDCl_3$) δ 7.32–7.27 (2H, m), 7.25–7.17 (3H, m), 4.11 (1H, tdd, *J* = 9.3, 6.3, 3.4 Hz), 2.93 (1H, ddd, *J* = 13.9, 9.2, 4.8 Hz), 2.80 (1H, dd, *J* = 13.1, 6.2 Hz), 2.71 (1H, ddd, *J* = 13.8, 9.0, 7.3 Hz), 2.60 (1H, dd, *J* = 13.1, 8.8 Hz), 2.38–2.30 (4H, m), 2.31–2.21 (1H, m), 2.05 (1H, dtd, *J* = 14.2, 9.2, 4.7 Hz), 1.88–1.70 (1H, m), 1.59–1.48 (4H, m), 1.46–1.34 (2H, m); ^{13}C NMR (126 MHz, $CDCl_3$) δ 141.2, 128.7, 128.5, 126.2, 67.8, 54.7, 39.1, 35.4, 35.1, 26.0, 24.4 (the following signal was missing in the ^{13}C NMR and were identified by analysing the ^{1}H–^{13}C HMBC (500 MHz, $CDCl_3$) 35.1); HRMS (ESI$^+$): found MNa$^+$ 256.1660, $[C_{15}H_{23}NONa]^+$ requires 256.1672.

1-(1-Azido-4-phenylbutan-2-yl)piperidine (96) and 1-(2-azido-4-phenylbutyl)piperidine (96')

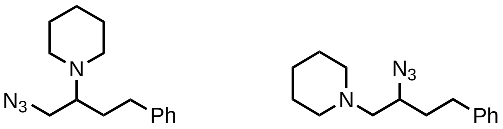

Following **GP7**, **11** (25 mg, 0.1 mmol) and NaN_3 (16 mg, 0.25 mmol) gave **96** and **96'** (26 mg, 99%) as an oil. **94**:**94'** = 3:1. R*f* 0.60 [petrol:EtOAc (8:2)]; FT-IR ν_{max} (film)/cm^{-1} 2961, 2359, 2341, 1716, 1489, 1447, 1314, 1258, 1093, 1066, 1024; ^{1}H NMR (500 MHz, $CDCl_3$) δ 7.34–7.24 (4H, m), 7.24–7.14 (6H, m), 3.48 (1H, m), 3.41 (1H, dd, *J* = 12.7, 7.6 Hz), 3.07 (1H, dd, *J* = 12.6, 5.2 Hz), 2.81 (1H, ddd, *J* = 14.5, 9.6, 5.4 Hz), 2.73–2.61 (4H, m), 2.60–2.50 (4H, m), 2.49–2.41 (2H, m), 2.40–2.31 (3H, m), 1.91–1.73 (3H, m), 1.73–1.63 (2H, m), 1.62–1.51 (8H, m), 1.49–1.39 (4H, m); ^{13}C NMR (126 MHz, $CDCl_3$, mixture of isomers) δ 142.1, 141.3, 128.5, 128.4, 128.4, 128.4, 126.0, 125.8, 63.6, 63.5, 59.1, 55.0, 50.9, 49.8, 34.4, 33.2, 32.4, 30.2, 26.5, 26.0, 25.0, 24.3; HRMS (ASAP): Found MH$^+$ 259.1917 $C_{15}H_{23}N_4$ requires 259.1911.

5-Phenyl-3-(piperidin-1-yl)pentanenitrile (97)

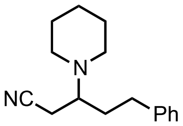

Following **GP7**, **11** (25 mg, 0.1 mmol) and NaCN (12 mg, 0.25 mmol) gave **97** (23 mg, 95%) as an oil. R_f 0.60 [petrol:EtOAc (8:2)]; FT-IR ν_{max} (film)/cm^{-1} 2933, 2853, 2359, 2341, 1495, 1454, 1259, 1035; ^{1}H NMR (500 MHz, $CDCl_3$) δ 7.31–7.26 (2H, m), 7.23–7.17 (3H, m), 2.85–2.78 (1H, m), 2.78–2.65 (2H, m), 2.64–2.53 (2H, m), 2.46 (1H, dd, *J* = 16.8, 5.5 Hz), 2.39–2.33 (2H, m), 2.30 (1H, dd, *J* = 16.9, 7.1 Hz), 2.00–1.89 (1H, m), 1.89–1.78 (1H, m), 1.69–1.51 (4H, m), 1.49–1.39 (2H, m); ^{13}C NMR (126 MHz, $CDCl_3$) δ 141.8, 128.6, 128.5, 126.1, 119.5, 60.5, 49.4, 33.1, 32.5, 26.5, 24.9, 16.9; HRMS (ASAP): Found MH$^+$ 243.1857 $C_{16}H_{23}N_2$ requires 243.1856.

1-(2-Fluoro-4-phenylbutyl)piperidine (98)

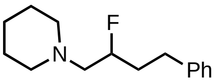

A tube equipped with a stirring bar was capped with a Supelco aluminium crimp seal with septum (PTFE/butyl), evacuated and refilled with N_2 (x 3). A solution of **11** (48 μL, 0.2 mmol, 1.0 equiv.) in $CHCl_3$ (0.2 mL, 1.0 M) was added followed by Et_3N•3HF (228 μL, 1.4 mmol, 7.0 equiv.). The mixture was stirred at 60 ºC for 16 h. After cooling to room temperature, H_2O (10 mL) was added and the layer were separated. The aquous layer was extracted with CH_2Cl_2 (3 x 10 mL). The combined organic layers were dried ($MgSO_4$), filtered and evaporated. Purification by flash column chromatography on silica gel gave **98** (7.5 mg, 32%) as an oil. R_f 0.40 [CH_2Cl_2:acetone (99:1)]; FT-IR ν_{max} (film)/cm^{-1} 2961, 2359, 2341, 1716, 1489, 1447, 1314, 1258, 1093, 1066, 1024; ^{1}H NMR (500 MHz, $CDCl_3$) δ 7.32–7.26 (2H, m), 7.23–7.16 (3H, m), 4.68 (1H, dddd, *J* = 50.2, 11.3, 7.3, 3.3 Hz), 2.82 (1H, ddd, *J* = 14.7, 9.8, 5.3 Hz), 2.70 (1H, ddd, *J* = 13.1, 9.2, 6.7 Hz), 2.65–2.53 (1H, m), 2.50–2.34 (5H, m), 2.05–1.76 (2H, m), 1.58 (4H, p, *J* = 5.6 Hz), 1.50–1.38 (2H, m); ^{13}C NMR (126 MHz, $CDCl_3$) δ 141.5, 128.6, 128.6, 126.1, 91.7 (d, *J* = 169.7 Hz), 63.5 (d, *J* = 21.0 Hz), 55.3, 35.7 (d, *J* = 21.0 Hz), 31.4 (d, *J* = 4.6 Hz), 26.0, 24.3; ^{19}F NMR (376 MHz, $CDCl_3$) δ –181.5; HRMS (ASAP): Found MH$^+$ 236.1814, $C_{15}H_{23}NF$ requires 236.1809.

1-(4-Phenylbutan-2-yl)piperidine (99)

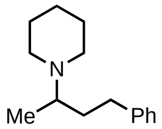

A tube equipped with a stirring bar was charged with NaI (75 mg, 0.5 mmol, 5.0 equiv.) and then capped with a Supelco aluminium crimp seal with septum (PTFE/butyl), evacuated and refilled with N_2 (x 3). A solution of **11** (25 mg, 0.1 mmol, 1.0 equiv.) in CH_3CN (0.5 mL) was added and the mixture was stirred at room temperature for 18 h. The mixture was cooled to 0 °C, treated with $LiAlH_4$ (0.10 mL, 0.1 mmol, 1.0 equiv., 1.0 M in THF) and stirred for 5 minutes. H_2O (0.04 mL) and 1 M NaOH (0.04 mL) were added. The mixture was stirred for 15 minutes and diluted with 1 M KOH (3 mL) and EtOAc (3 mL). The layers were separated and the organic layer was dried ($MgSO_4$), filtered and evaporated to give **99** (21 mg, 96%) as an oil. FT-IR ν_{max} (film)/cm^{-1} 2359, 2342; ^{1}H NMR (500 MHz, $CDCl_3$) δ 7.29–7.23 (2H, m), 7.22–7.12 (3H, m), 2.71–2.52 (3H, m), 2.51–2.44 (2H, m), 2.42–2.31 (2H, m), 1.90–1.80 (1H, ddt, *J* = 13.4, 10.1, 5.9 Hz), 1.62–1.48 (5H, m), 1.42 (2H, p, *J* = 5.9 Hz), 0.98 (3H, d, *J* = 6.6 Hz). ^{13}C NMR (126 MHz, $CDCl_3$) δ 143.1, 128.6, 128.4, 125.7, 59.0, 49.4, 35.7, 33.4, 26.7, 25.2, 13.9; HRMS (ESI$^+$): found MH$^+$ 218.1895, $[C_{15}H_{24}N]^+$ requires 218.1909. Data in accordance with literature.[17]

1-(1-(2-Benzylphenoxy)propan-2-yl)piperidine (103)

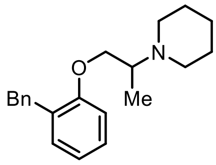

A tube equipped with a stirring bar was charged with NaI (75 mg, 0.5 mmol, 5.0 equiv.) and then capped with a Supelco aluminium crimp seal with septum (PTFE/butyl), evacuated and refilled with N_2 (x 3). A 0.1 M solution of **102** (35 mg, 0.1 mmol, 1.00 equiv.) in CH_3CN (1.0 mL) was added and the mixture was stirred at room temperature for 18 h. The mixture was cooled to 0 °C, treated with $LiAlH_4$ (0.10 mL, 0.1 mmol, 1.0 equiv., 1.0 M in THF) and stirred for 5 minutes. H_2O (0.04 mL) and 1 M NaOH (0.04 mL) were added. The mixture was stirred for 15 minutes and diluted with 1 M KOH (3 mL) and EtOAc (3 mL). The layers were separated and the organic layer was dried ($MgSO_4$), filtered and evaporated to give **103** as an oil (15 mg, 49%). ^{1}H NMR (400 MHz, $CDCl_3$) δ 7.33–7.00 (7H, m), 6.94–6.79 (2H, m), 4.09–4.03 (1H, m), 3.98 (2H,

s), 3.08–2.98 (1H, m), 2.64–2.50 (4H, m), 1.62–1.53 (4H, m), 1.46–1.36 (2H, m), 1.14 (3H, d, J = 6.8 Hz). Data in accordance with literature.[18]

10 Olefin Aminochlorination Scale-Up by Batch-to-Flow

10.1 General Experimental Details

The flow process was performed with the set-up shown in (Figure) on a Masterflex L/R model 77200-60 pump connected with a photochemical 450 nm LED reactor (PennOC photoreactor M1, Figure) containing Aldtech FT tubing (1.6 mm internal diameter). The calculated volume of solvent in the reactor was 6.66 mL.

10.2 General Flow Procedure

To a 250 mL flask charged with a stirring bar, was added NCS (9.310 g, 70 mmol, 1.0 equiv.), $Ru(bpy)_3Cl_2 \bullet 6H_2O$ (26.2 mg, 0.35 mmol 0.05 mol%) and CH_2Cl_2 (120 mL, 0.58 M). The heterogeneous solution was sonicated for 5 minutes until complete solubilisation of NCS, then cooled to 0 °C. **1** (7 mL, 70 mmol, 1.0 equiv.) was then added dropwise over 10 minutes under vigorous stirring. The solution was then allowed to warm to room temperature stirring for 1 h. TFA (32 mL, 420 mmol, 6.0 equiv.) was then added giving a homogeneous bright orange solution which was divided in three fractions. Each fraction was poured in a 100 mL flask (approx. 53 mL of crude in each flask). Prior to the pumping of the reaction, the reactor was fully liquid filled with CH_2Cl_2 from the solvent reservoir. **10** (31.5 mL, 70 mmol, 1.0 equiv.) was divided in three portions (10.5 mL, 23.33 mmol, 0.33 equiv. each) and added sequentially in each of the three flasks under vigorous stirring, pumping the solution into the system at the end of every addition. Once the entire content of the three flasks was pumped through the reactor (approx. 3-4 minutes into the system), CH_2Cl_2 was allowed to flush from the solvent reservoir, until all the reaction solution had been collected (approx. 2 minutes). The reaction solution was pumped at 46 mL/min resulting in a theoretical residence time of 8.7 s within the photochemical reactor. The collected homogeneous orange solution was added dropwise over 30 min to a 0 °C solution of 3.5 M sodium hydroxide (200 mL, 10 equiv.). The organic phase was collected, and the aqueous phase extracted with CH_2Cl_2 (50 mL x 3). The combined organic phases were dried ($MgSO_4$), filtered, and evaporated to give an oil. The crude was then purified by column chromatography on silica gel eluting cyclohexane:EtOAc (9:1) to give **11** as an oil (8.44 g, 48%). Low yield was the result of the partial evaporation of the product under the high vacuum used. The reaction was repeated on the same scale adding 1,3,5-trimethoxybenzene as internal standard. The yield in this case was 87% (17 mmol min^{-1}).

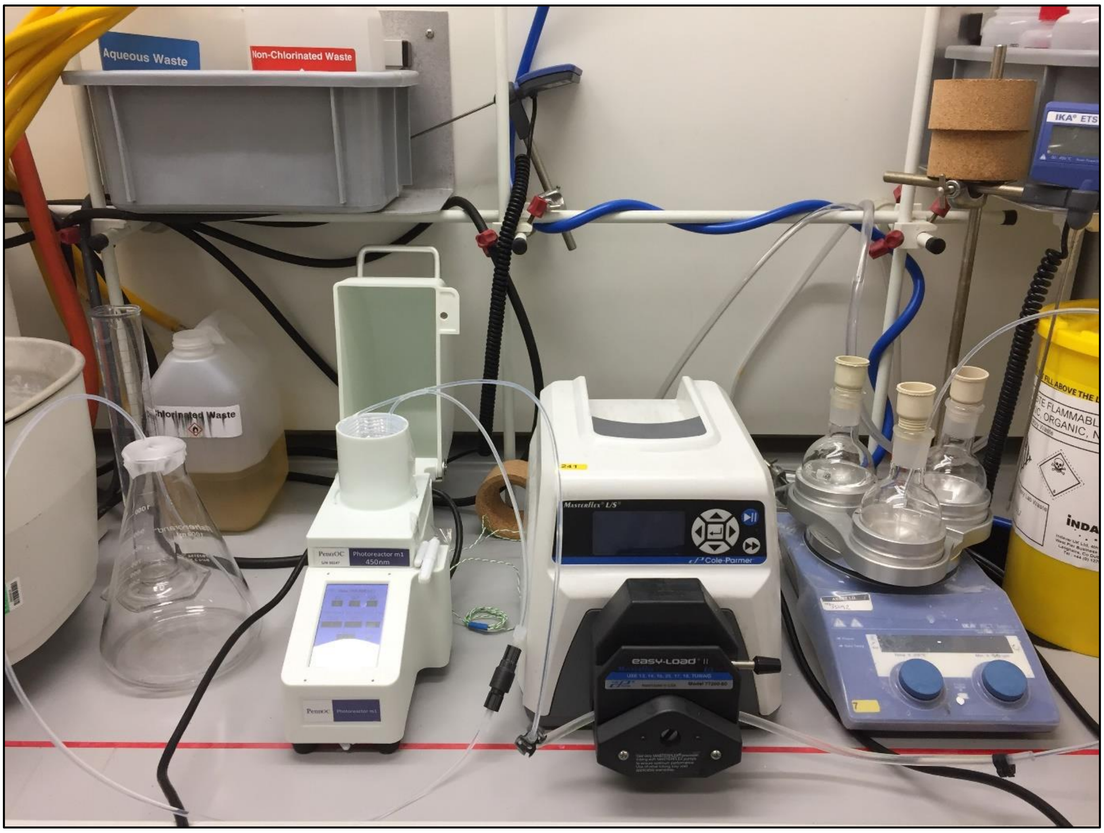

Figure 5.

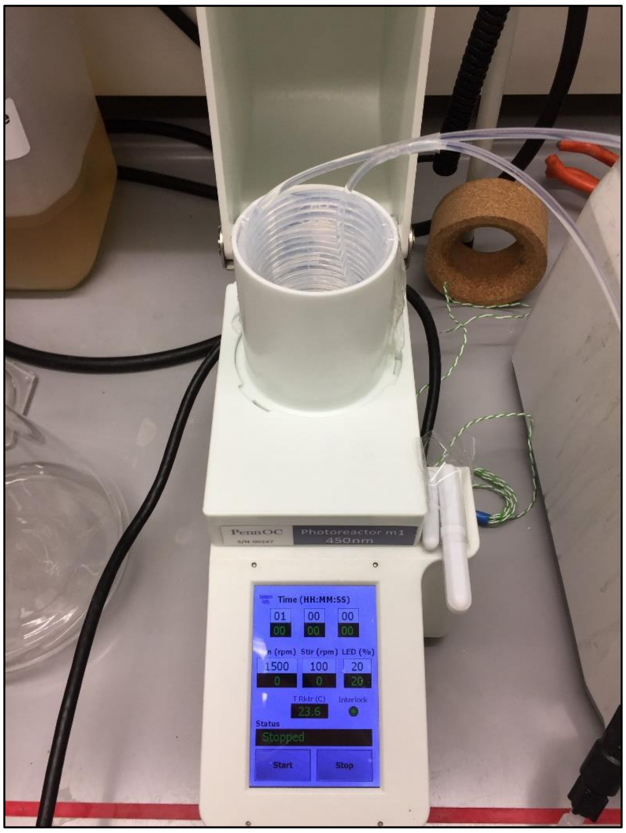

Figure 6.

11 NMR Spectra

S6 – ^{1}H NMR (500 MHz, CDCl$_3$)

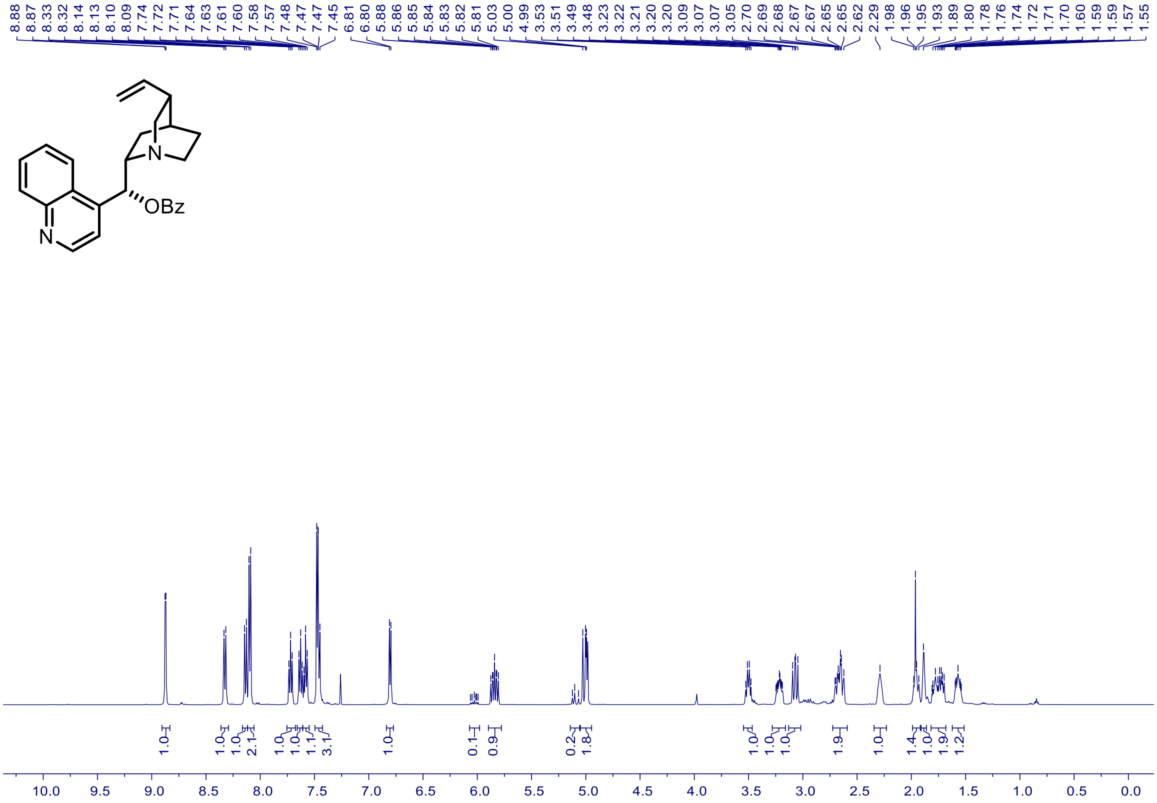

S6 – ^{13}C NMR (125 MHz, CDCl$_3$)

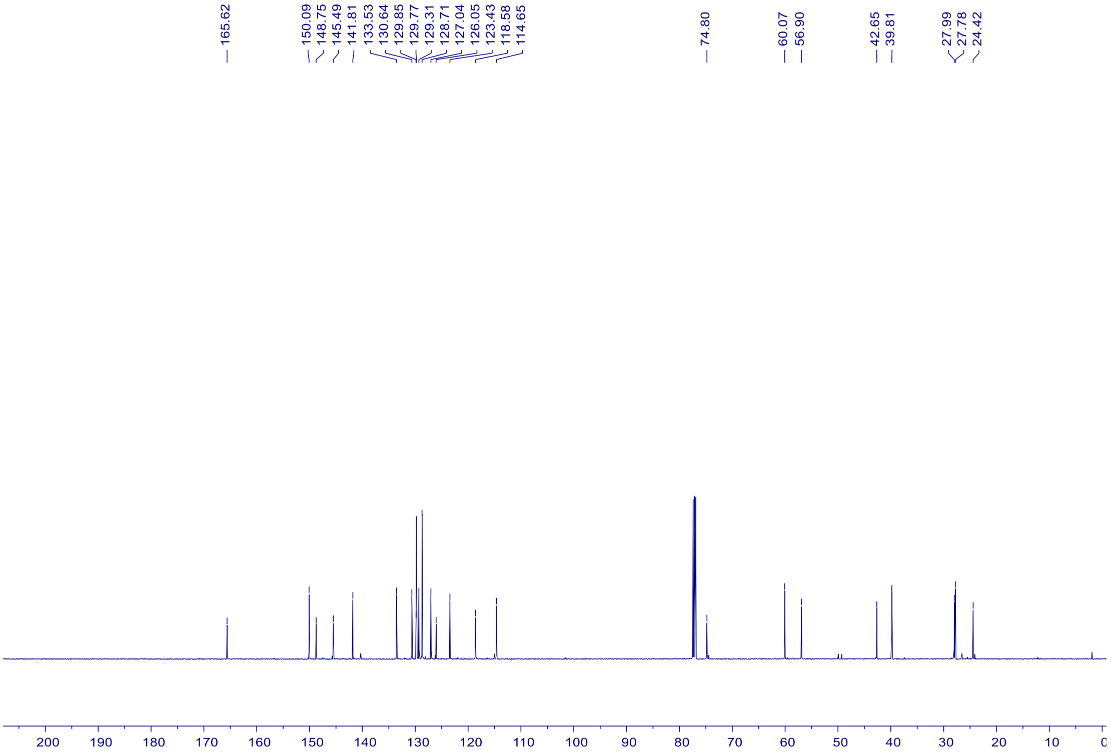

S7 – ^{1}H NMR (400 MHz, $CDCl_3$)

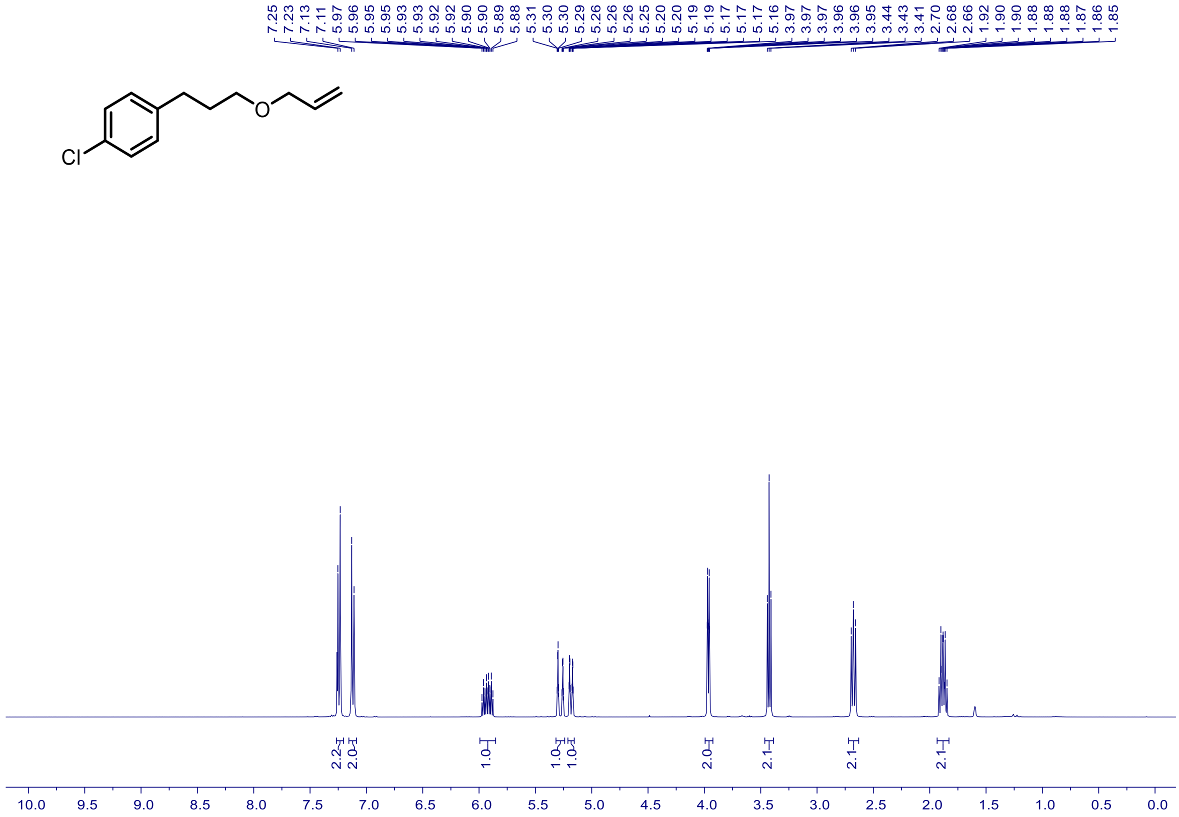

S7 – ^{13}C NMR (100 MHz, $CDCl_3$)

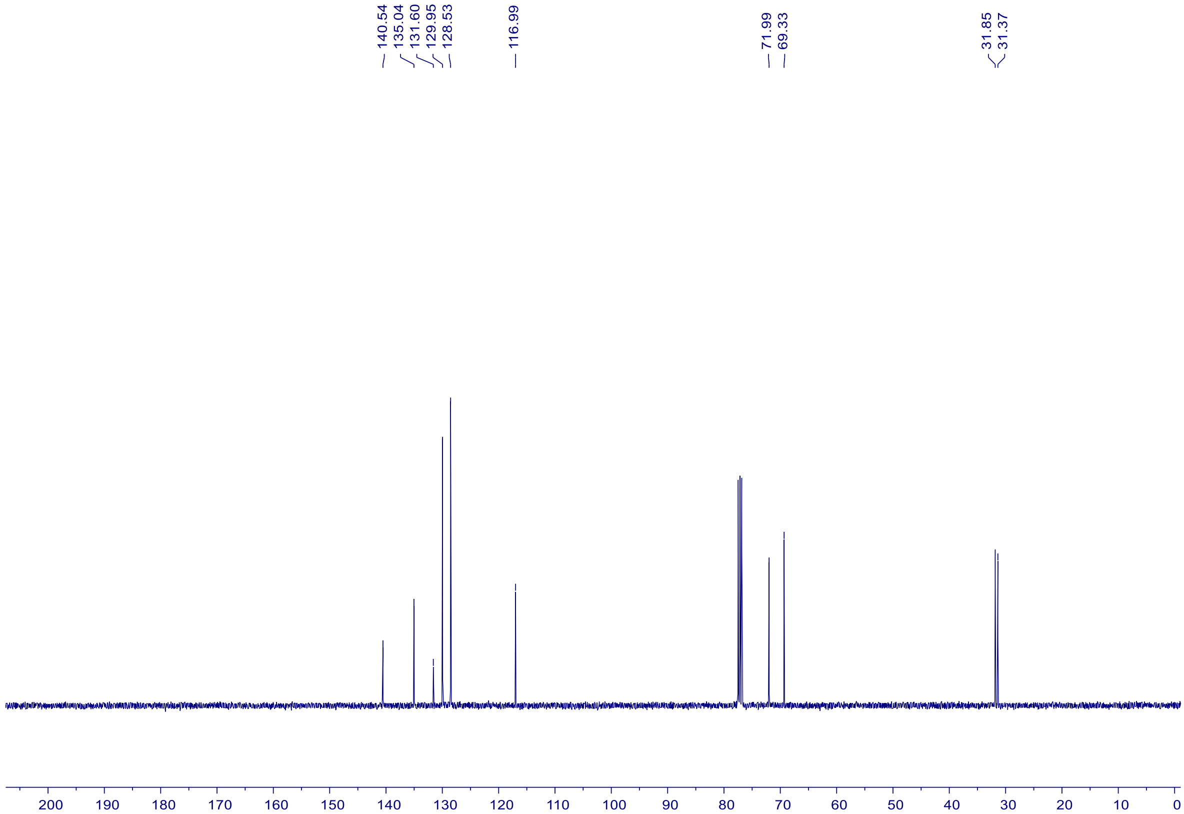

S9 - ^{1}H NMR (400 MHz, $CDCl_3$)

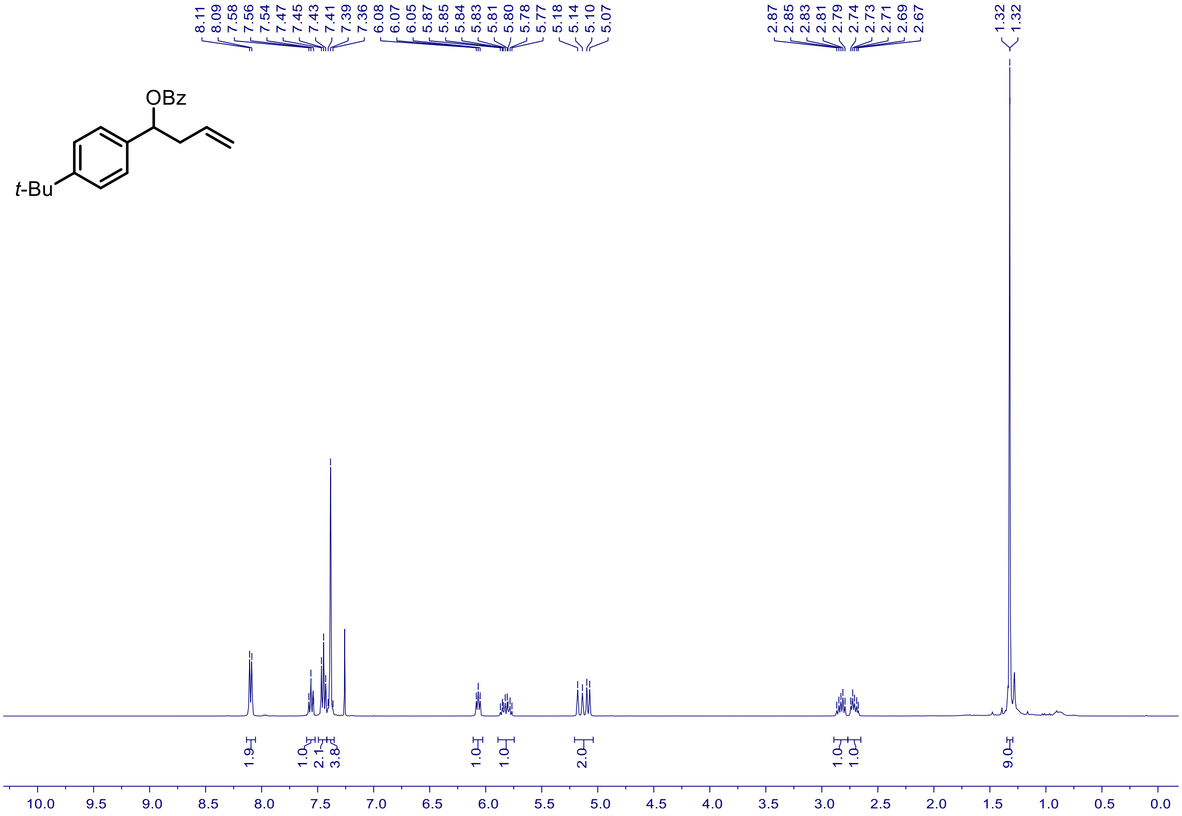

S9 – ^{13}C NMR (125 MHz, $CDCl_3$)

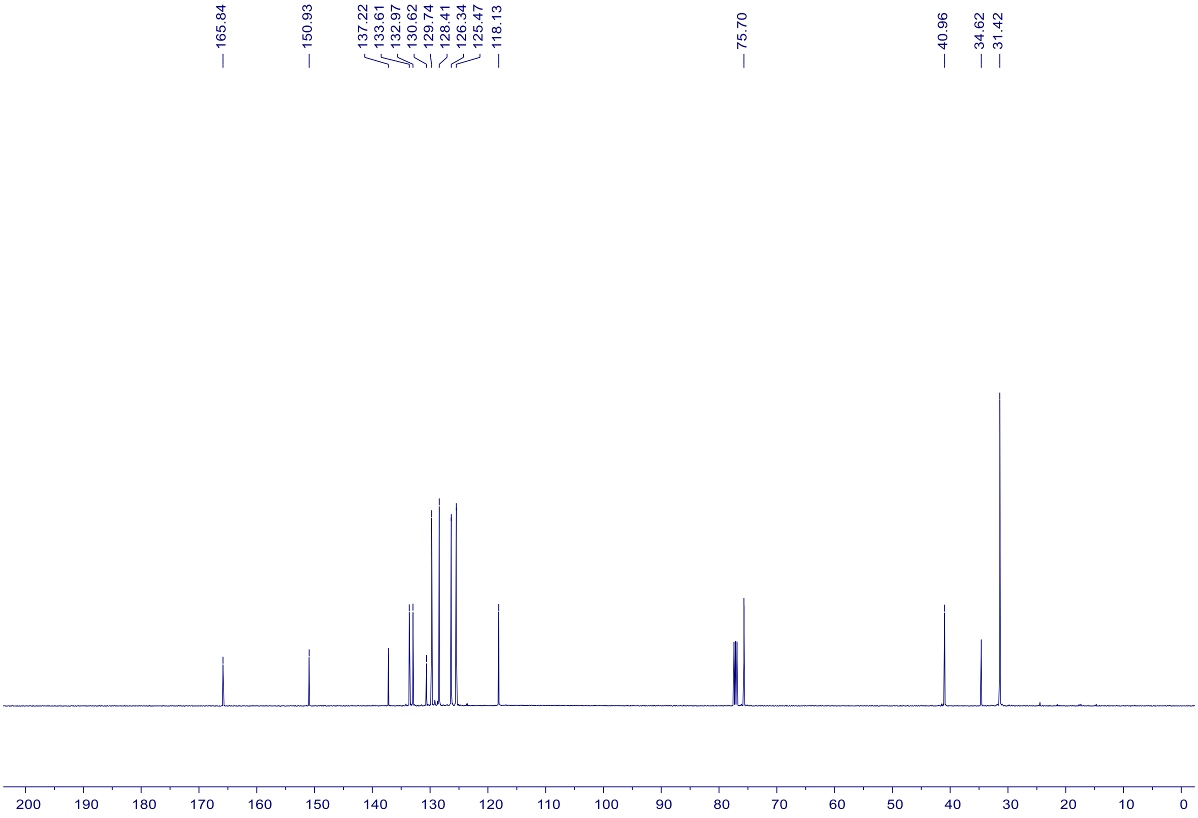

11 – ^{1}H NMR (500 MHz, $CDCl_3$)

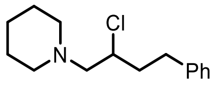

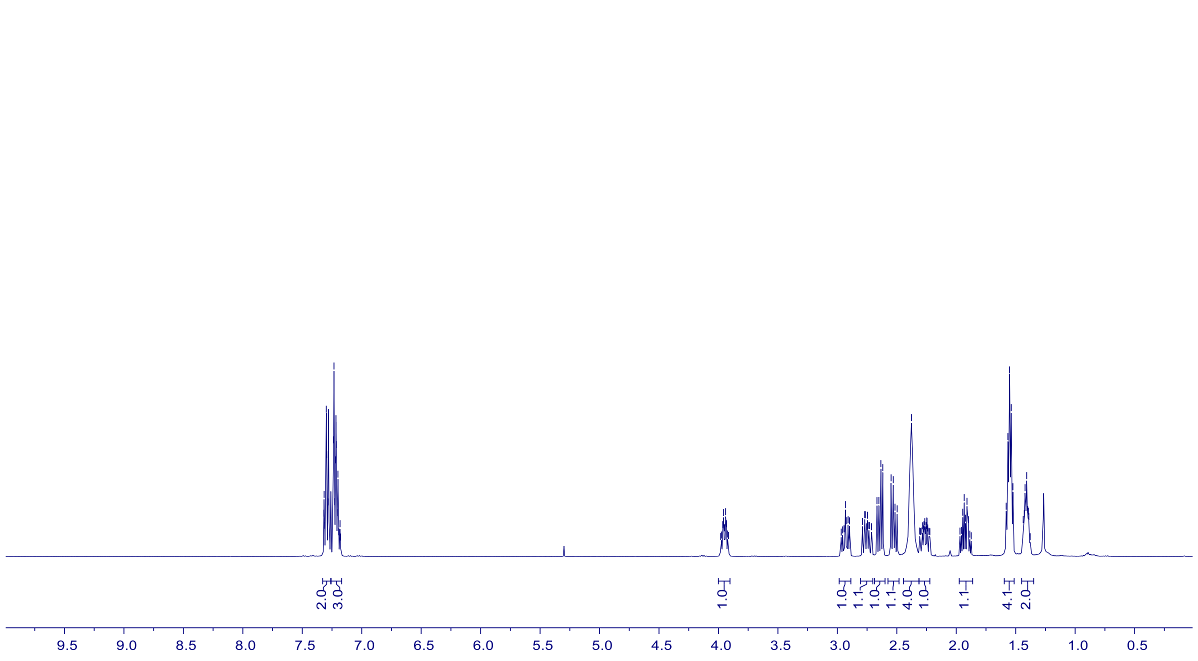

11 – ^{13}C NMR (126 MHz, $CDCl_3$)

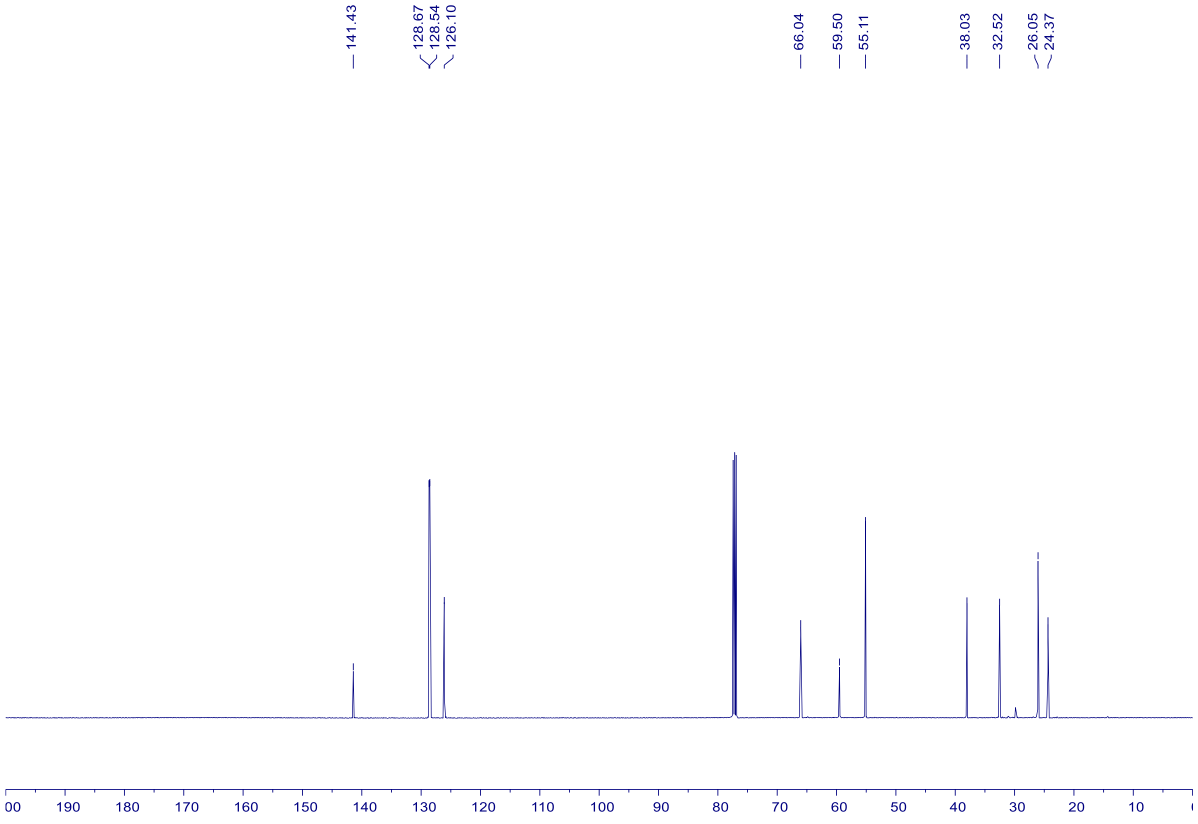

12 – ^{1}H NMR (500 MHz, $CDCl_3$)

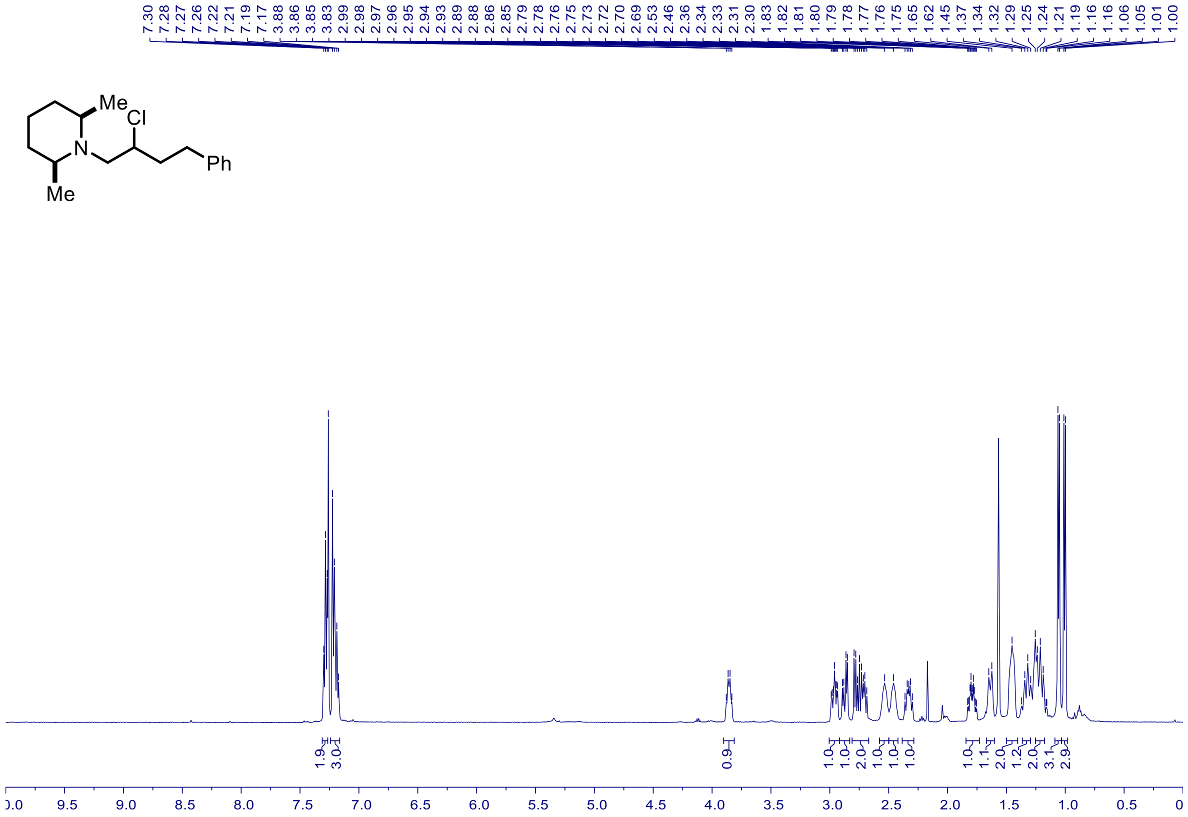

12 – ^{13}C NMR (126 MHz, $CDCl_3$)

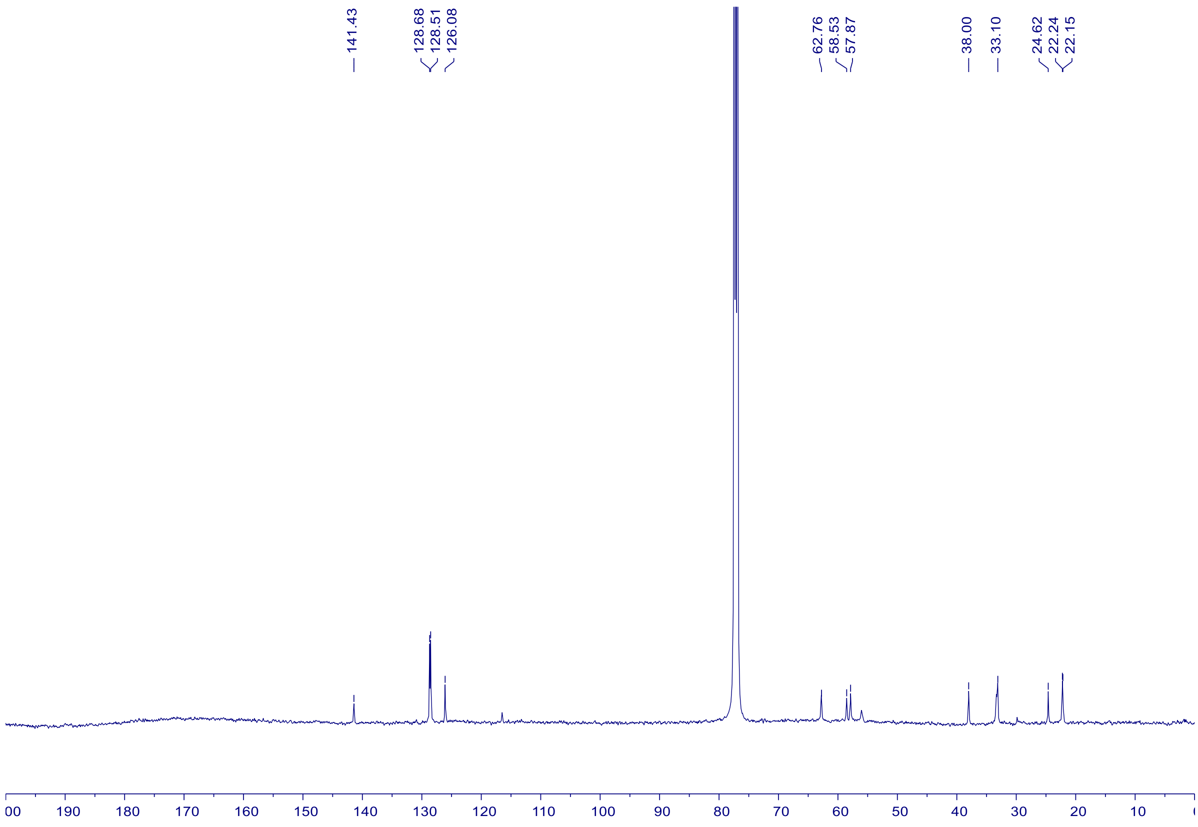

13 – ^{1}H NMR (500 MHz, CDCl$_3$)

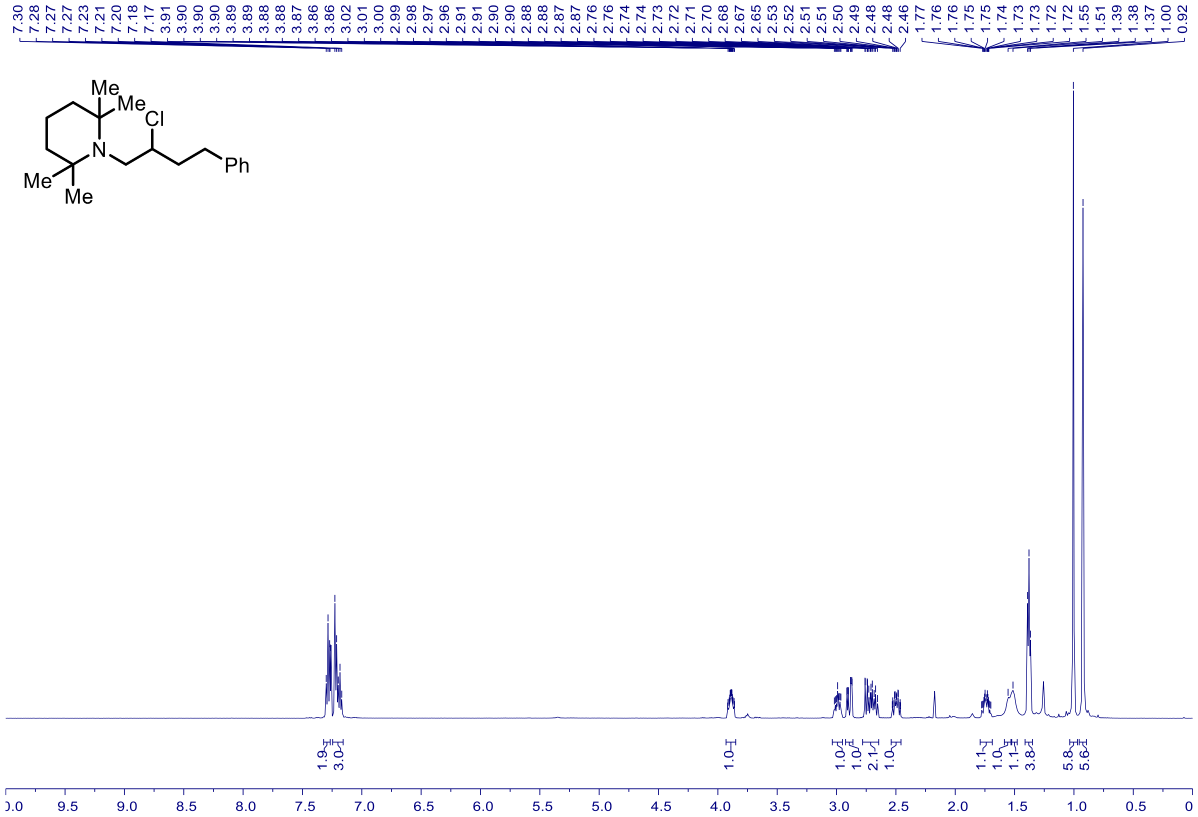

13 – ^{13}C NMR (126 MHz, CDCl$_3$)

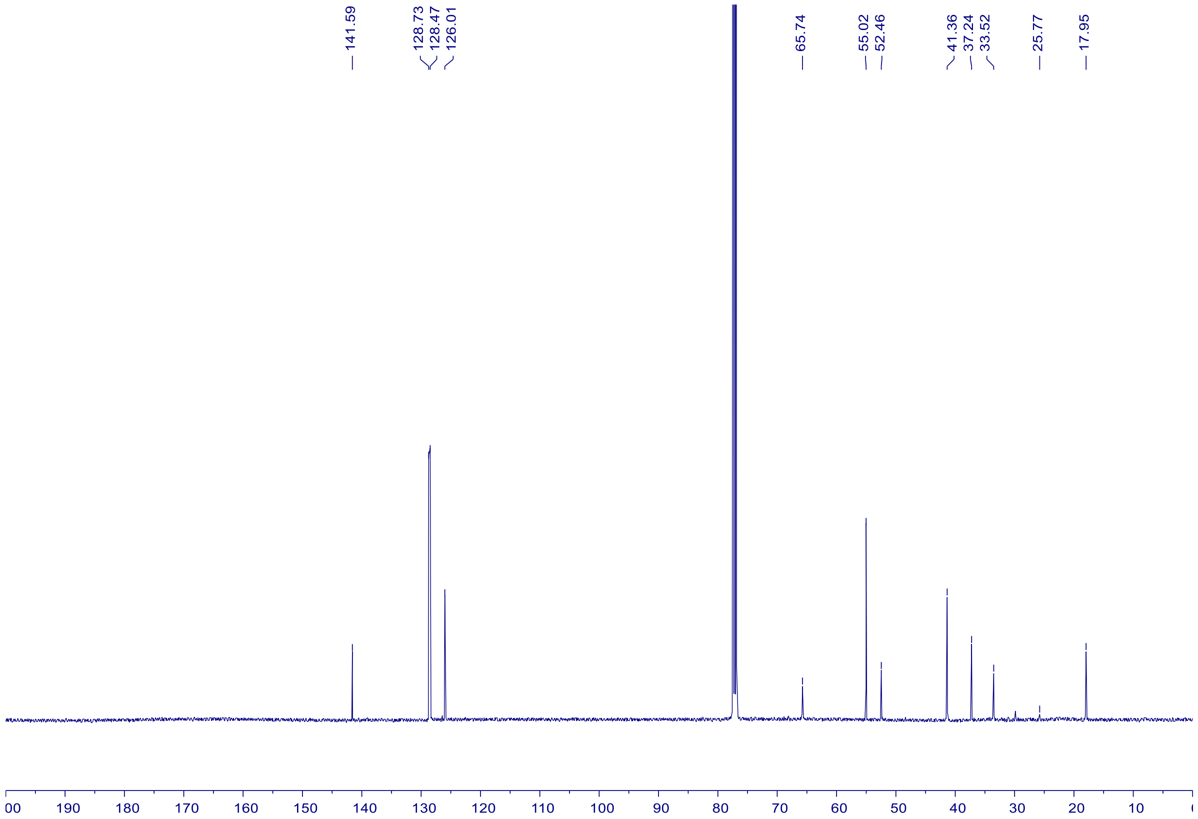

14 – ^{1}H NMR (500 MHz, $CDCl_3$)

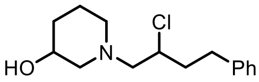

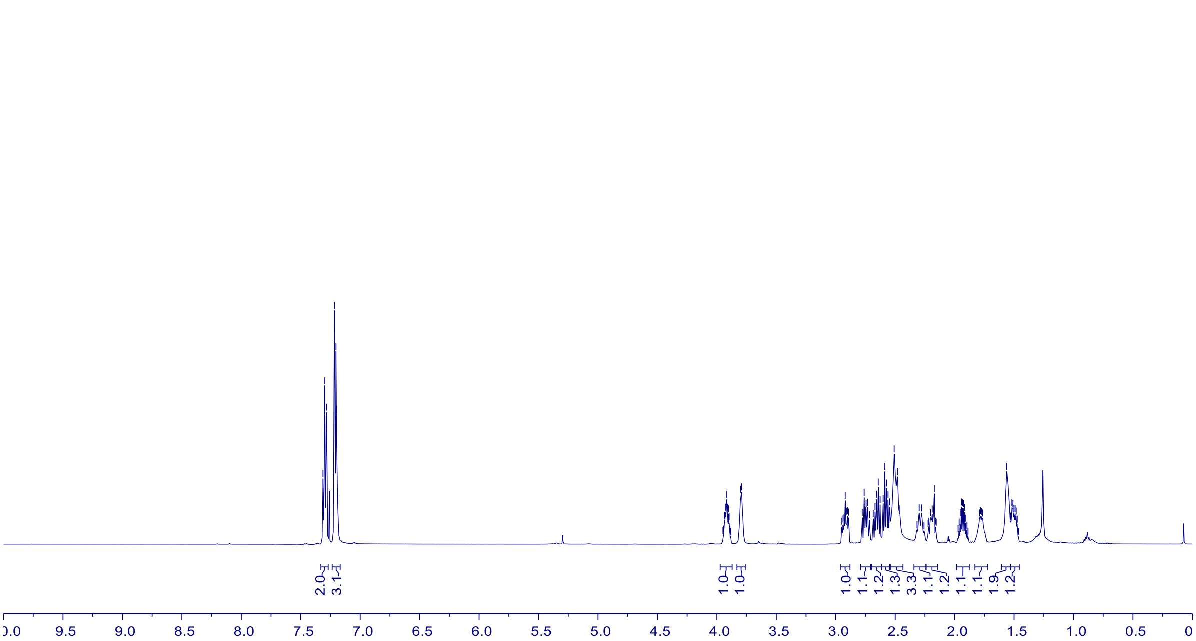

14 – ^{13}C NMR (126 MHz, $CDCl_3$)

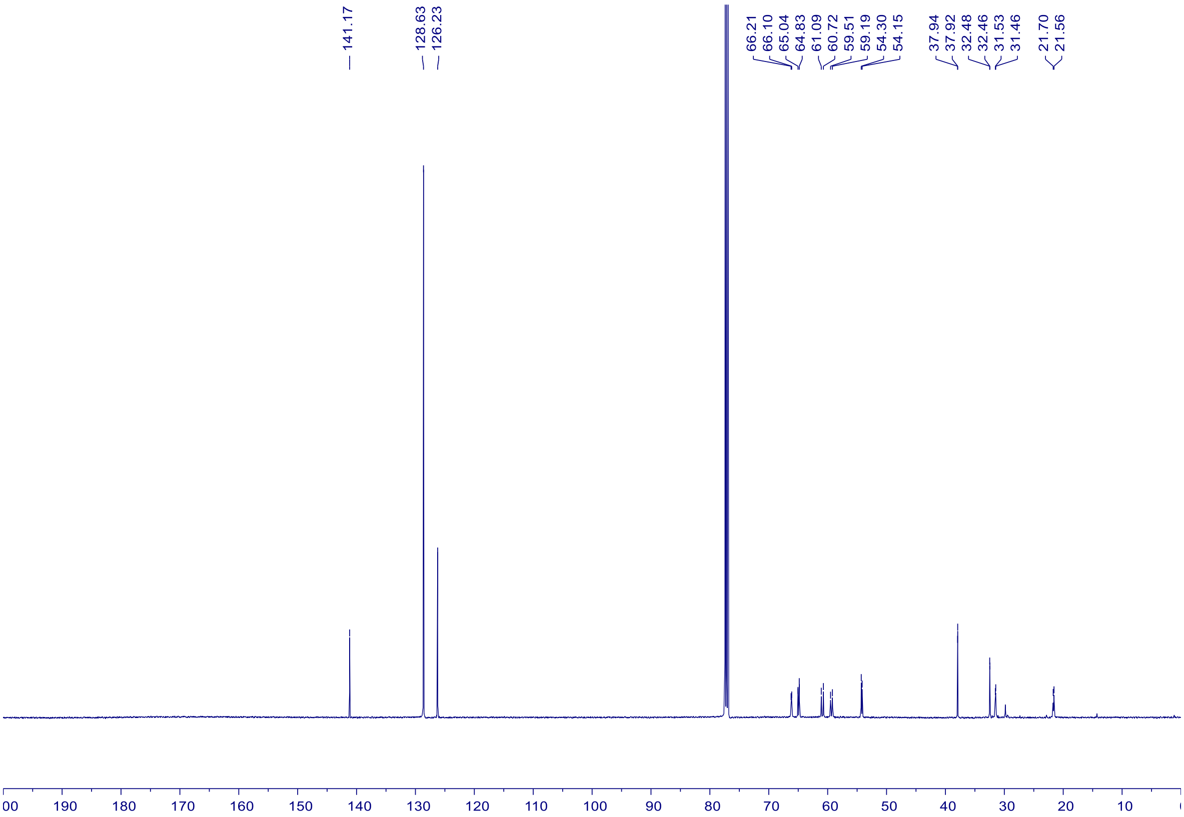

15 – ^{1}H NMR (500 MHz, CDCl$_3$)

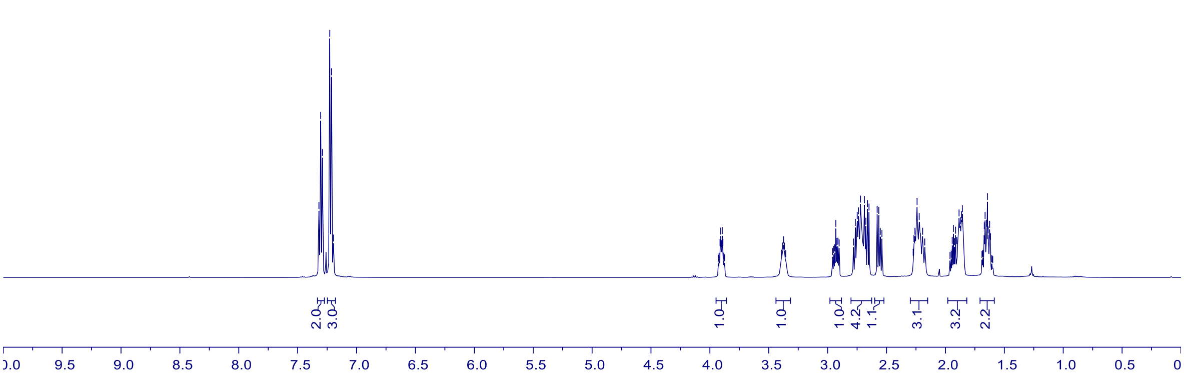

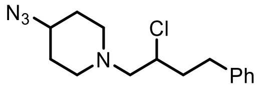

15 – ^{13}C NMR (126 MHz, CDCl$_3$)

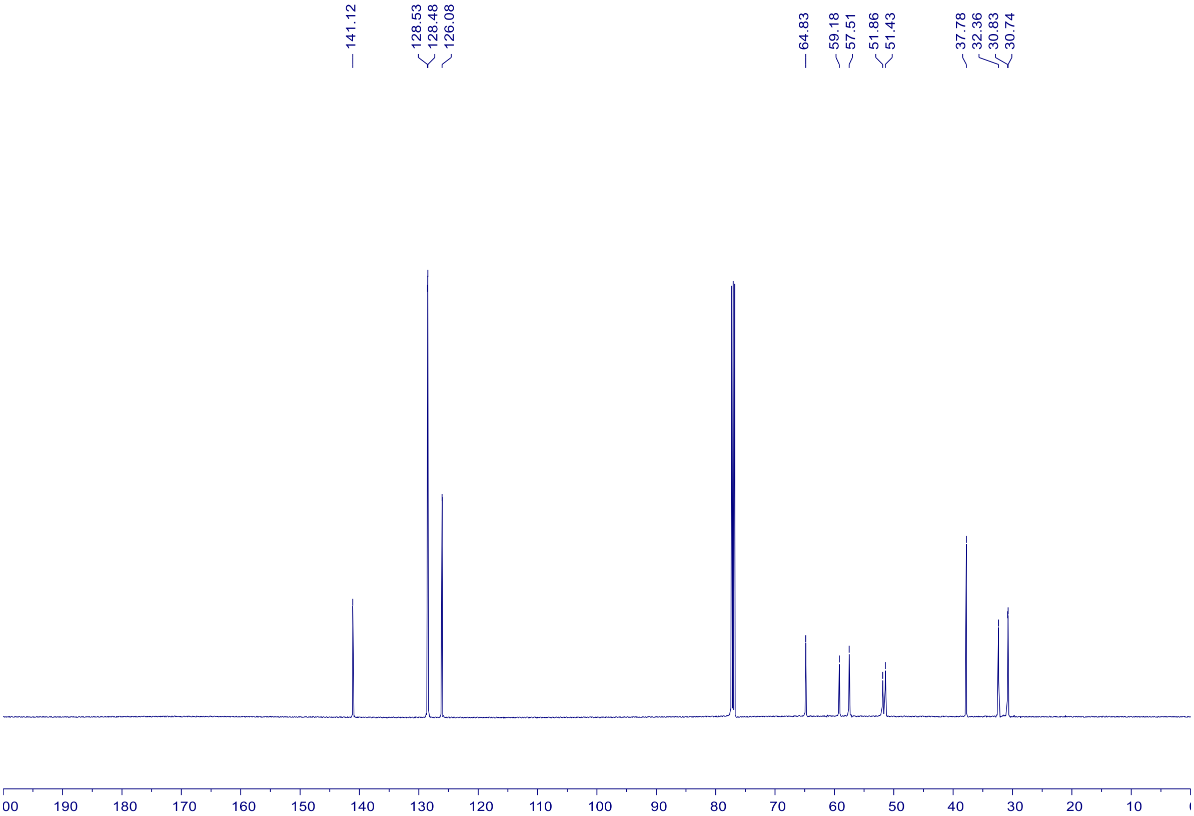

16 – ^{1}H NMR (500 MHz, $CDCl_3$)

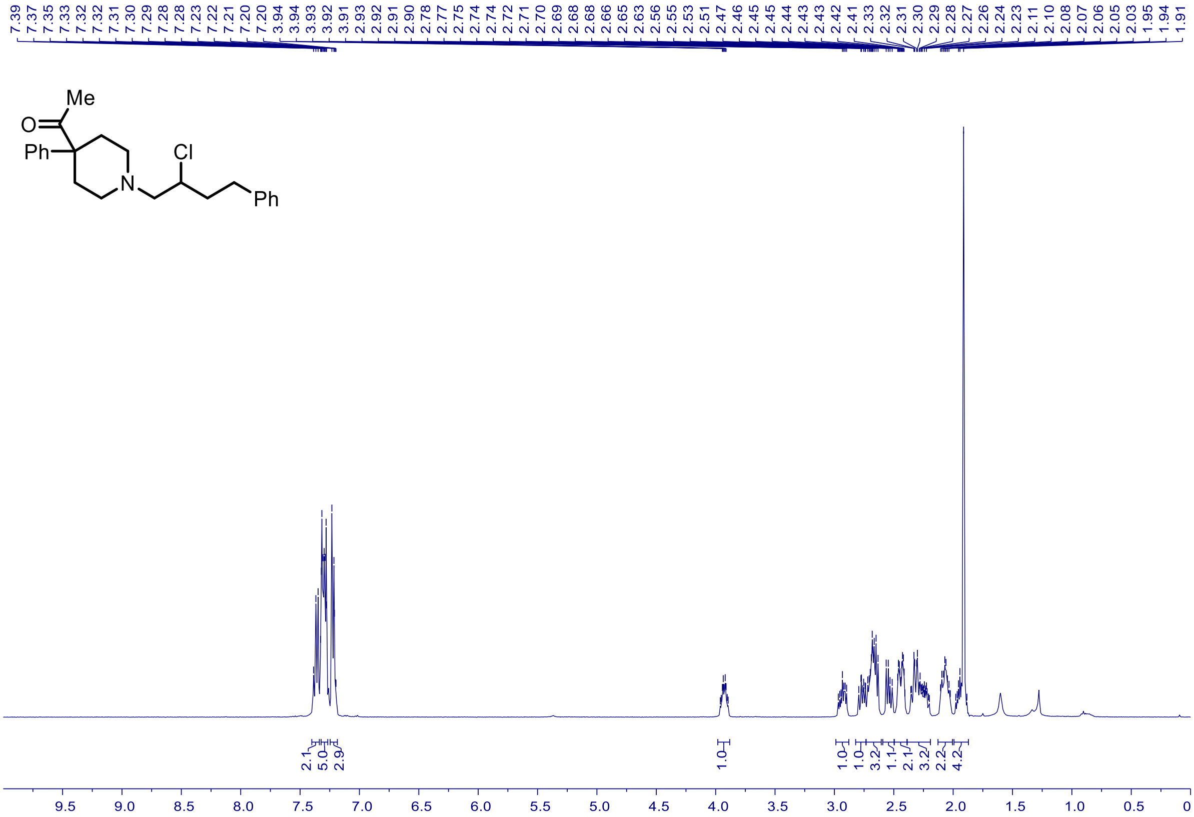

16 – ^{13}C NMR (126 MHz, $CDCl_3$)

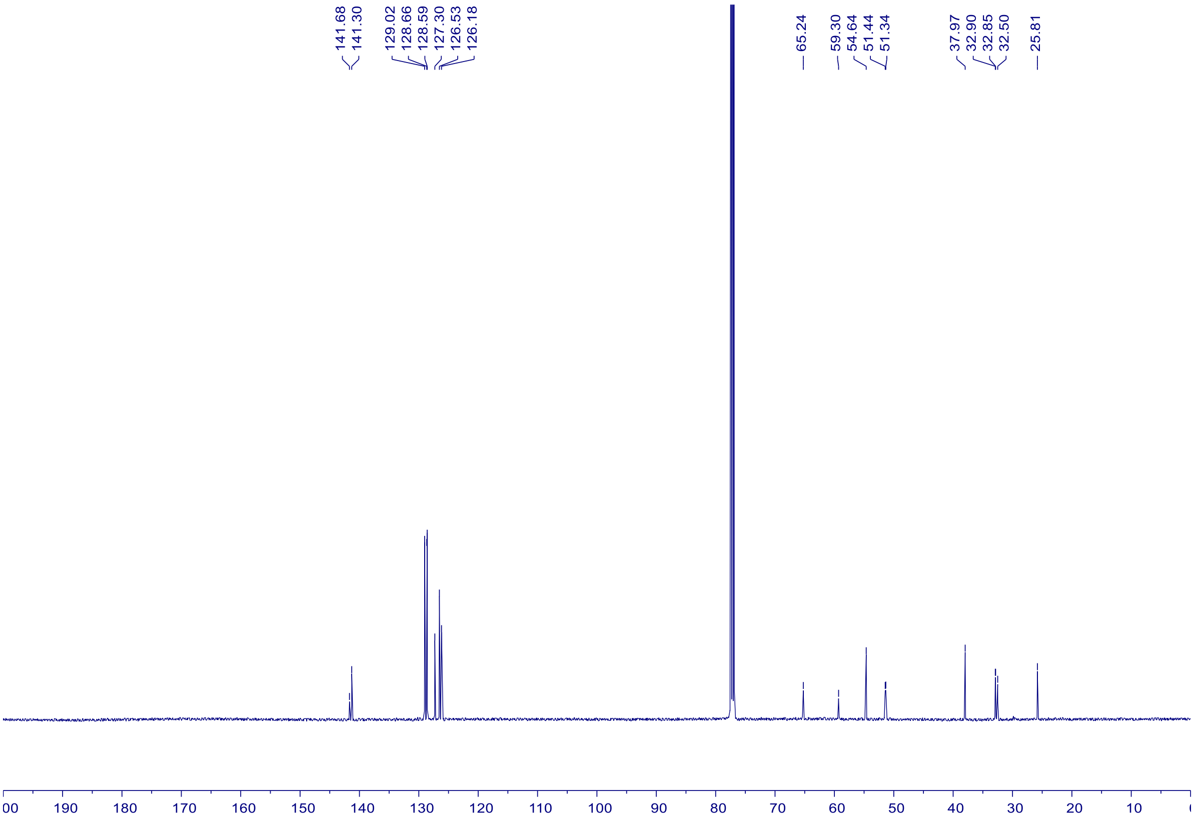

17 – ^{1}H NMR (500 MHz, $CDCl_3$)

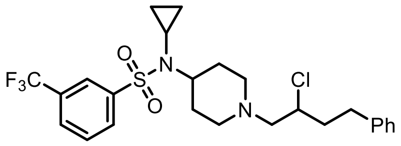

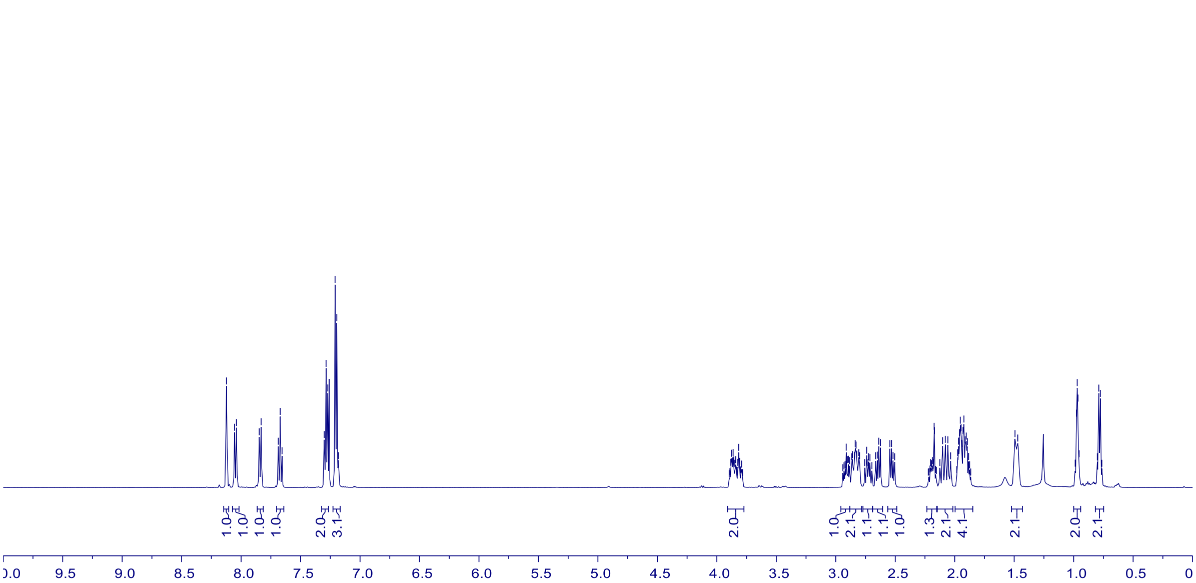

17 – ^{13}C NMR (126 MHz, $CDCl_3$)

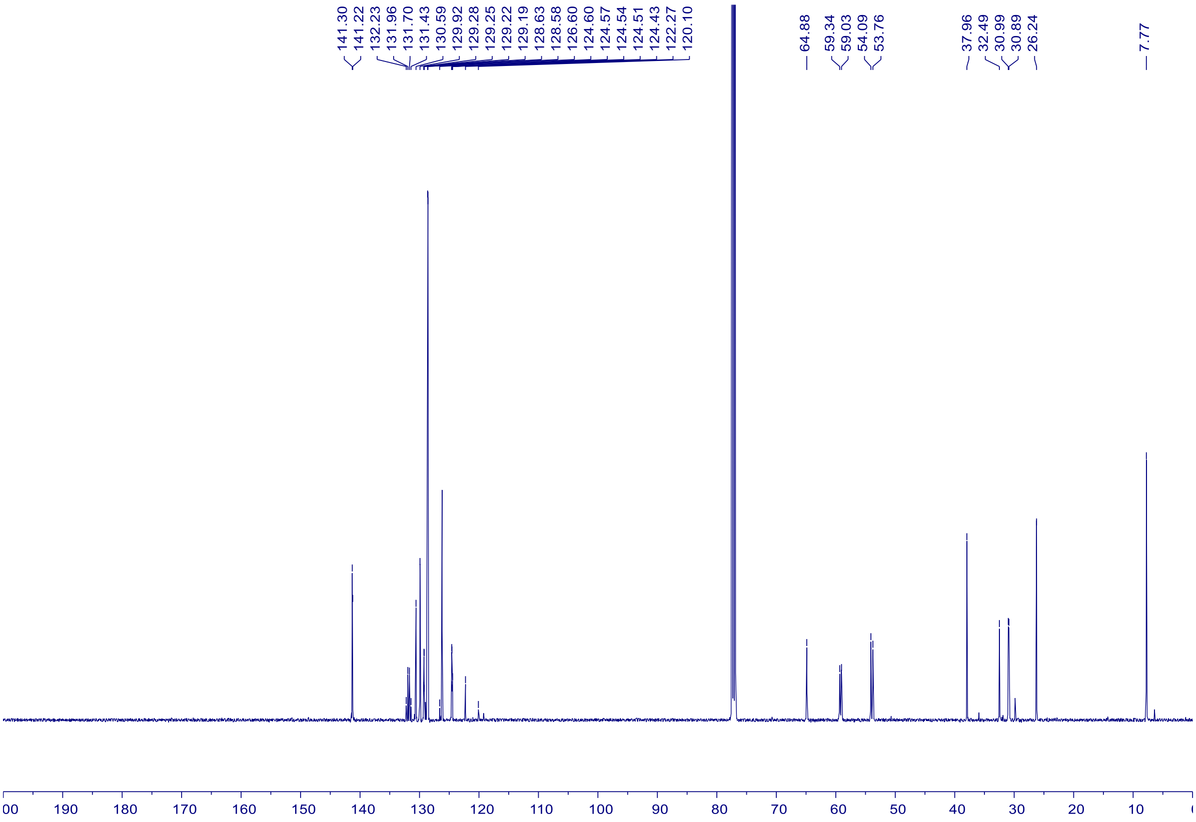

17 – ^{19}F NMR (471 MHz, $CDCl_3$)

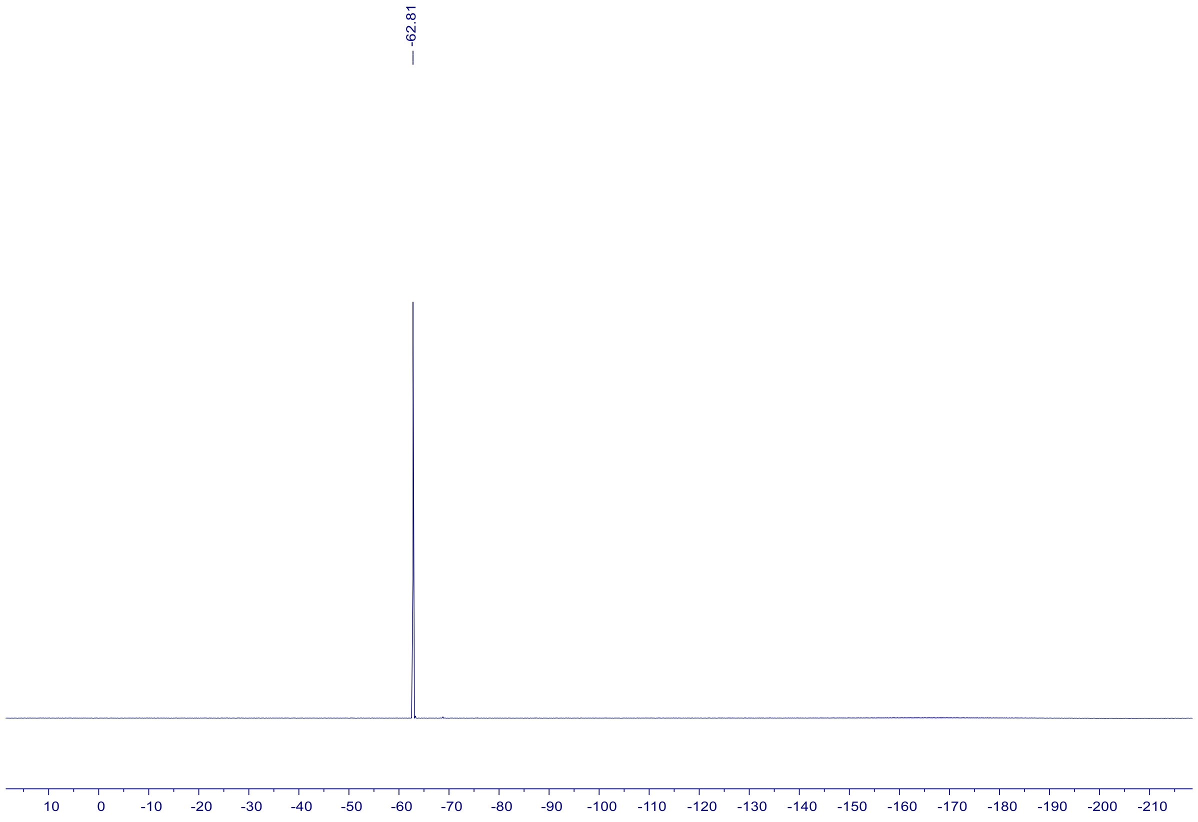

18 – ^{1}H NMR (500 MHz, CDCl$_3$)

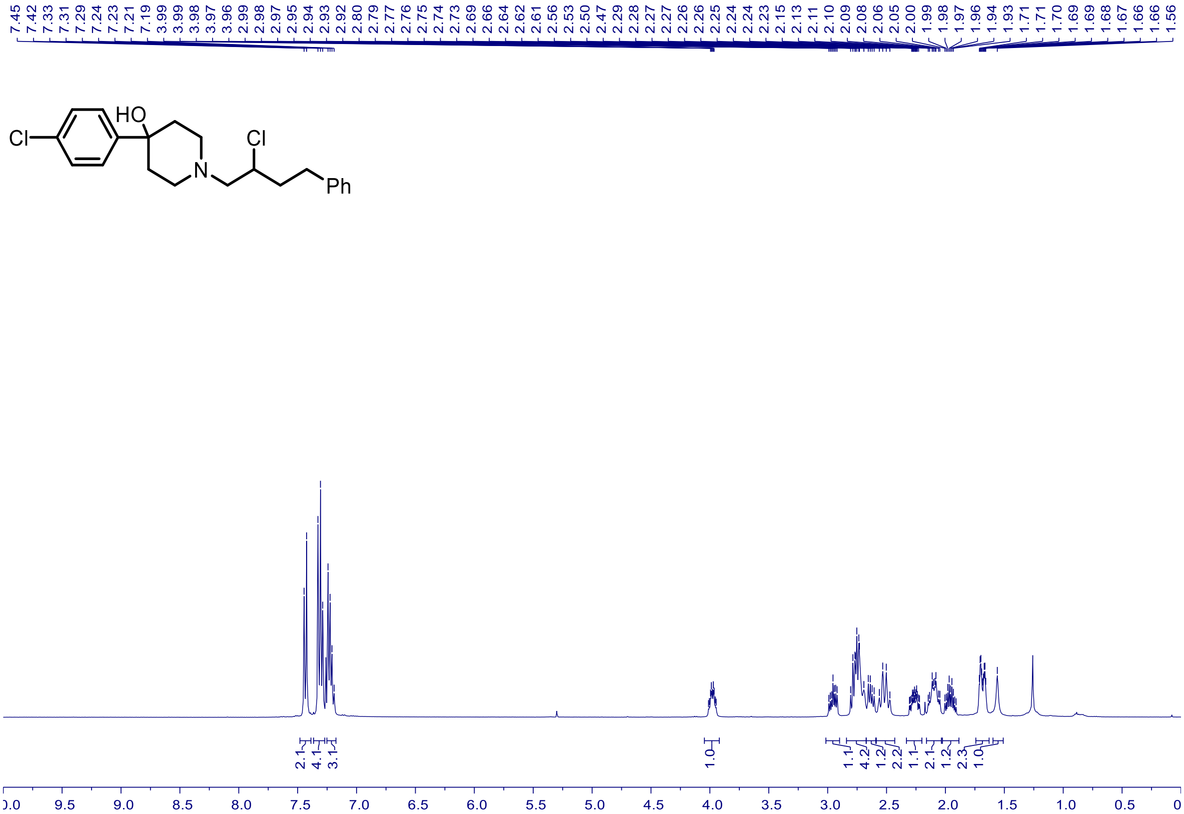

18 – ^{13}C NMR (126 MHz, CDCl$_3$)

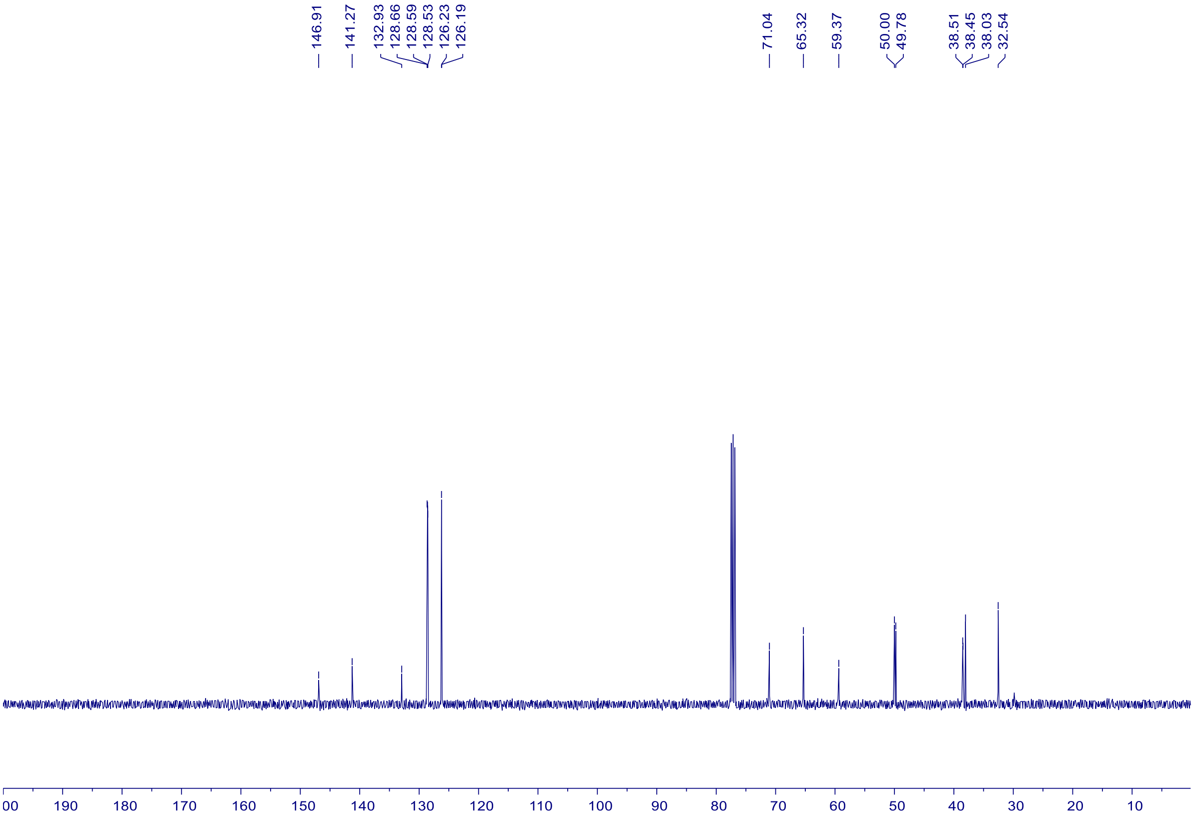

19 – ^{1}H NMR (500 MHz, $CDCl_3$)

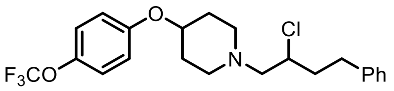

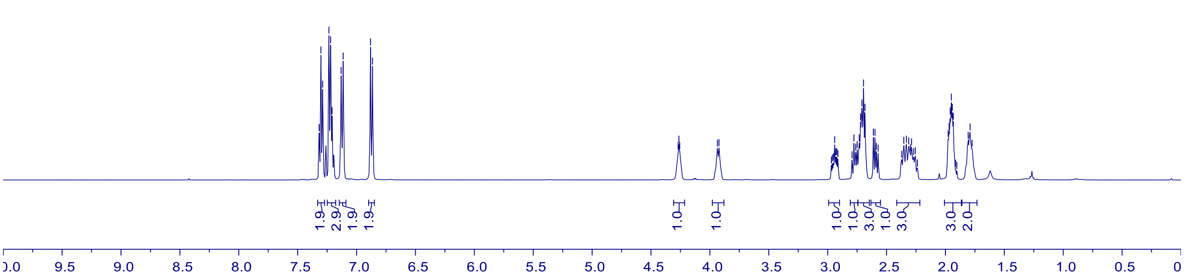

19 – ^{13}C NMR (126 MHz, $CDCl_3$)

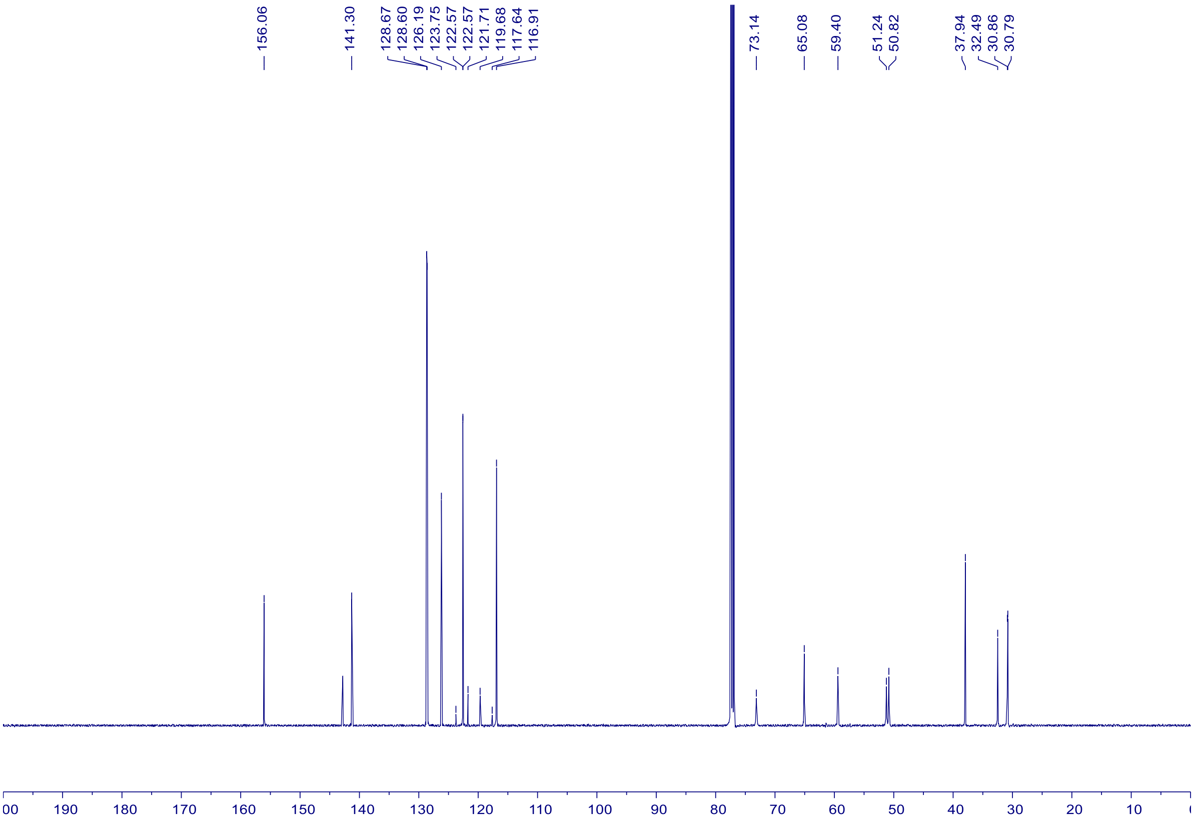

19 – ^{19}F NMR (471 MHz, $CDCl_3$)

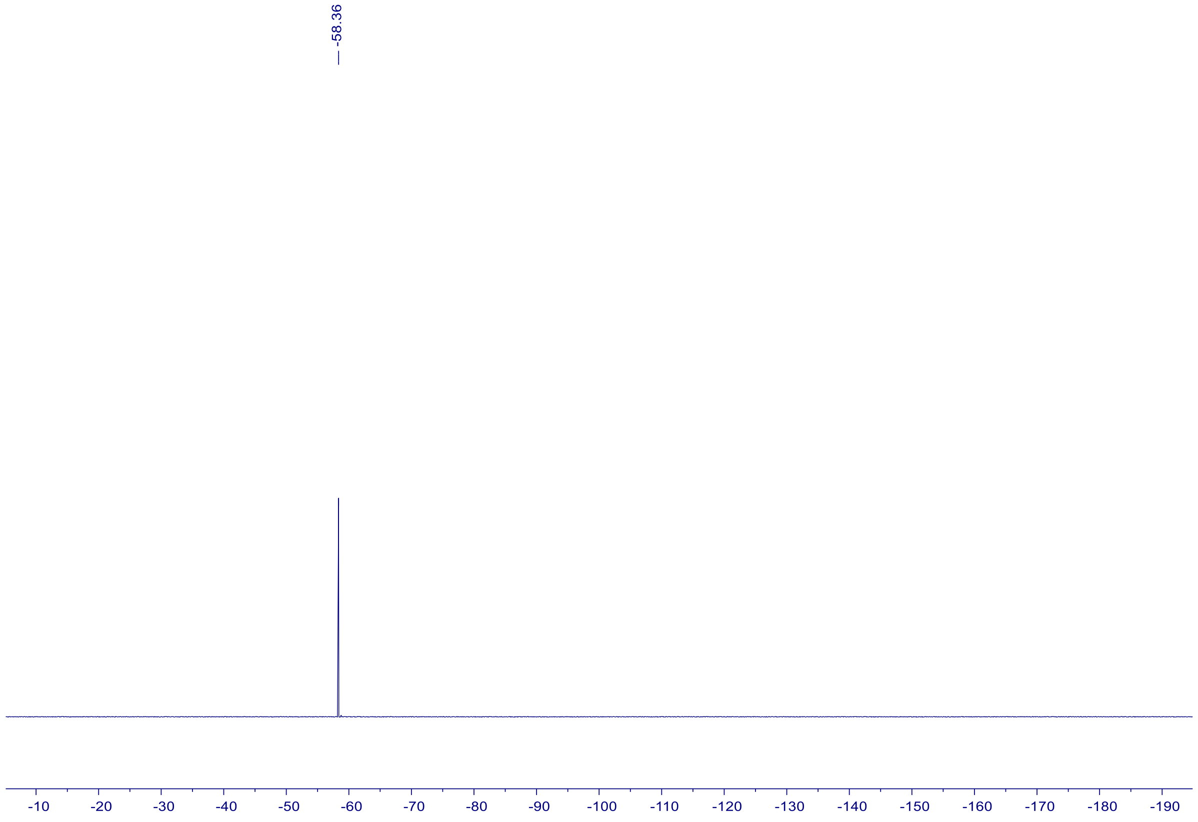

20 – ^{1}H NMR (500 MHz, CDCl3)

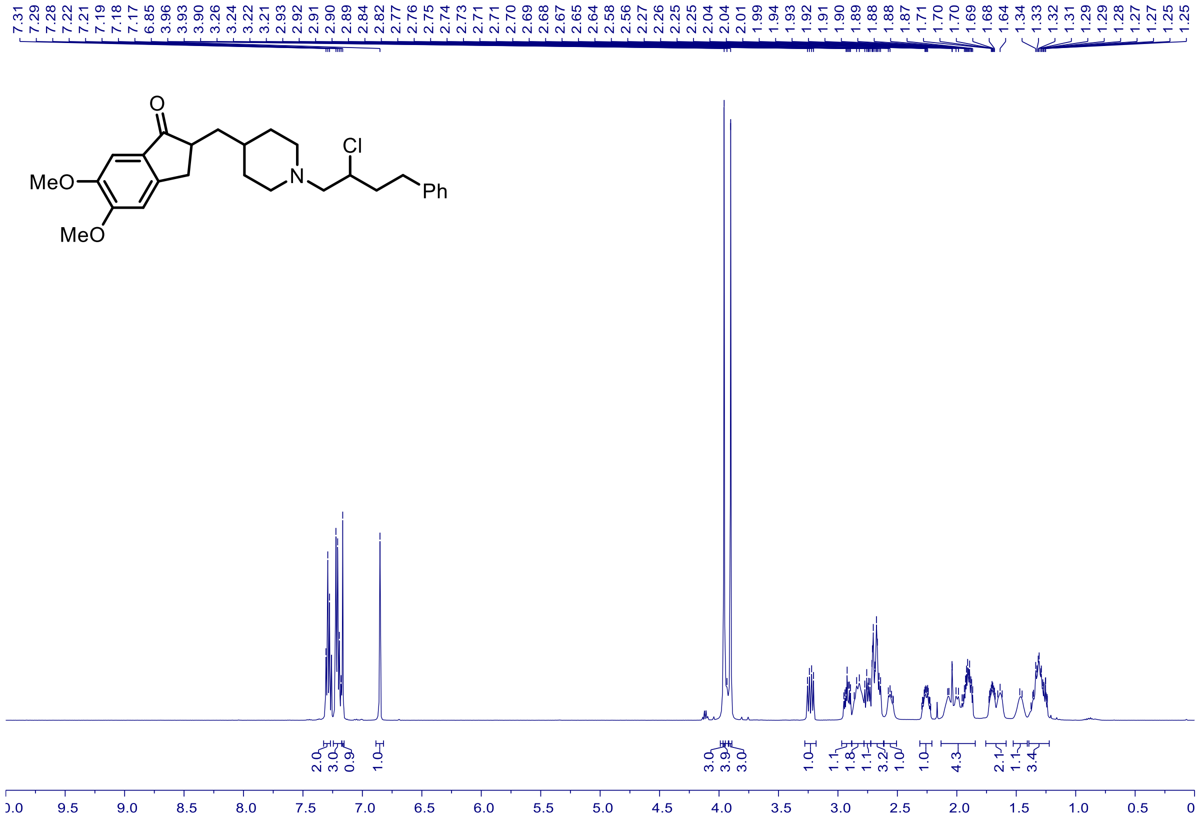

20 – ^{13}C NMR (126 MHz, CDCl3)

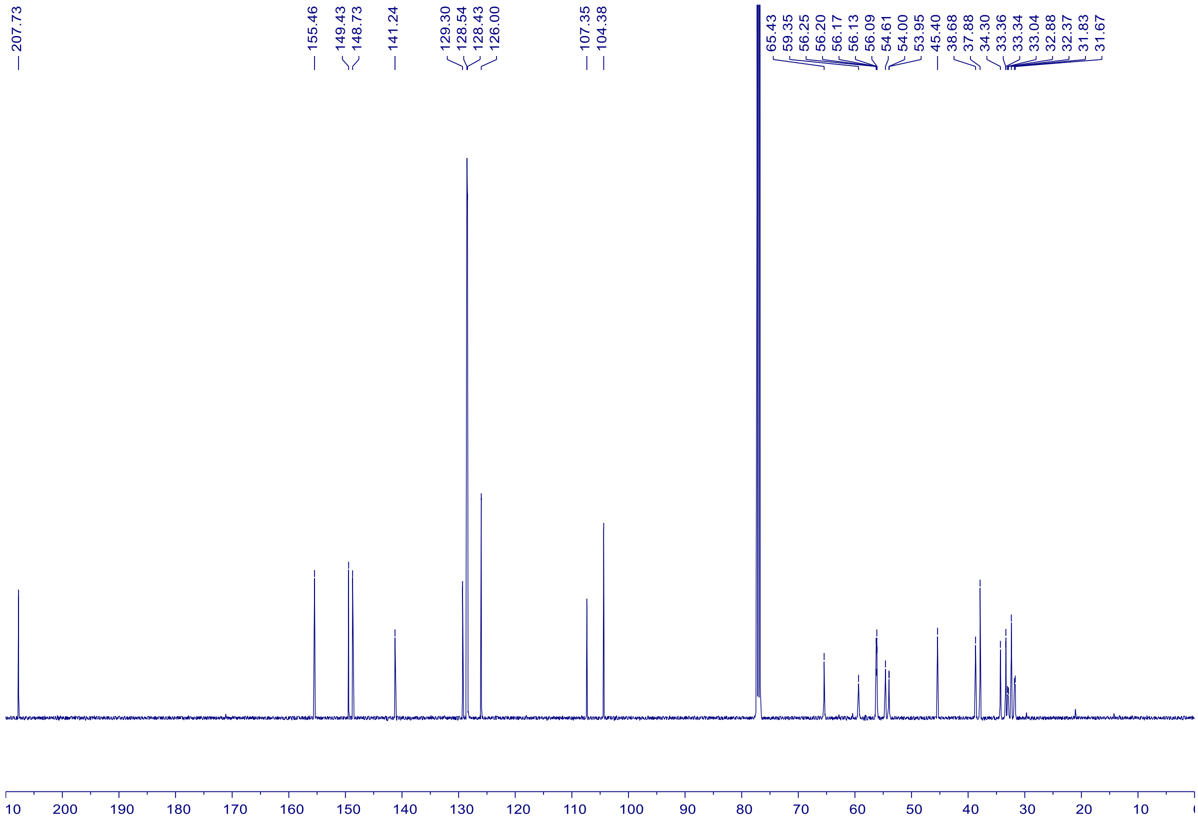

21 – ^{1}H NMR (500 MHz, $CDCl_3$)

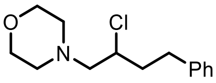

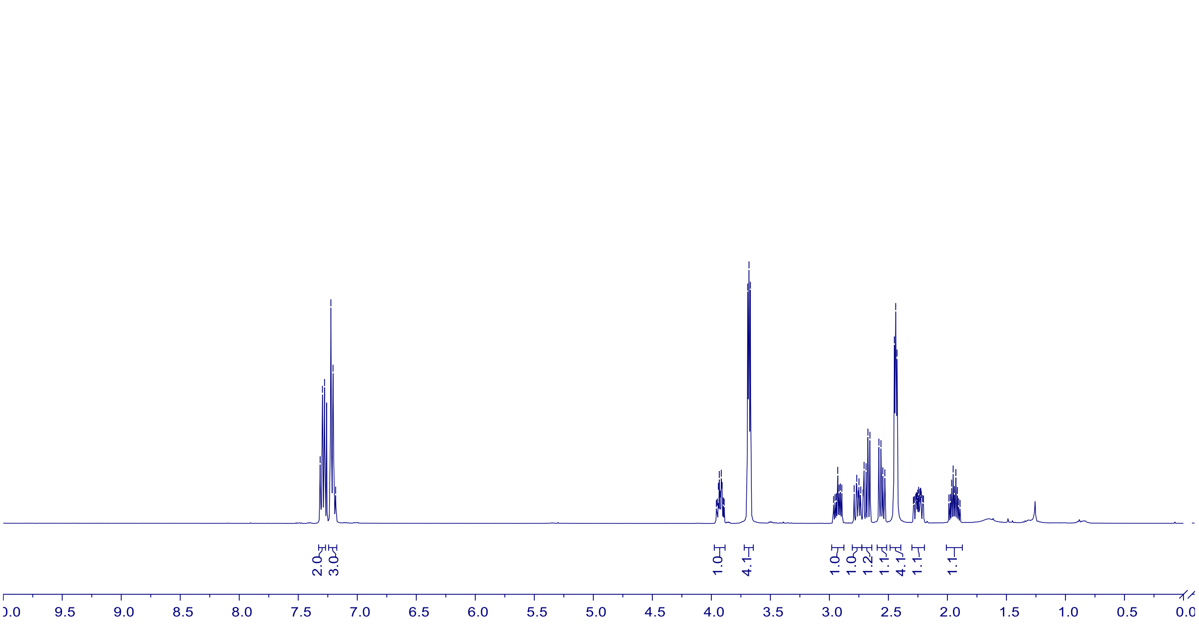

21 – ^{13}C NMR (126 MHz, $CDCl_3$)

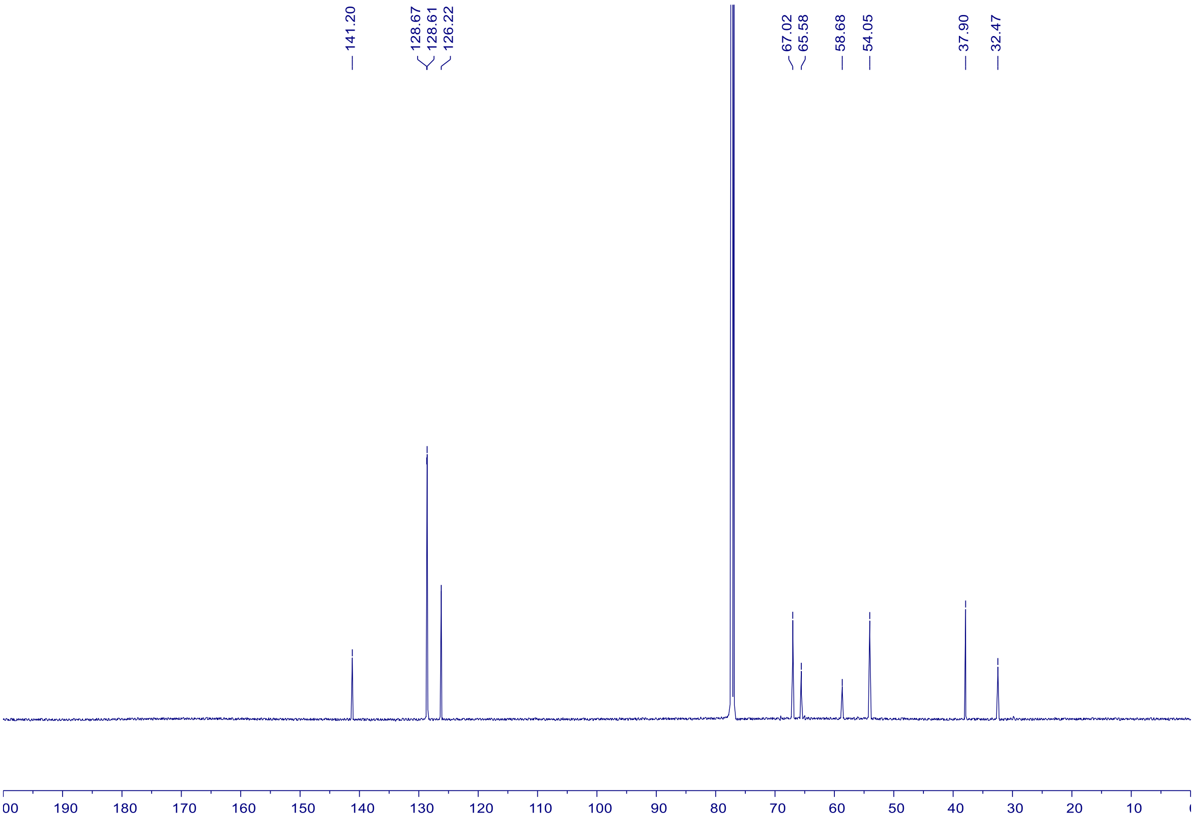

22 – ^{1}H NMR (500 MHz, $CDCl_3$)

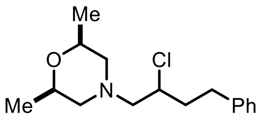

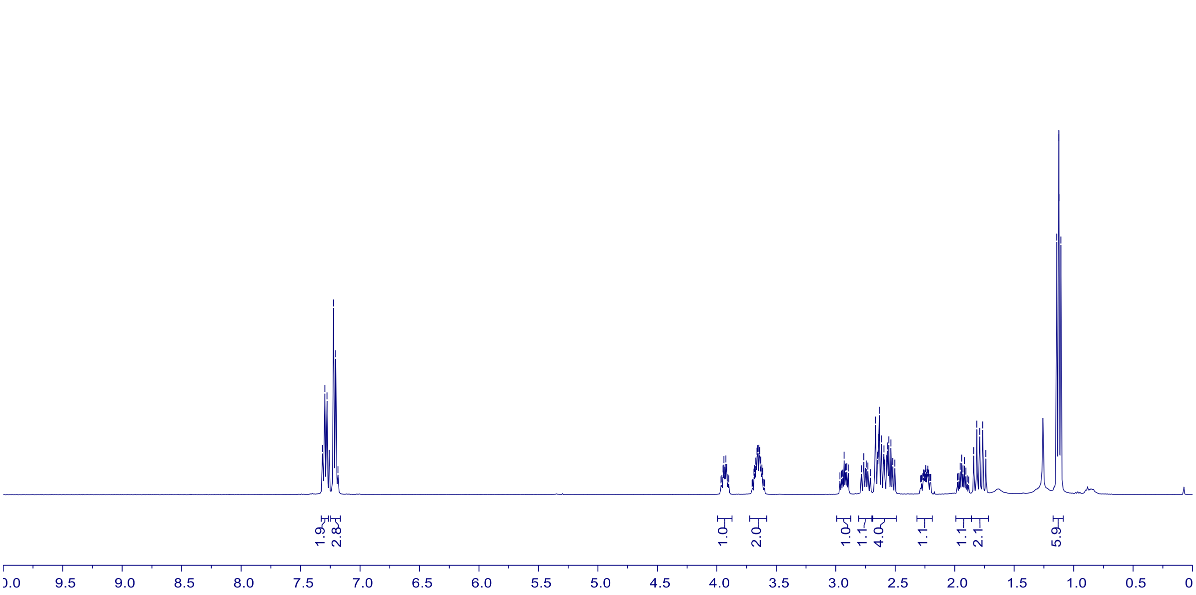

22 – ^{13}C NMR (126 MHz, $CDCl_3$)

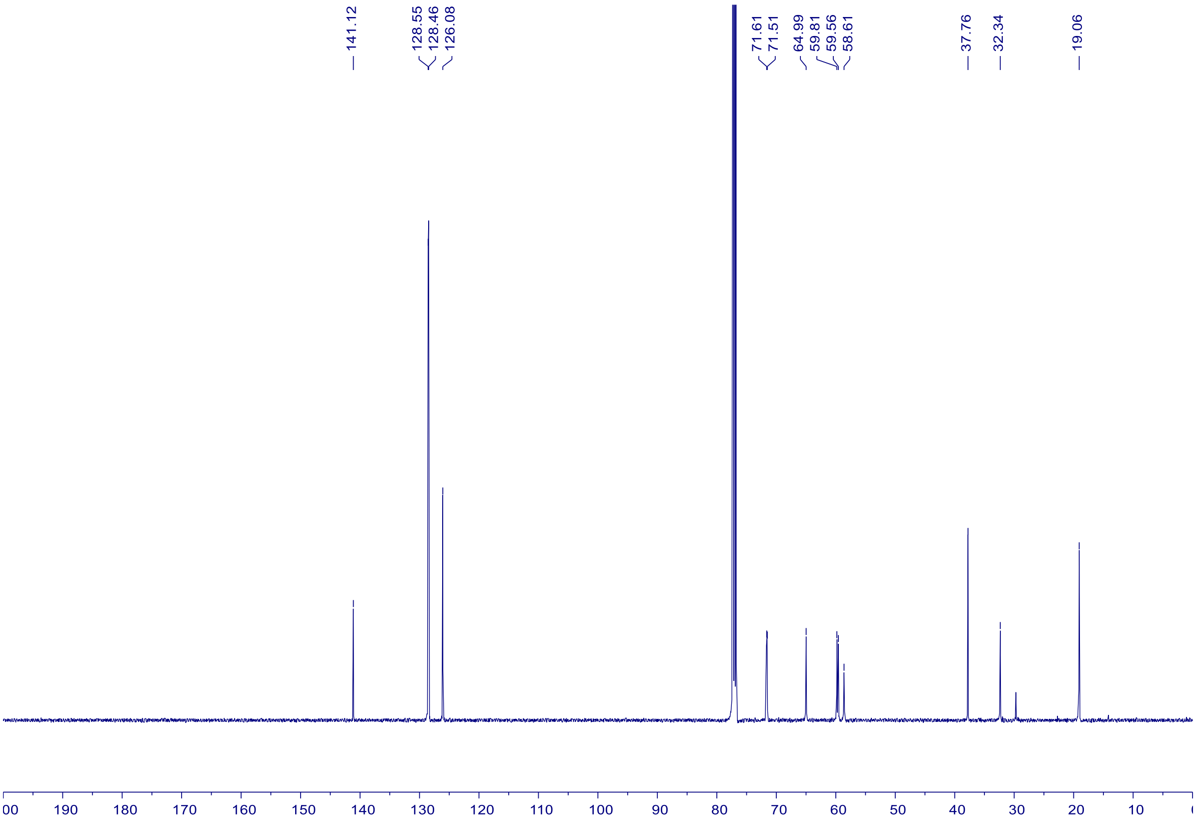

23 – ^{1}H NMR (500 MHz, CDCl$_3$)

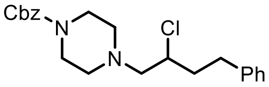

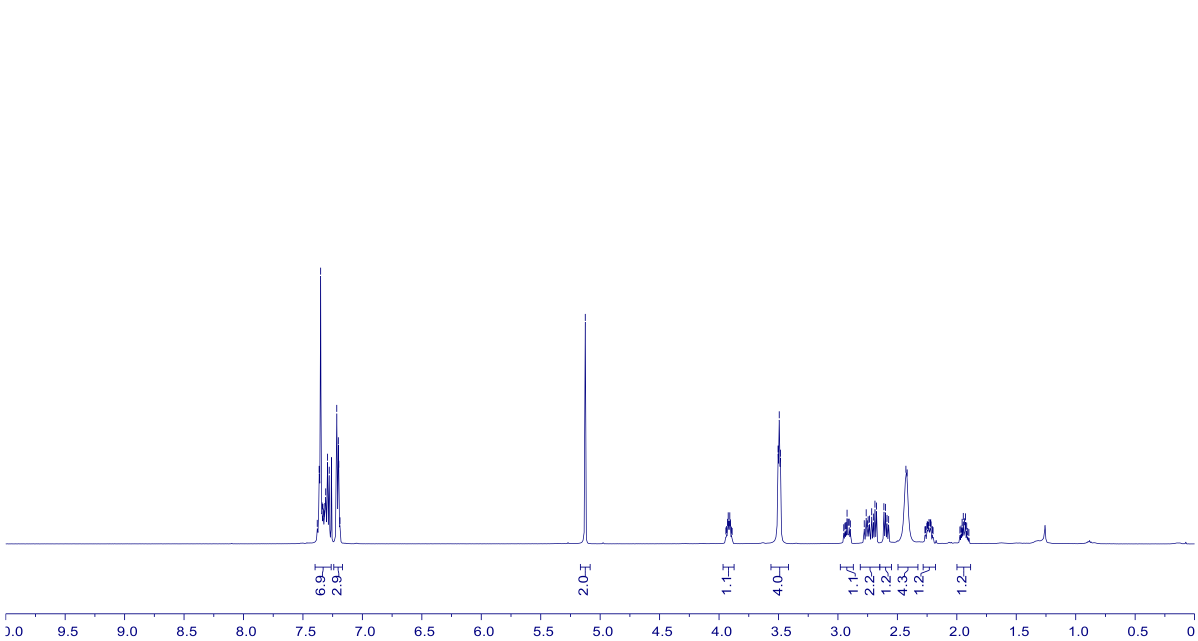

23 – ^{13}C NMR (126 MHz, CDCl$_3$)

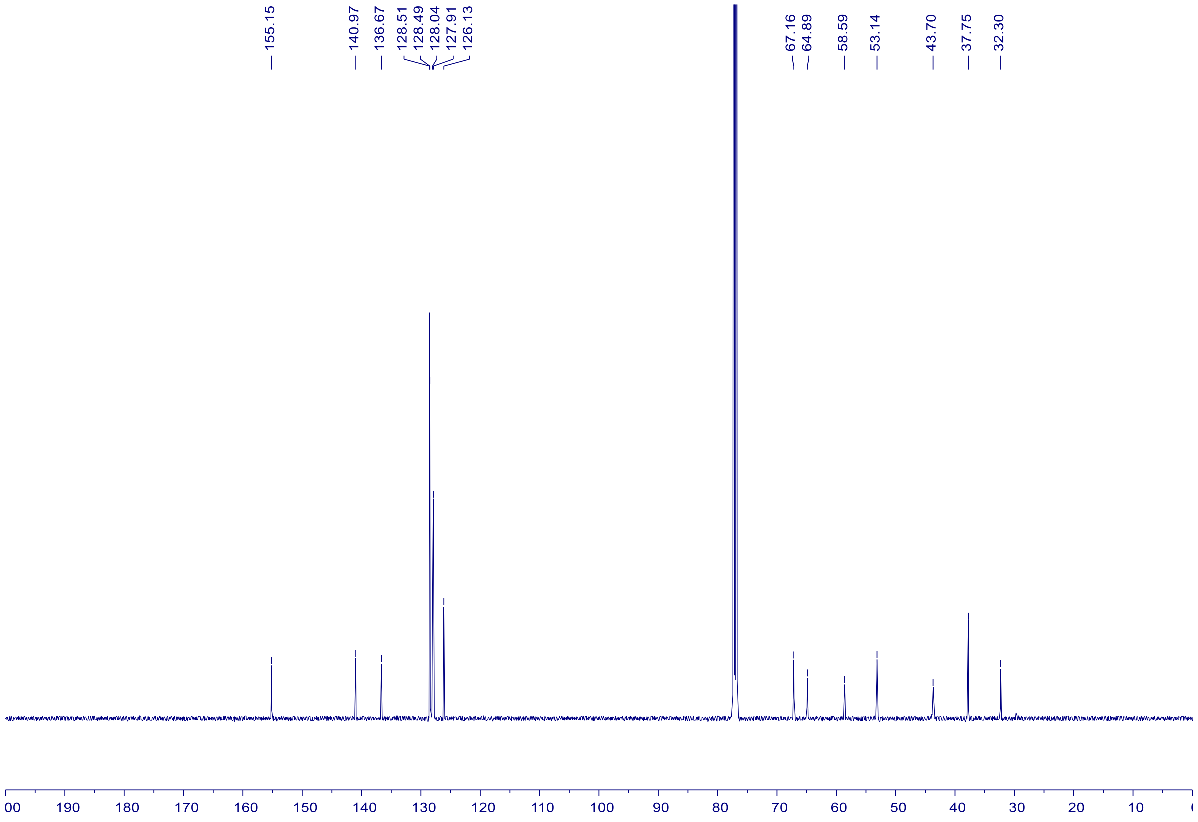

25 – ^{1}H NMR (500 MHz, $CDCl_3$)

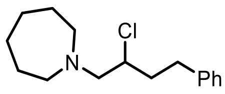

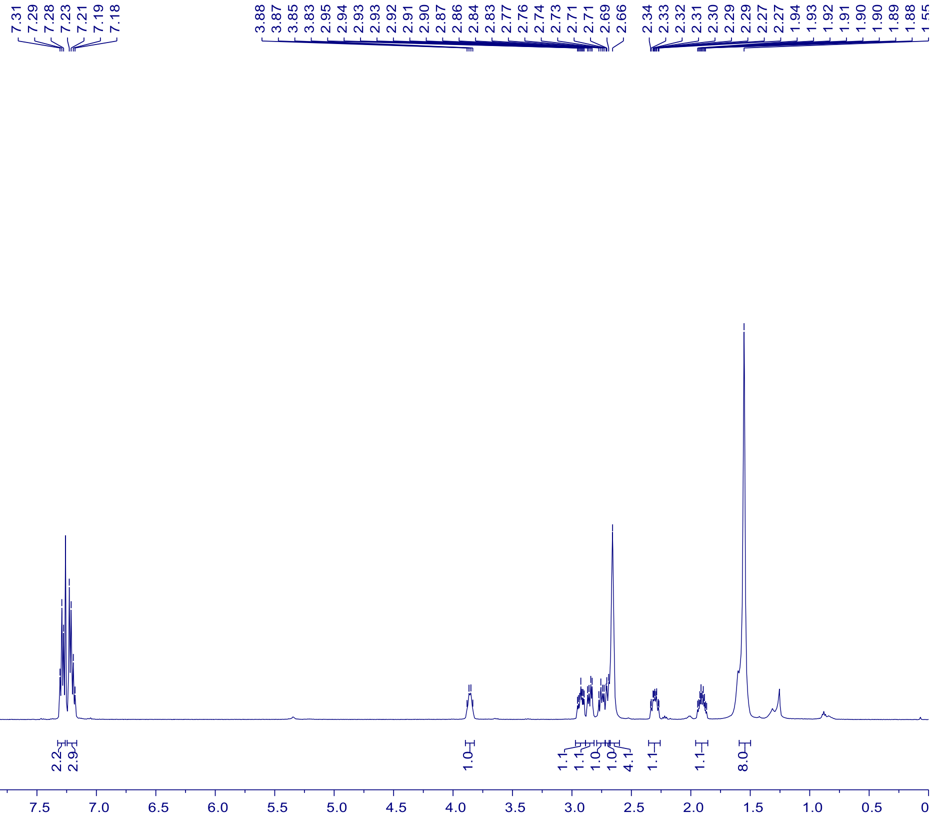

25 – ^{13}C NMR (126 MHz, $CDCl_3$)

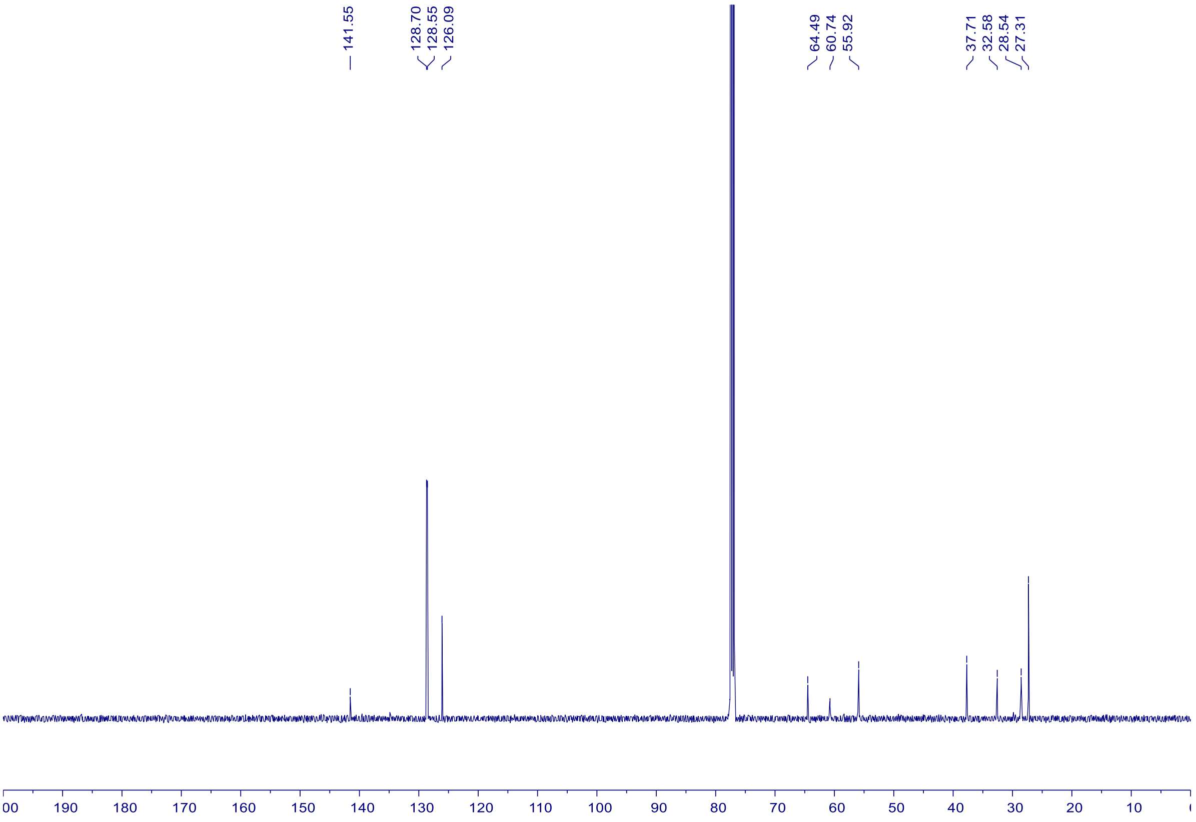

26 – ^{1}H NMR (500 MHz, $CDCl_3$)

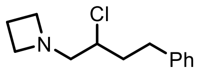

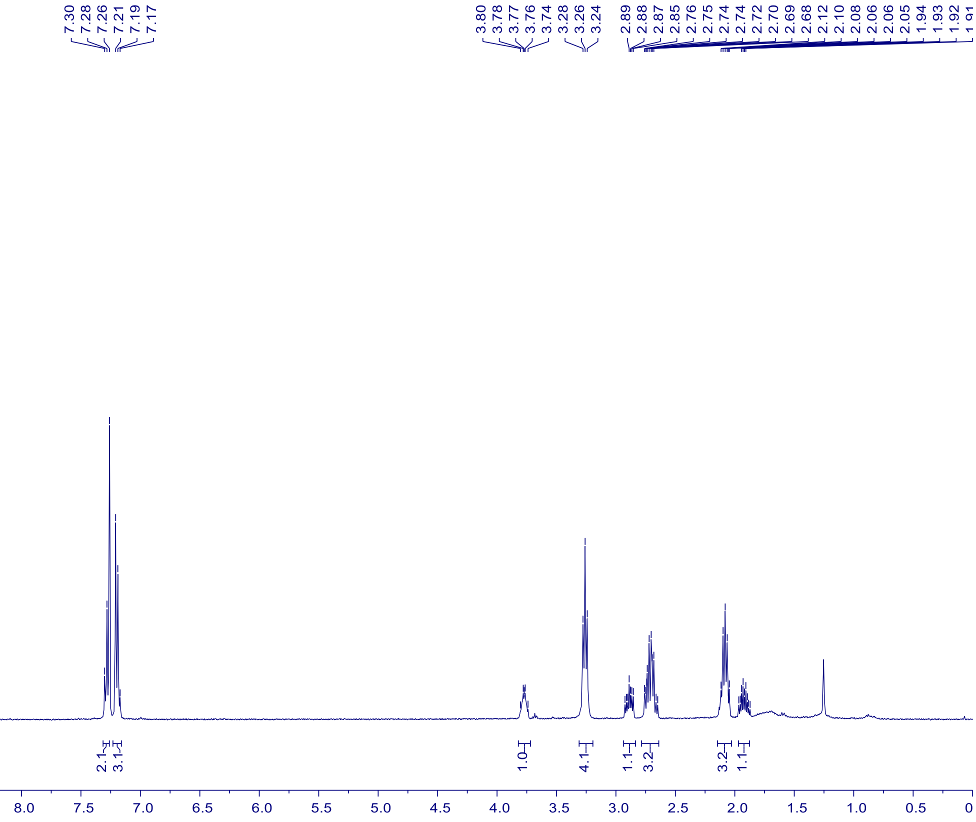

26 – ^{13}C NMR (126 MHz, $CDCl_3$)

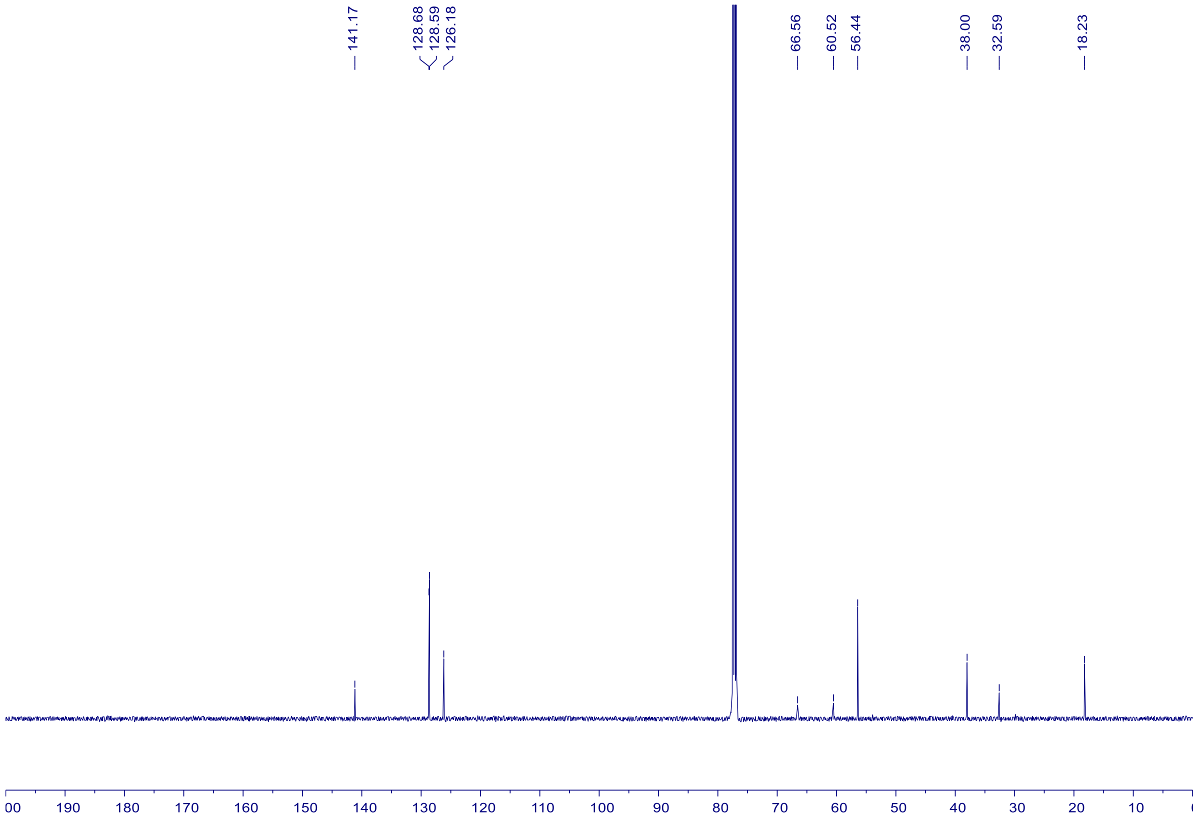

27 – ^{1}H NMR (500 MHz, $CDCl_3$)

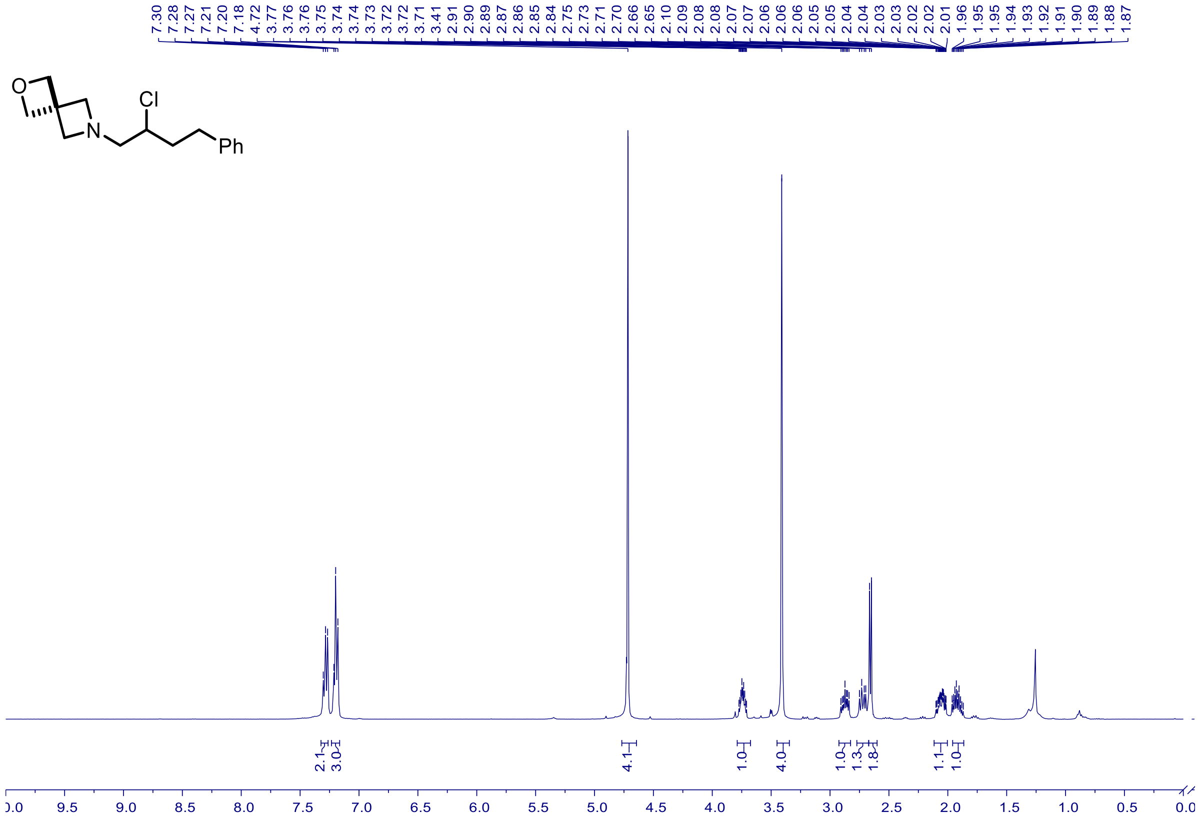

27 – ^{13}C NMR (126 MHz, $CDCl_3$)

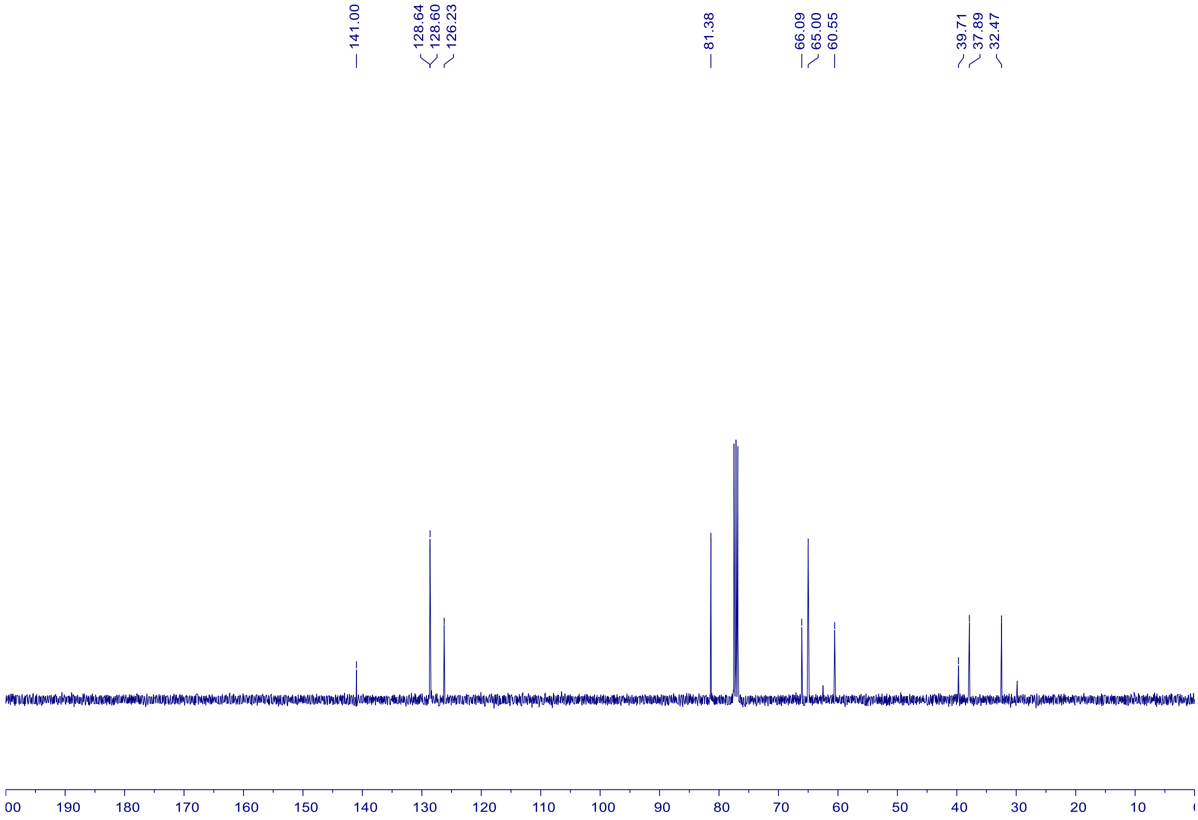

28 – ^{1}H NMR (500 MHz, $CDCl_3$)

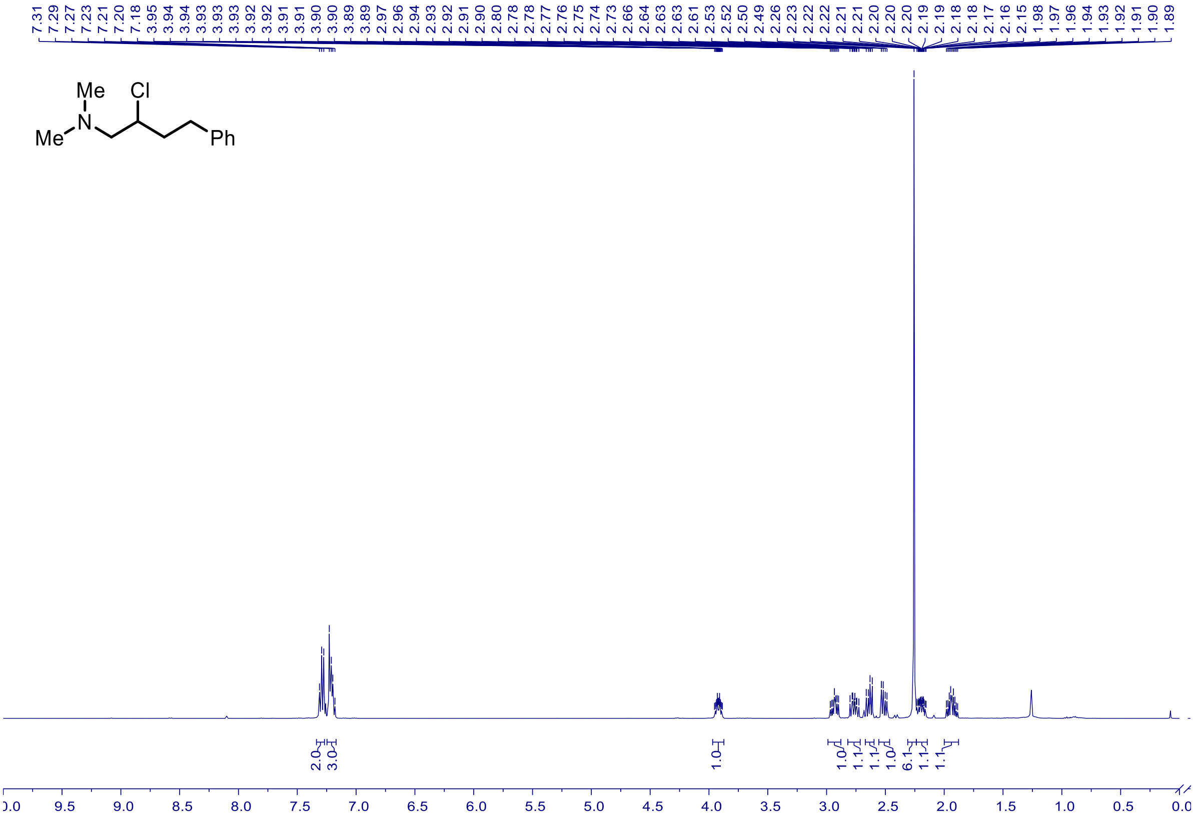

28 – ^{13}C NMR (126 MHz, $CDCl_3$)

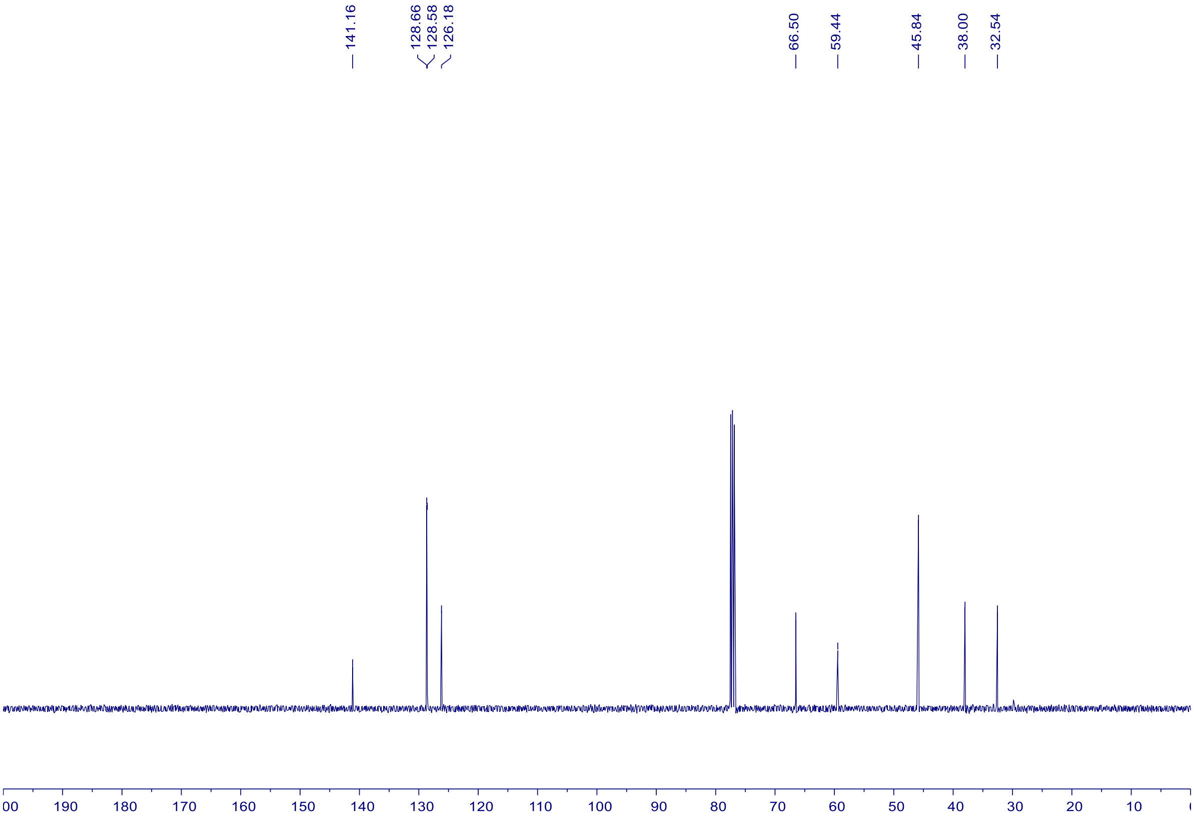

29 – ^{1}H NMR (500 MHz, $CDCl_3$)

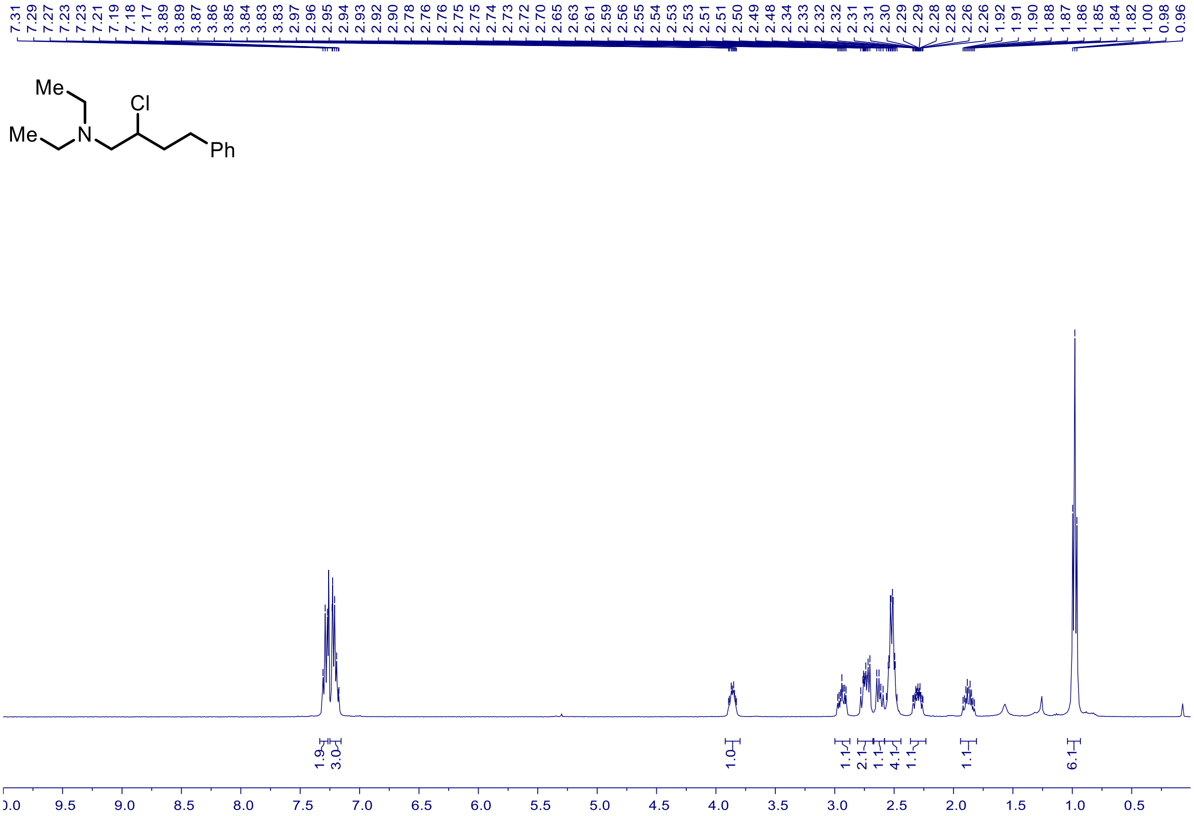

29 – ^{13}C NMR (126 MHz, $CDCl_3$)

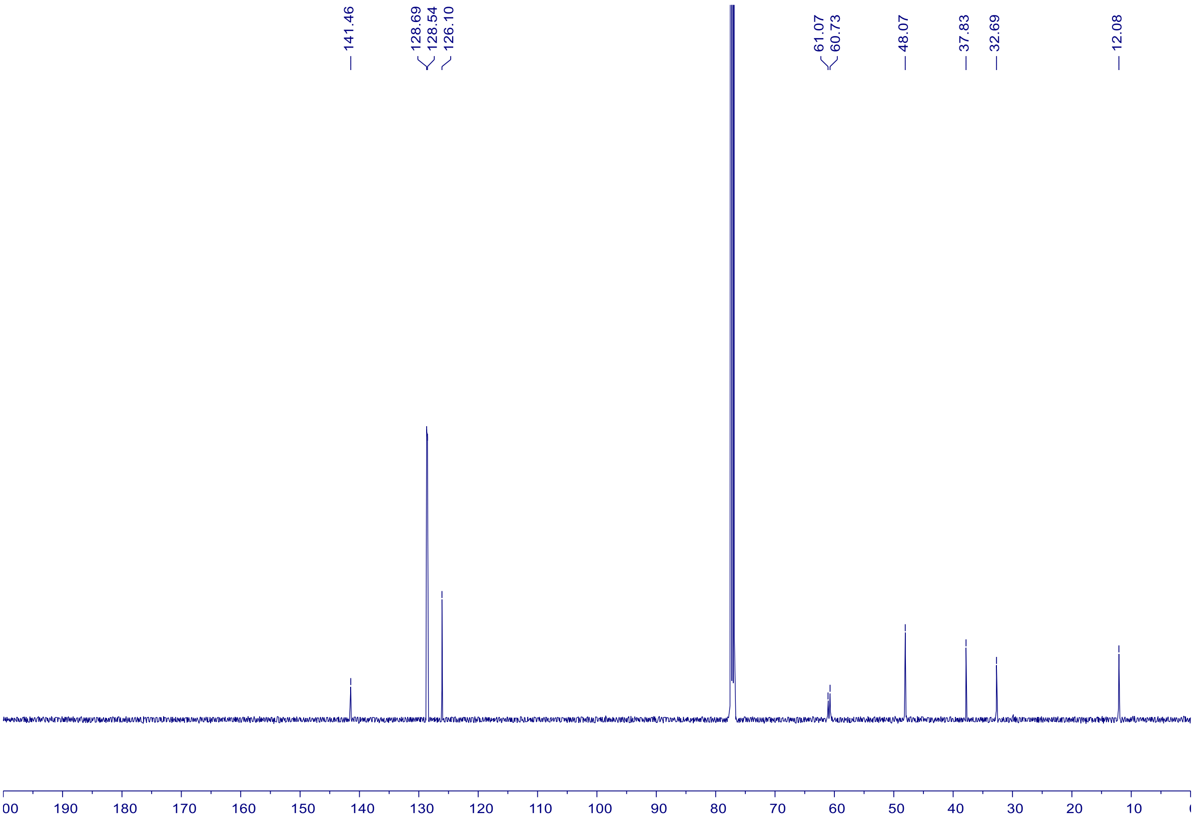

30 – ^{1}H NMR (500 MHz, CDCl$_3$)

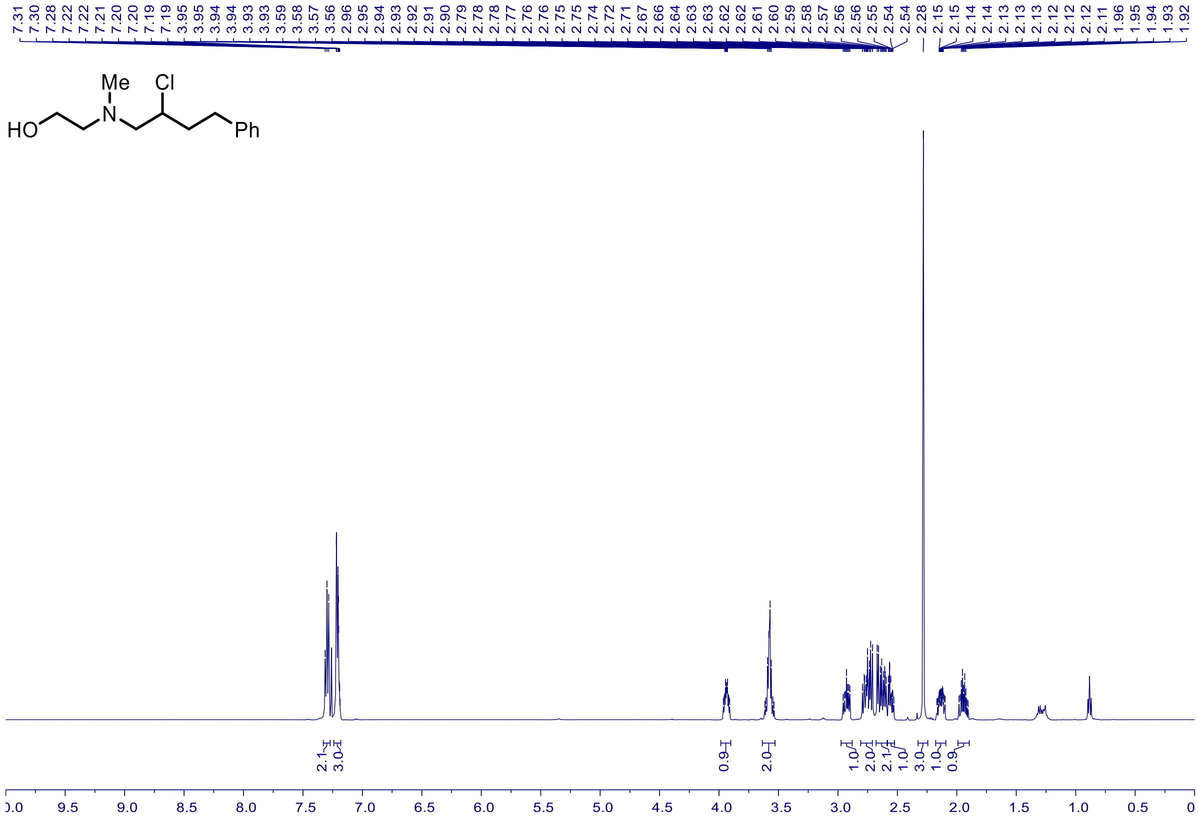

30 – ^{13}C NMR (126 MHz, CDCl$_3$)

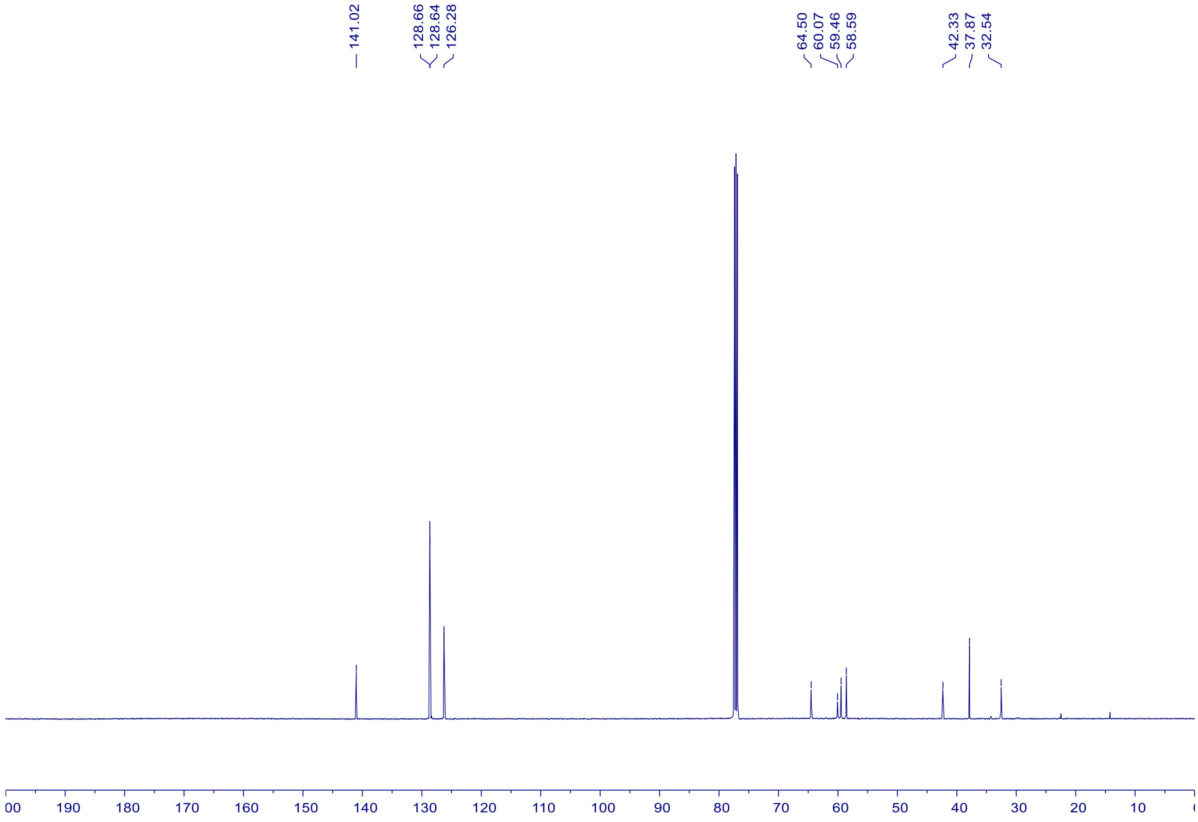

31 – ¹H NMR (500 MHz, $CDCl_3$)

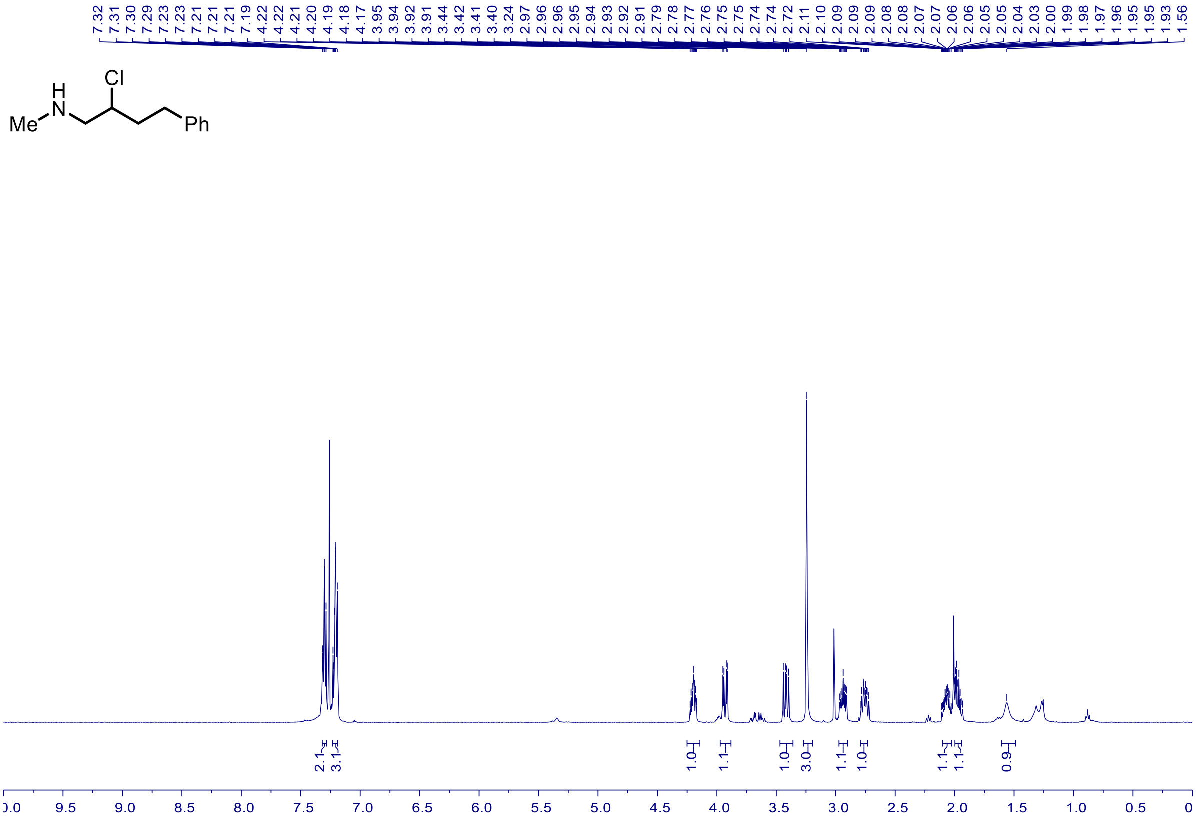

31 – ¹³C NMR (126 MHz, $CDCl_3$)

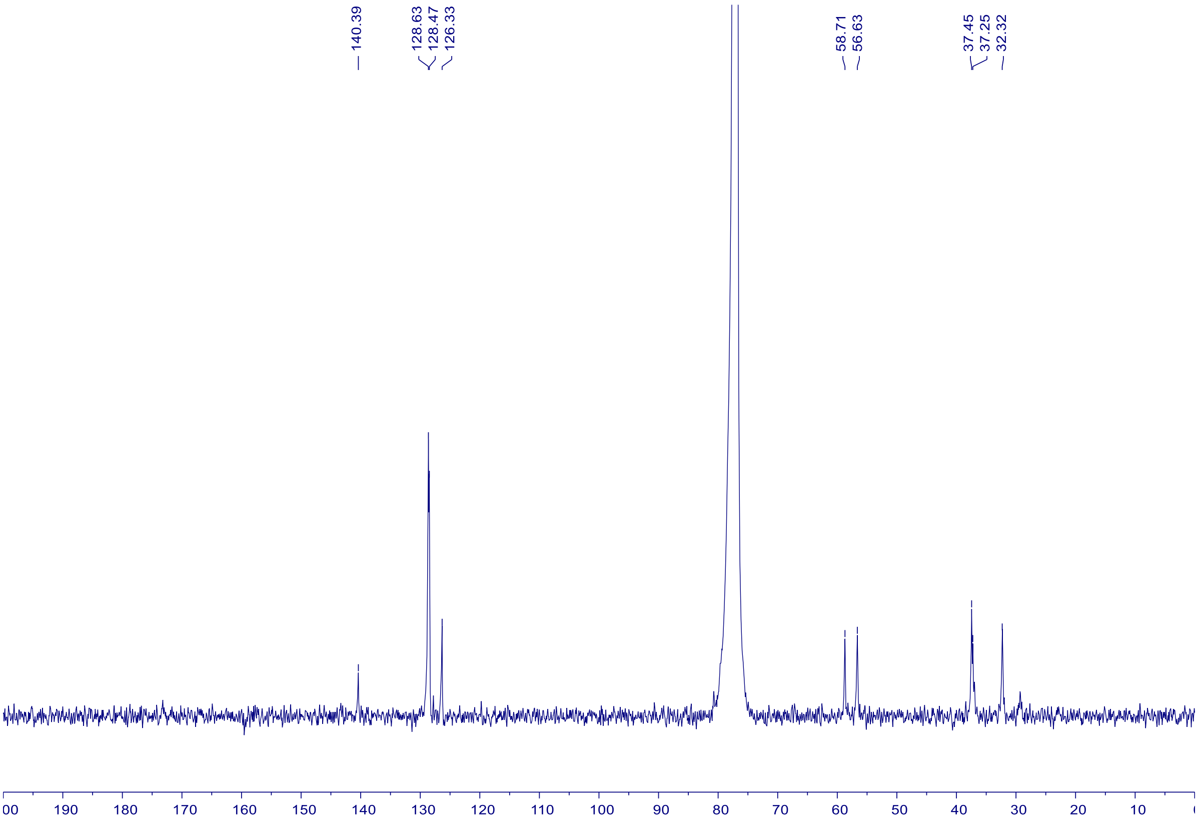

32 – ^{1}H NMR (500 MHz, $CDCl_3$)

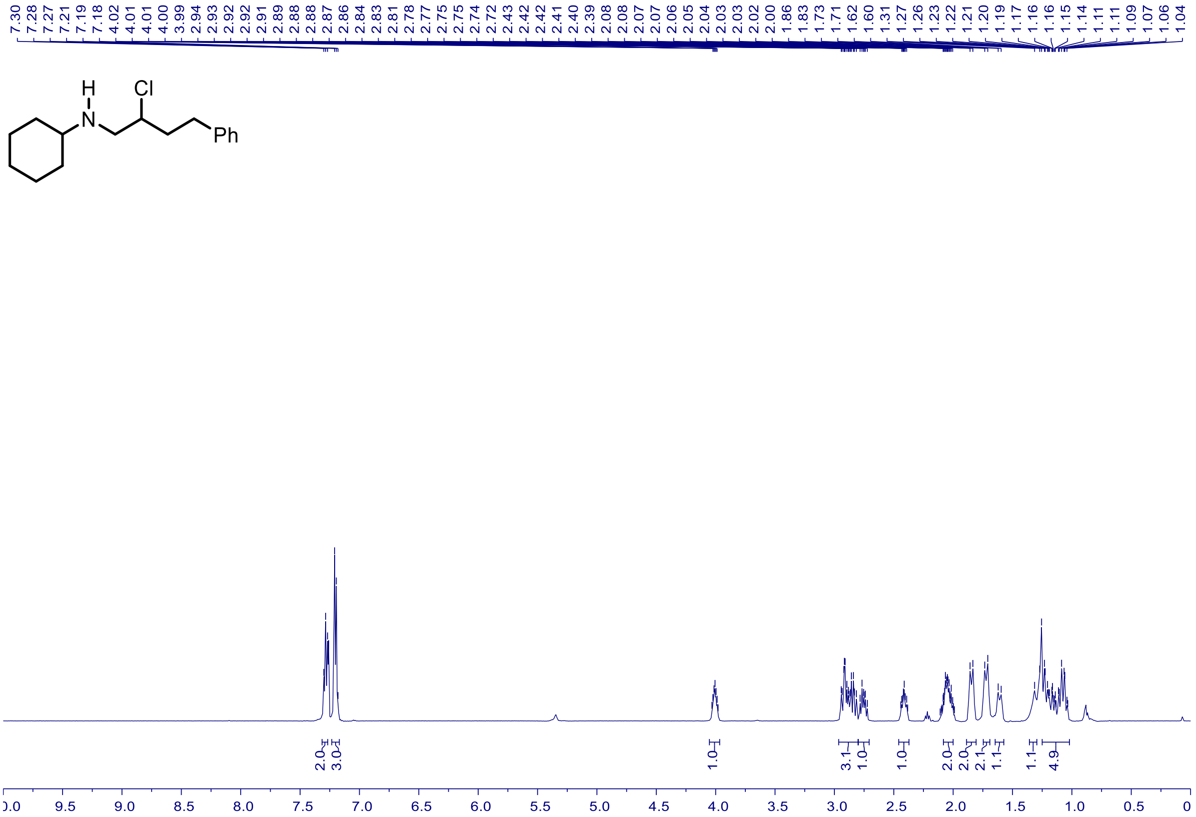

32 – ^{13}C NMR (126 MHz, $CDCl_3$)

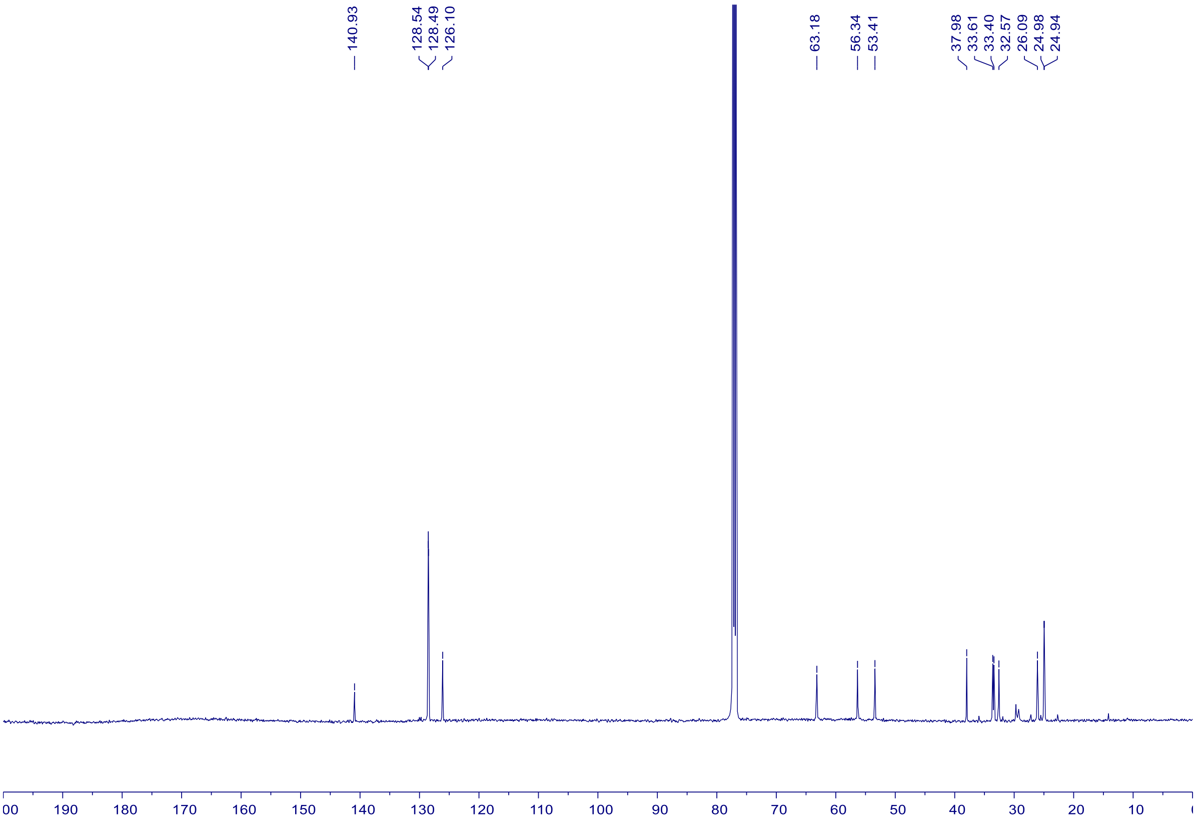

33 – ^{1}H NMR (500 MHz, $CDCl_3$)

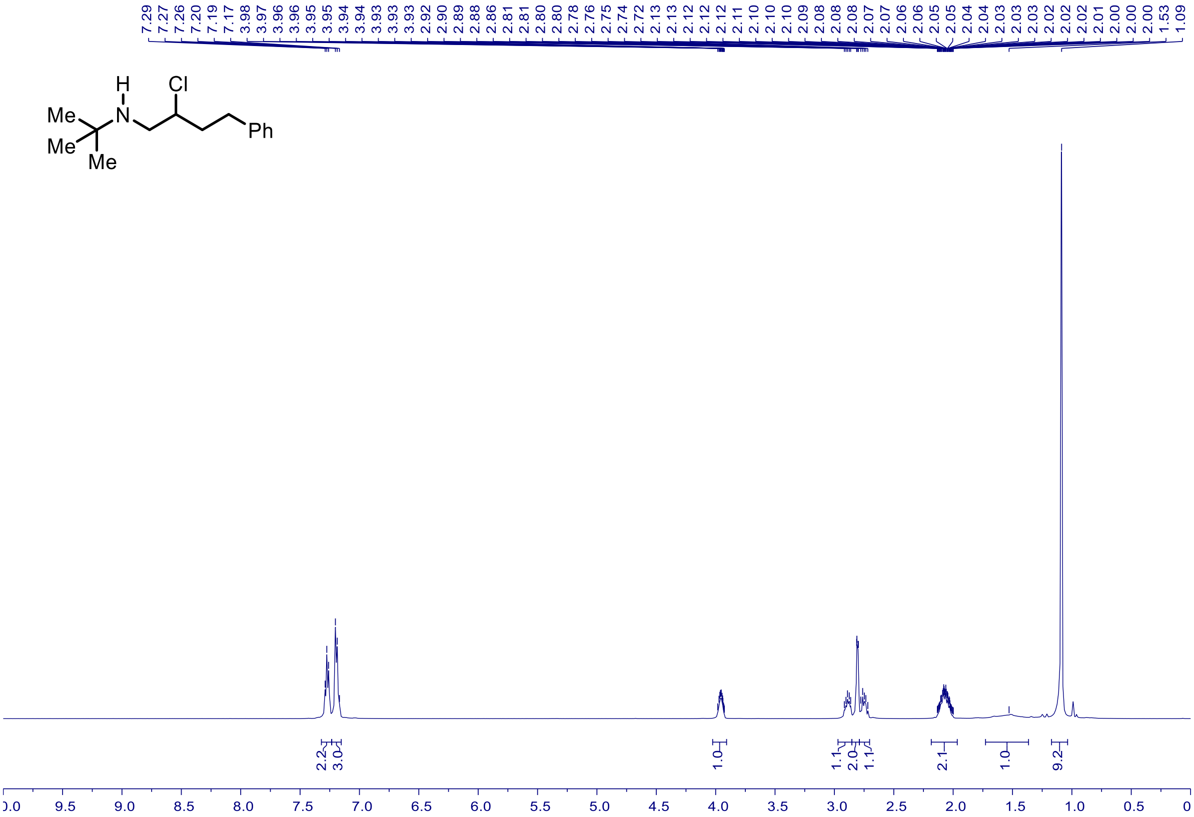

33 – ^{13}C NMR (126 MHz, $CDCl_3$)

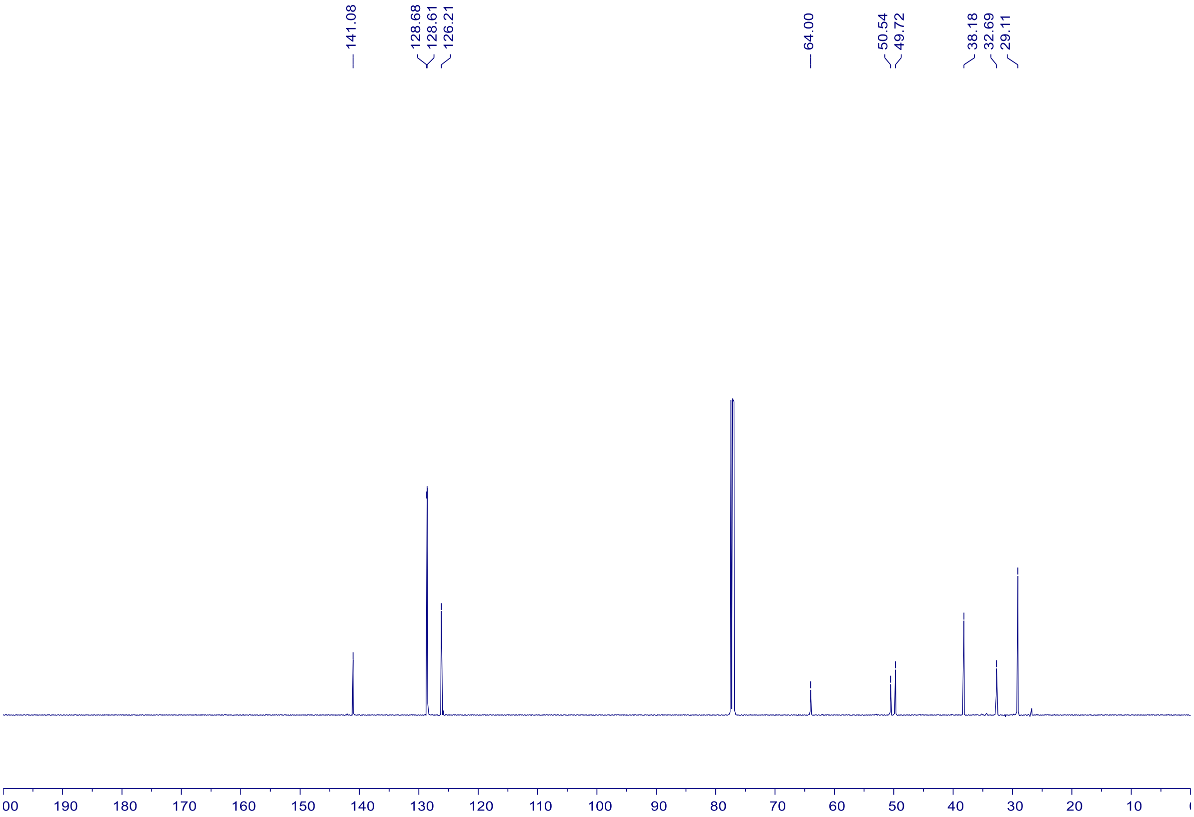

34 – ^{1}H NMR (500 MHz, $CDCl_3$)

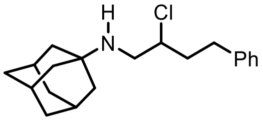

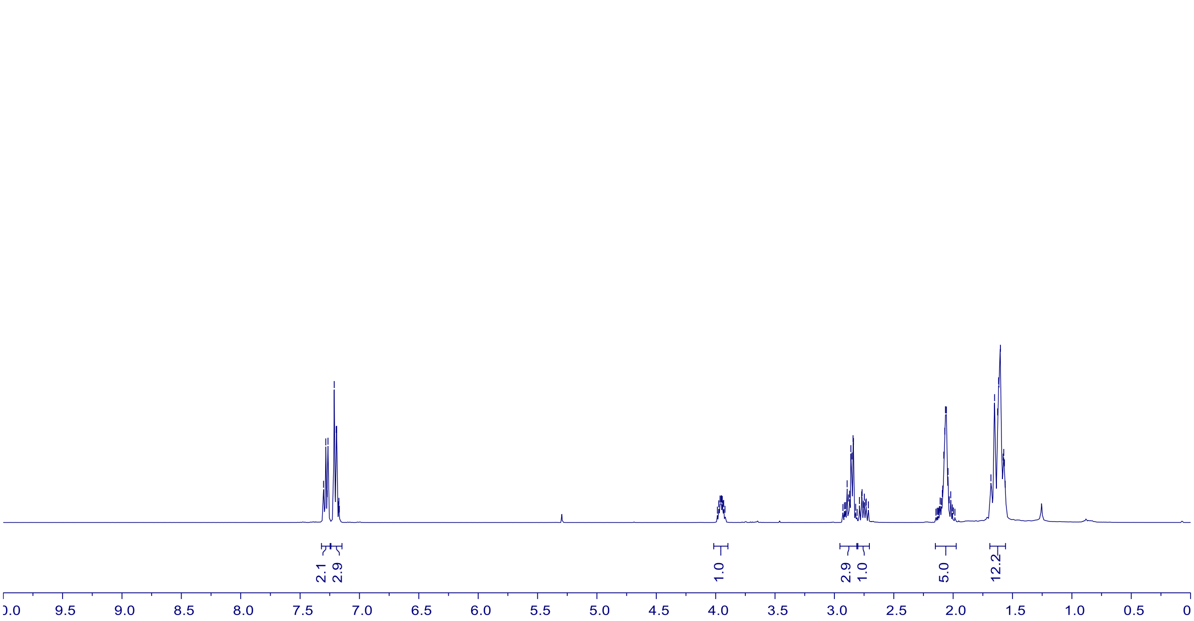

34 – ^{13}C NMR (126 MHz, $CDCl_3$)

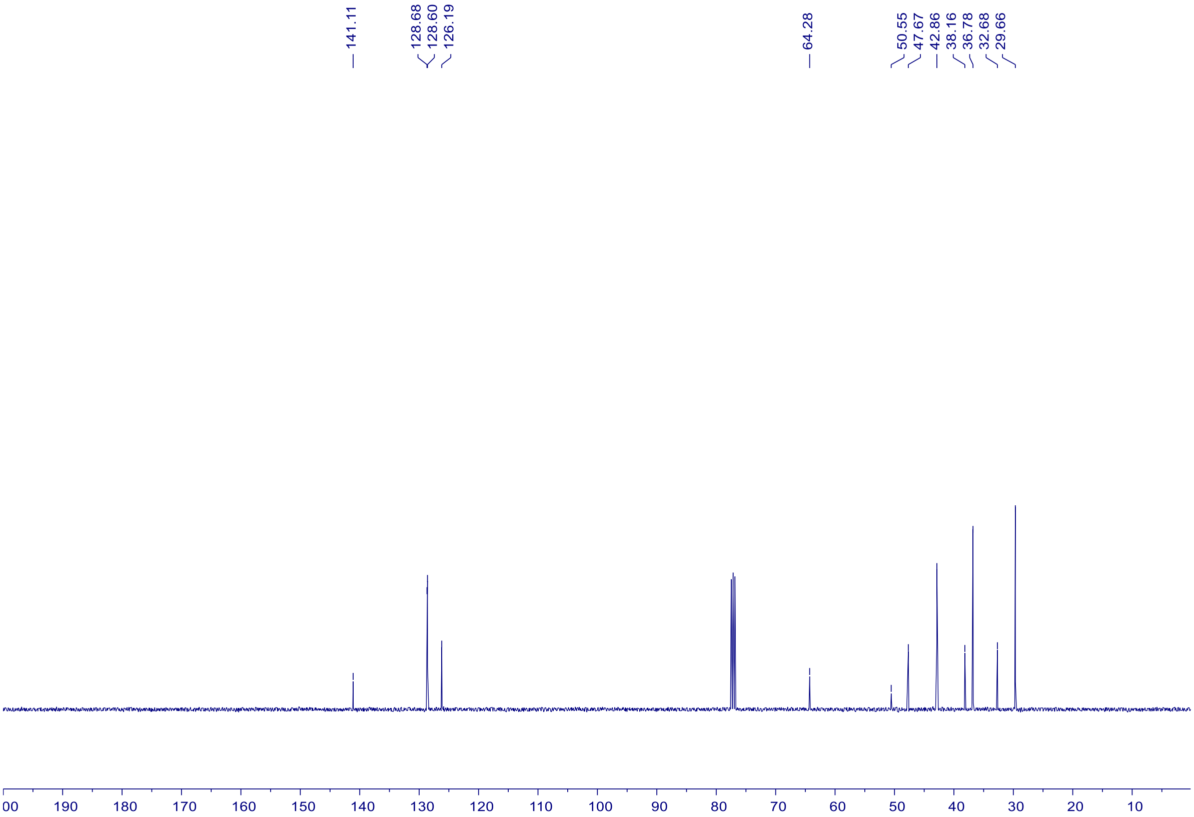

35 – ^{1}H NMR (500 MHz, CDCl$_3$, diastereomers)

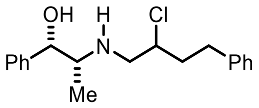

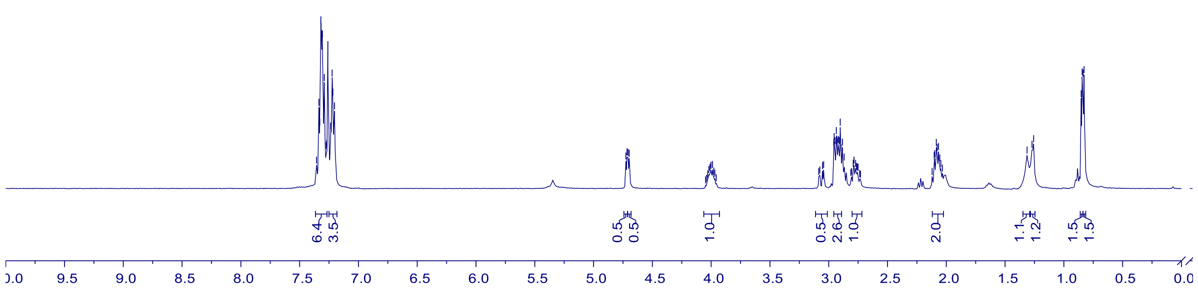

35 – ^{13}C NMR (126 MHz, CDCl$_3$)

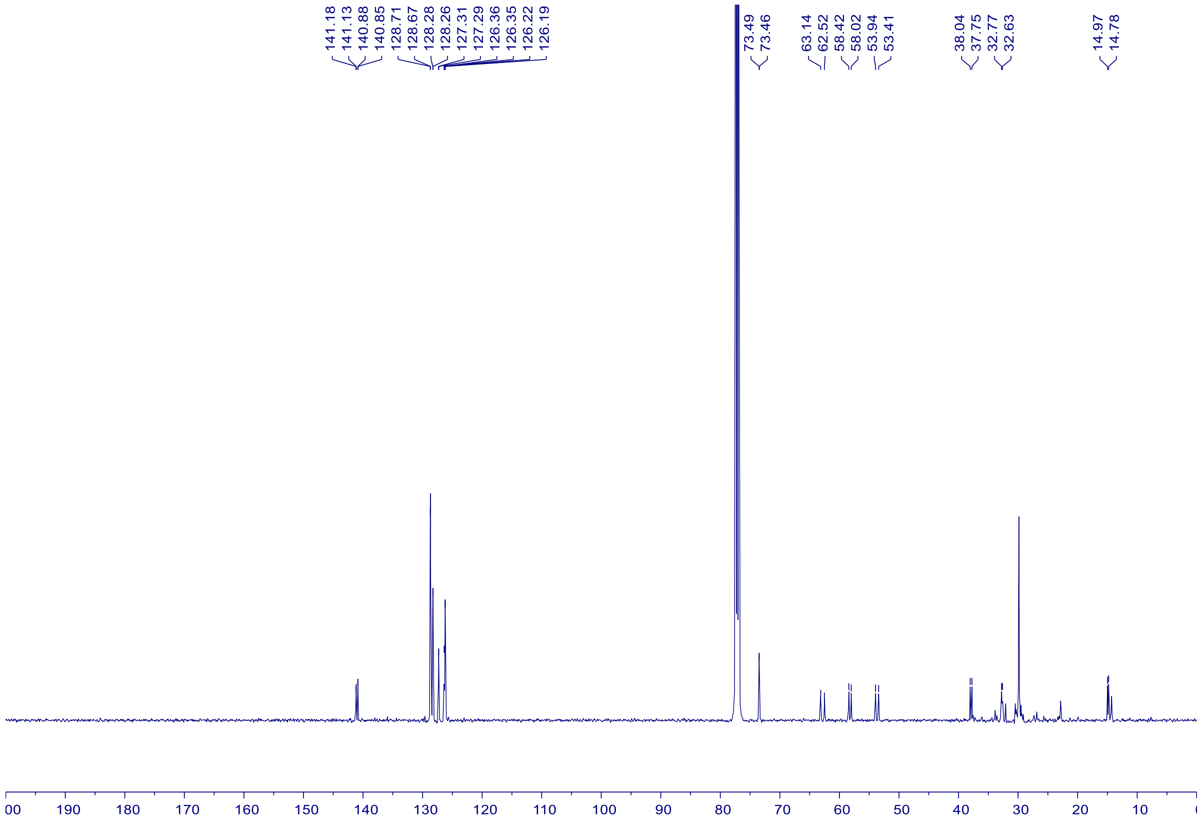

36 – ^{1}H NMR (500 MHz, $CDCl_3$, diastereomers)

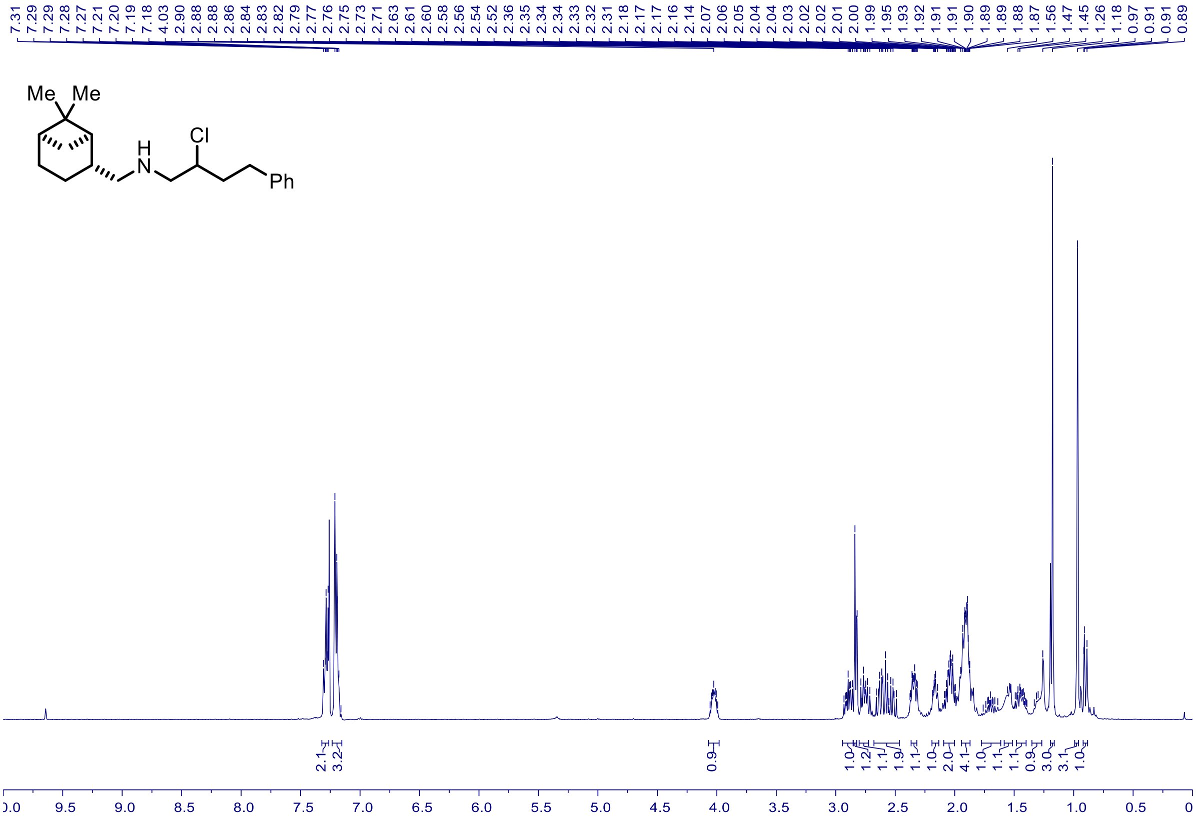

36 – ^{13}C NMR (126 MHz, $CDCl_3$)

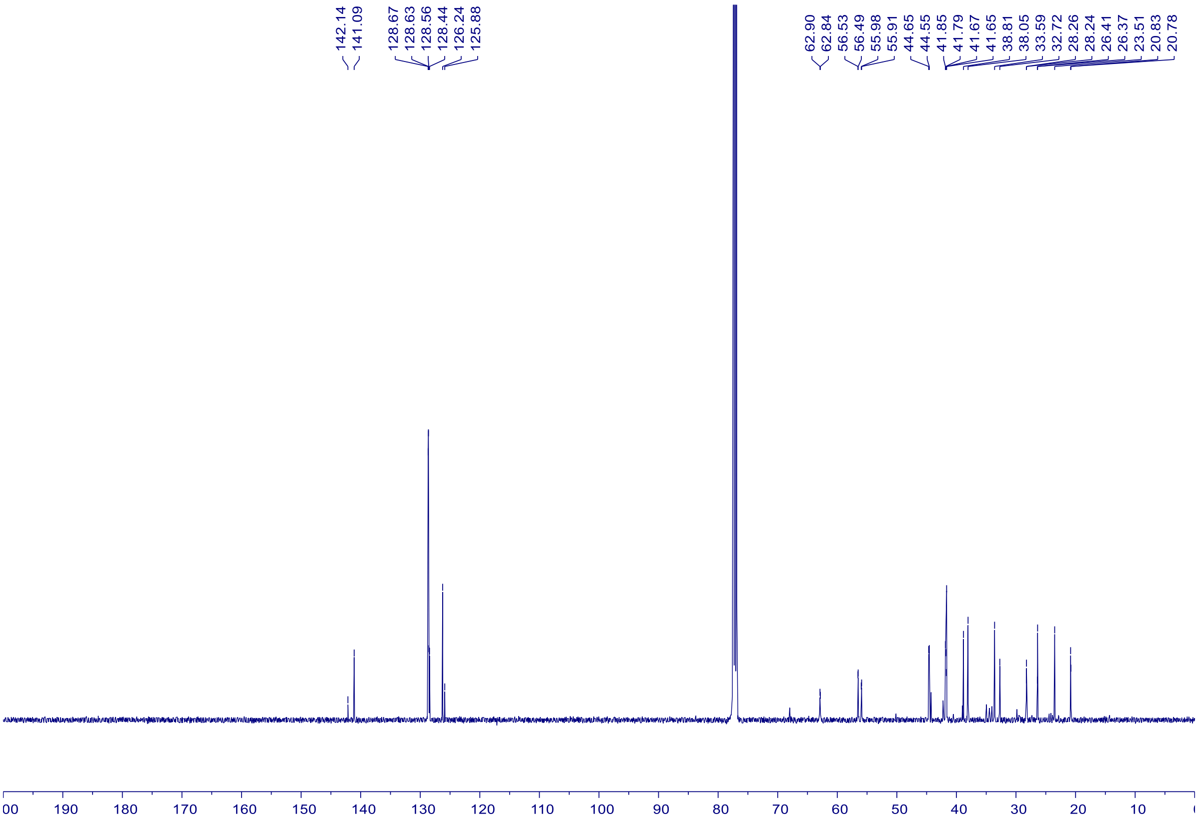

37 – ^{1}H NMR (500 MHz, $CDCl_3$)

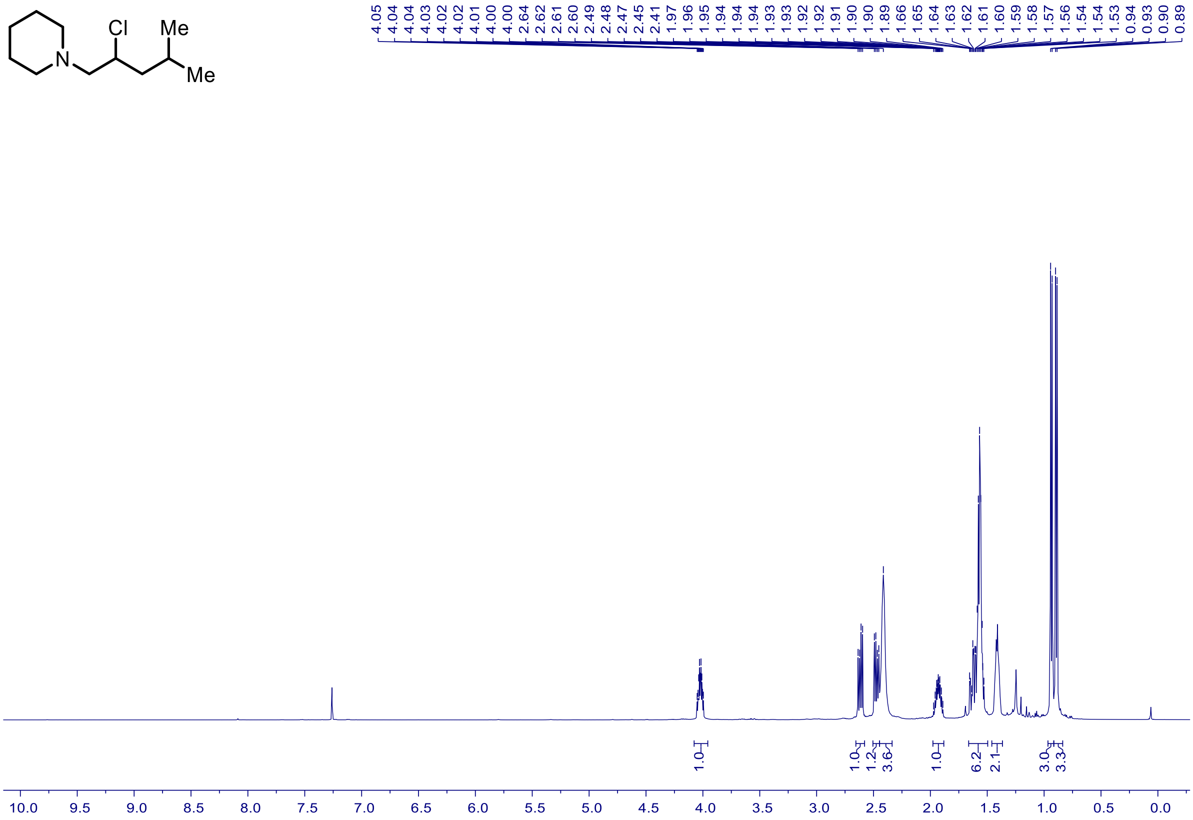

37 – ^{13}C NMR (125 MHz, $CDCl_3$)

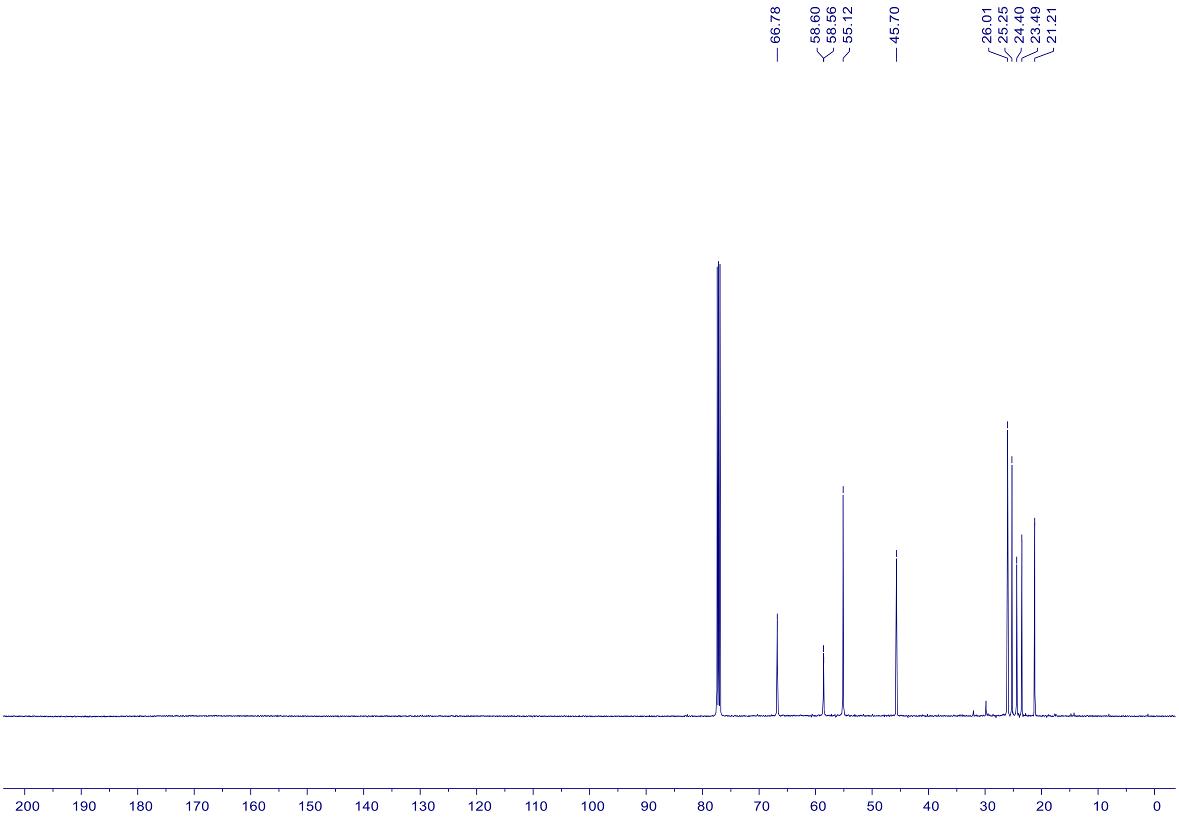

38 – ^{1}H NMR (400 MHz, $CDCl_3$)

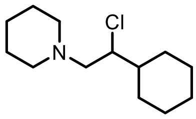

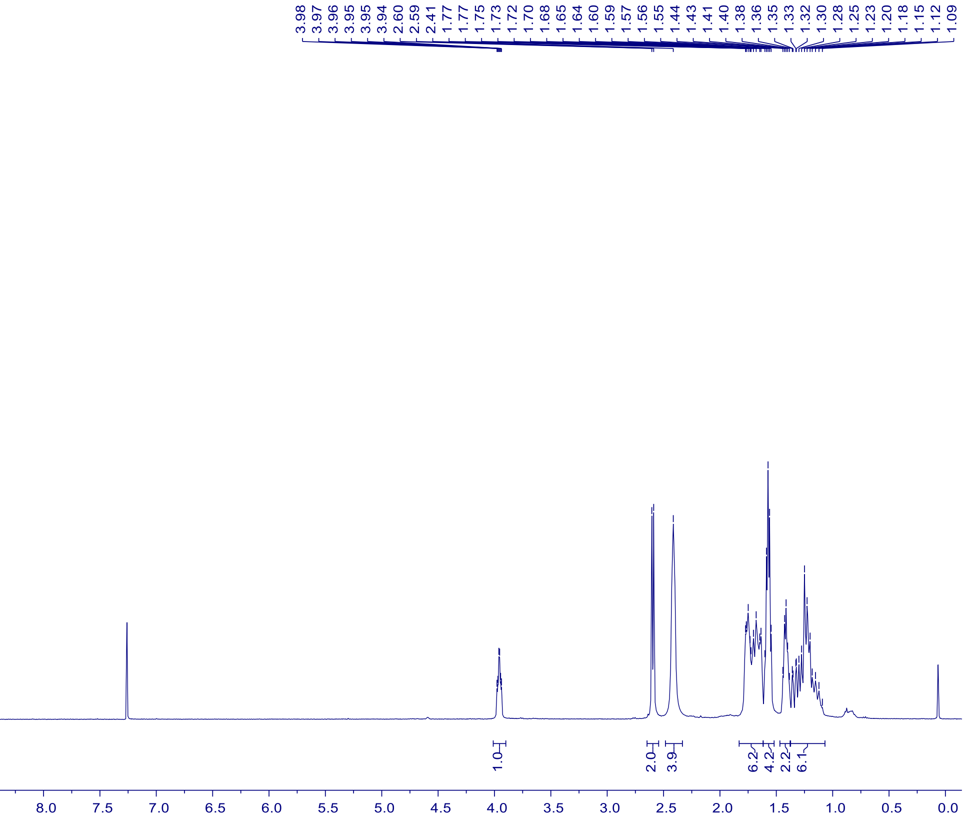

38 – ^{13}C NMR (100 MHz, $CDCl_3$)

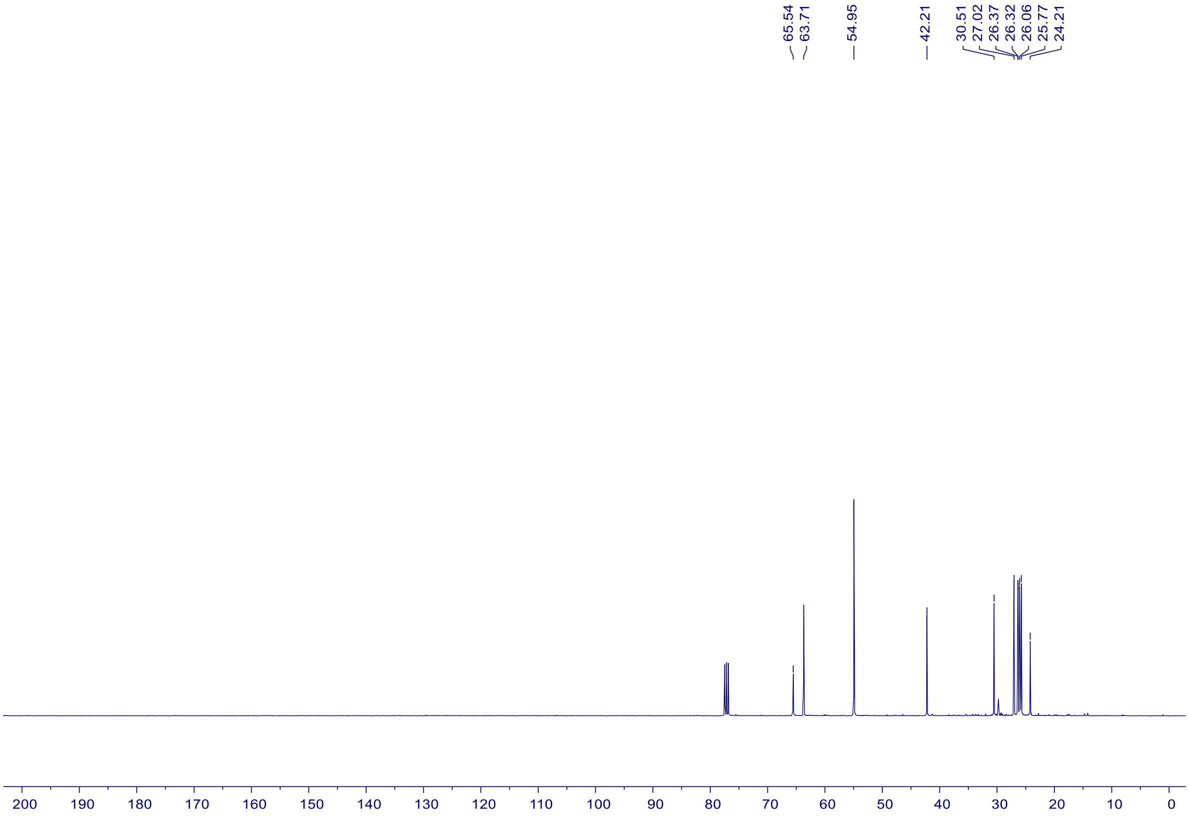

39 – ^{1}H NMR (500 MHz, $CDCl_3$)

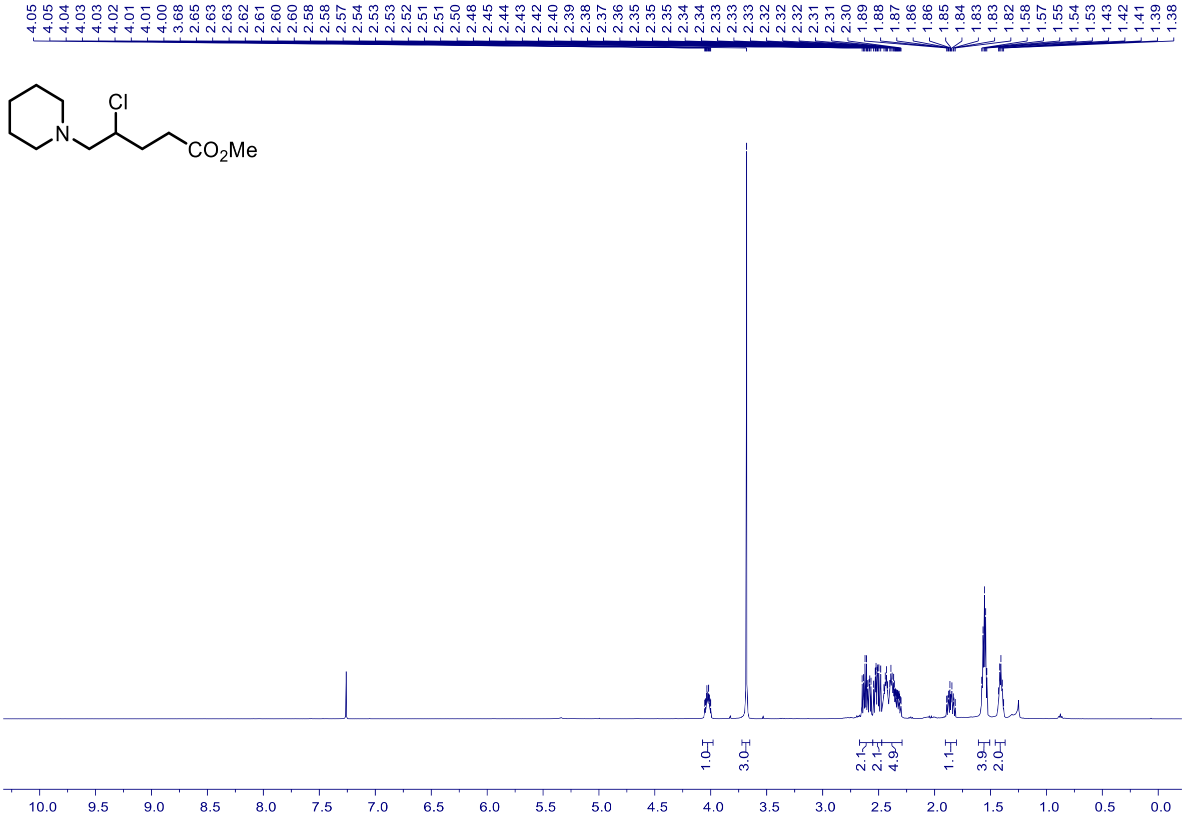

39 – ^{13}C NMR (125 MHz, $CDCl_3$)

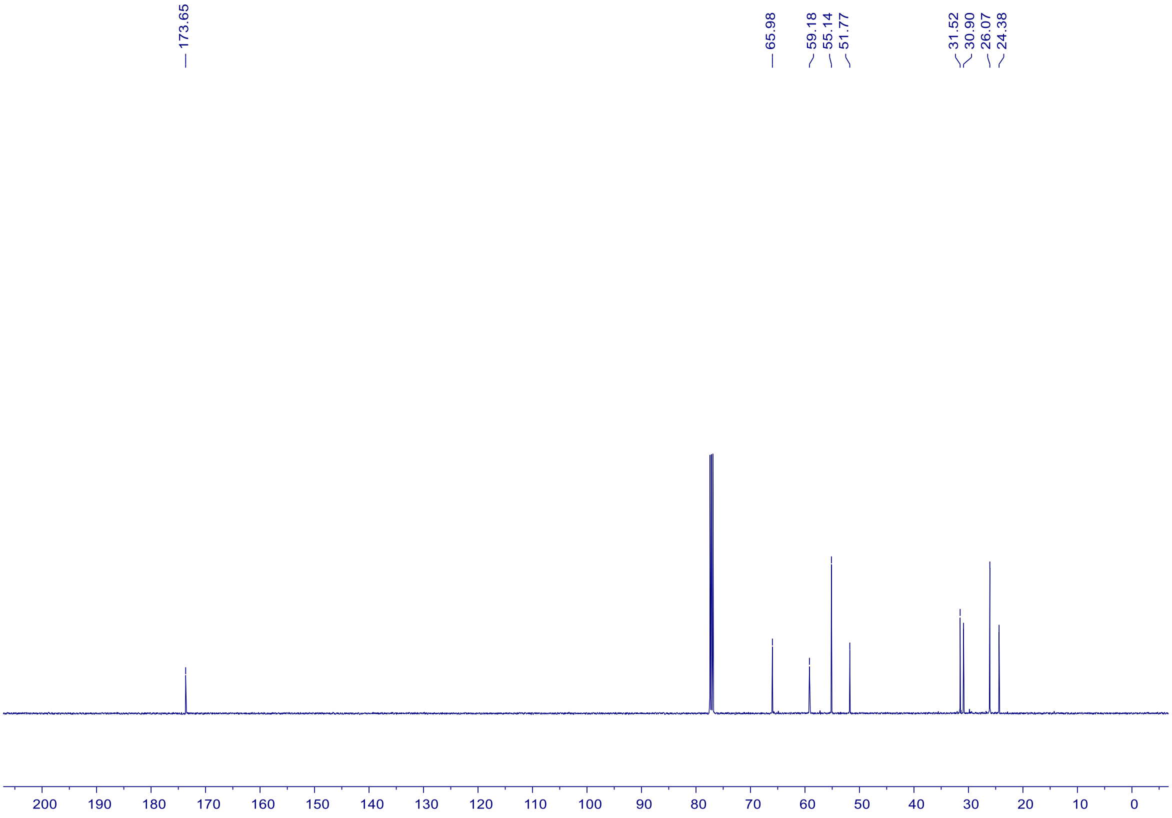

40 – ^{1}H NMR (500 MHz, $CDCl_3$)

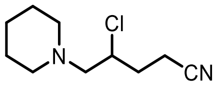

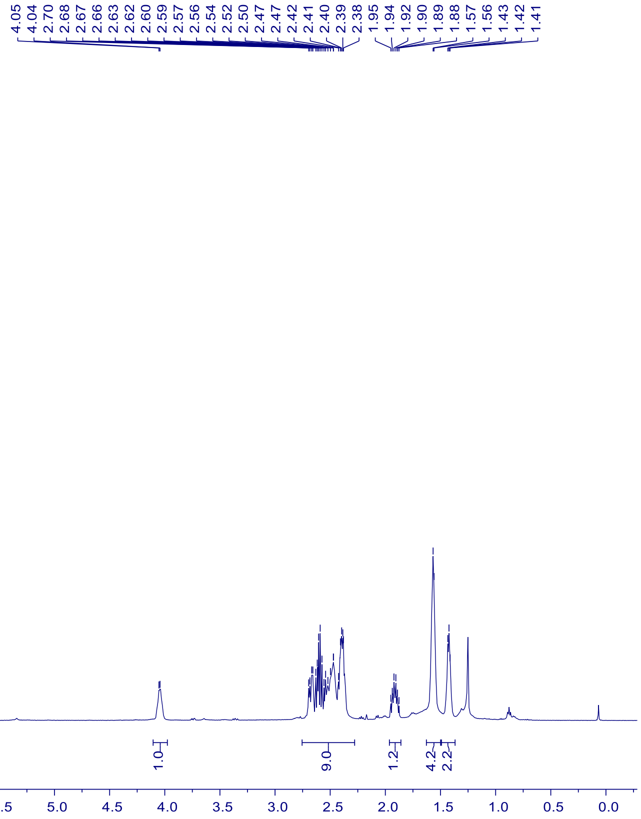

40 – ^{13}C NMR (125 MHz, $CDCl_3$)

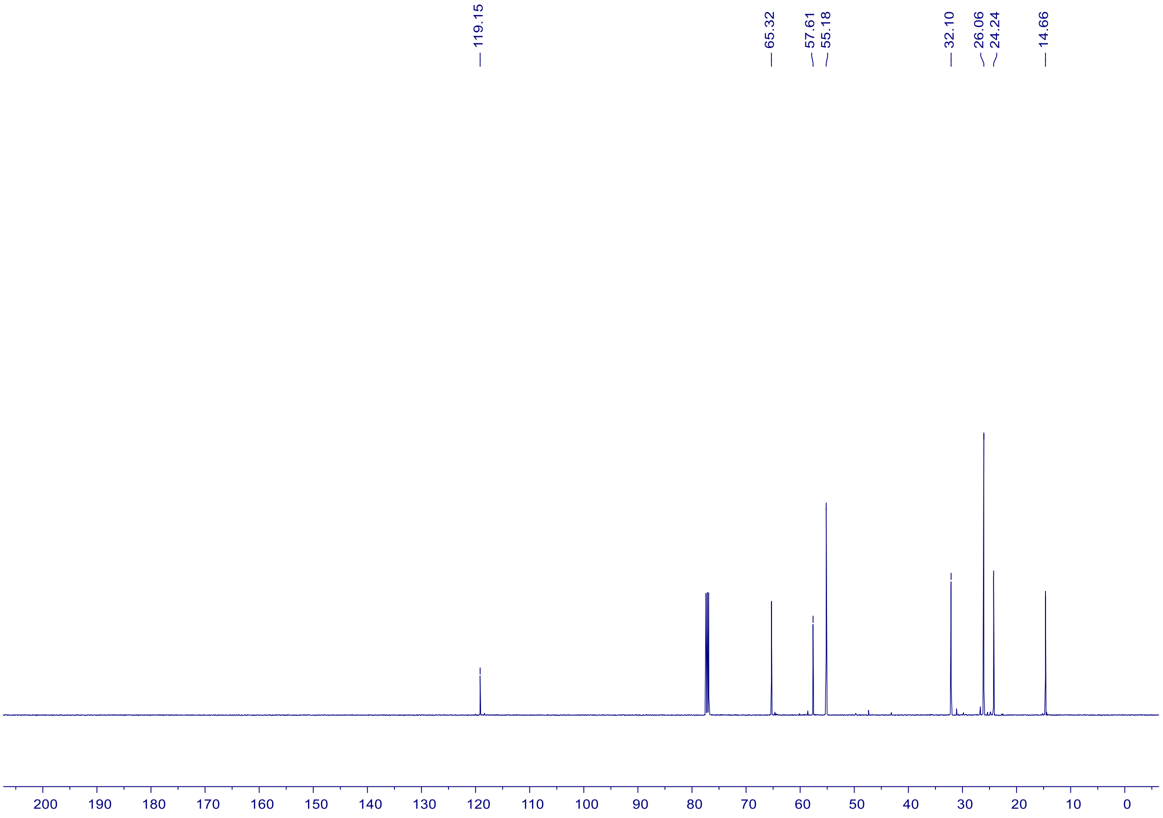

41 – ^{1}H NMR (400 MHz, CDCl3)

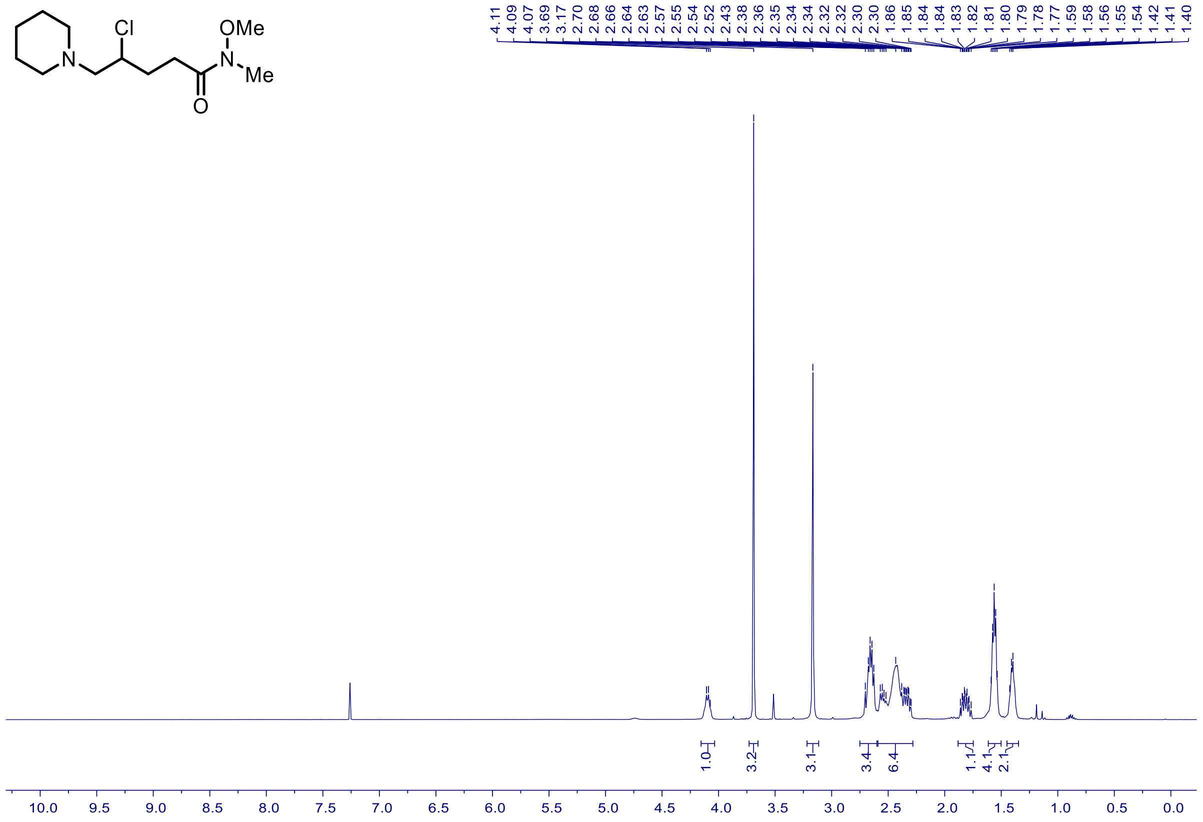

41 – ^{13}C NMR (100 MHz, CDCl3)

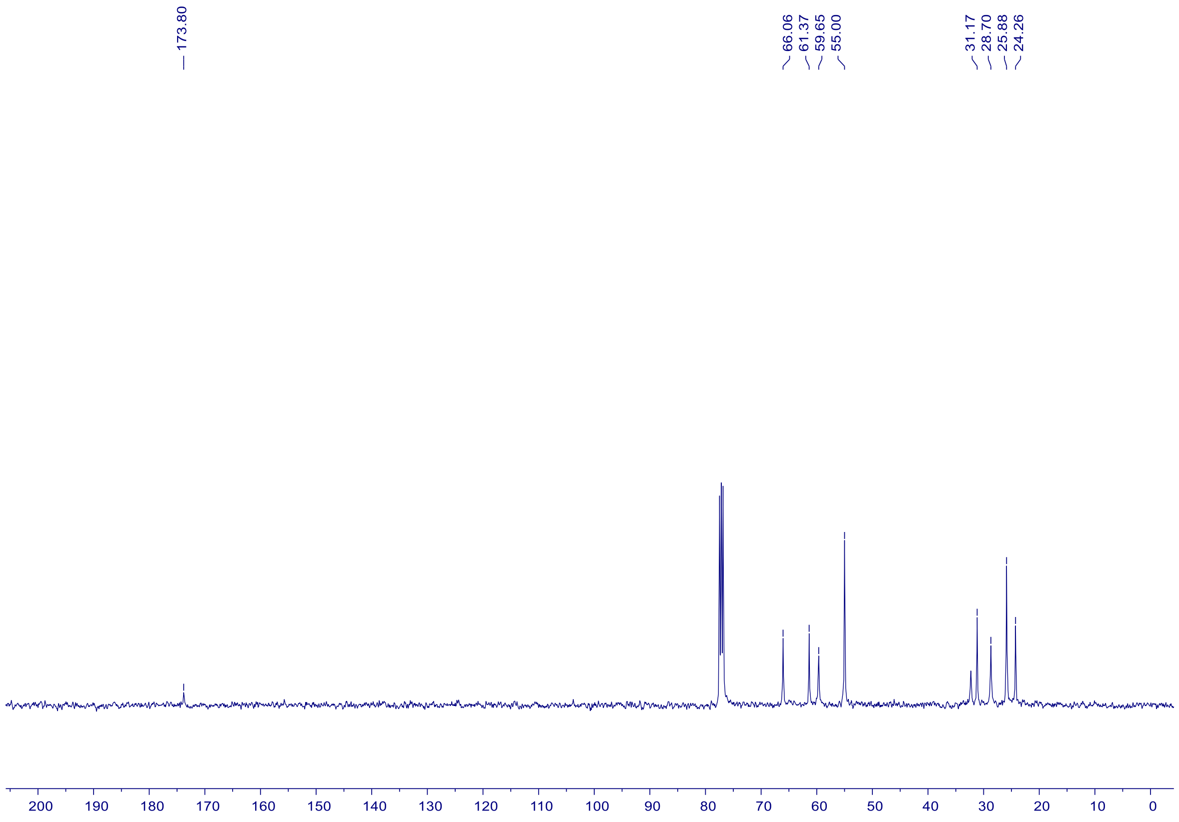

42 – ^{1}H NMR (500 MHz, CDCl$_3$)

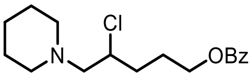

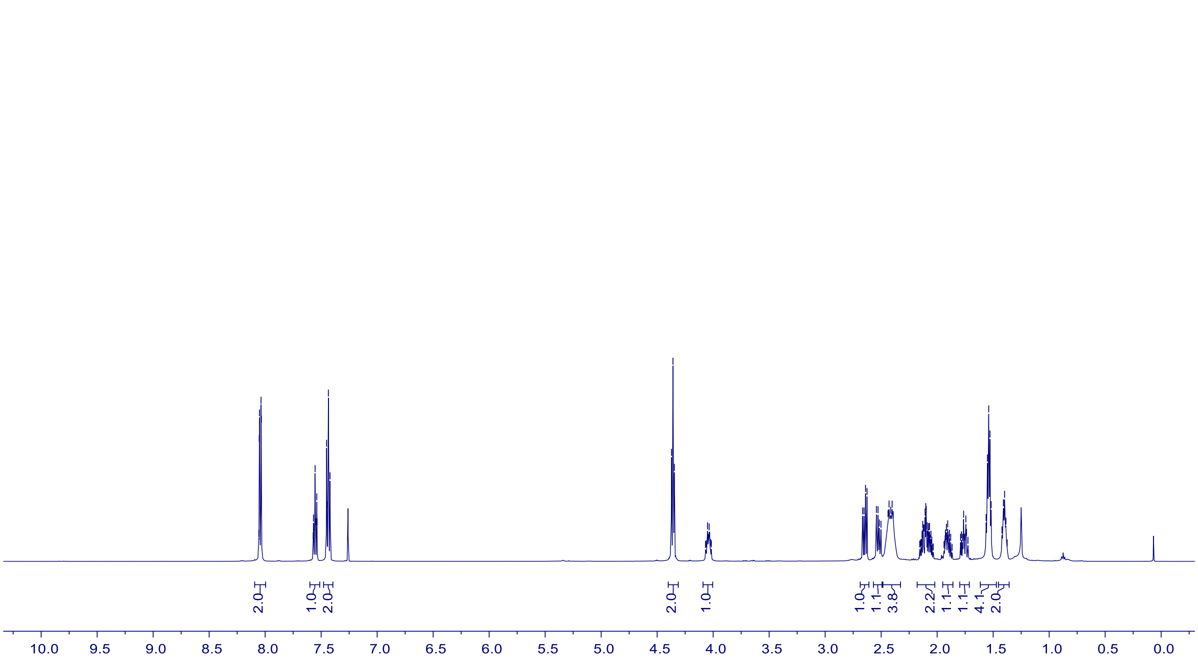

42 – ^{13}C NMR (125 MHz, CDCl$_3$)

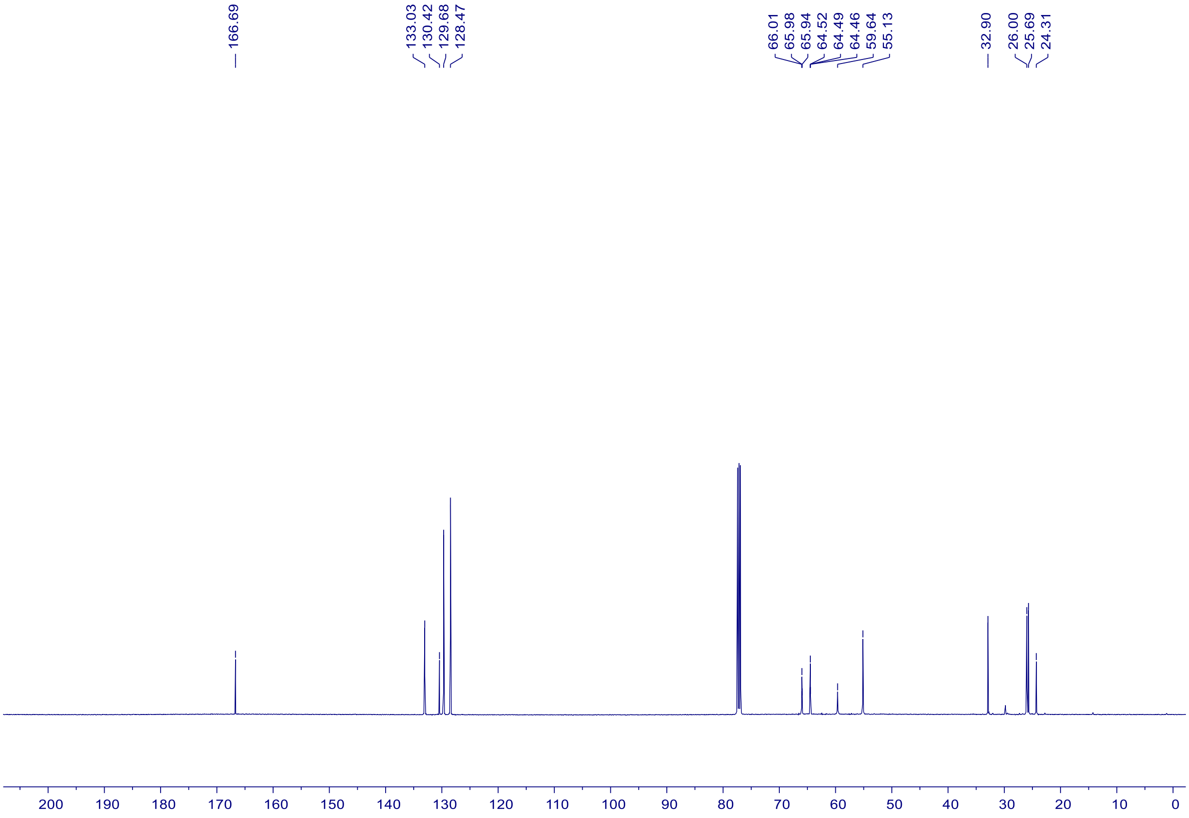

43 – ^{1}H NMR (400 MHz, $CDCl_3$)

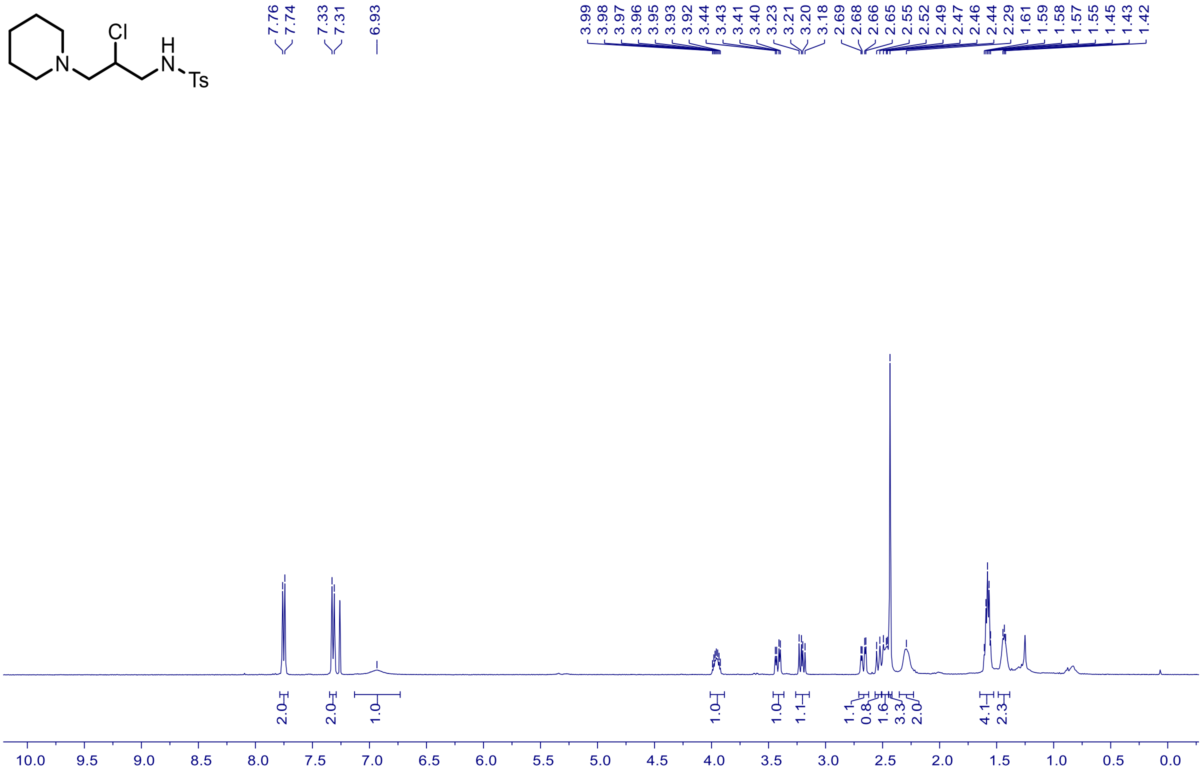

43 – ^{13}C NMR (125 MHz, $CDCl_3$)

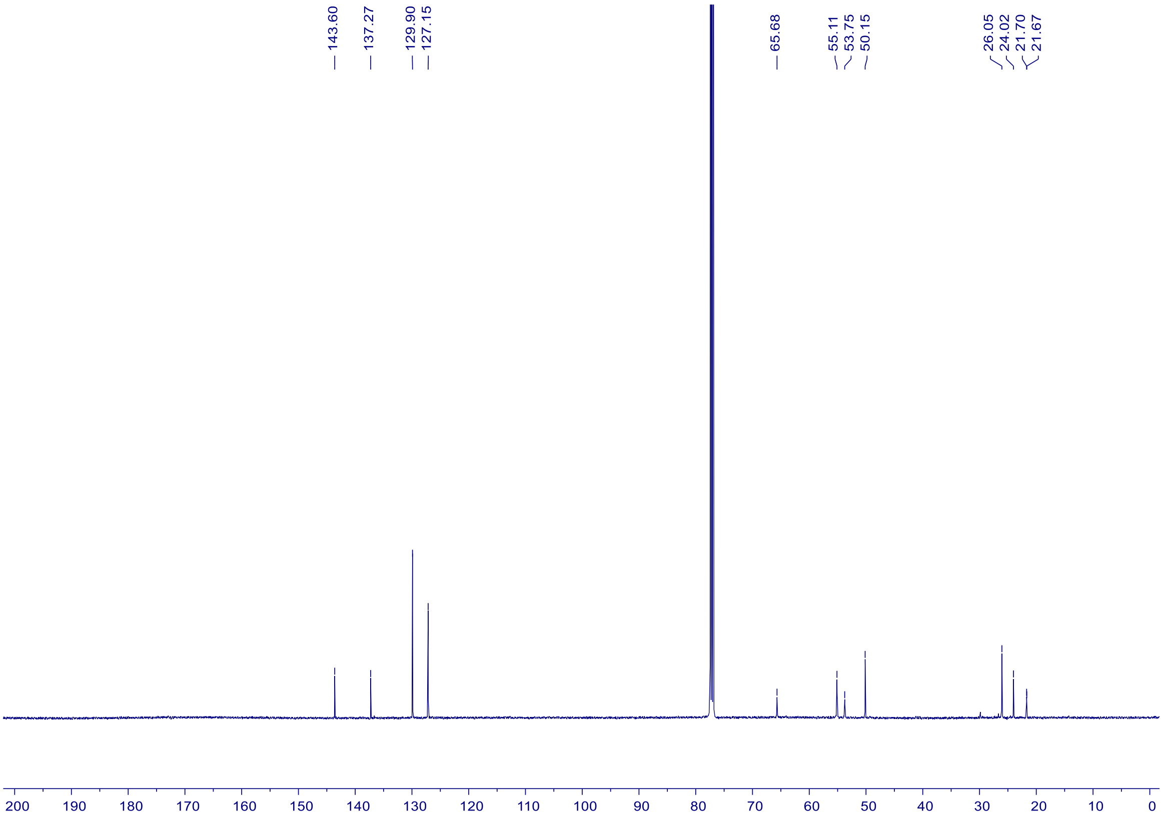

44 – ^{1}H NMR (500 MHz, $CDCl_3$)

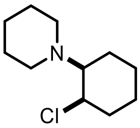

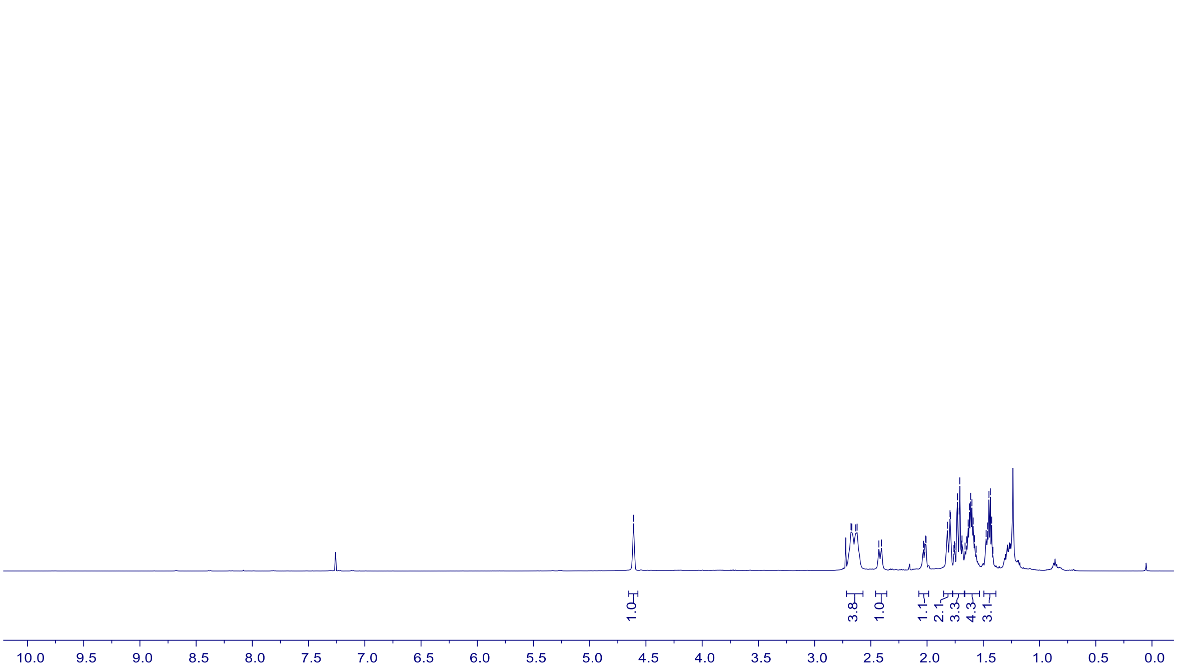

44 – ^{13}C NMR (125 MHz, $CDCl_3$)

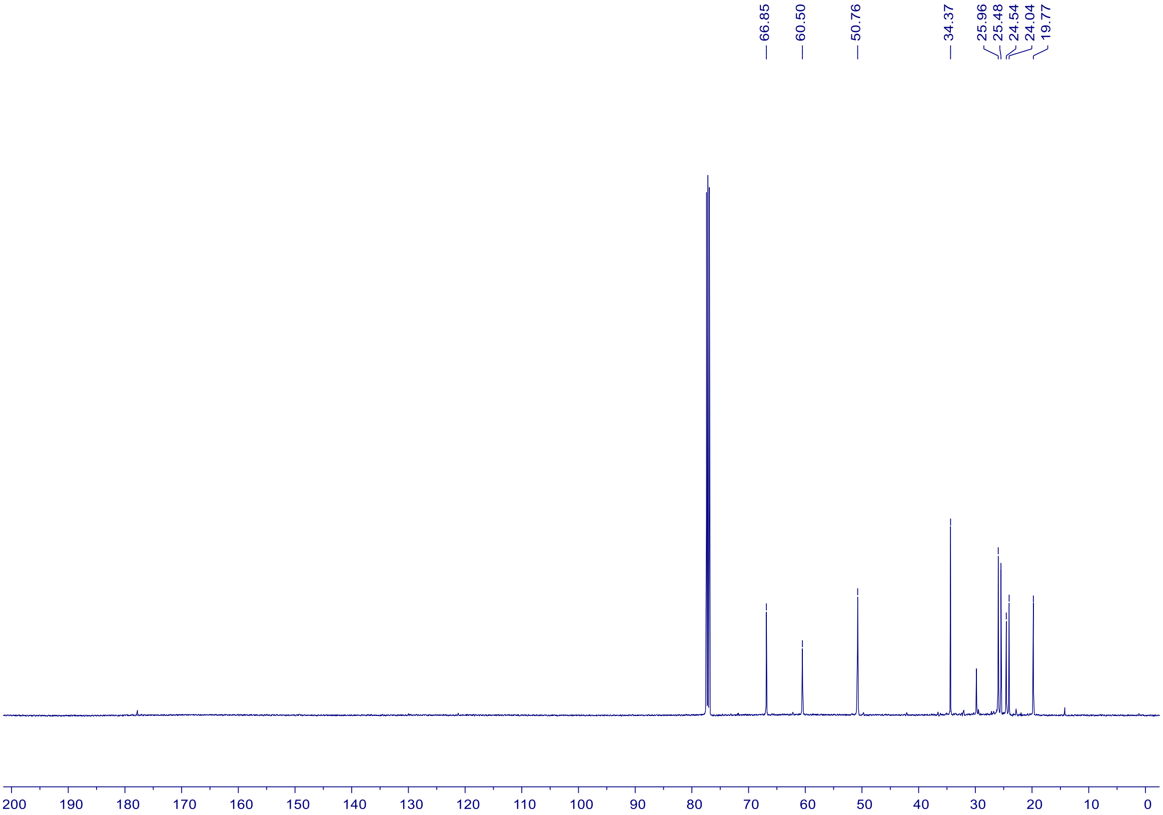

45 – ^{1}H NMR (500 MHz, $CDCl_3$)

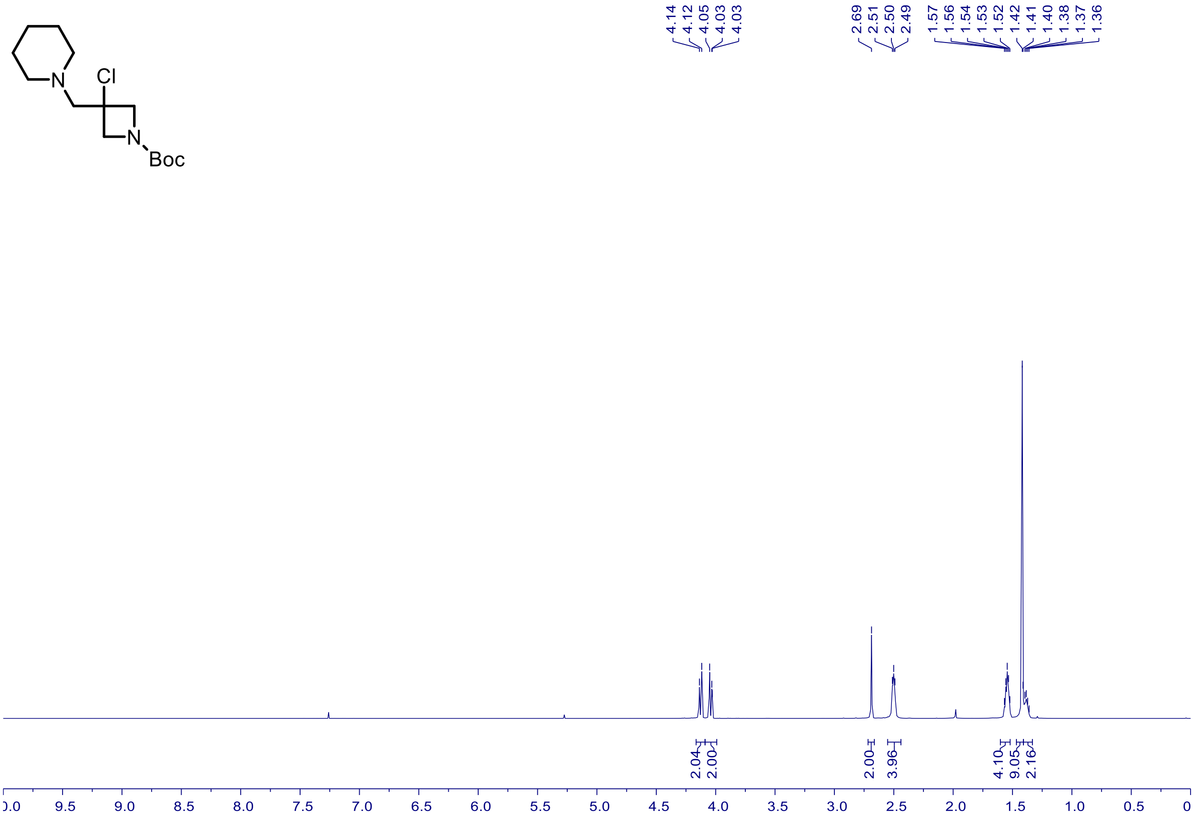

45 – ^{13}C NMR (126 MHz, $CDCl_3$)

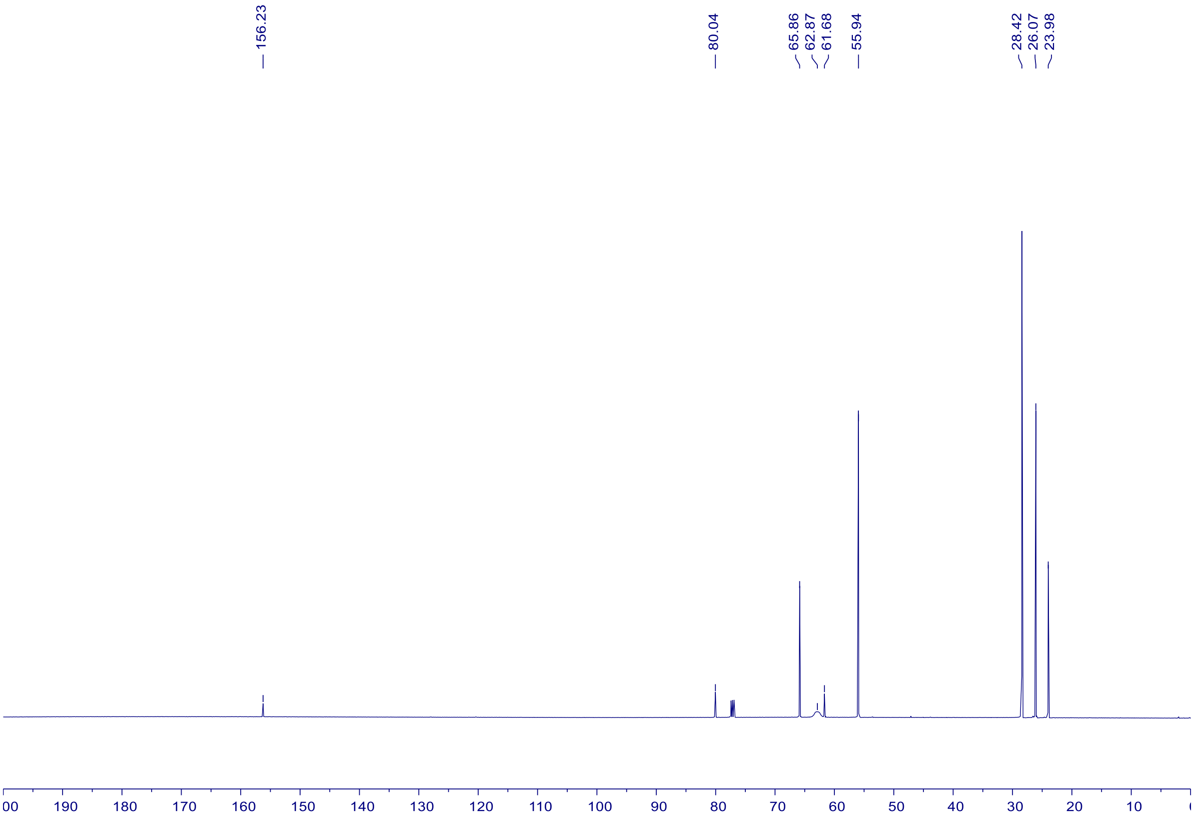

46 (second eluting isomer) – ^{1}H NMR (400 MHz, $CDCl_3$)

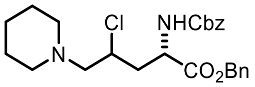

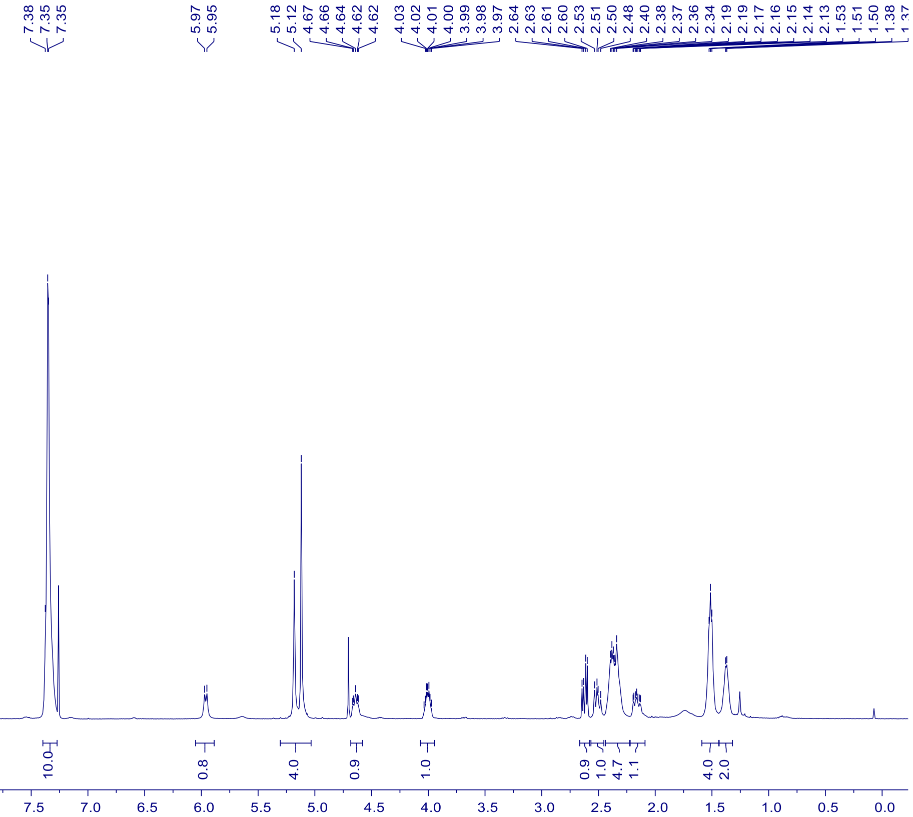

46 (second eluting isomer) – ^{13}C NMR (125 MHz, $CDCl_3$)

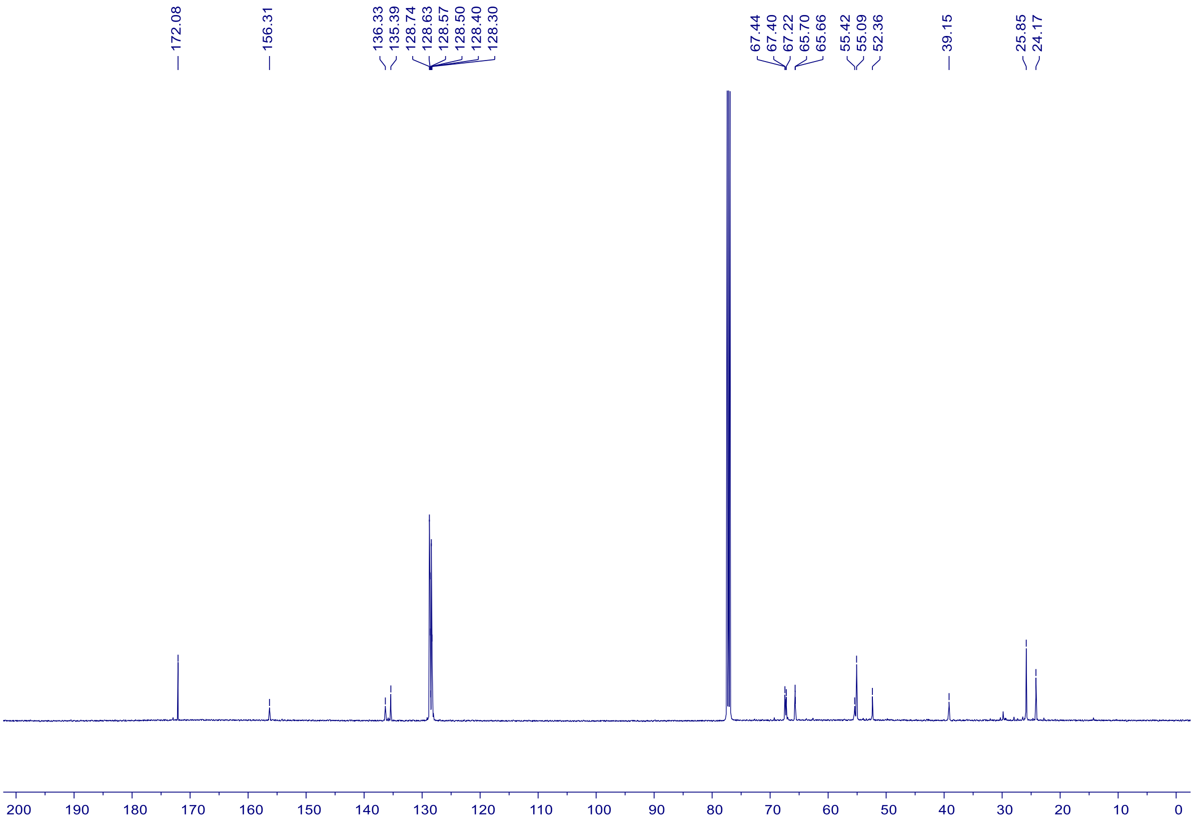

47 – ^{1}H NMR (500 MHz, CDCl3)

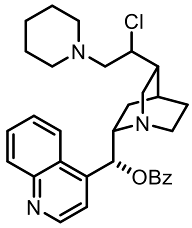

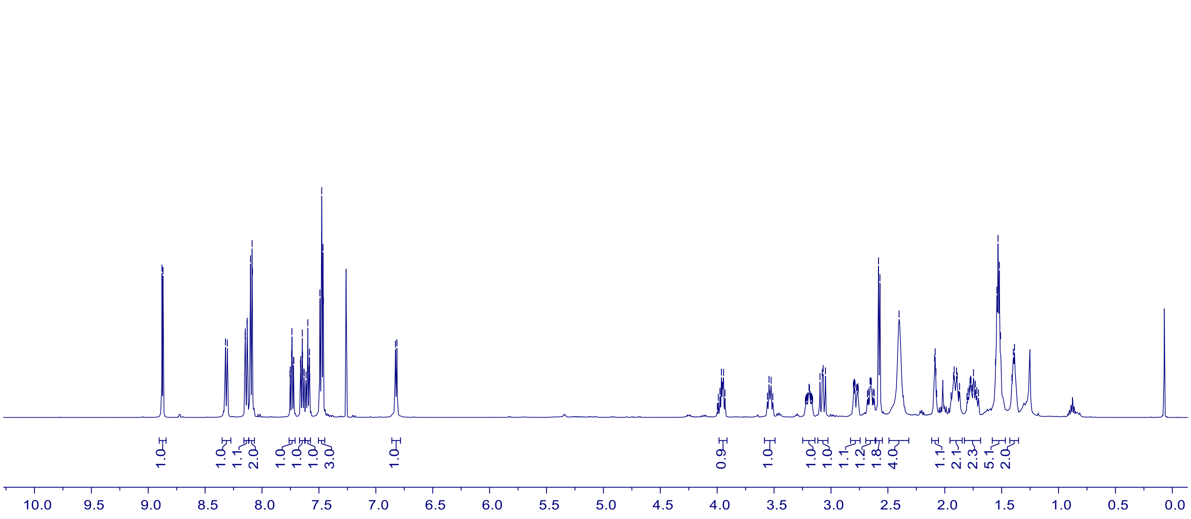

47 – ^{13}C NMR (125 MHz, CDCl3)

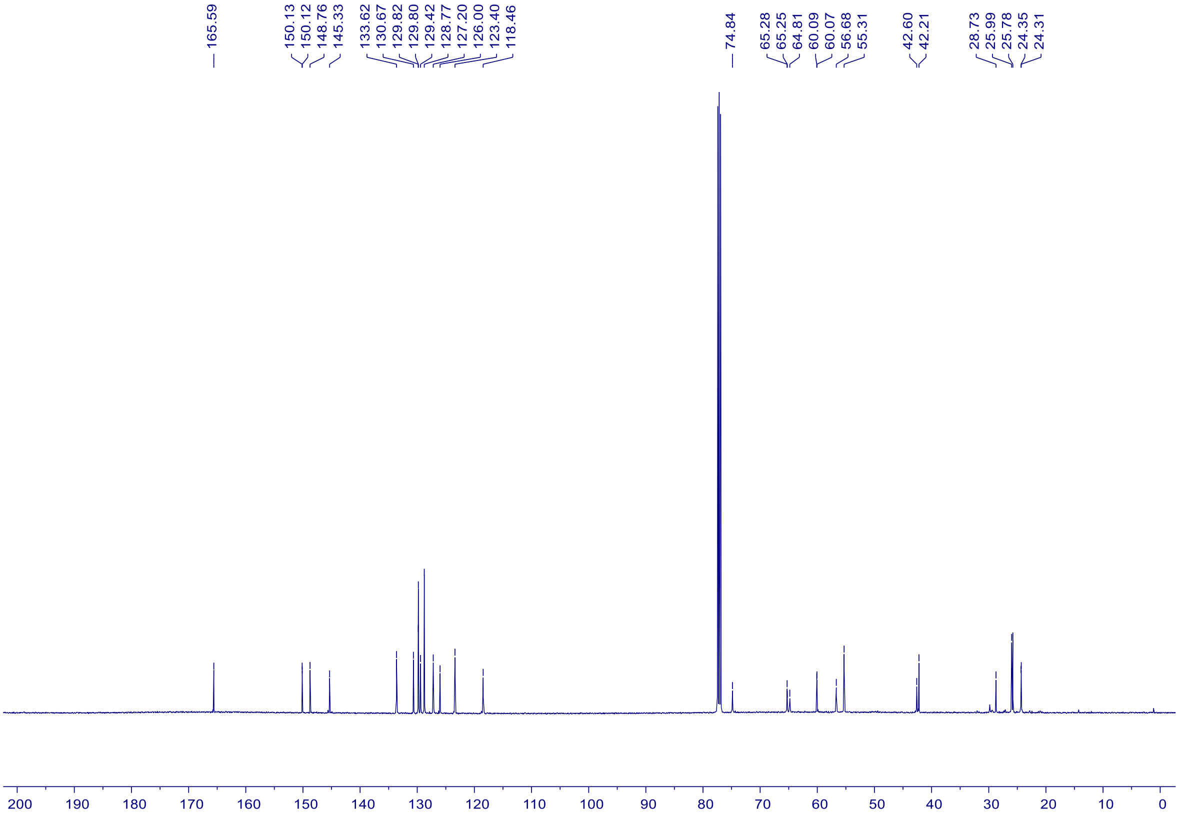

48 – ^{1}H NMR (500 MHz, CDCl$_3$)

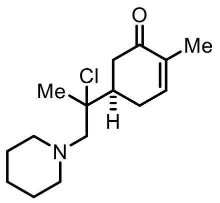

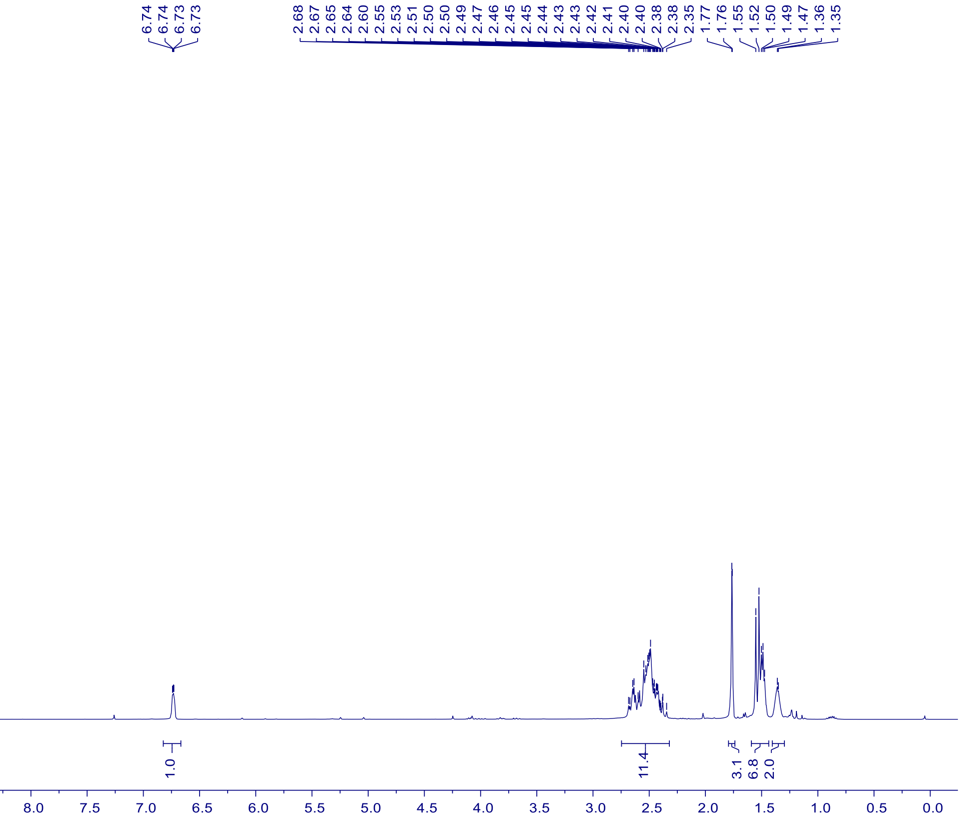

48 – ^{13}C NMR (125 MHz, CDCl$_3$, diastereomers)

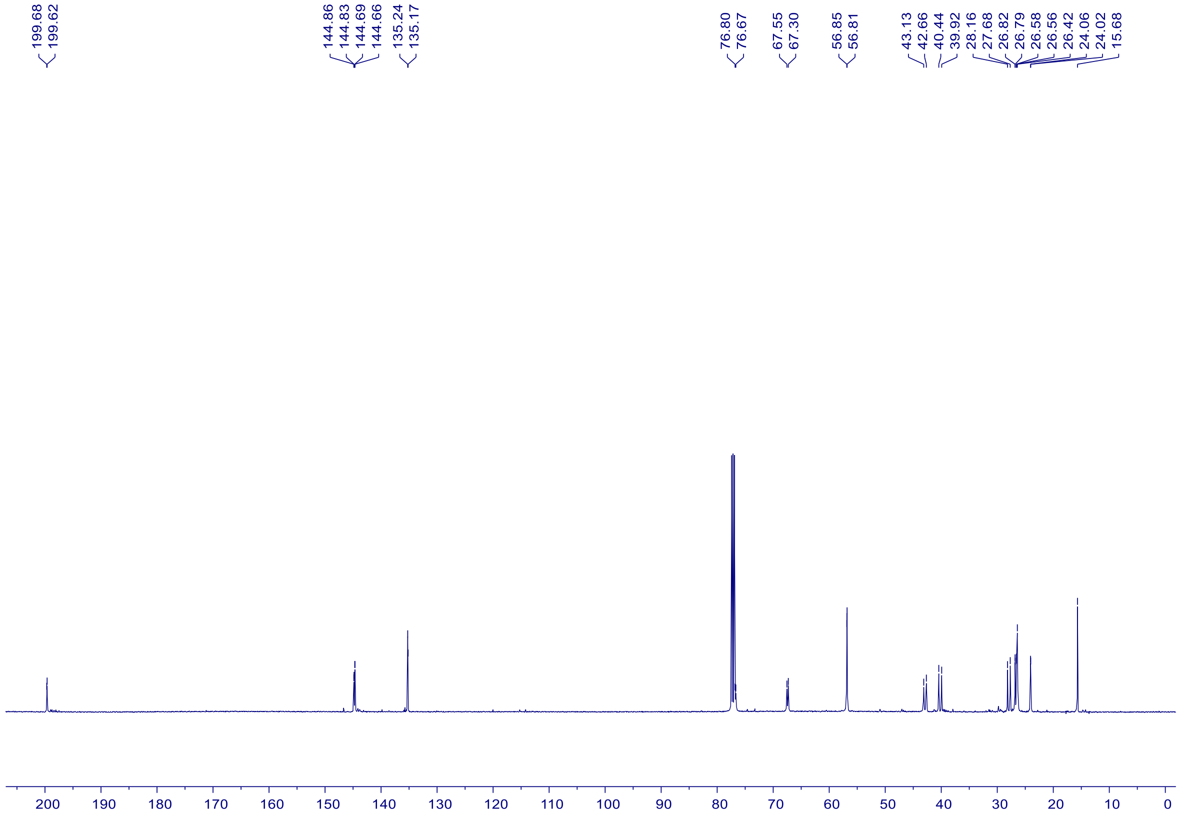

49 – ^{1}H NMR (400 MHz, $CDCl_3$)

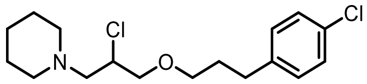

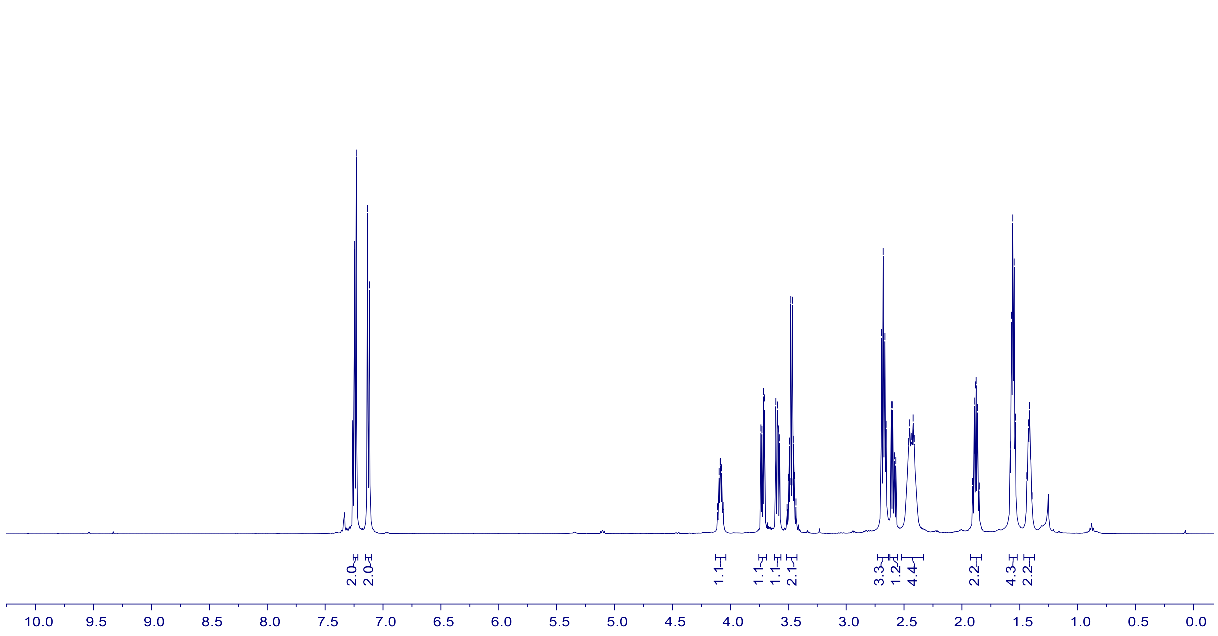

49 – ^{13}C NMR (125 MHz, $CDCl_3$)

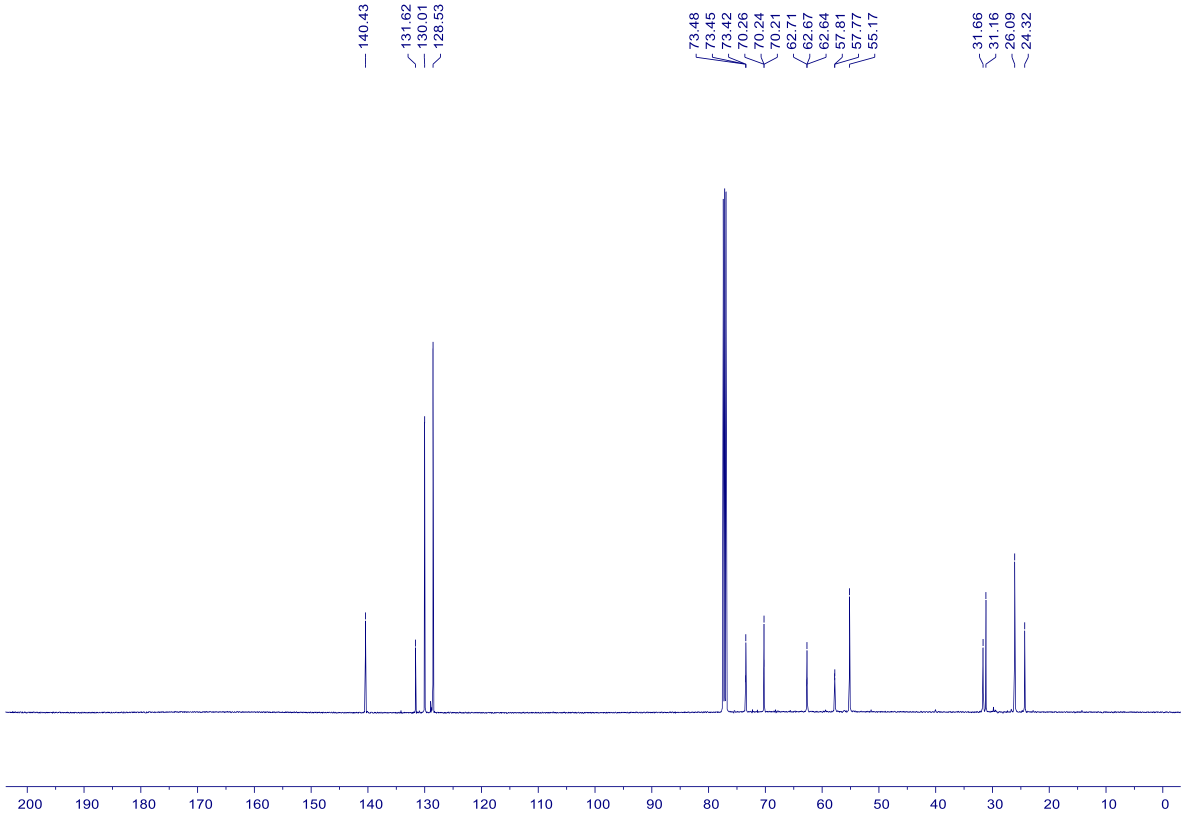

50 – ^{1}H NMR (500 MHz, $CDCl_3$)

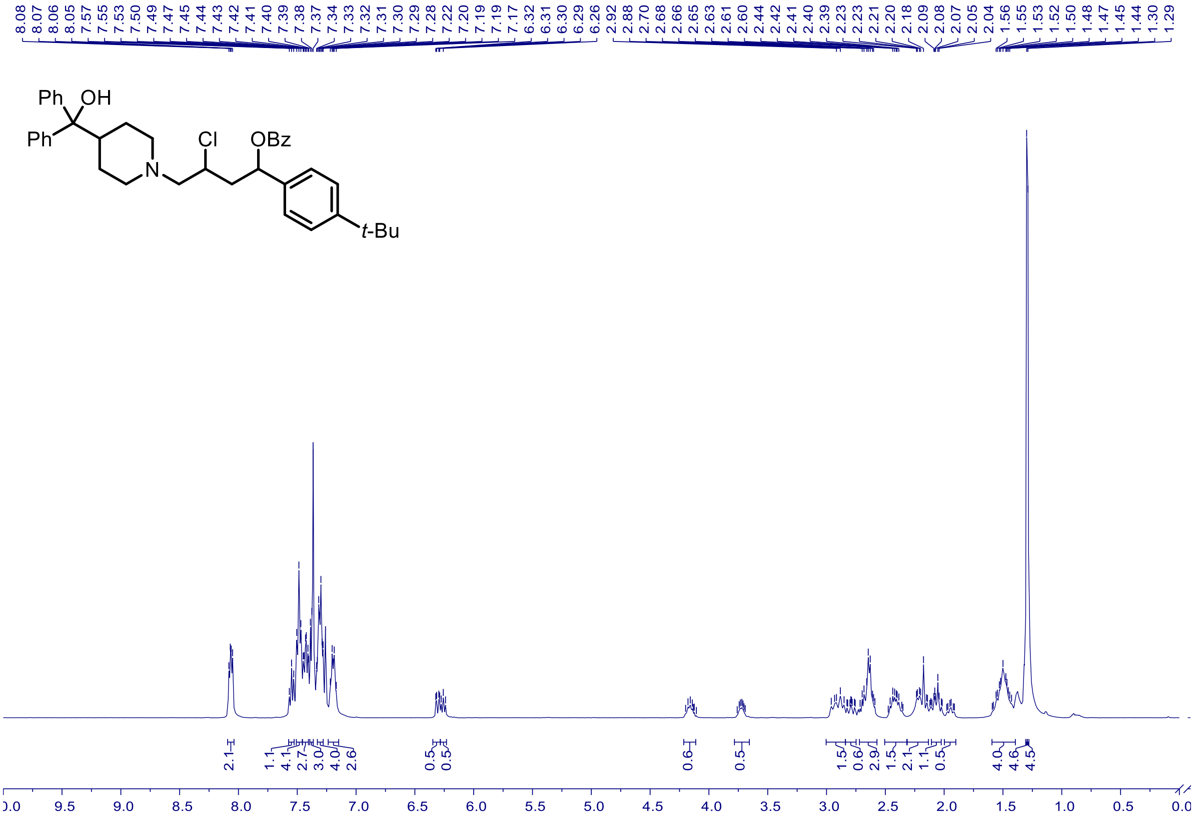

50 – ^{13}C NMR (126 MHz, $CDCl_3$)

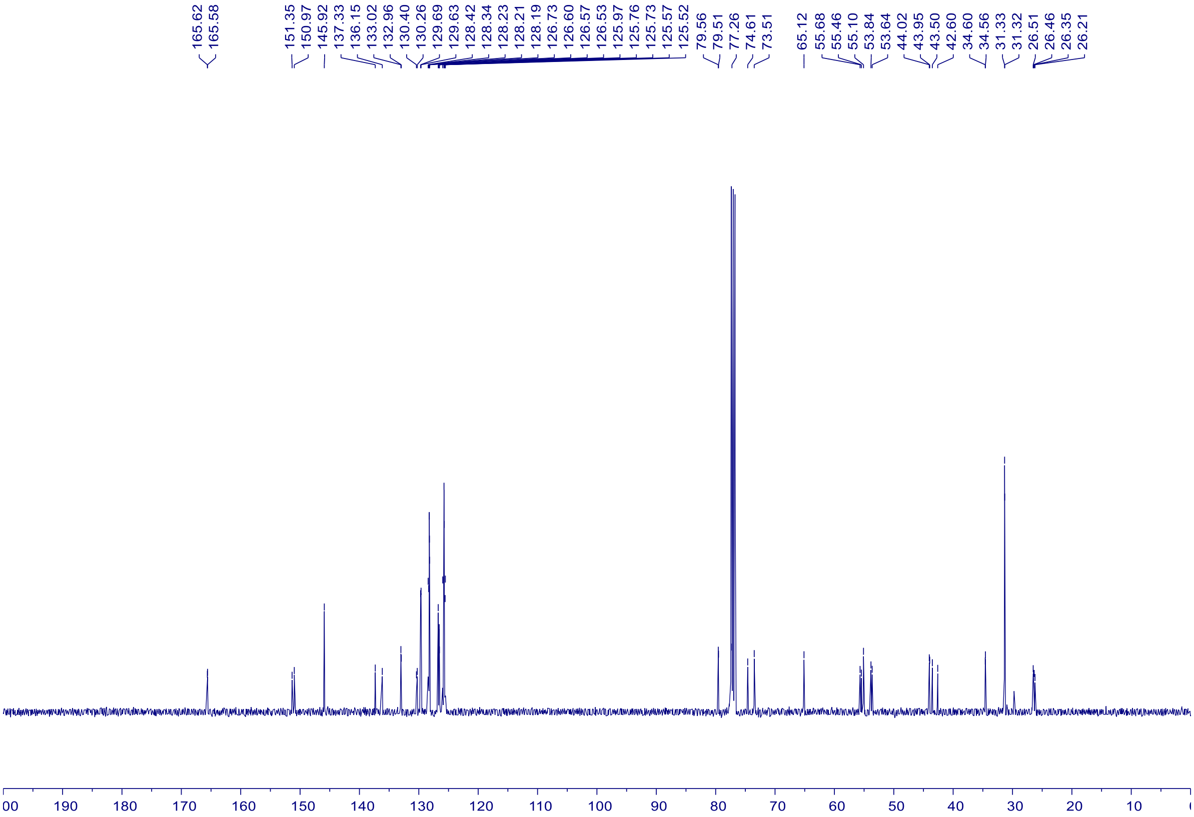

51 – ^{1}H NMR (500 MHz, CD_2Cl_2, reaction crude due to product decomposition)

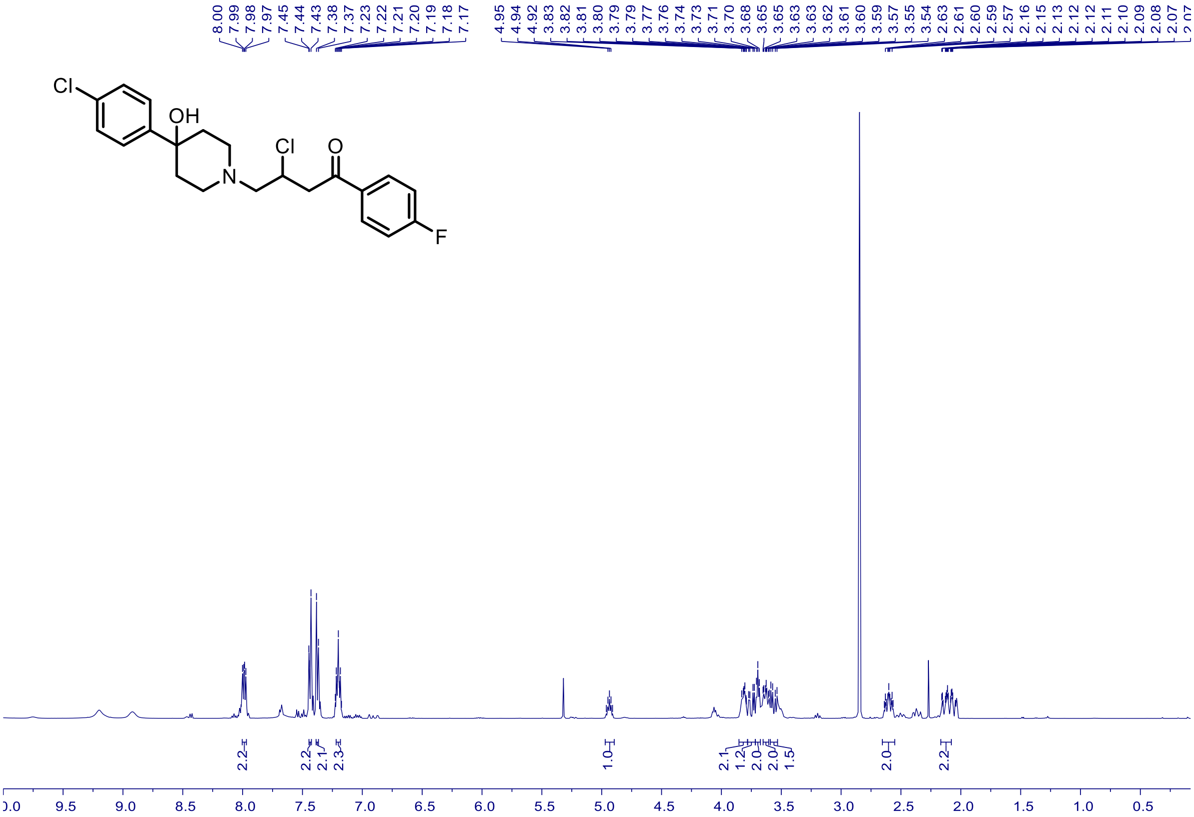

51 – ^{13}C NMR (126 MHz, $CDCl_3$, reaction crude)

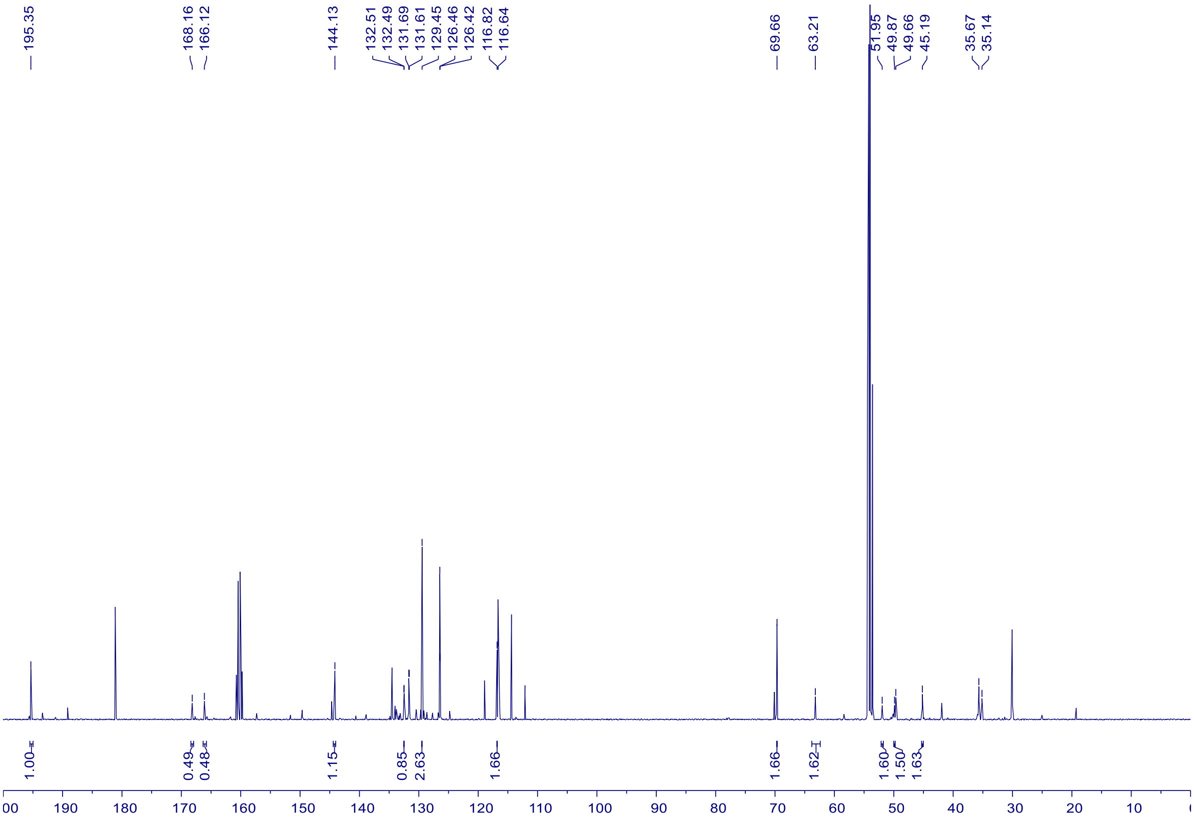

51 – ^{19}F NMR (471 MHz, $CDCl_3$, reaction crude)

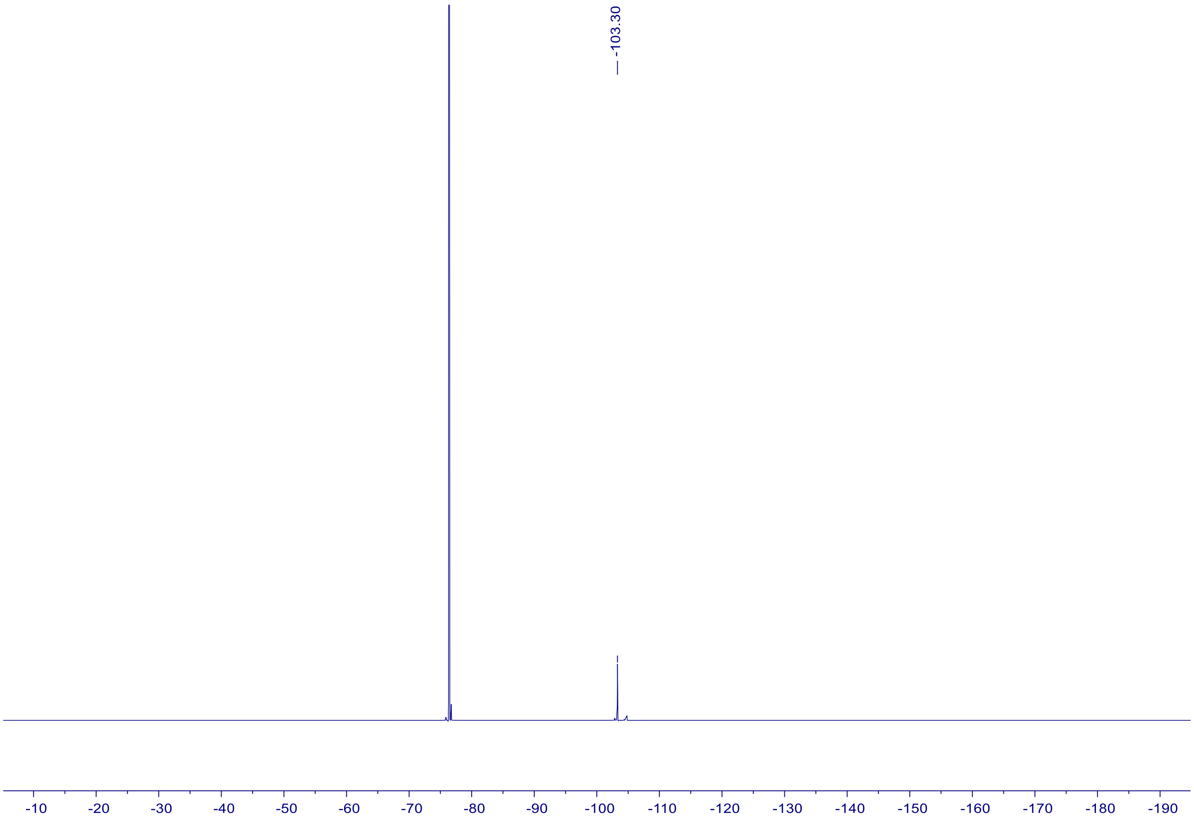

52 – ^{1}H NMR (500 MHz, $CDCl_3$)

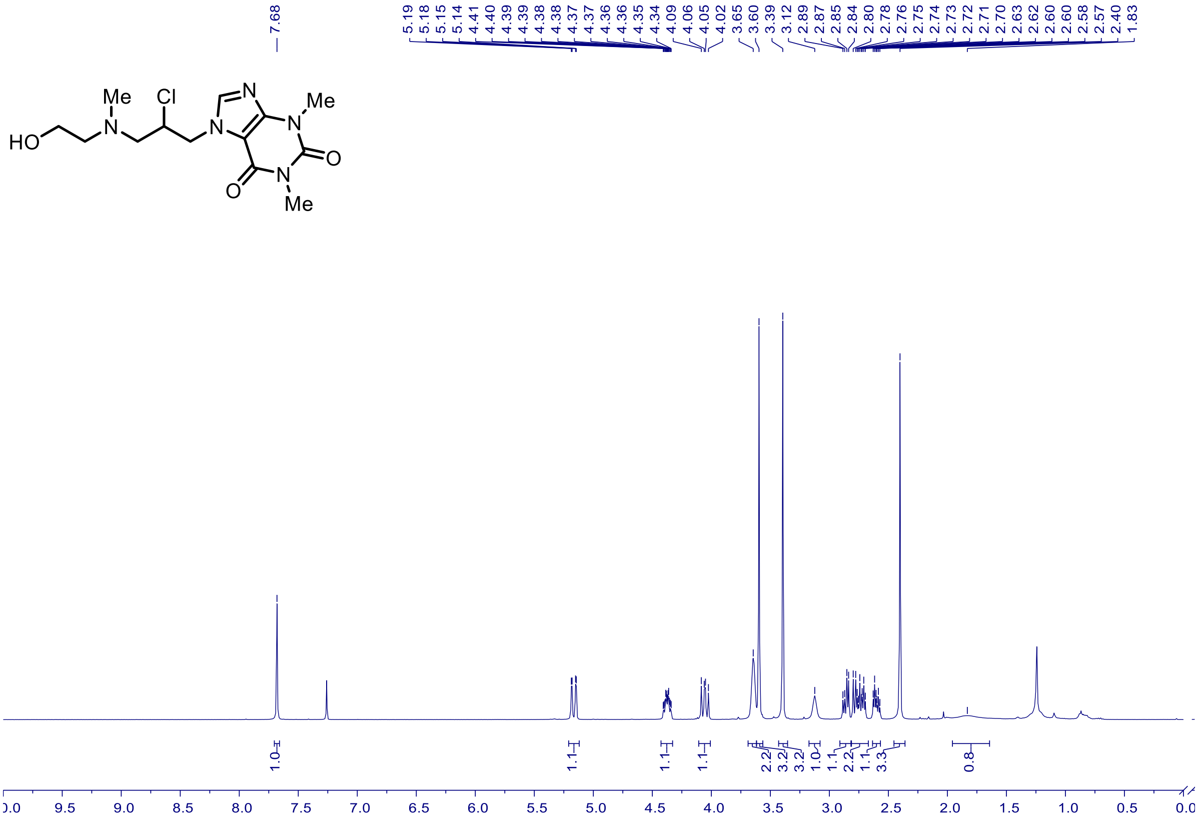

52 – ^{13}C NMR (126 MHz, $CDCl_3$)

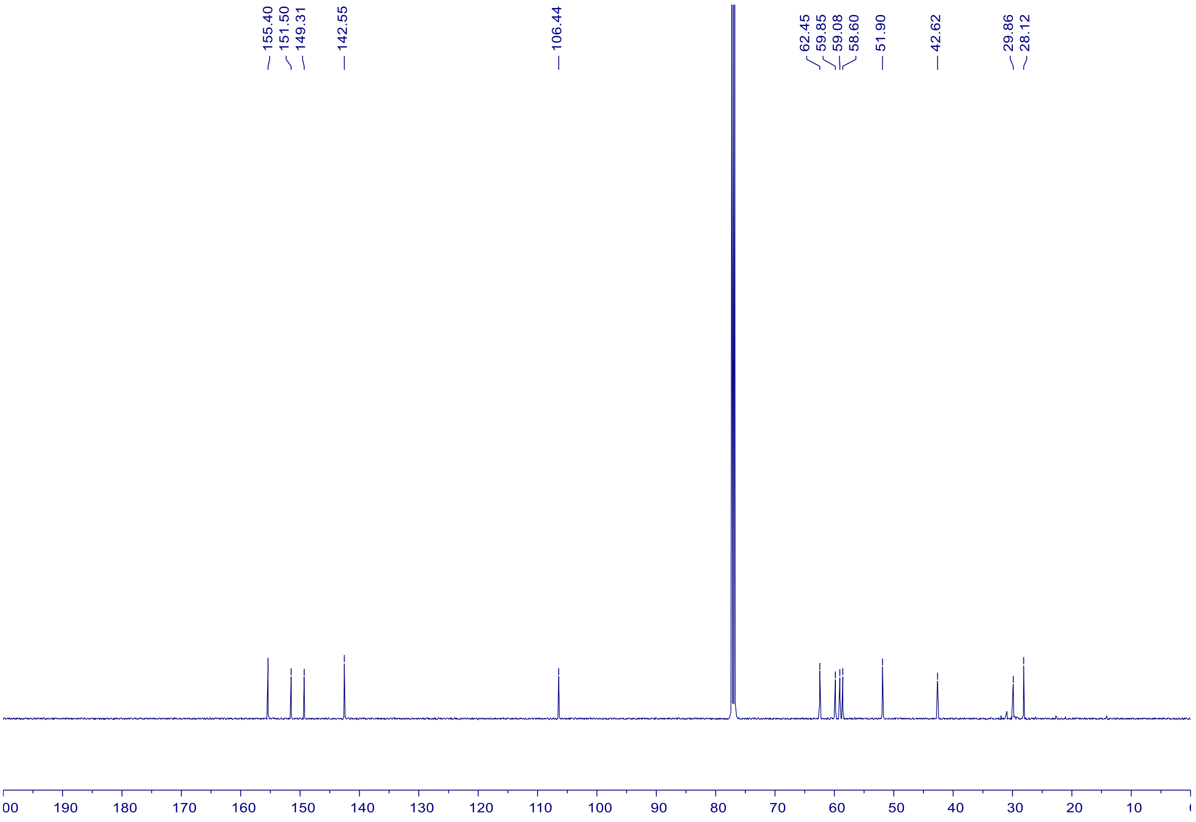

102 – ^{1}H NMR (500 MHz, $CDCl_3$)

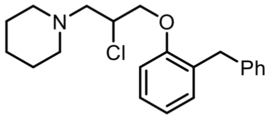

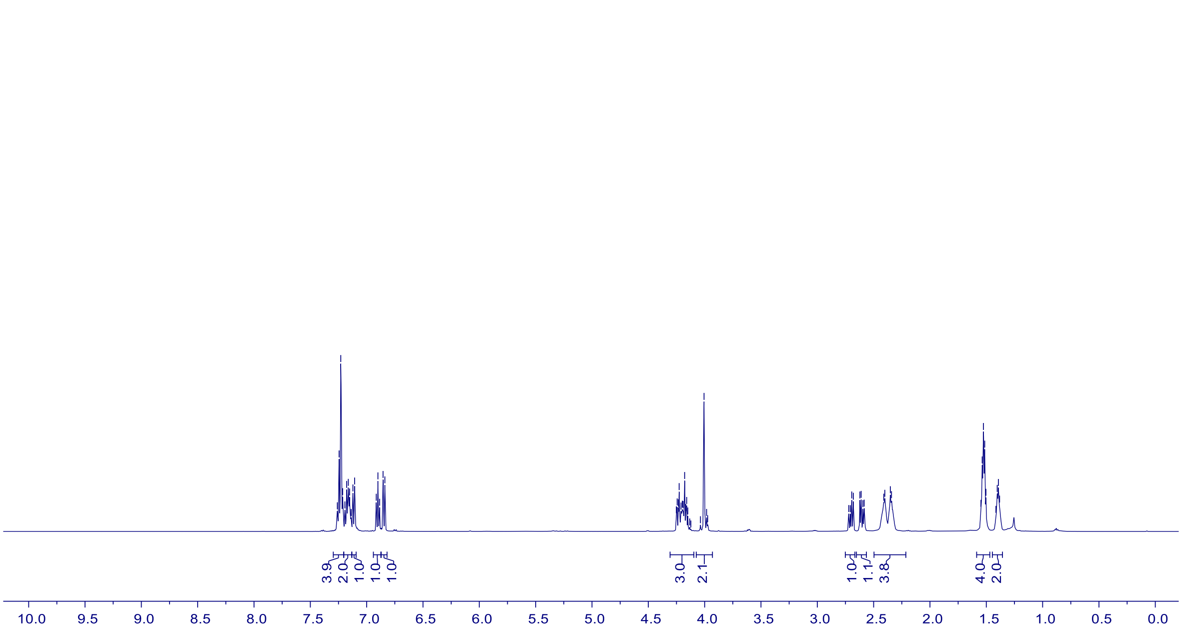

102 – ^{13}C NMR (125 MHz, $CDCl_3$)

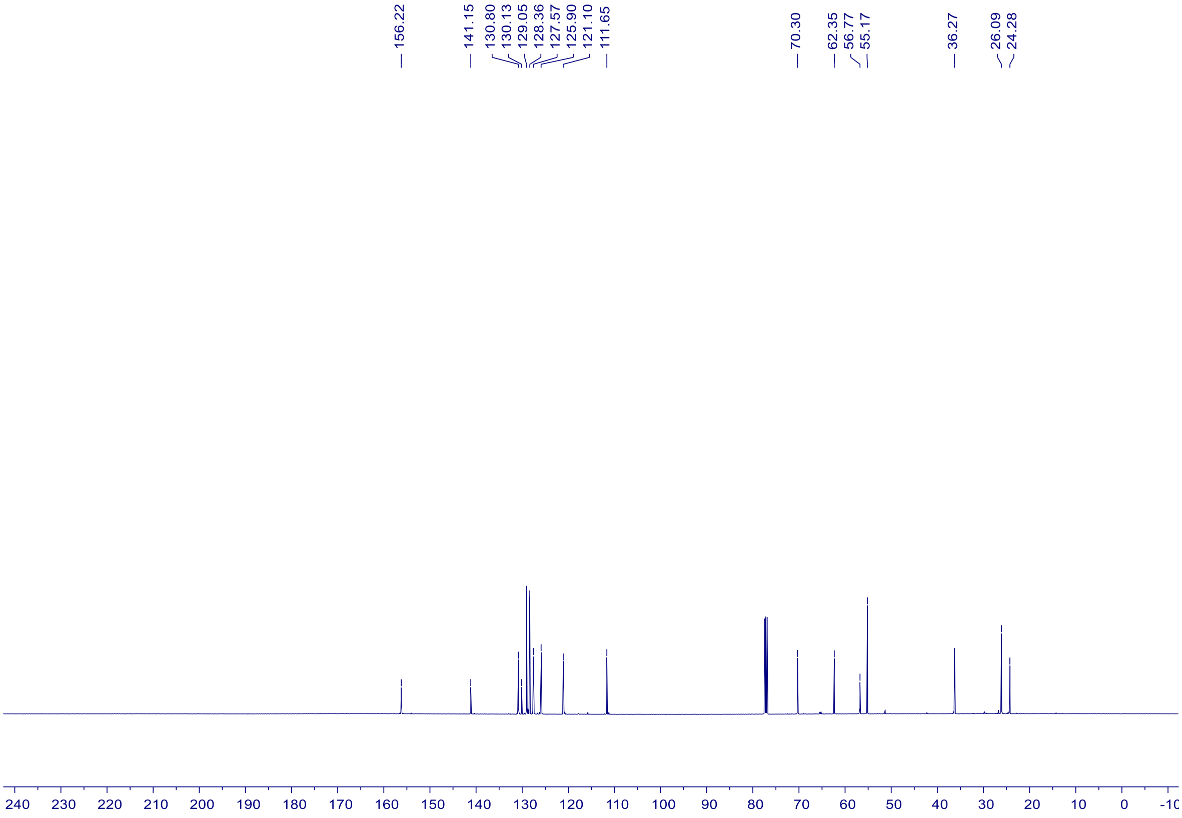

56 – ¹H NMR (500 MHz, $CDCl_3$)

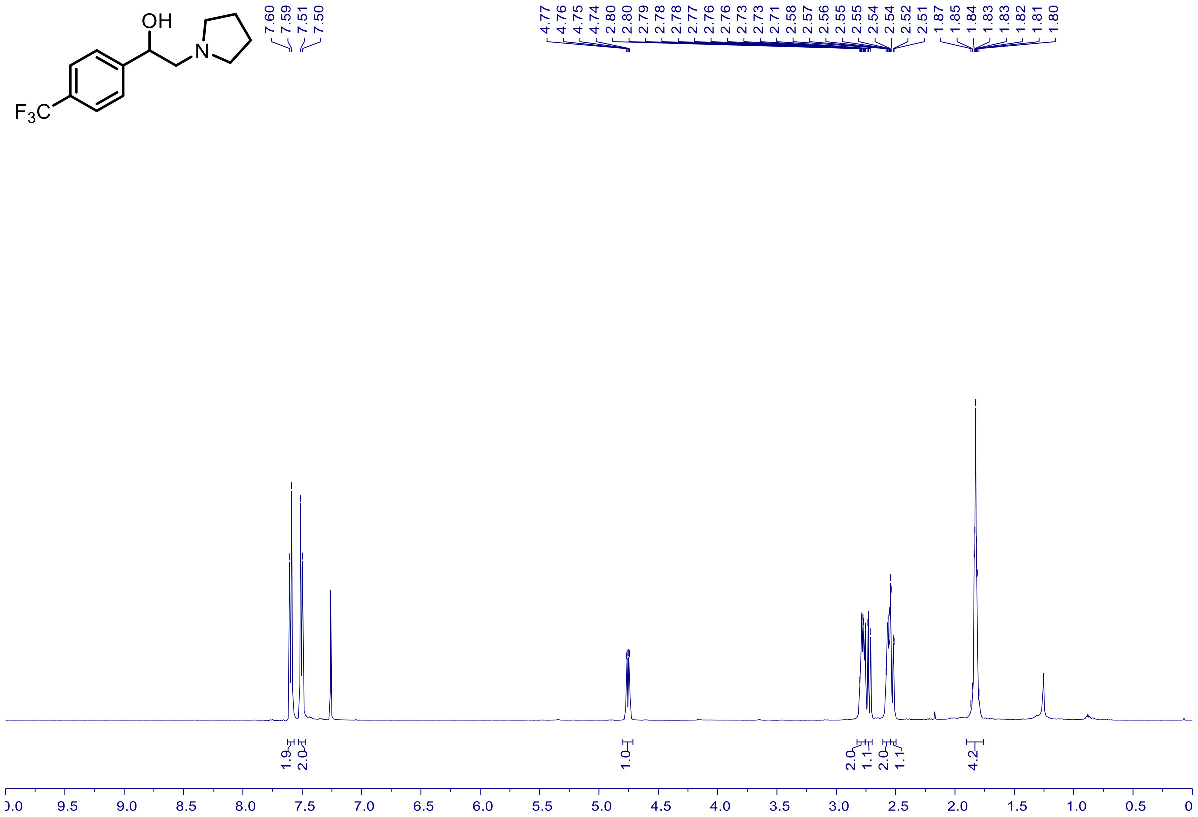

56 – ¹³C NMR (126 MHz, $CDCl_3$)

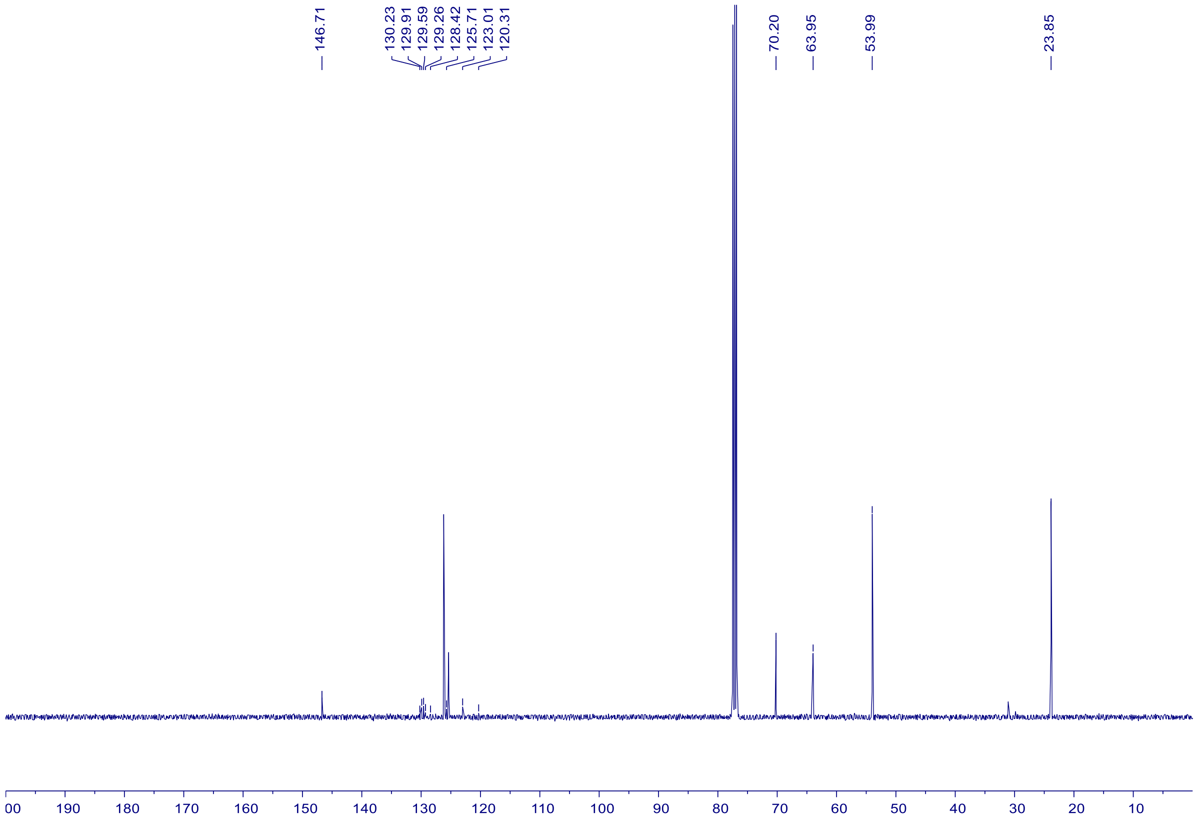

55 – ^{19}F NMR (471 MHz, $CDCl_3$)

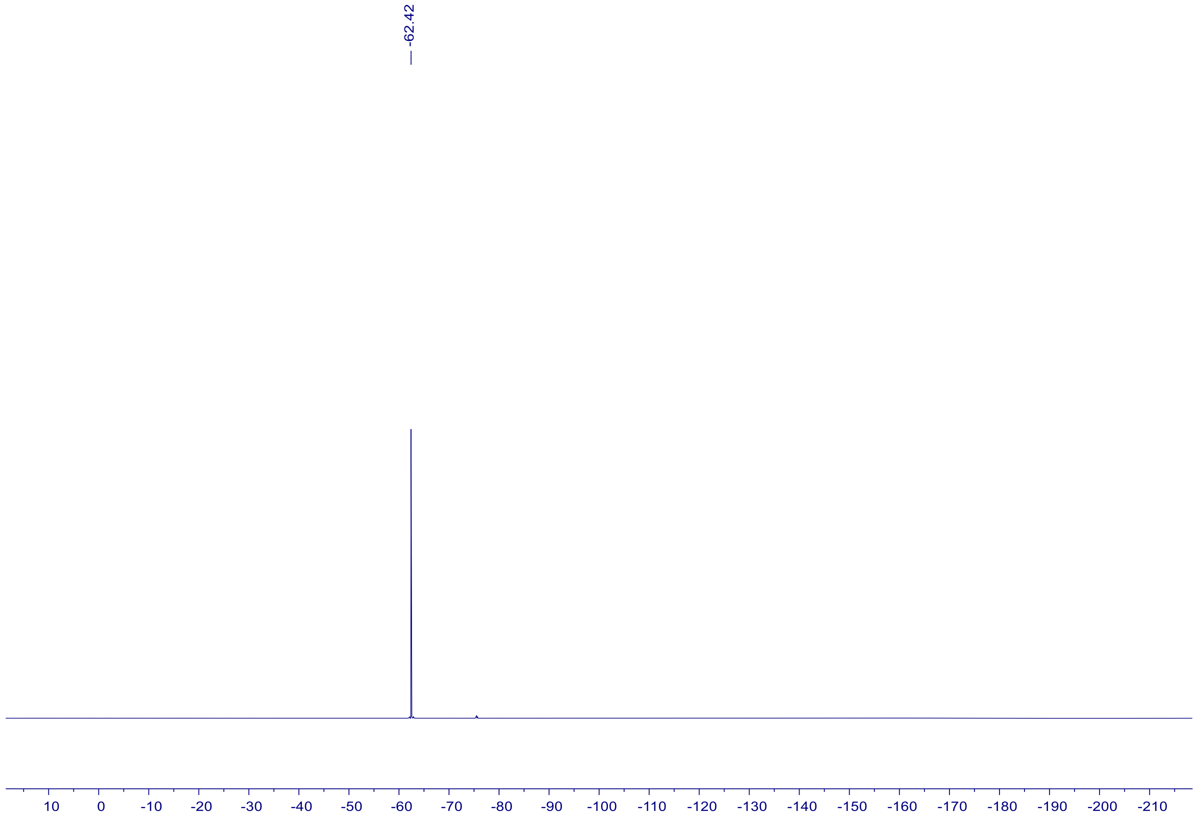

57 – ^{1}H NMR (500 MHz, $CDCl_3$)

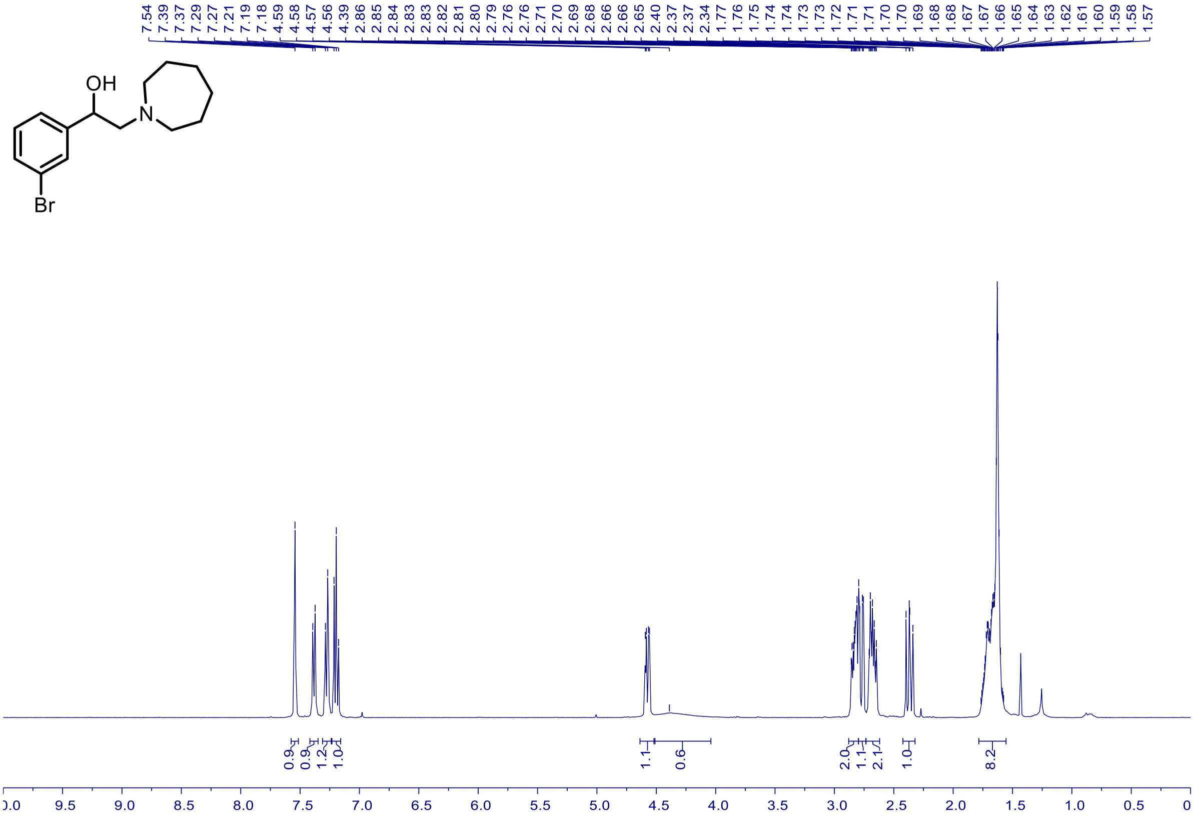

57 – ^{13}C NMR (126 MHz, $CDCl_3$)

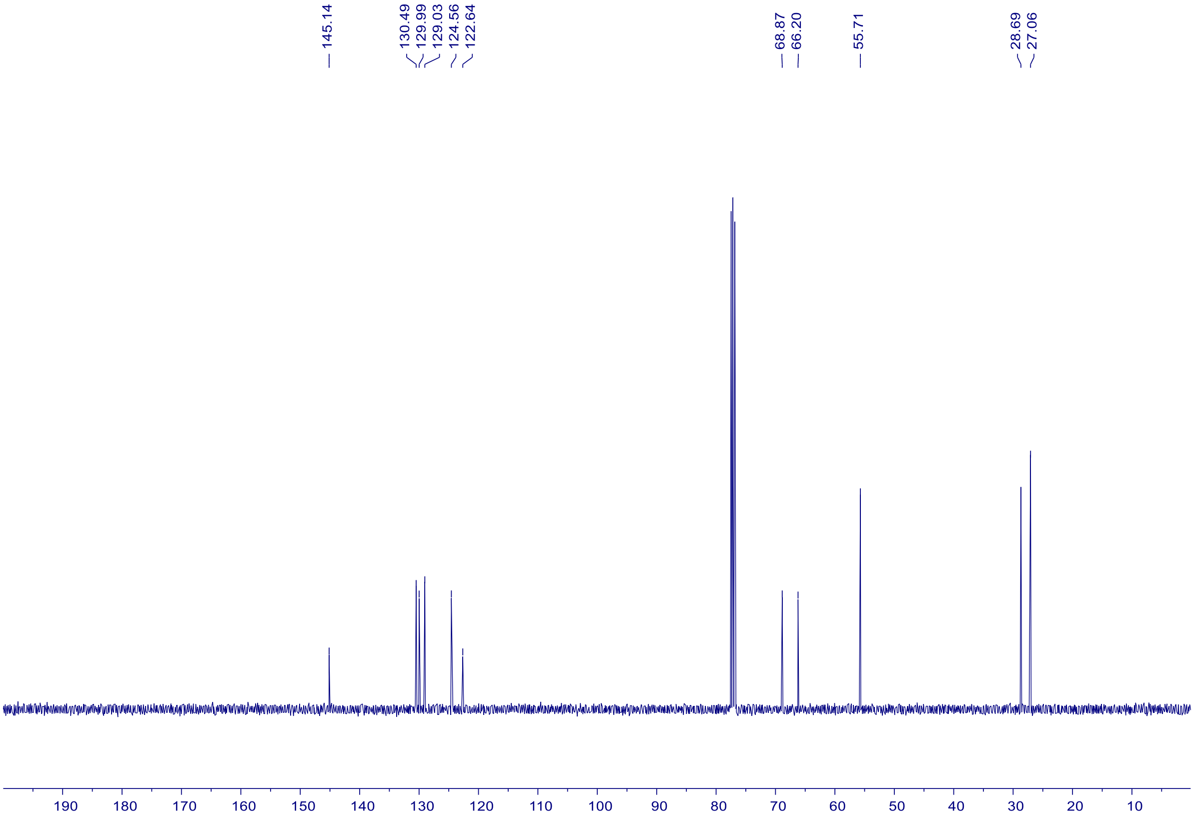

59 – ^{1}H NMR (400 MHz, CD_3CN)

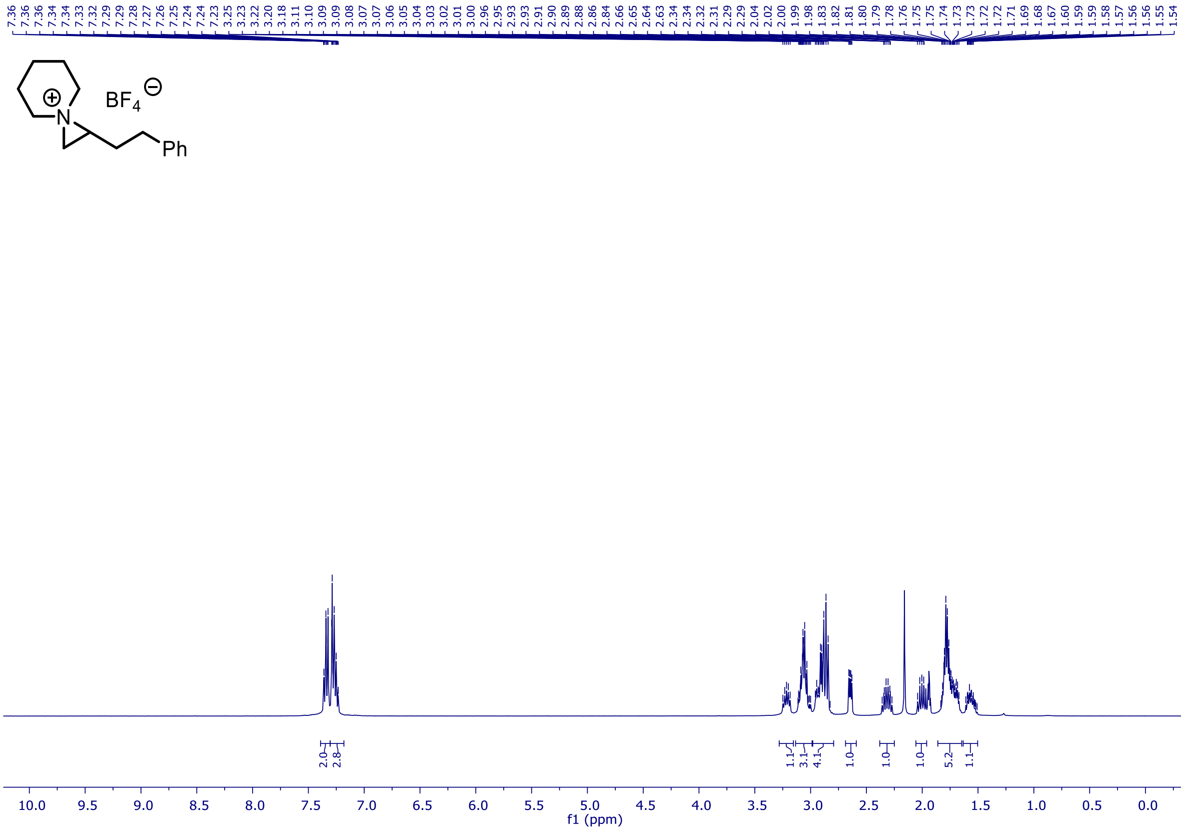

59 – ^{13}C NMR (100 MHz, CD_3CN)

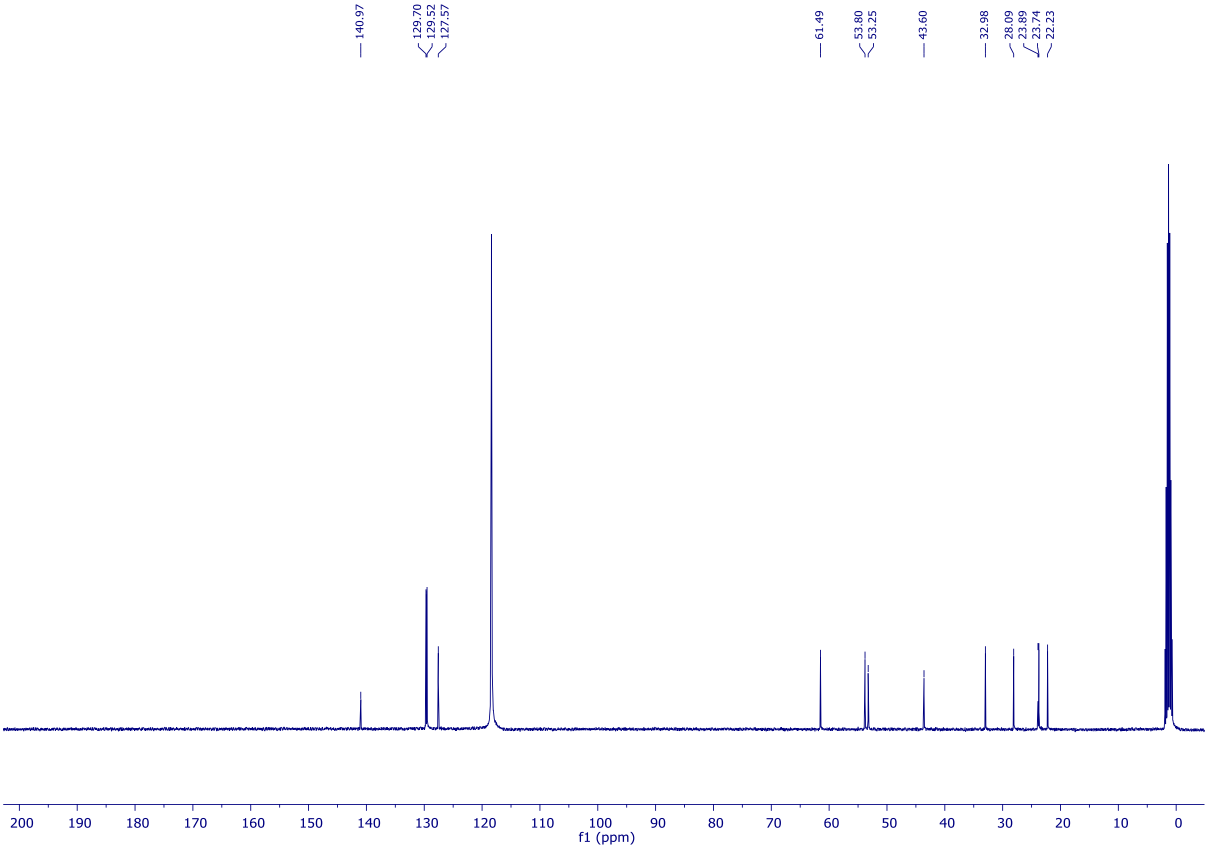

60 – ^{1}H NMR (500 MHz, $CDCl_3$)

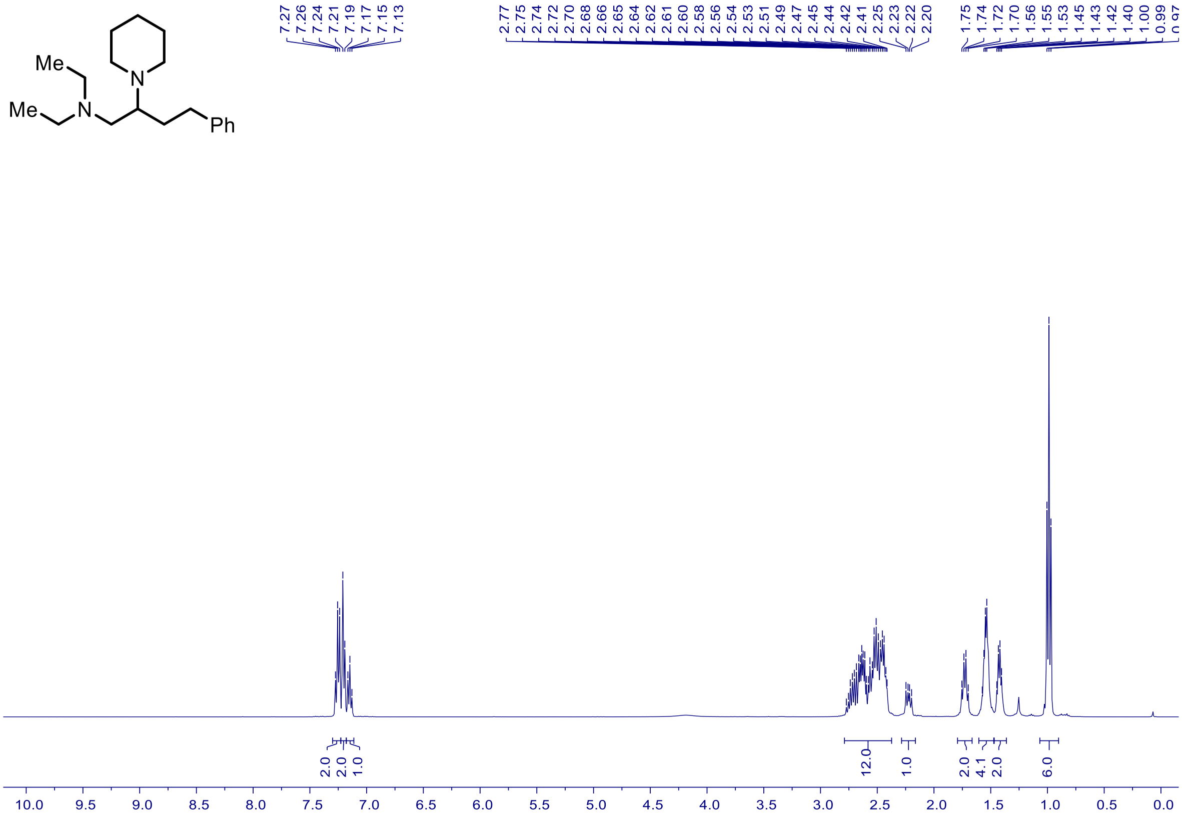

60 – ^{13}C NMR (125 MHz, $CDCl_3$)

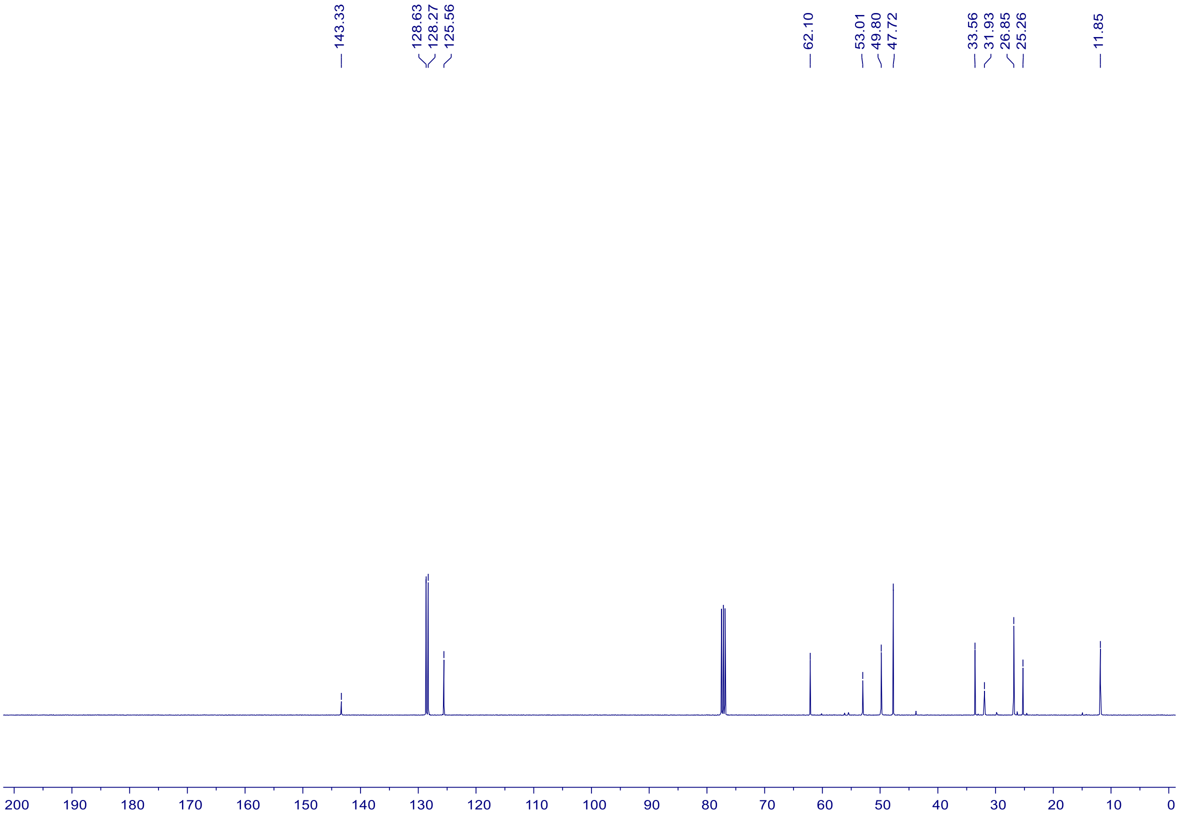

61 – ^{1}H NMR (500 MHz, $CDCl_3$)

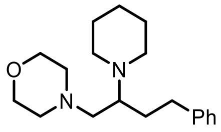

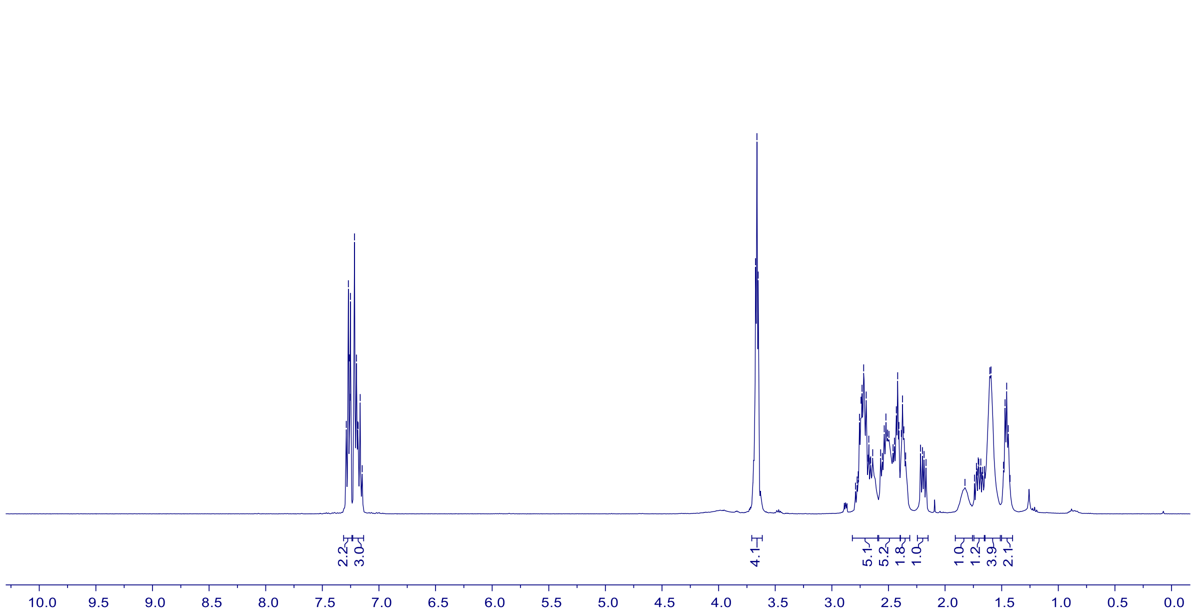

61 – ^{13}C NMR (126 MHz, $CDCl_3$)

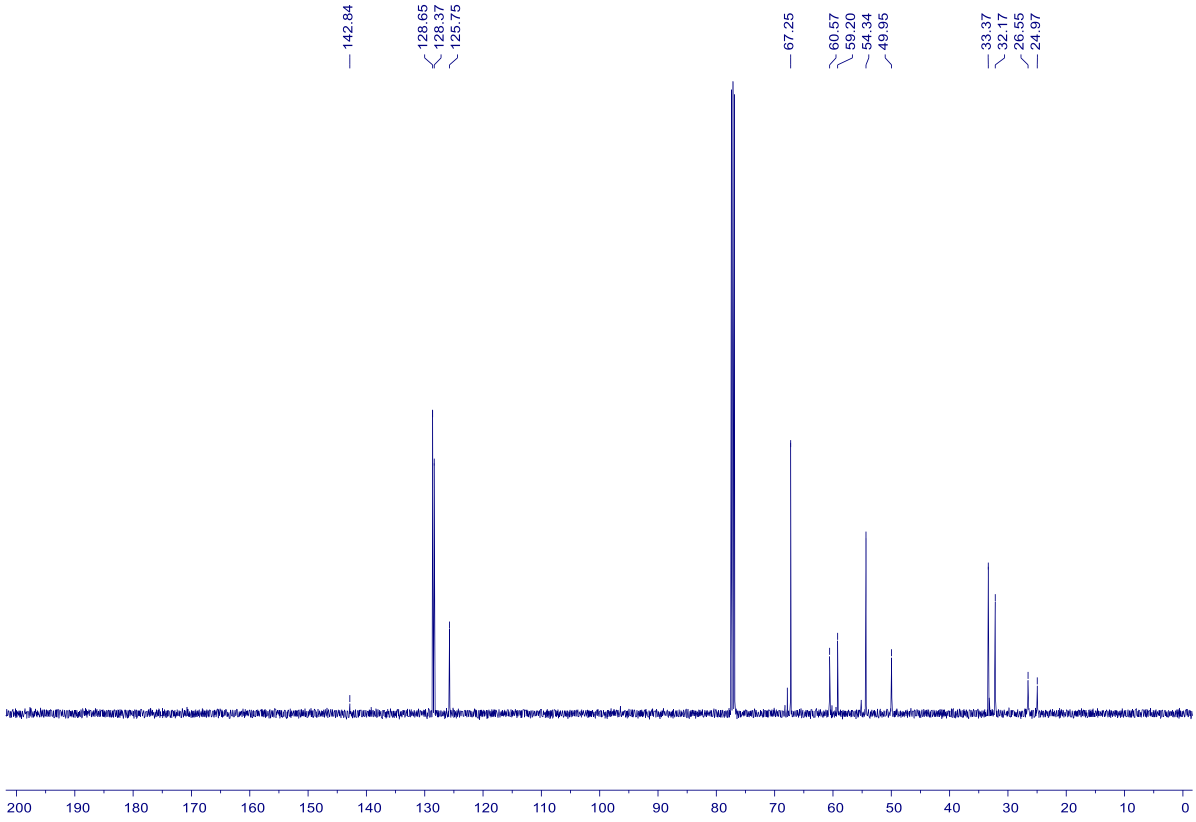

62 – ^{1}H NMR (500 MHz, CDCl$_3$)

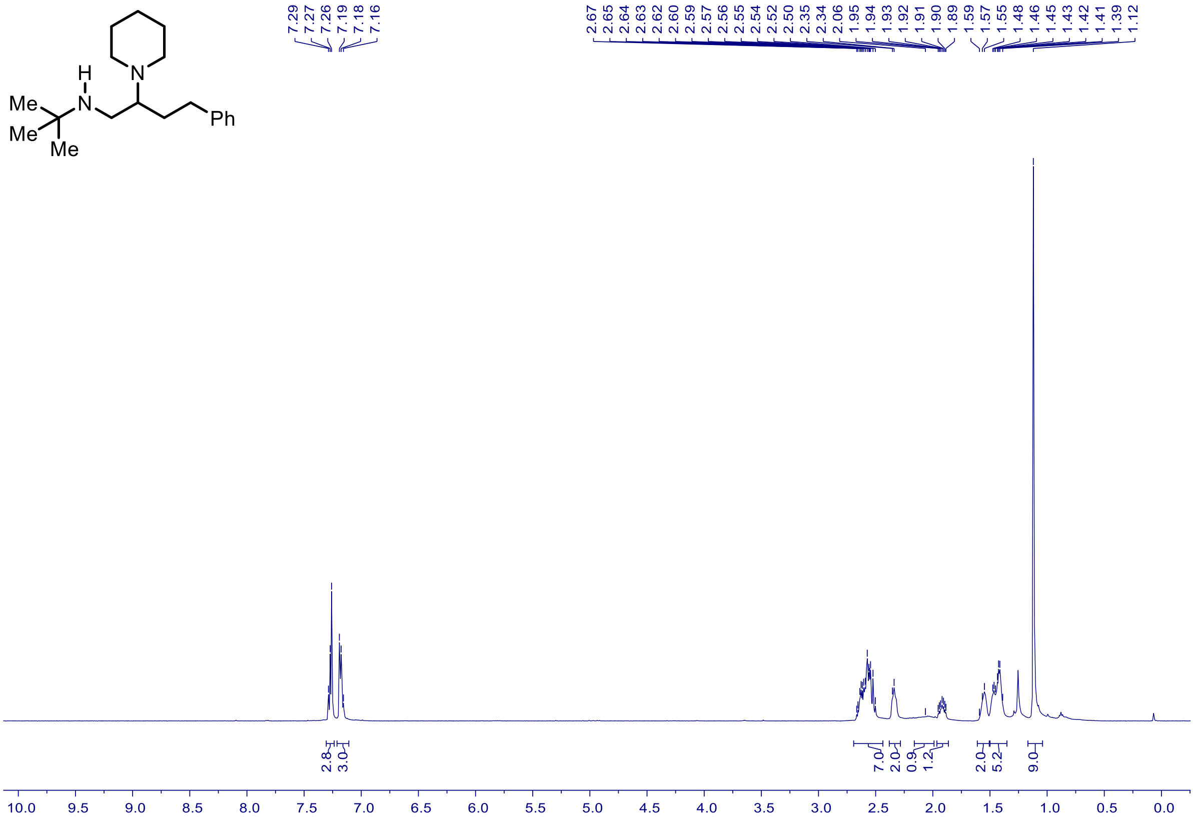

62 – ^{13}C NMR (125 MHz, CDCl$_3$)

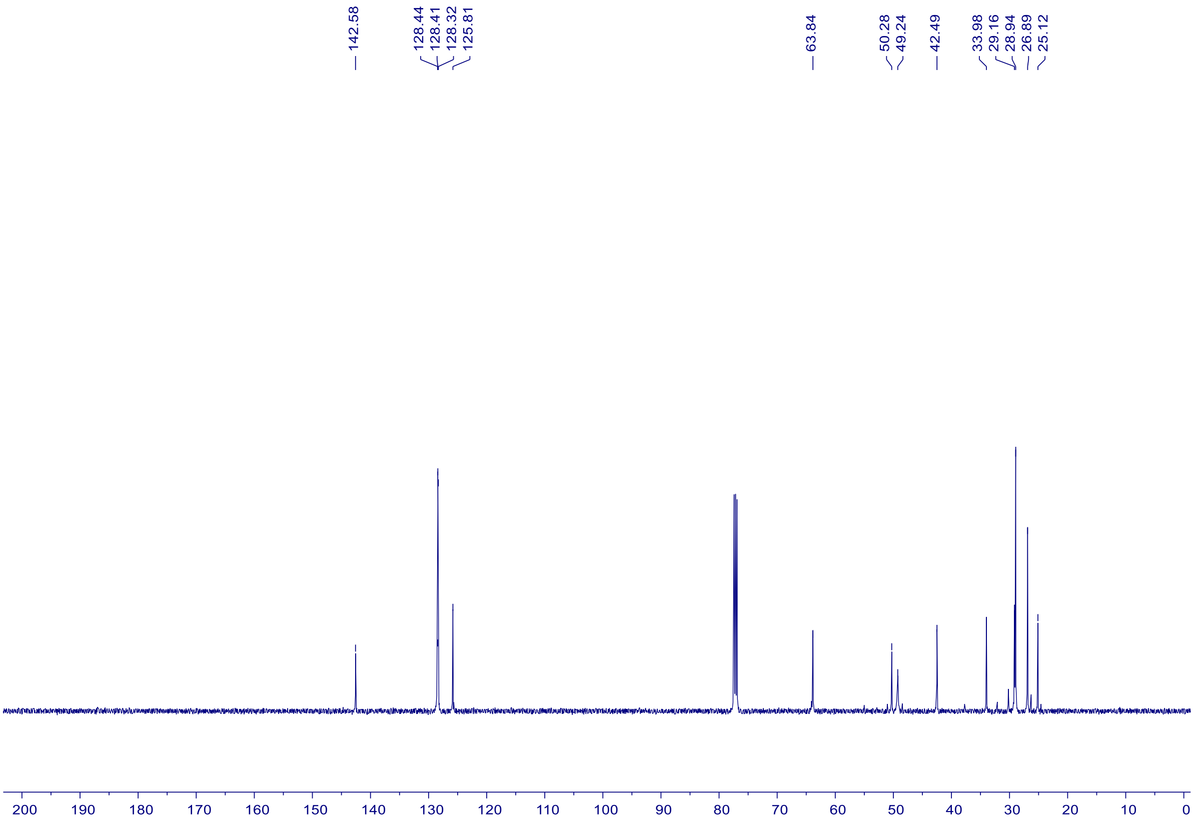

63 – ^{1}H NMR (500 MHz, $CDCl_3$)

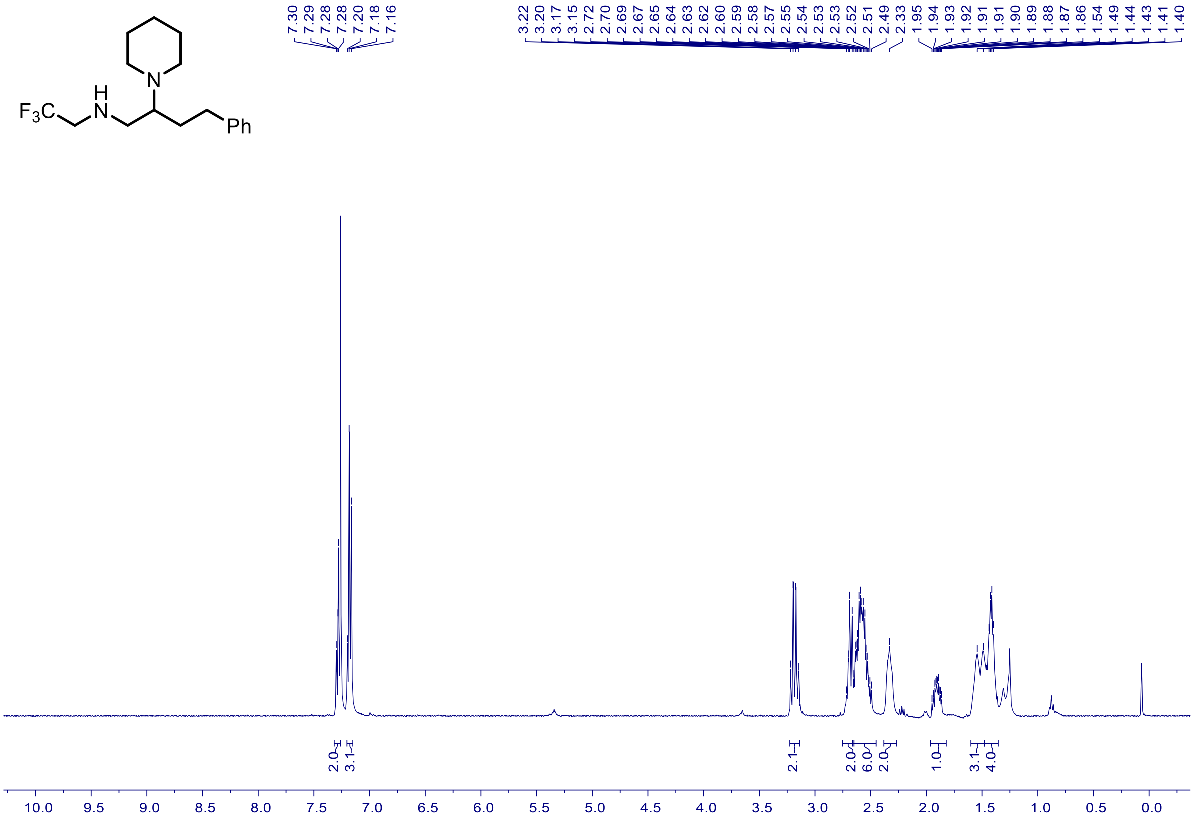

63 – ^{13}C NMR (126 MHz, $CDCl_3$)

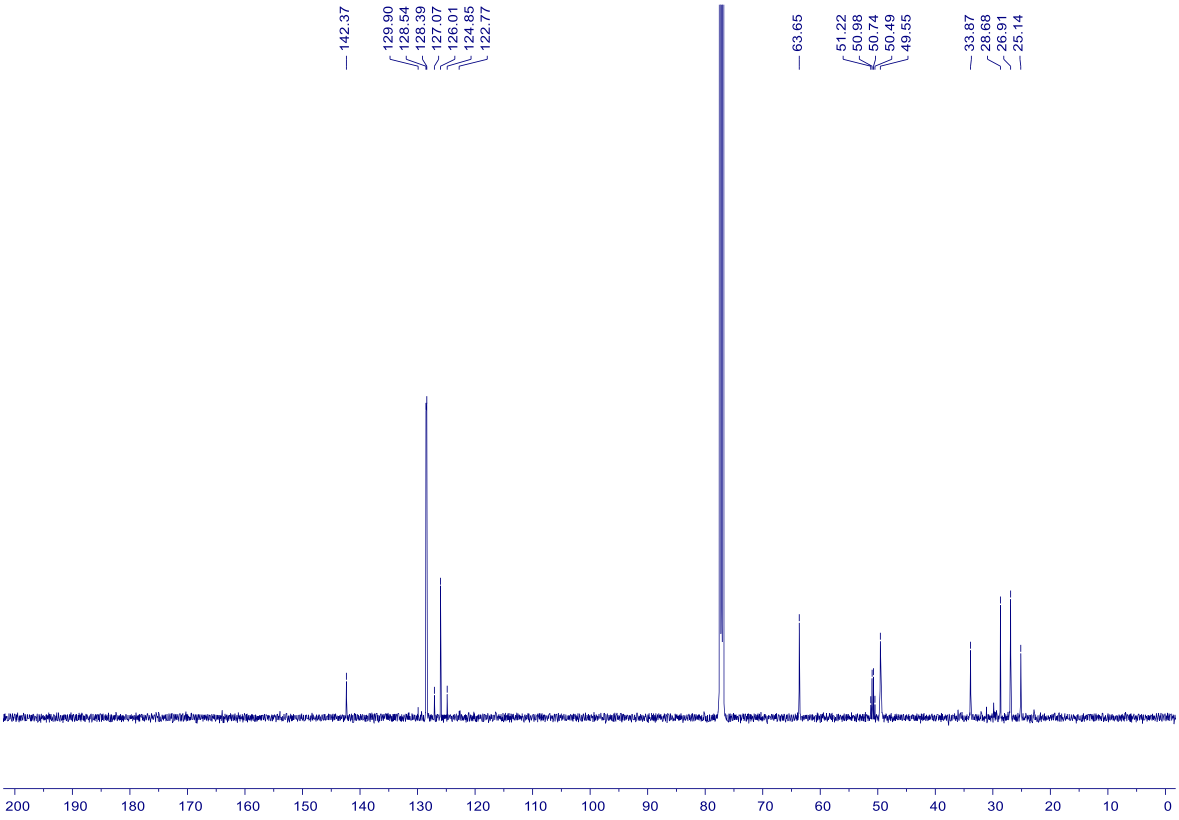

63 – ^{19}F NMR (376 MHz, $CDCl_3$)

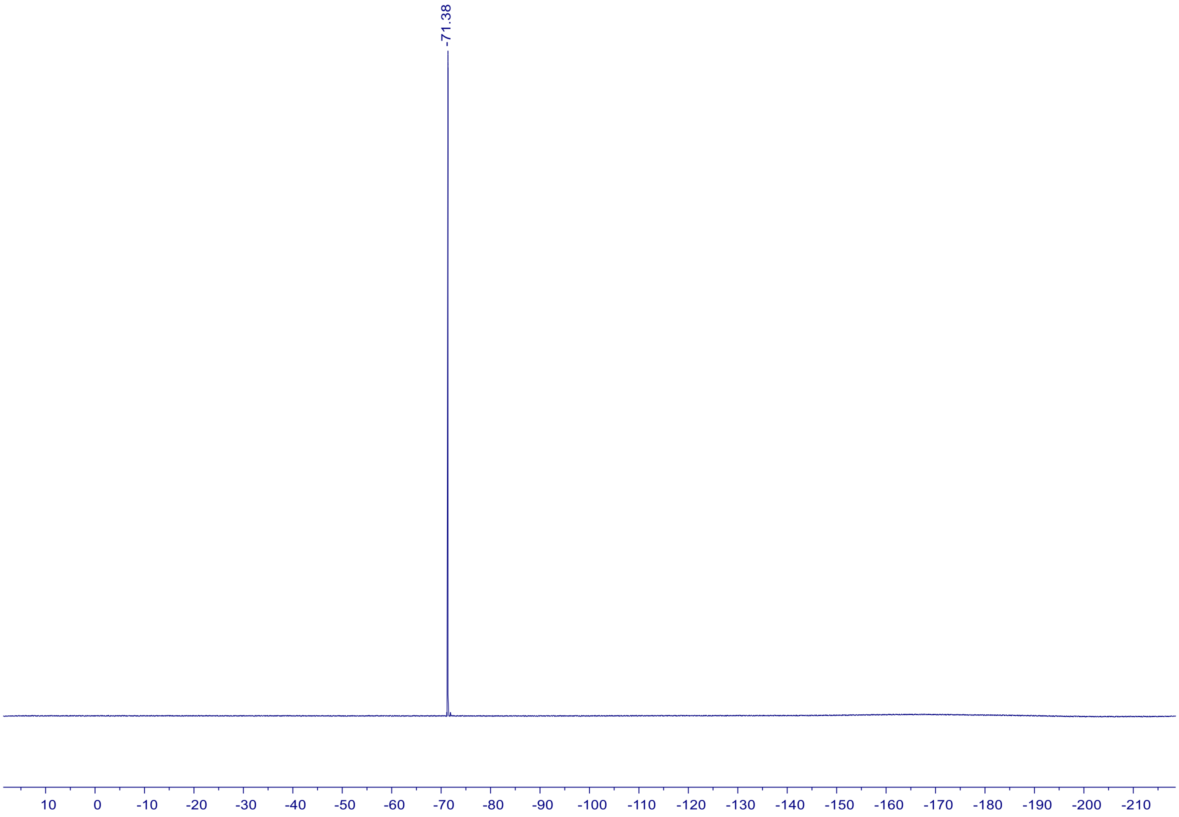

64 – ^{1}H NMR (500 MHz, CDCl$_3$)

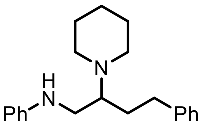

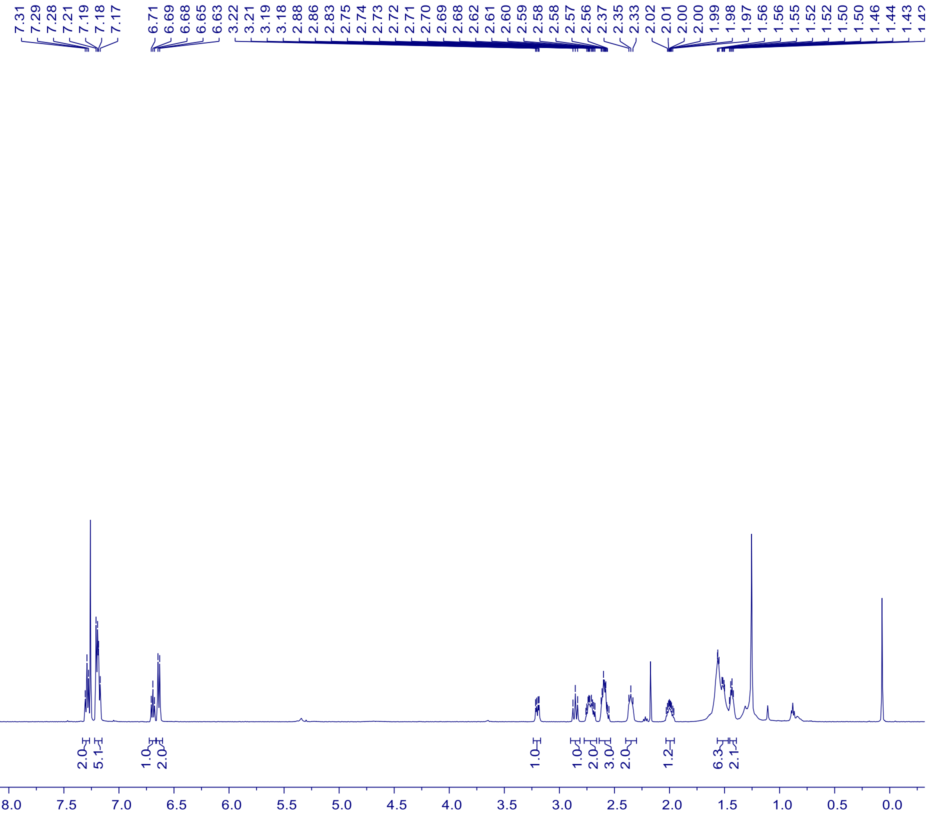

64 – ^{13}C NMR (126 MHz, CDCl$_3$)

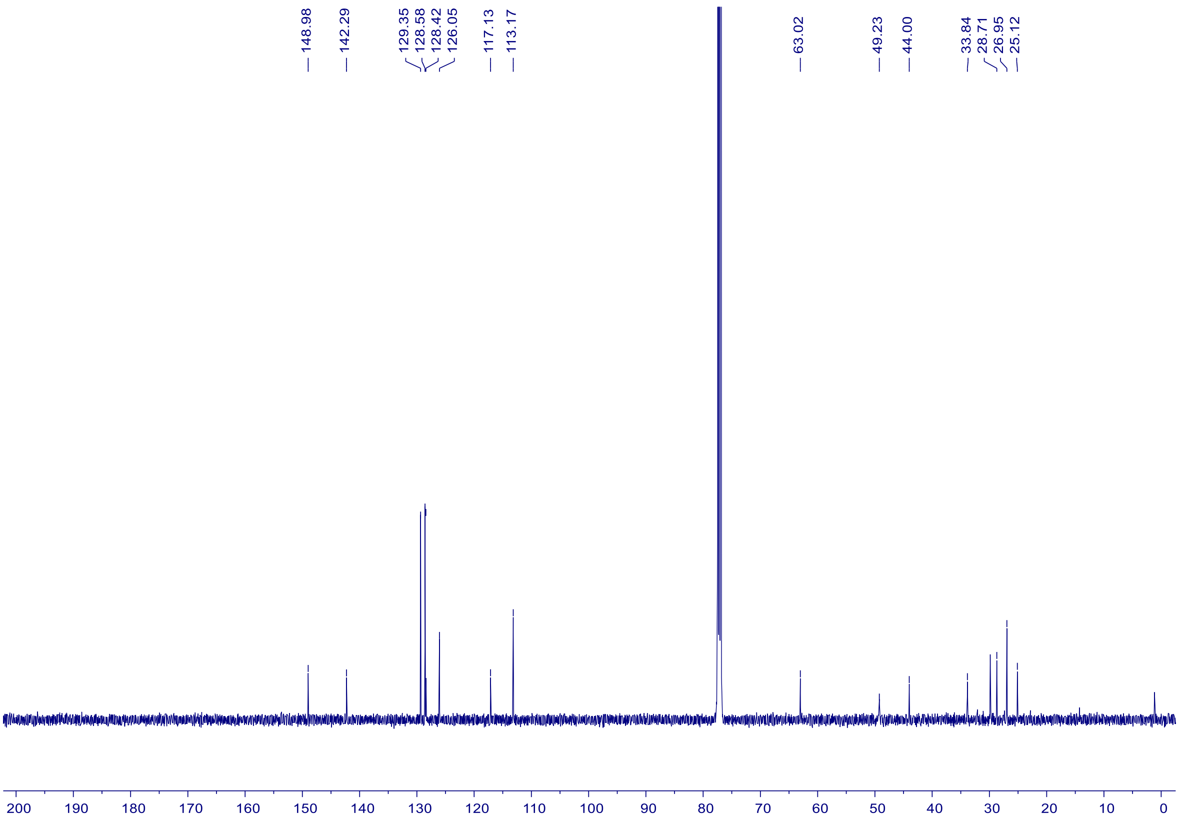

64’ – ^{1}H NMR (500 MHz, $CDCl_3$)

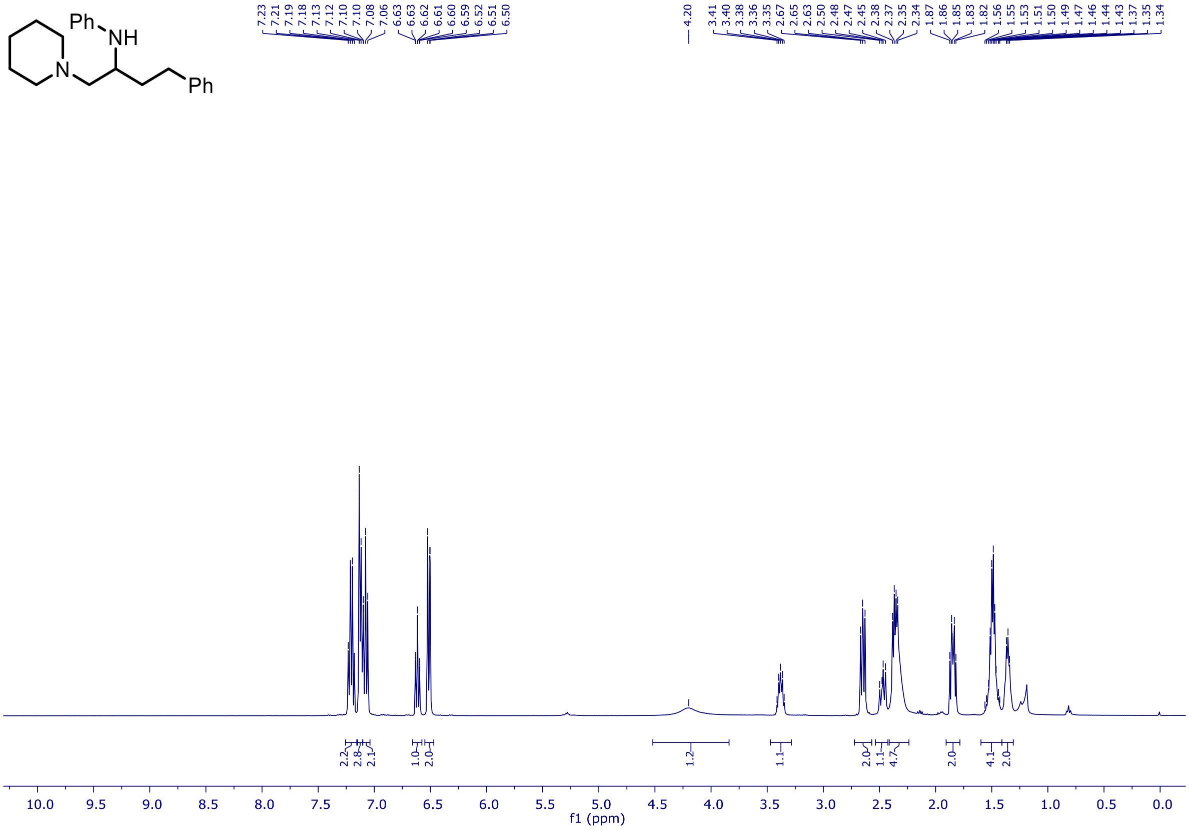

64’ – ^{13}C NMR (125 MHz, $CDCl_3$)

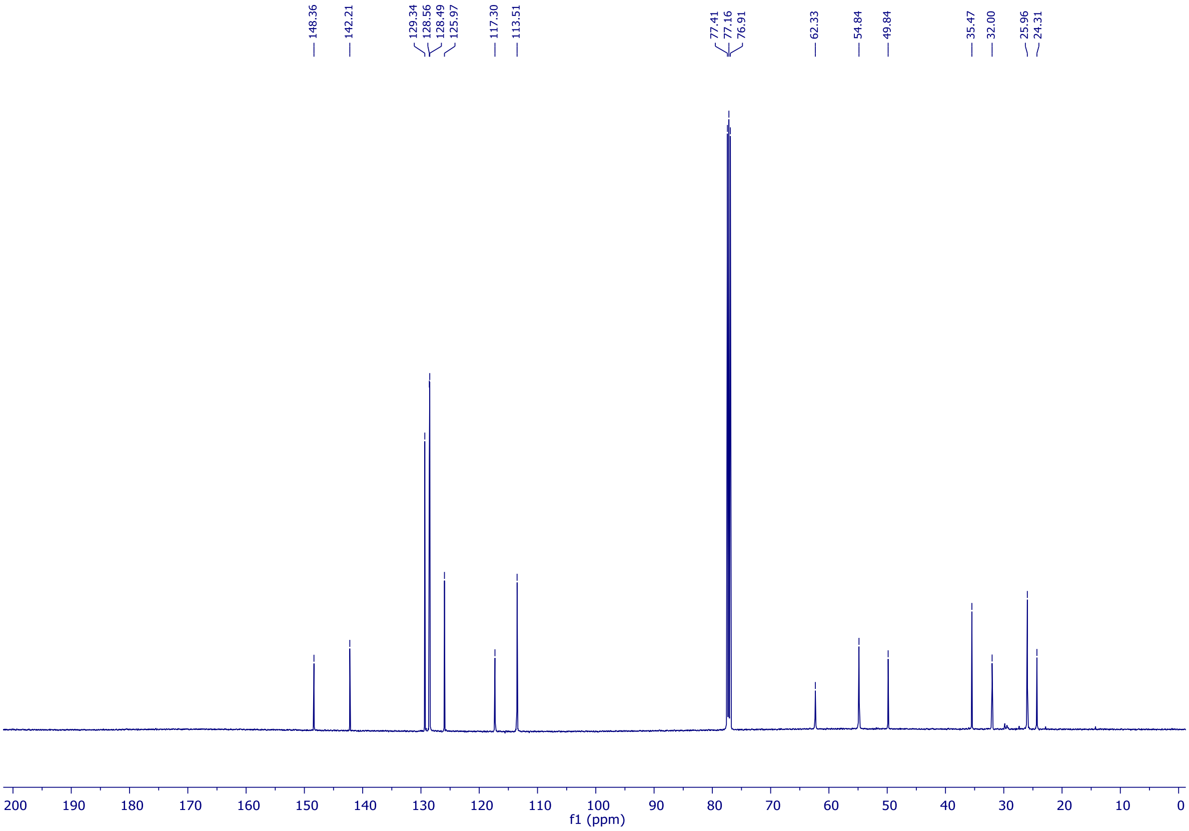

65 – ^{1}H NMR (500 MHz, $CDCl_3$)

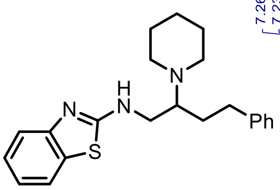

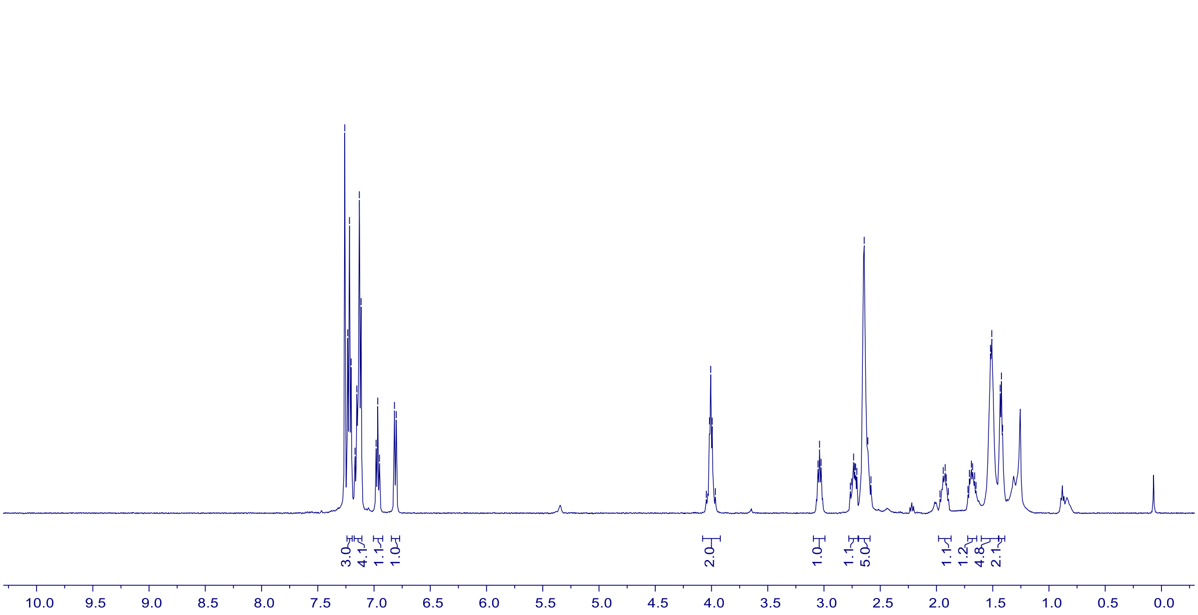

65 – ^{13}C NMR (126 MHz, $CDCl_3$)

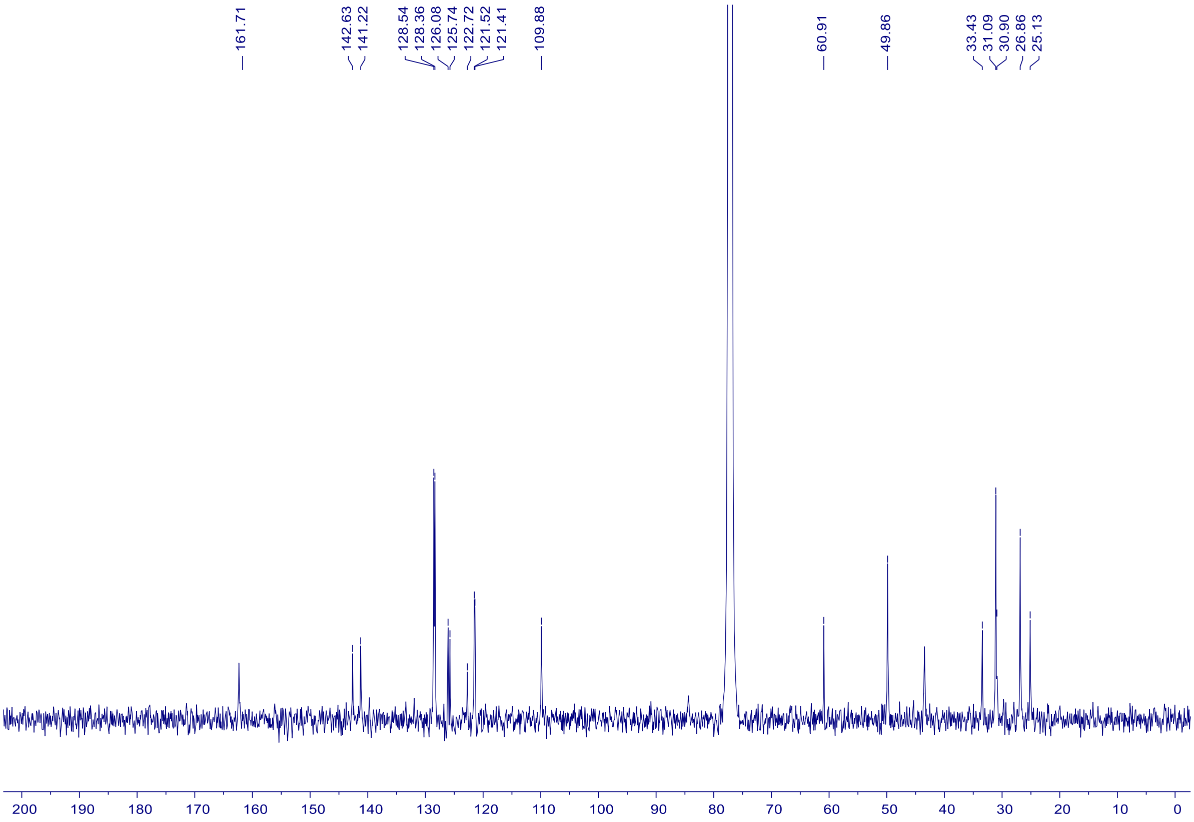

66 – ^{1}H NMR (500 MHz, $CDCl_3$)

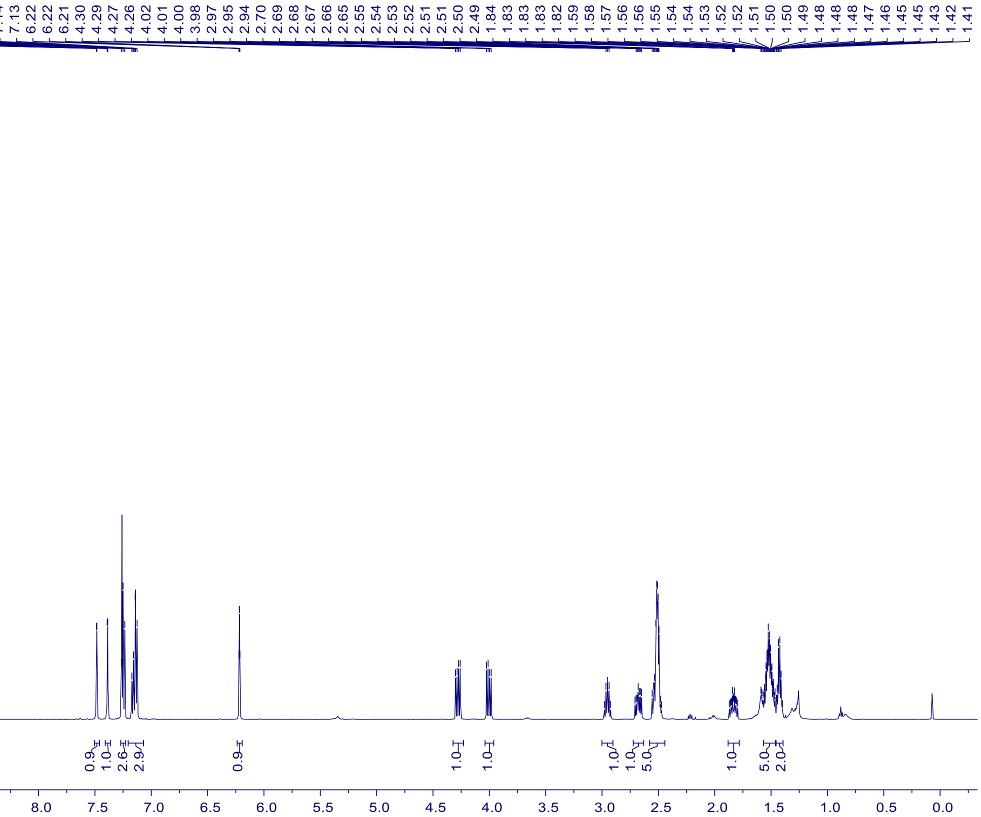

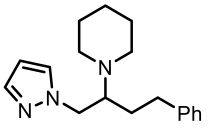

66 – ^{13}C NMR (126 MHz, $CDCl_3$)

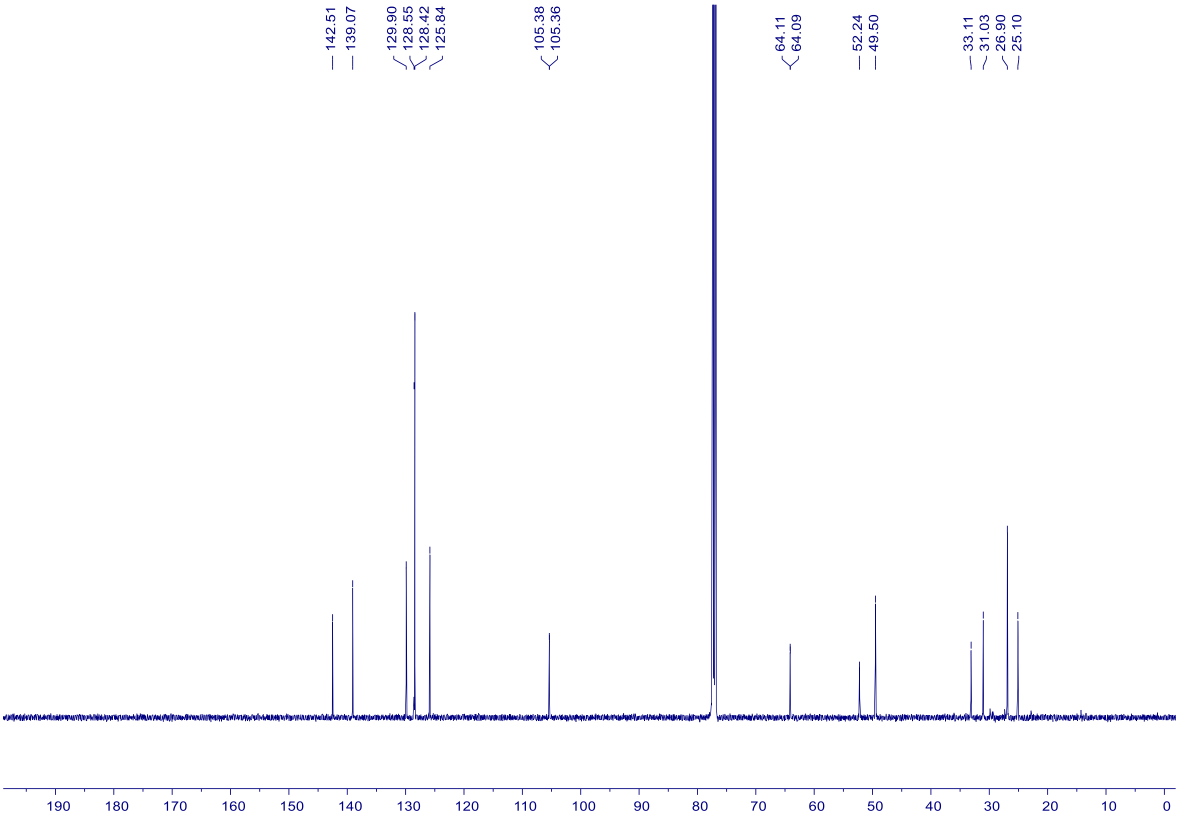

67 – ^{1}H NMR (500 MHz, CDCl$_3$)

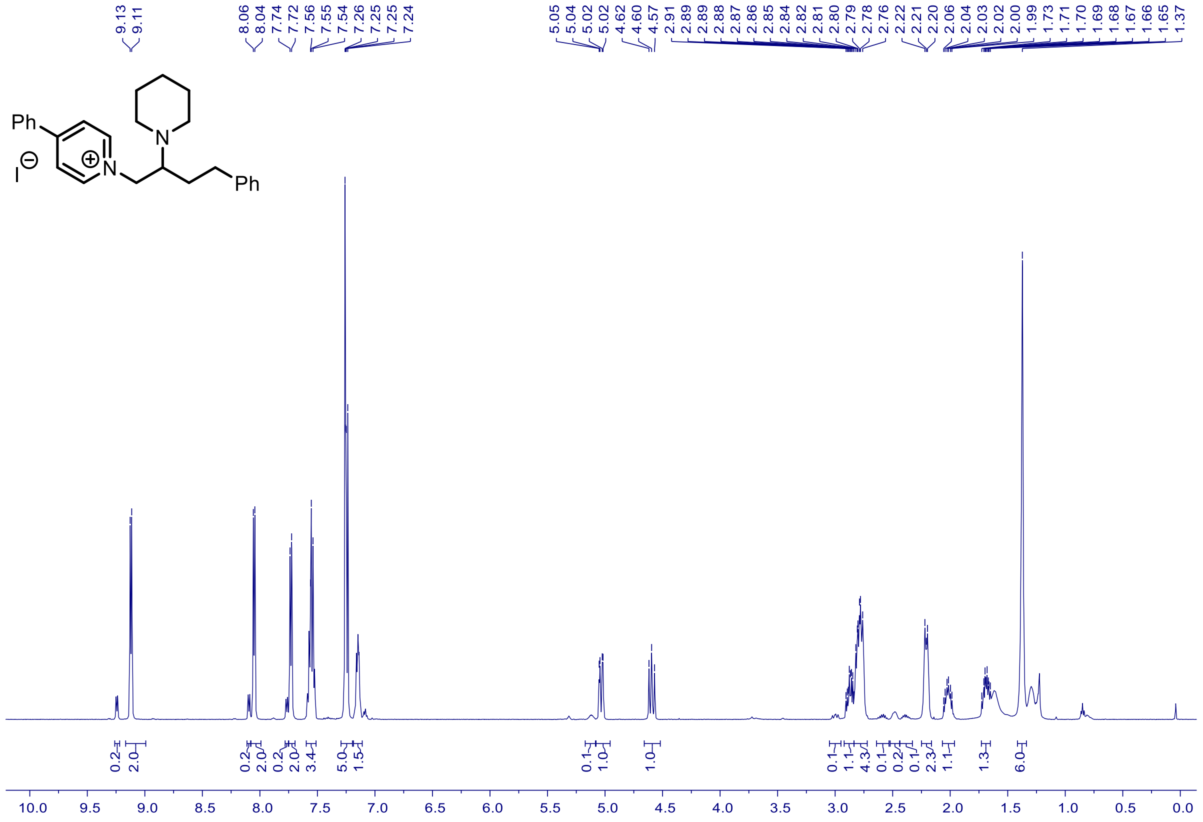

67 – ^{13}C NMR (126 MHz, CDCl$_3$)

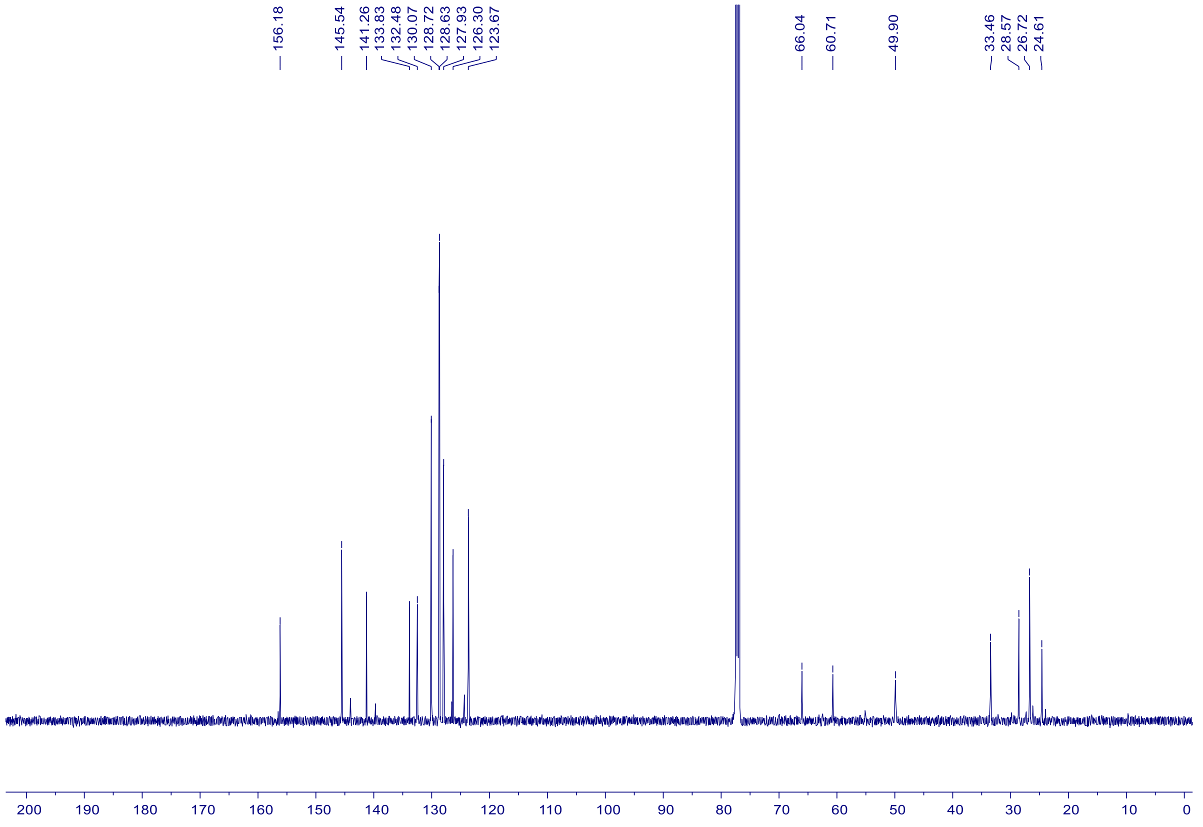

68 – ^{1}H NMR (500 MHz, $CDCl_3$)

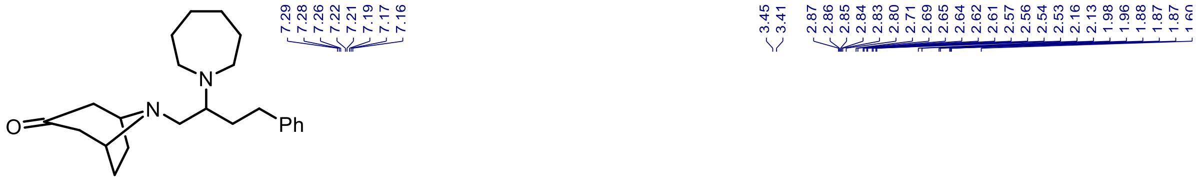

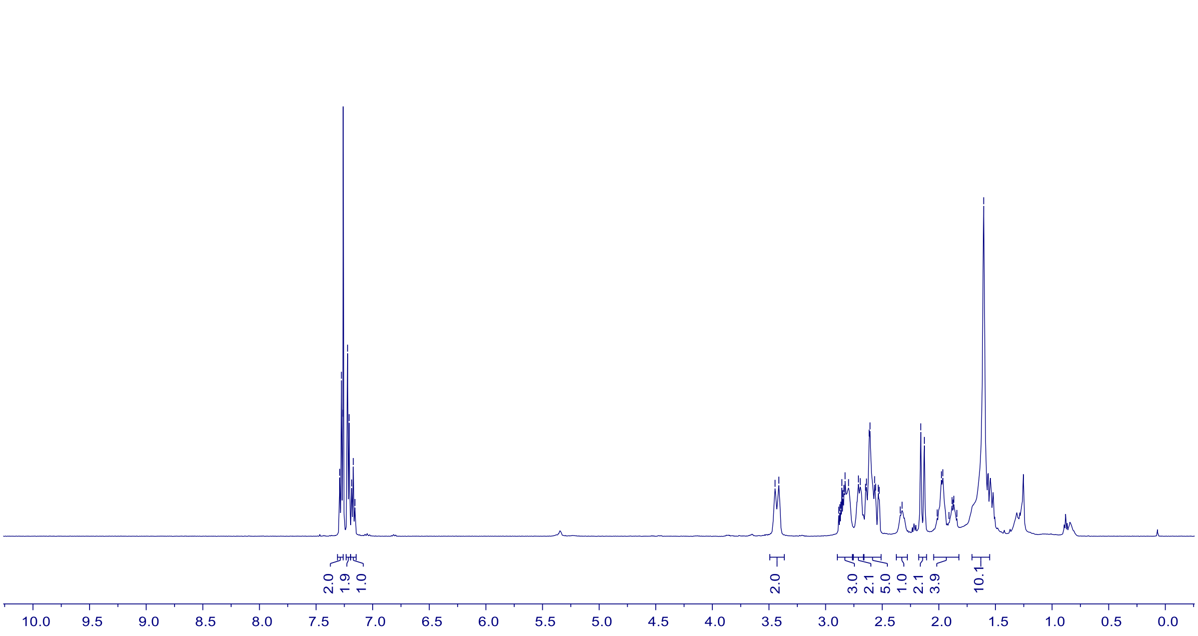

68 – ^{13}C NMR (126 MHz, $CDCl_3$)

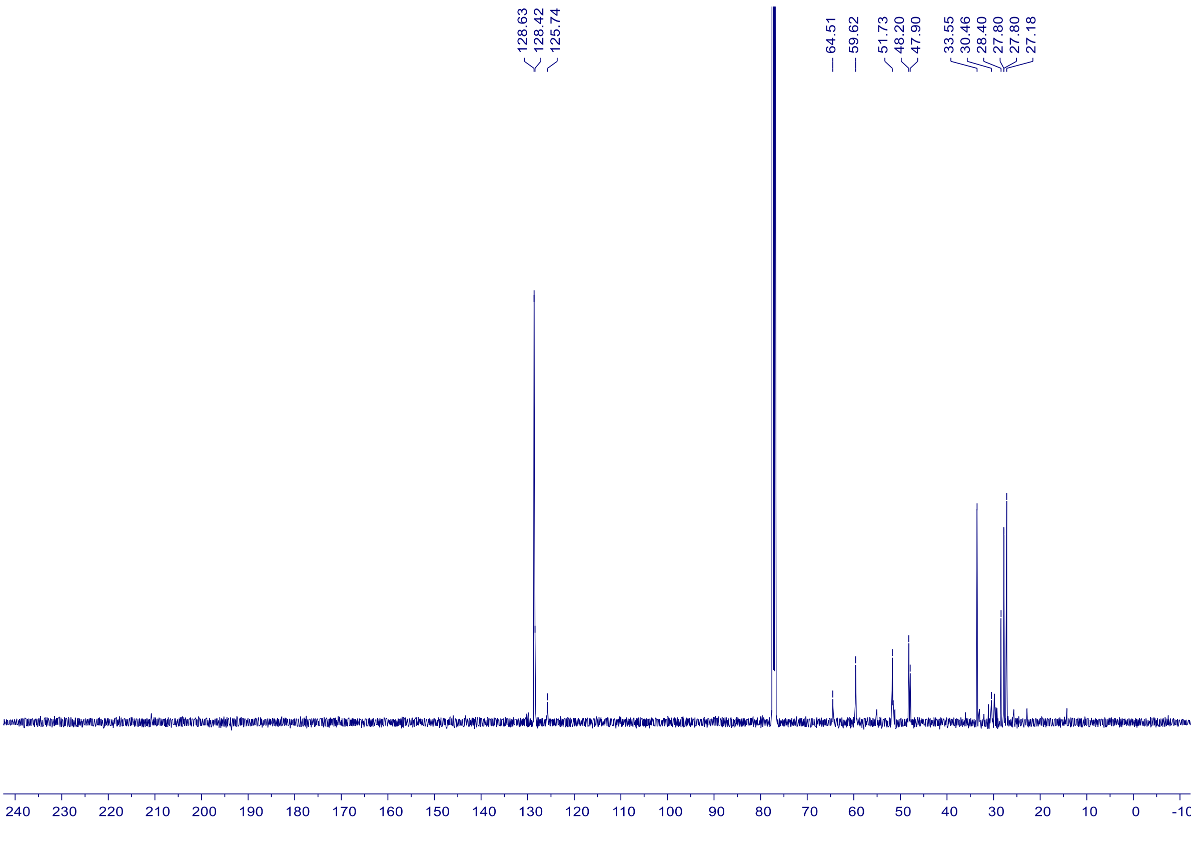

69 – ^{1}H NMR (500 MHz, $CDCl_3$)

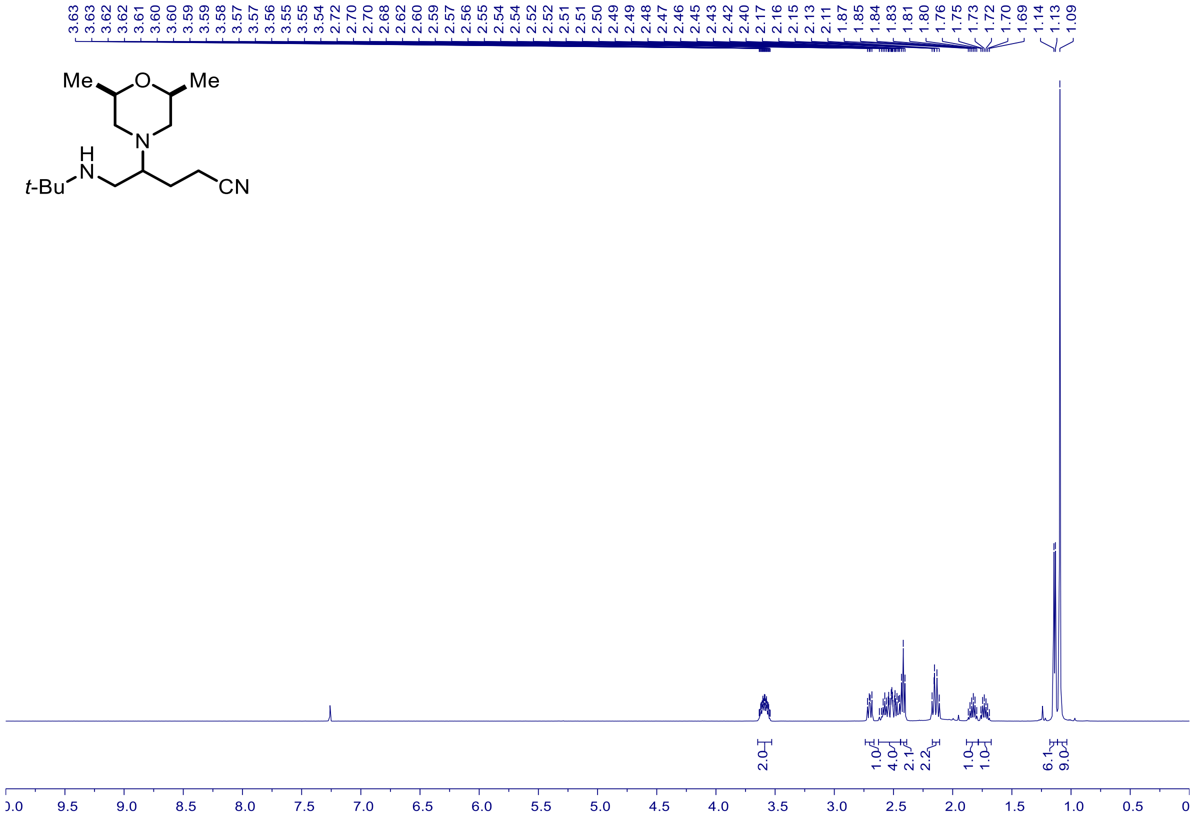

69 – ^{13}C NMR (126 MHz, $CDCl_3$)

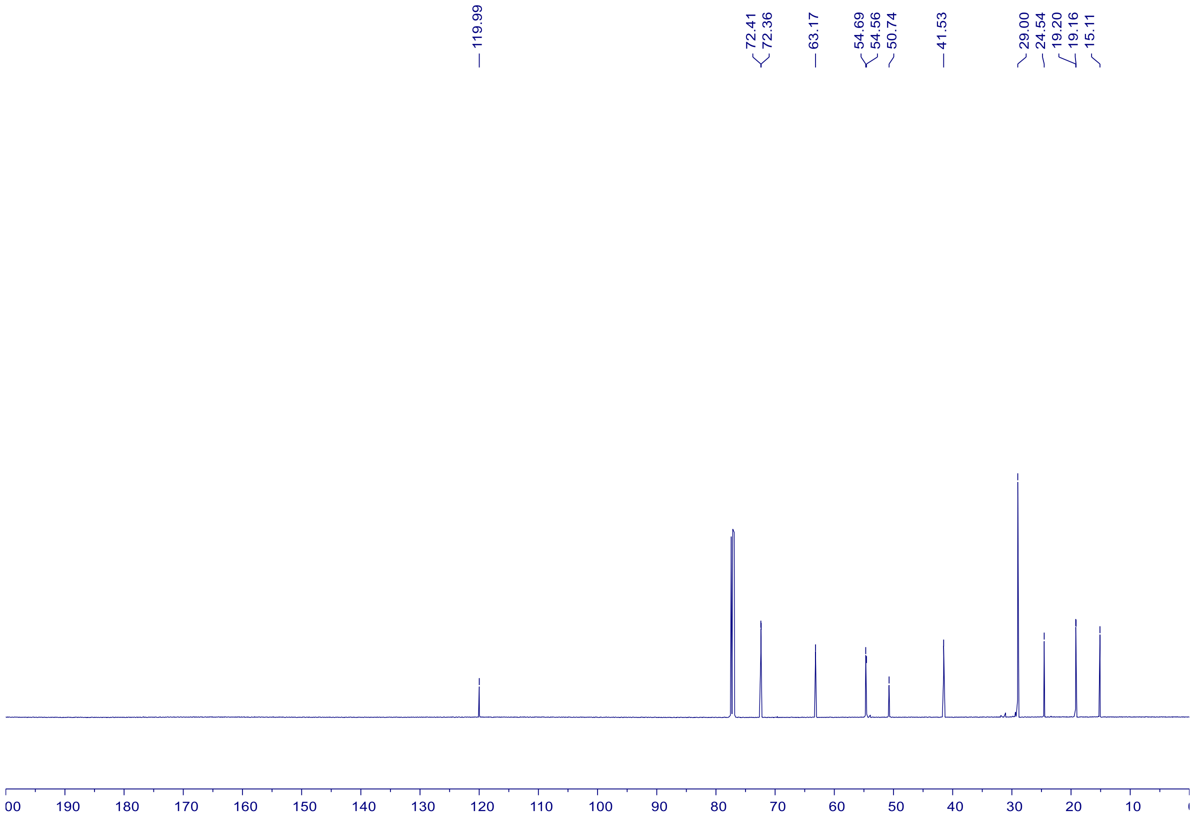

70 – ^{1}H NMR (500 MHz, $CDCl_3$)

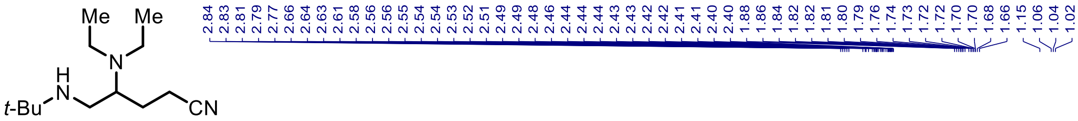

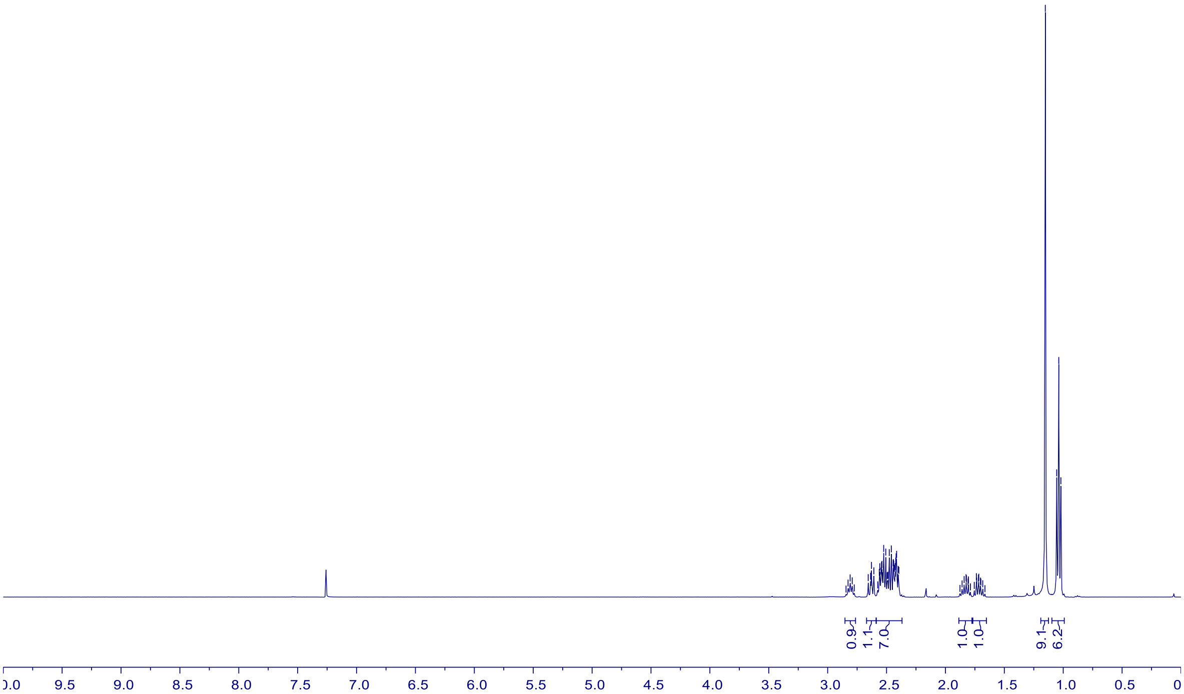

70 – ^{13}C NMR (126 MHz, $CDCl_3$)

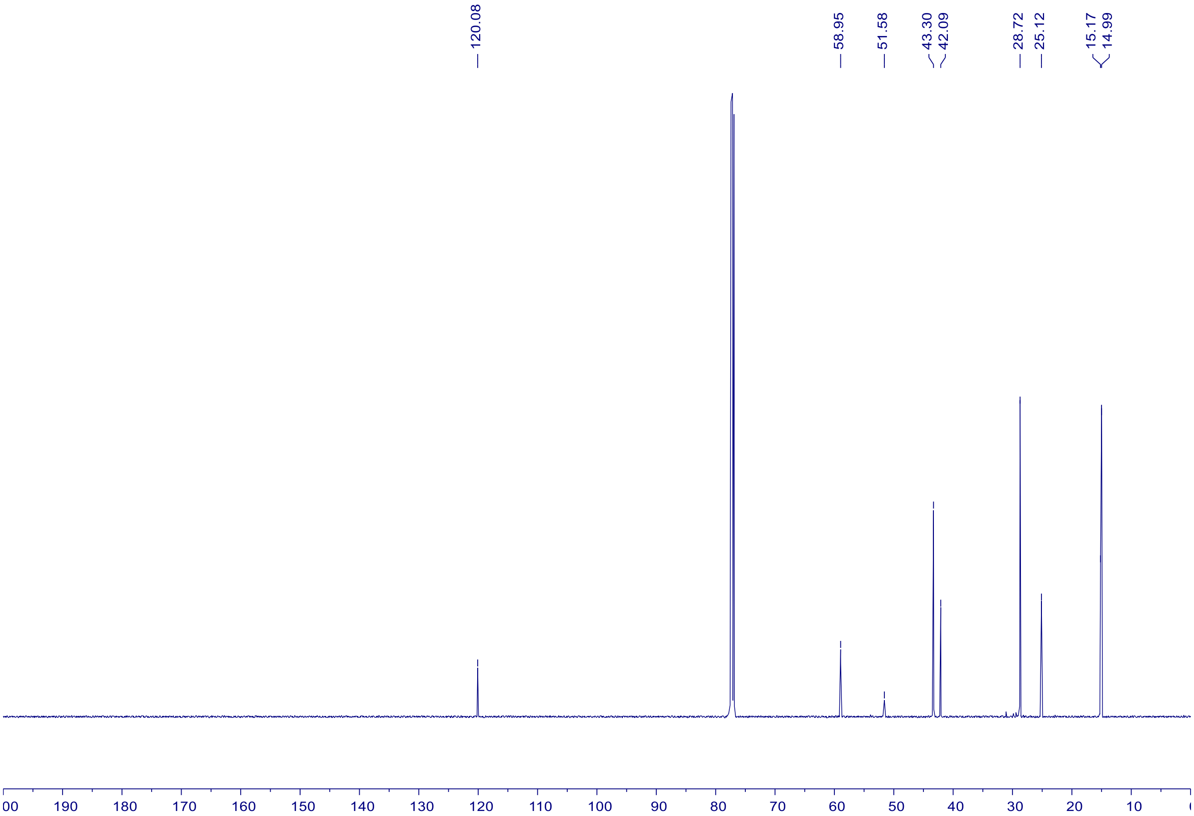

71 – ^{1}H NMR (500 MHz, CDCl$_3$)

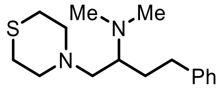

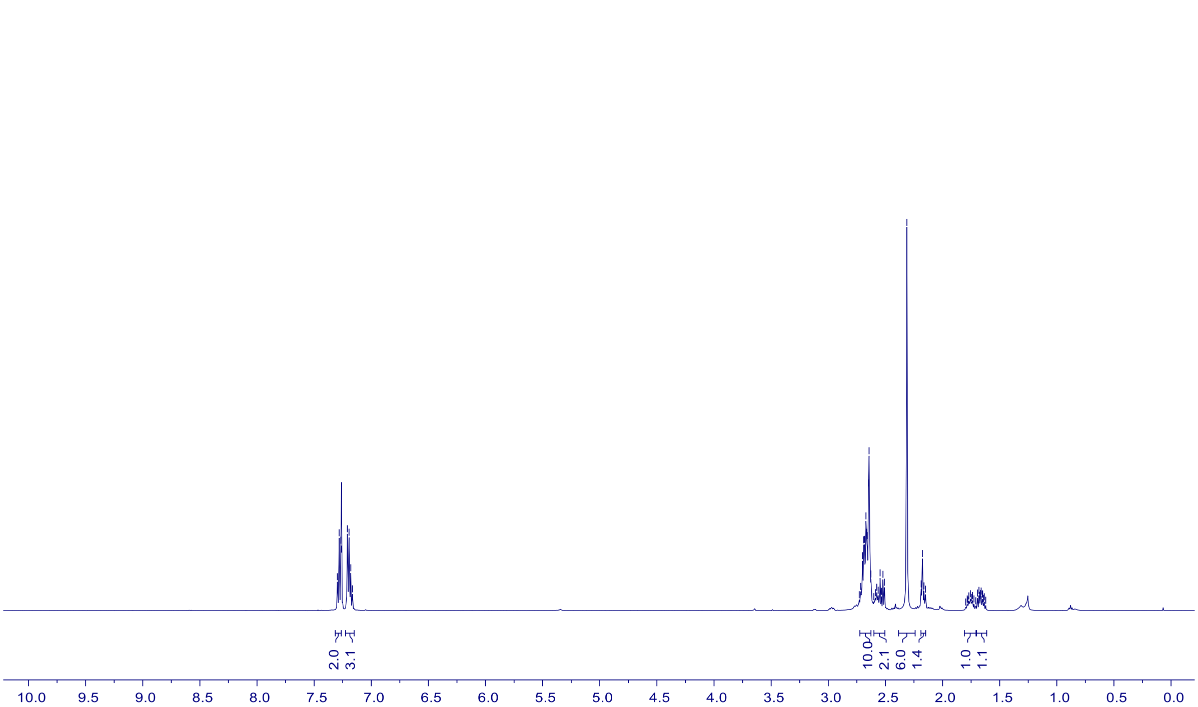

71 – ^{13}C NMR (126 MHz, CDCl$_3$)

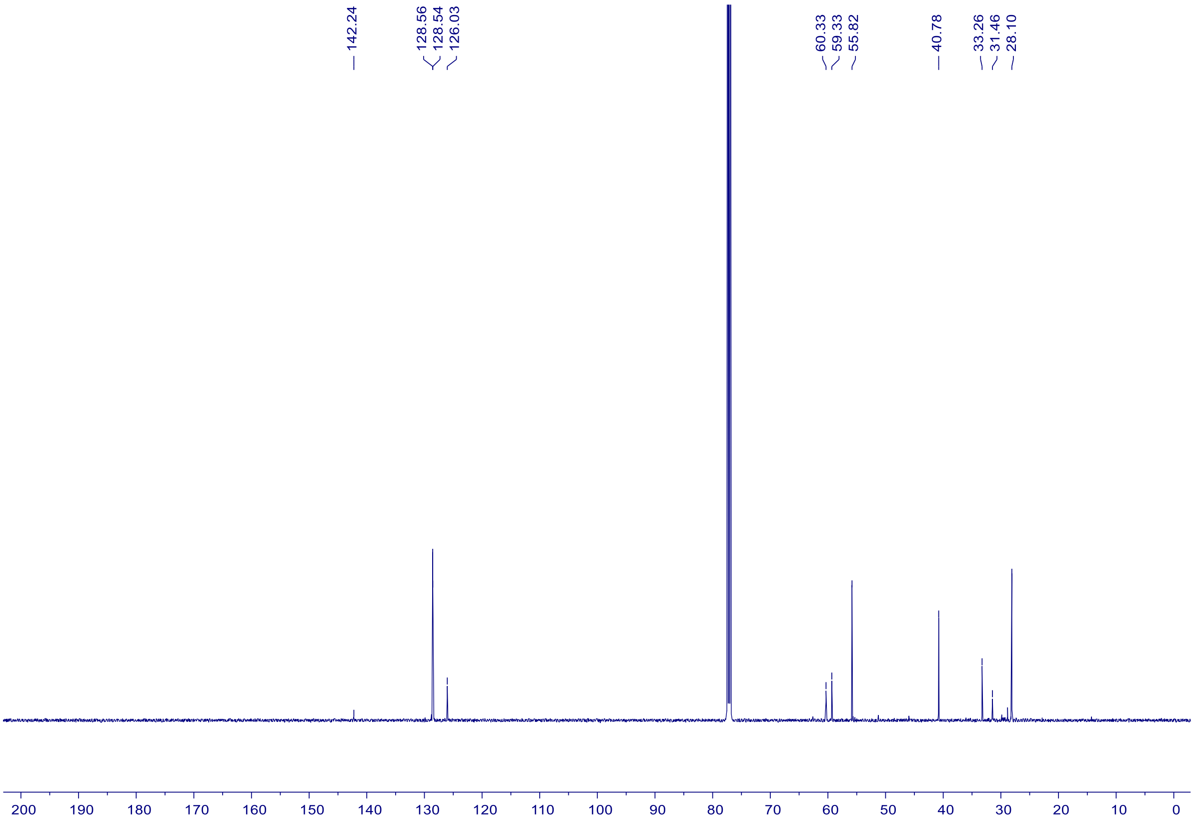

71 – ^{1}H–^{13}C HMBC (500 MHz, $CDCl_3$)

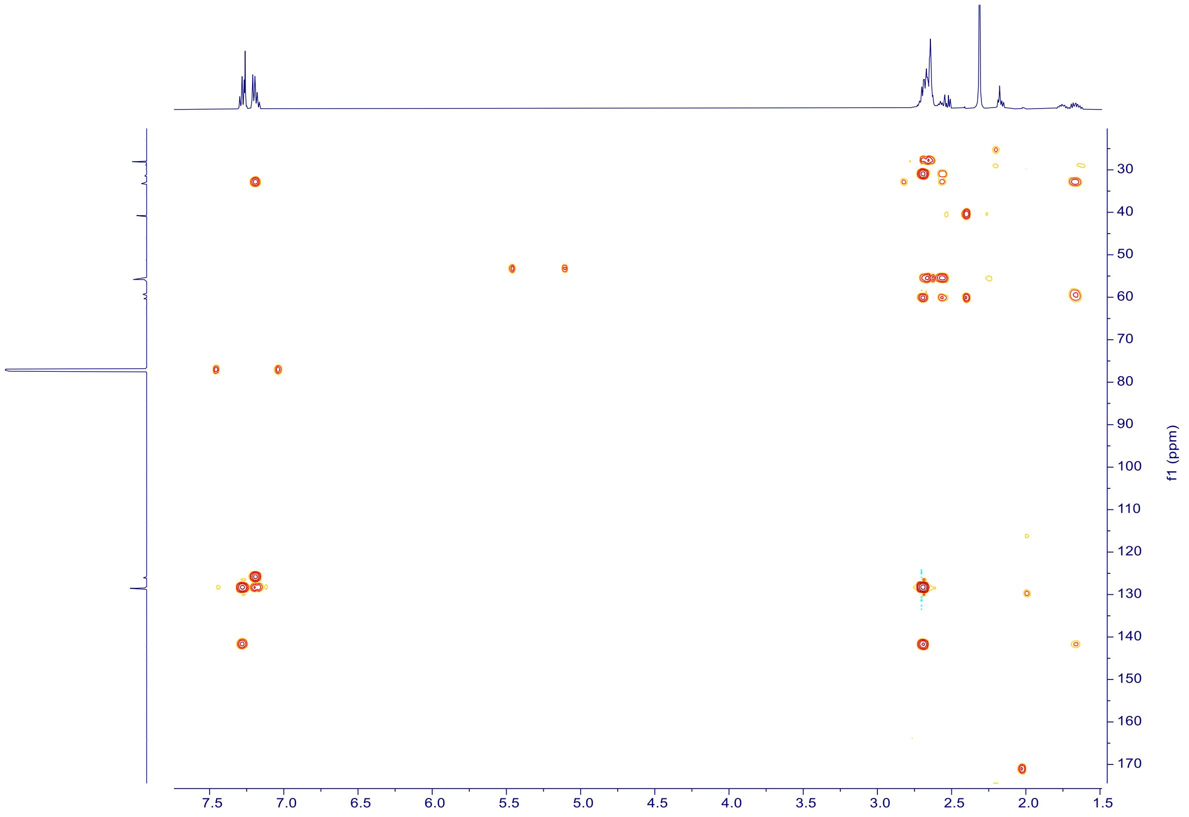

72 – ^{1}H NMR (500 MHz, $CDCl_3$)

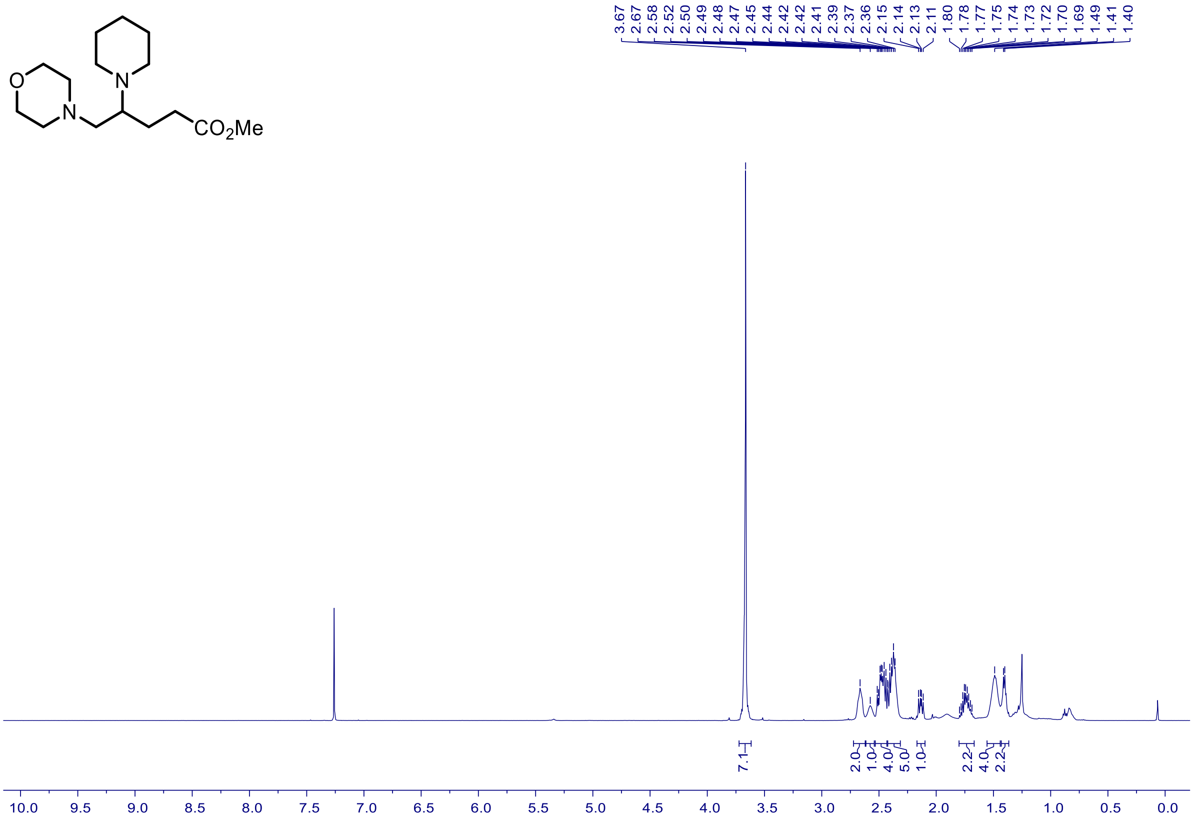

72 – ^{13}C NMR (126 MHz, $CDCl_3$)

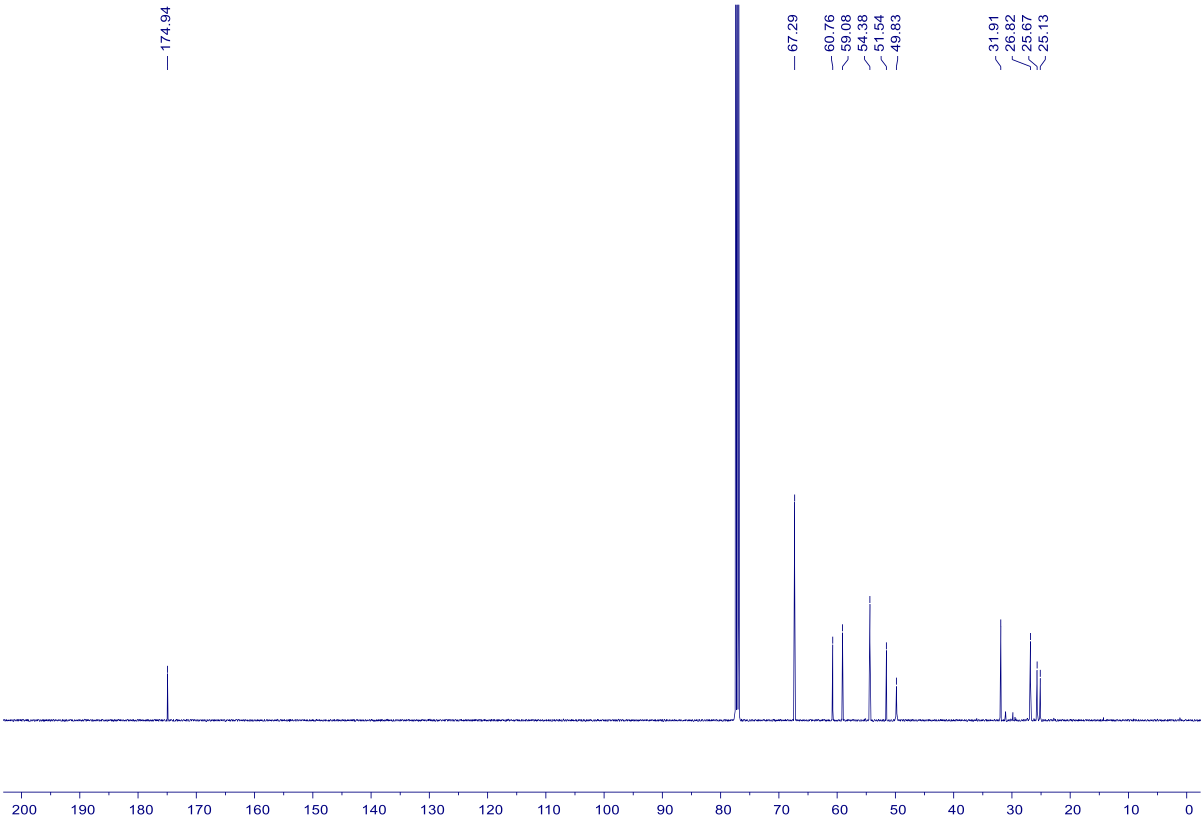

73 – ^{1}H NMR (500 MHz, CDCl$_3$)

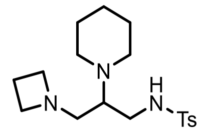

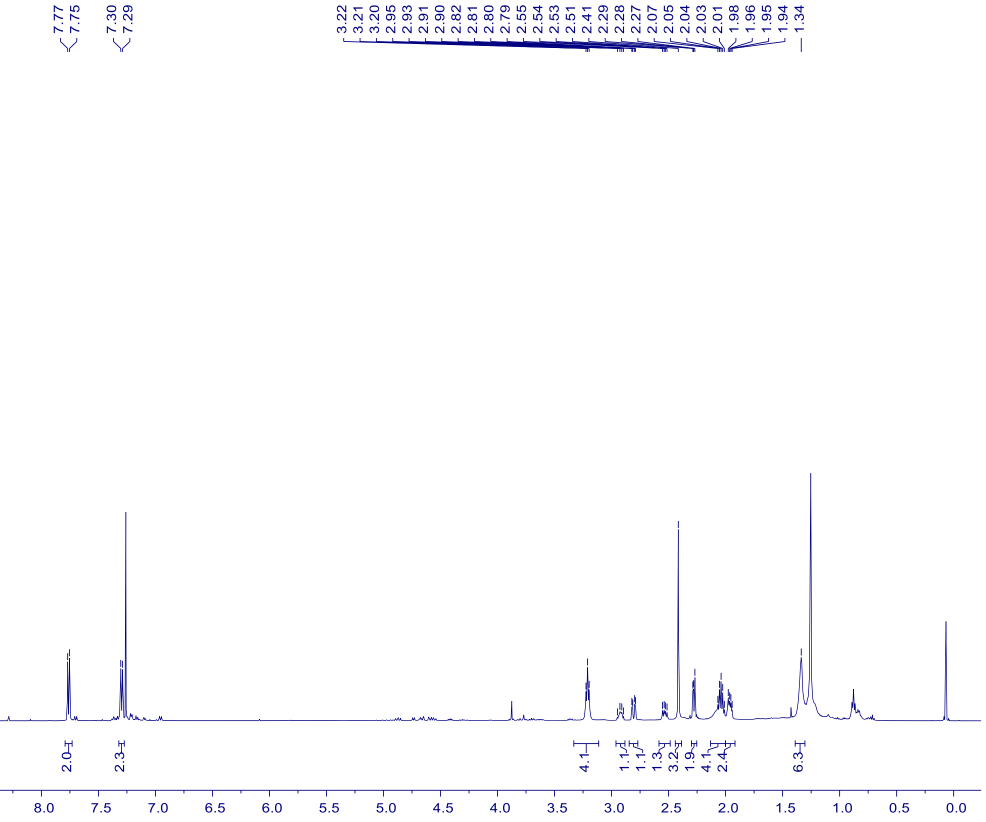

73 – ^{13}C NMR (125 MHz, CDCl$_3$)

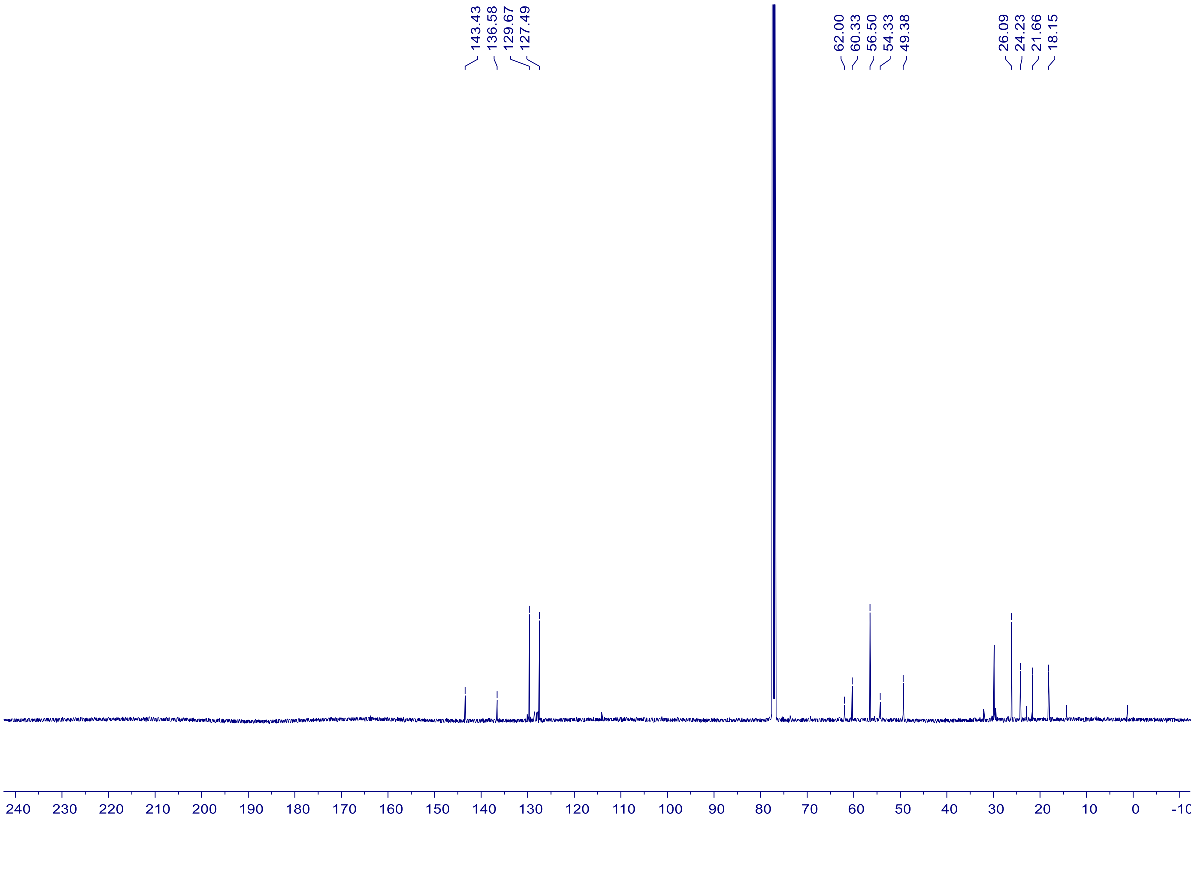

74 – ^{1}H NMR (500 MHz, $CDCl_3$)

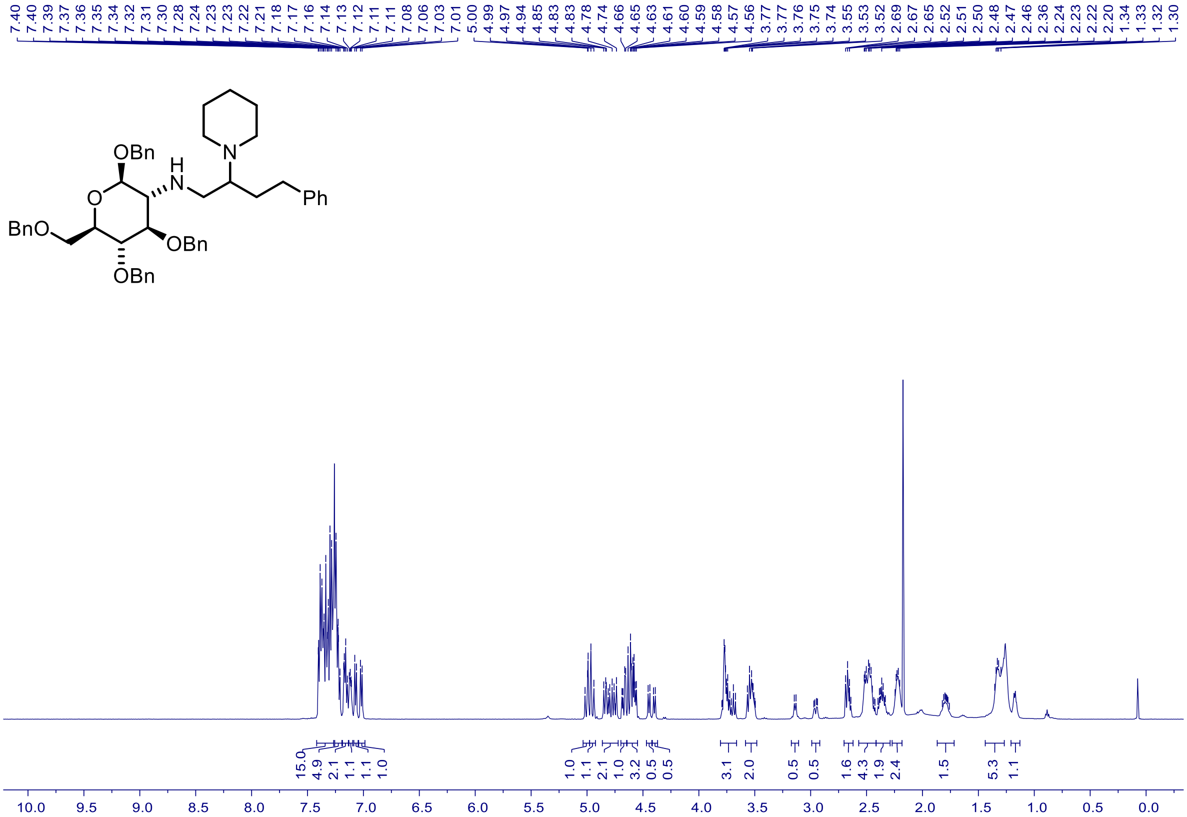

74 – ^{13}C NMR (126 MHz, $CDCl_3$)

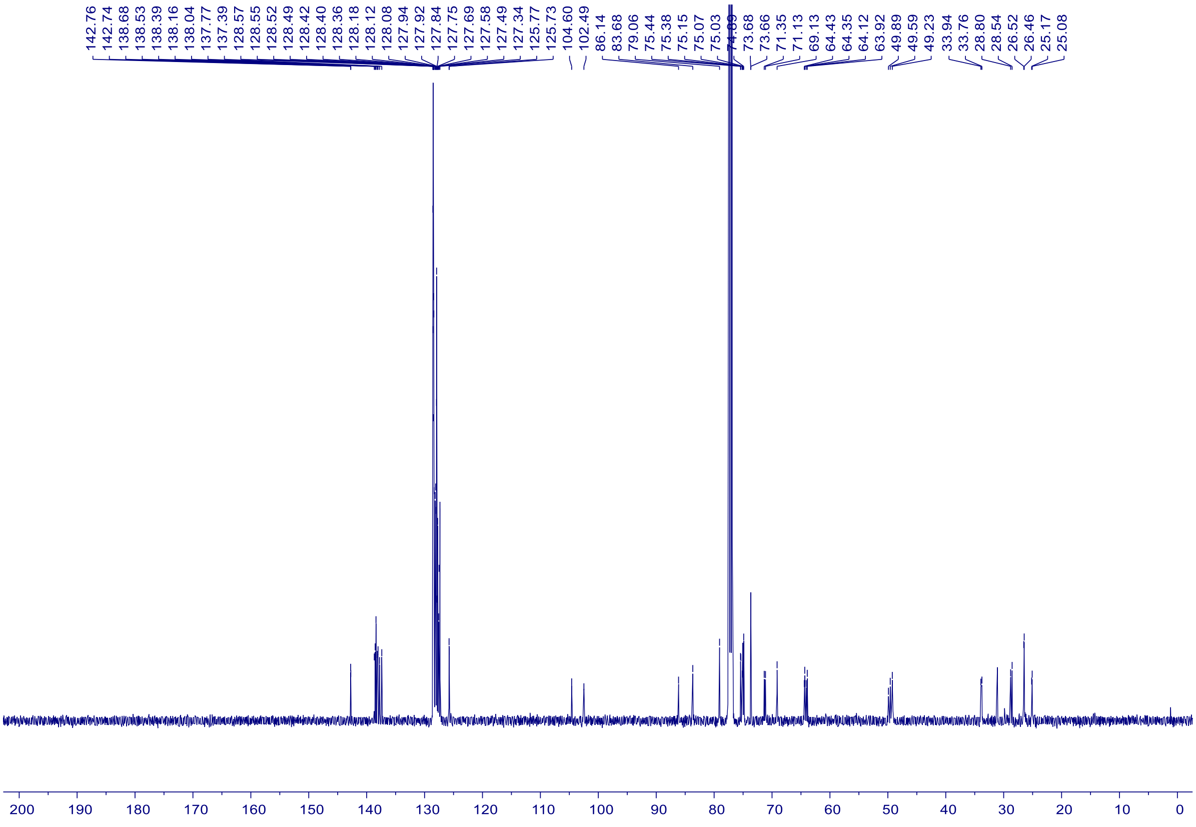

75 – ^{1}H NMR (500 MHz, $CDCl_3$)

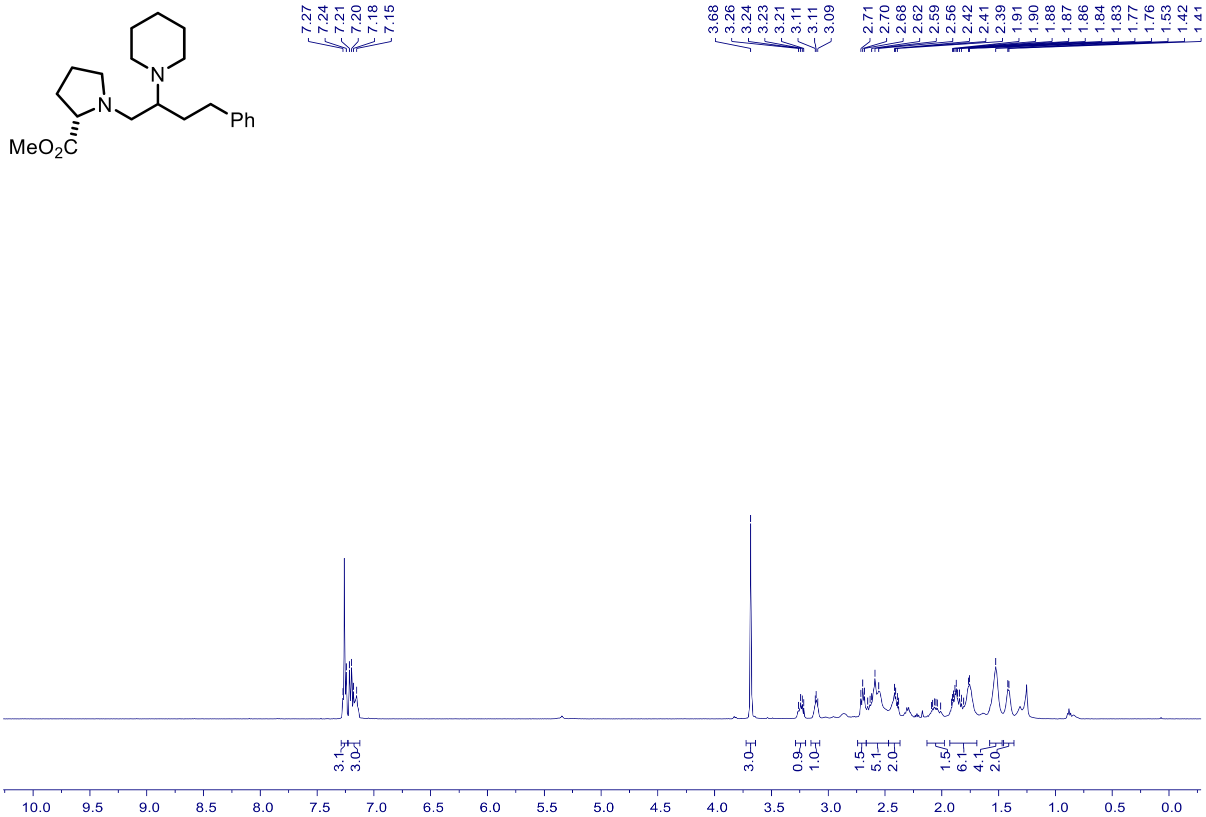

75 – ^{13}C NMR (126 MHz, $CDCl_3$)

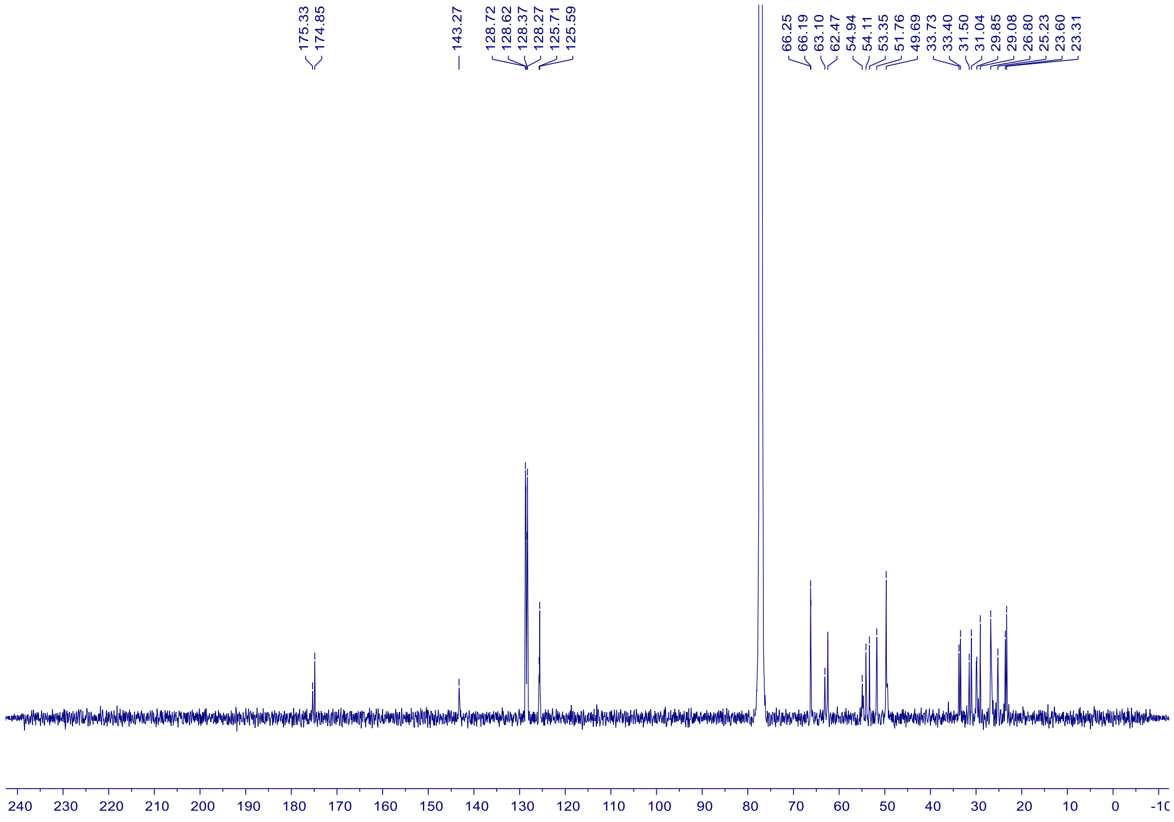

76 – ^{1}H NMR (500 MHz, $CDCl_3$)

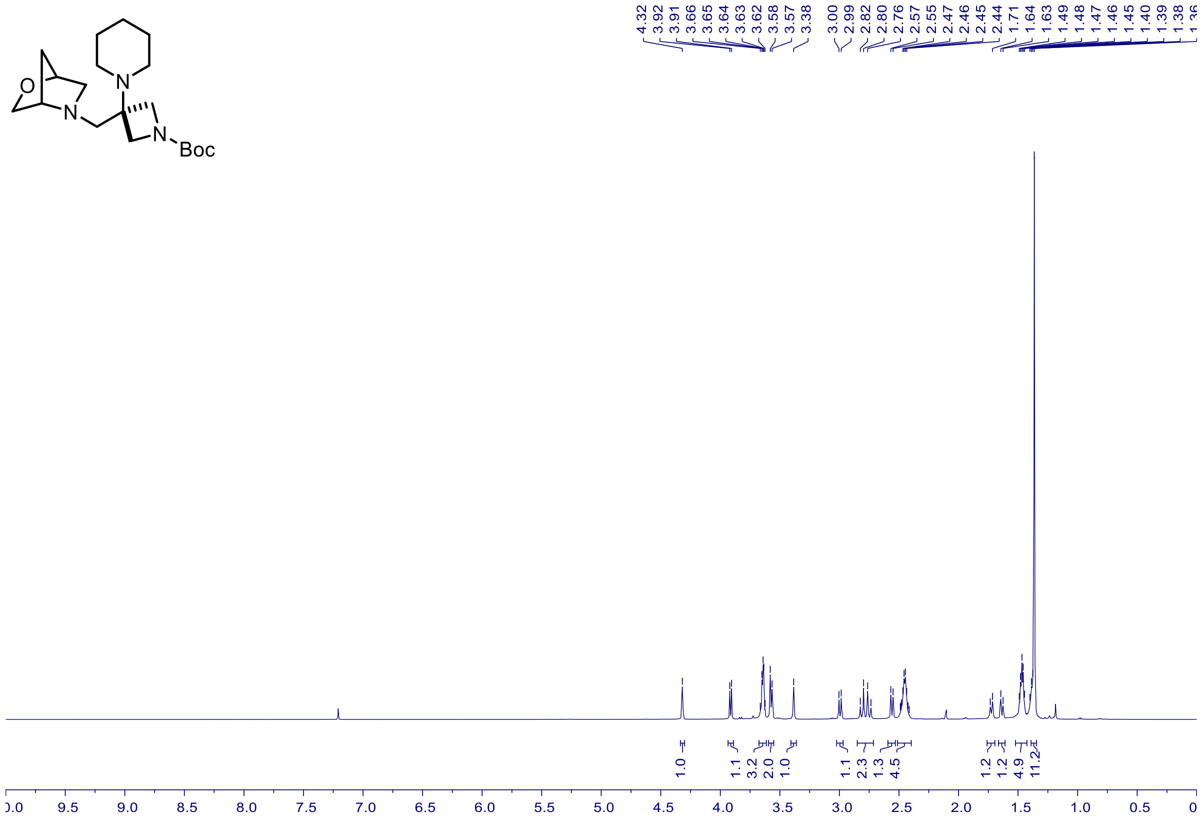

76 – ^{1}H NMR (500 MHz, $CDCl_3$)

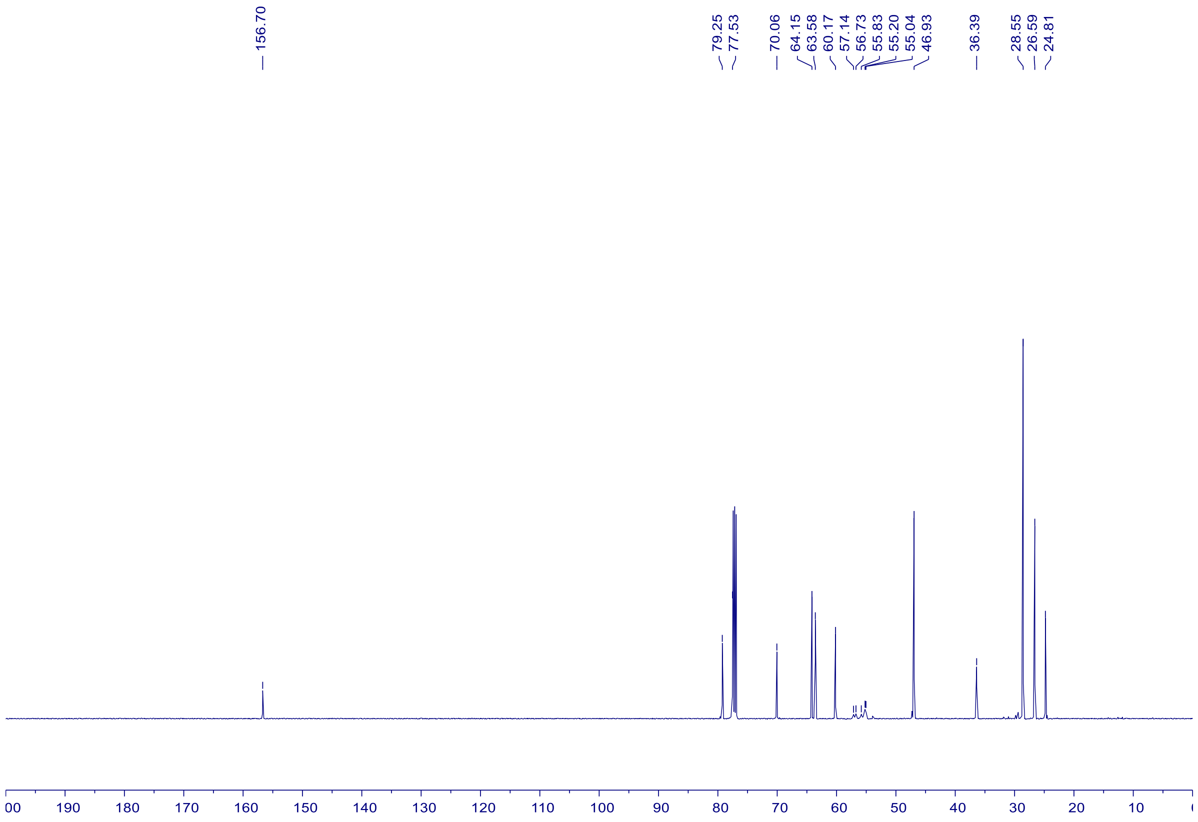

77 – ^{1}H NMR (500 MHz, CDCl$_3$)

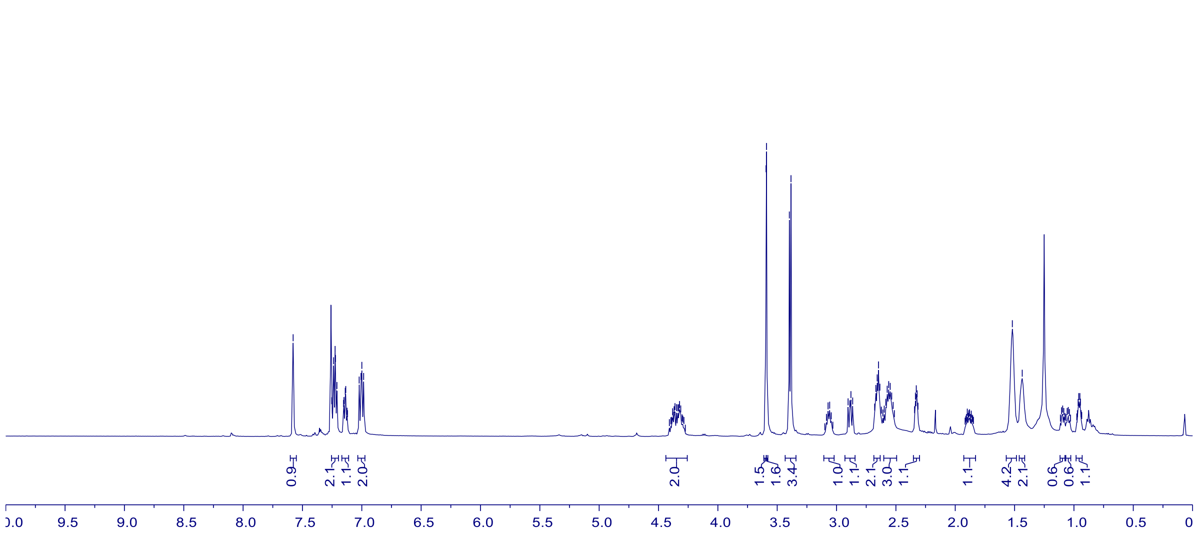

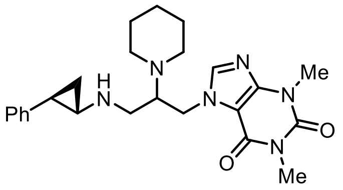

77 – ^{1}H NMR (500 MHz, CDCl$_3$)

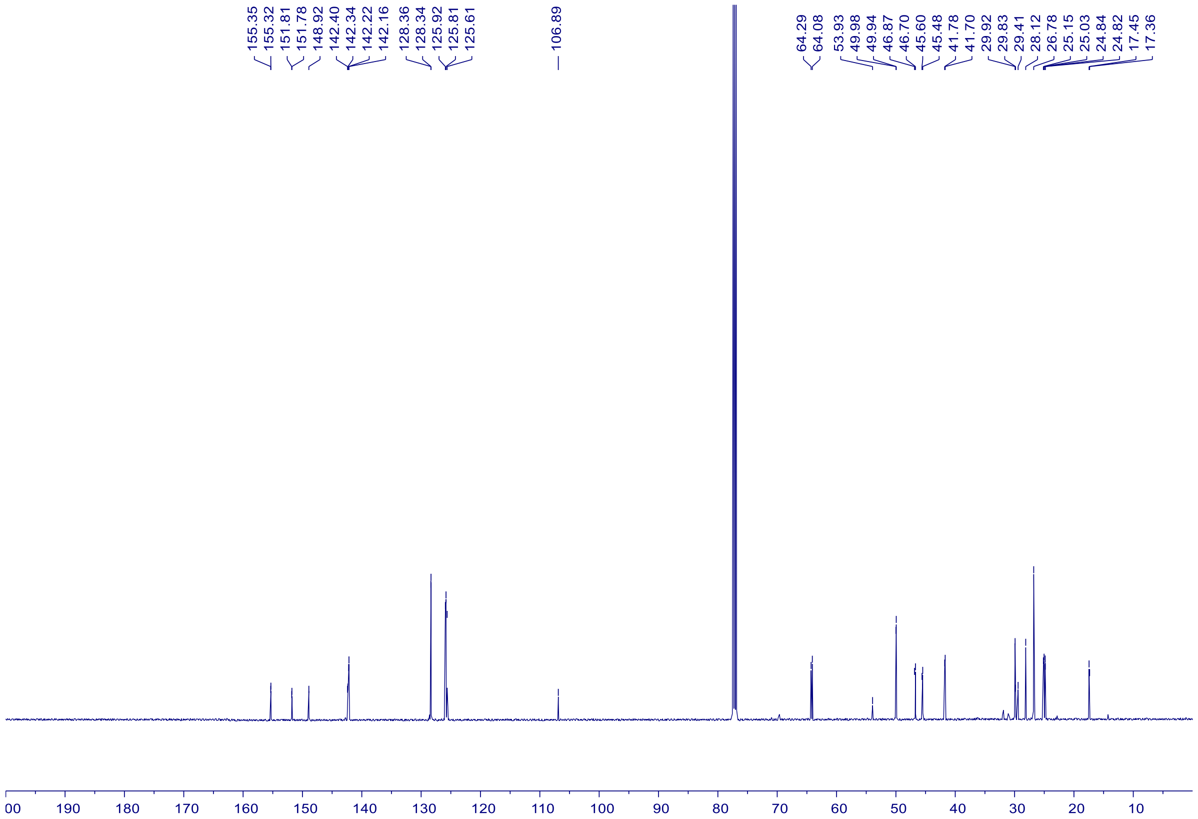

78 – ^{1}H NMR (500 MHz, CDCl$_3$)

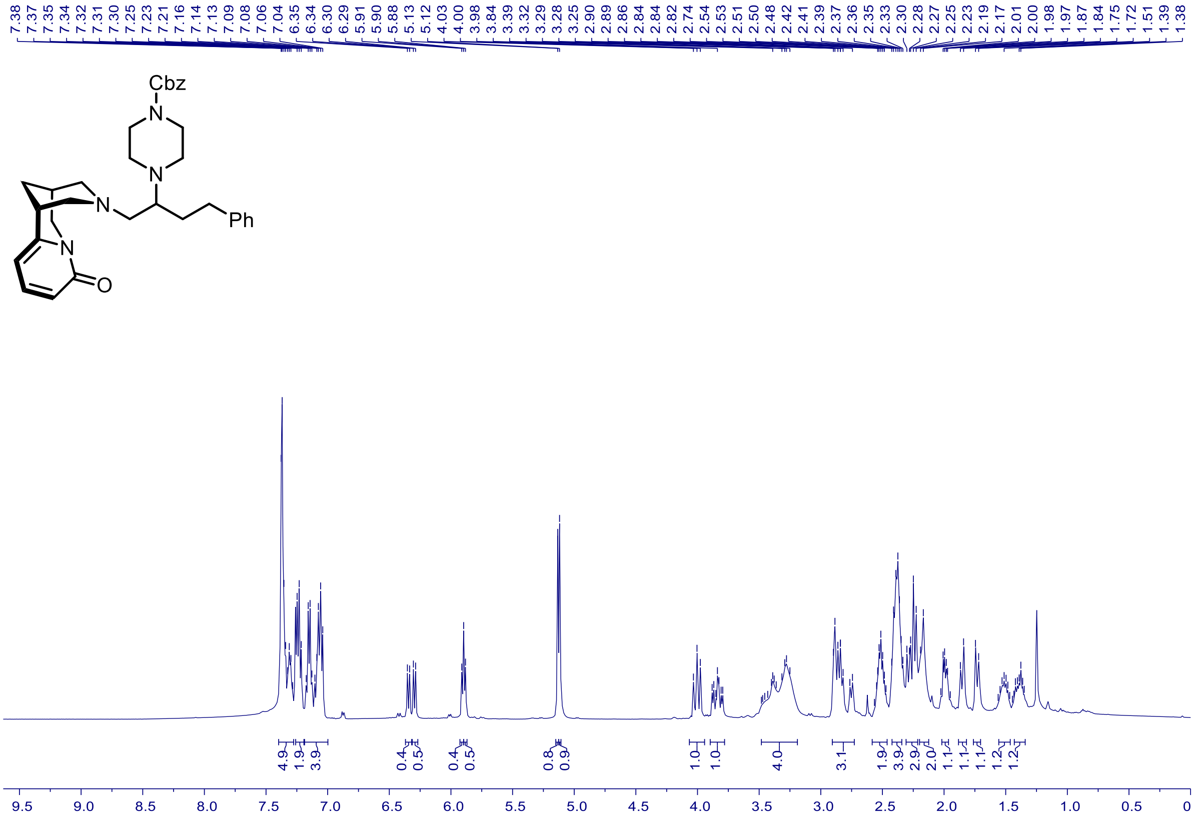

78 – ^{1}H NMR (500 MHz, CDCl$_3$)

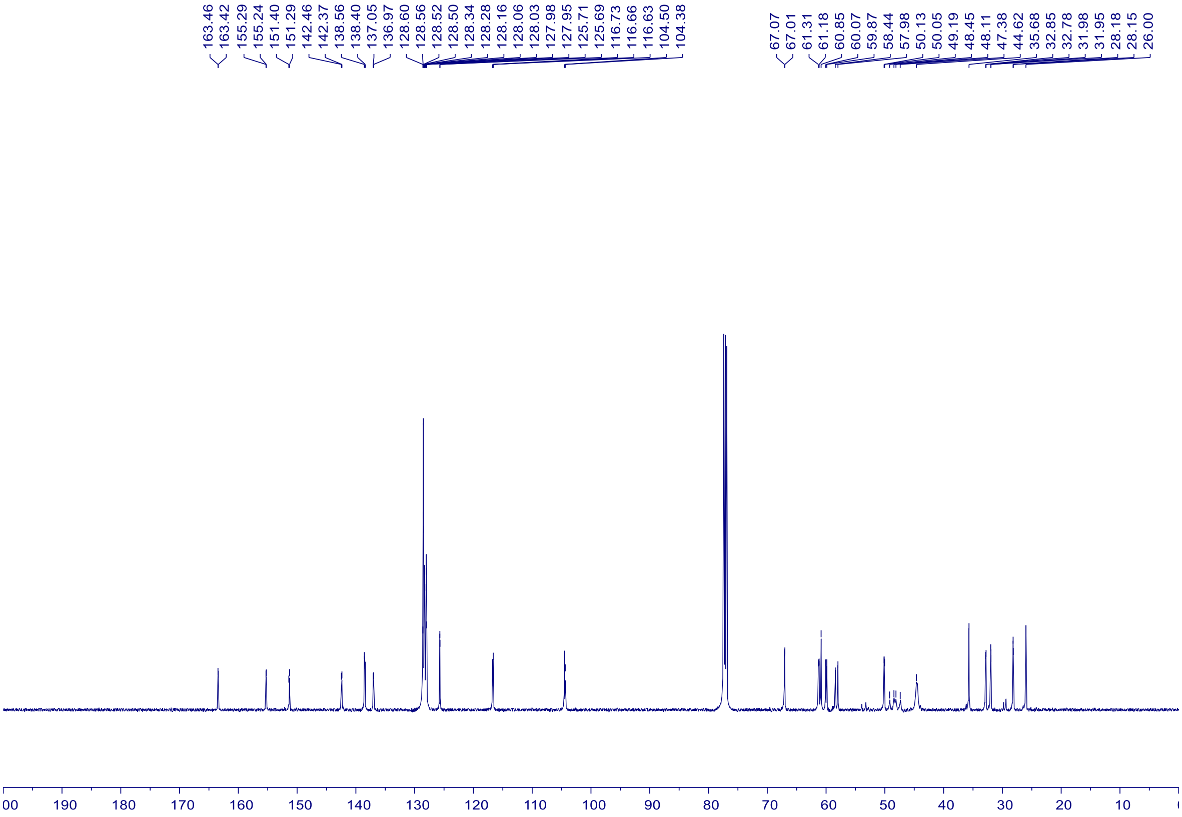

81 – ^{1}H NMR (500 MHz, $CDCl_3$)

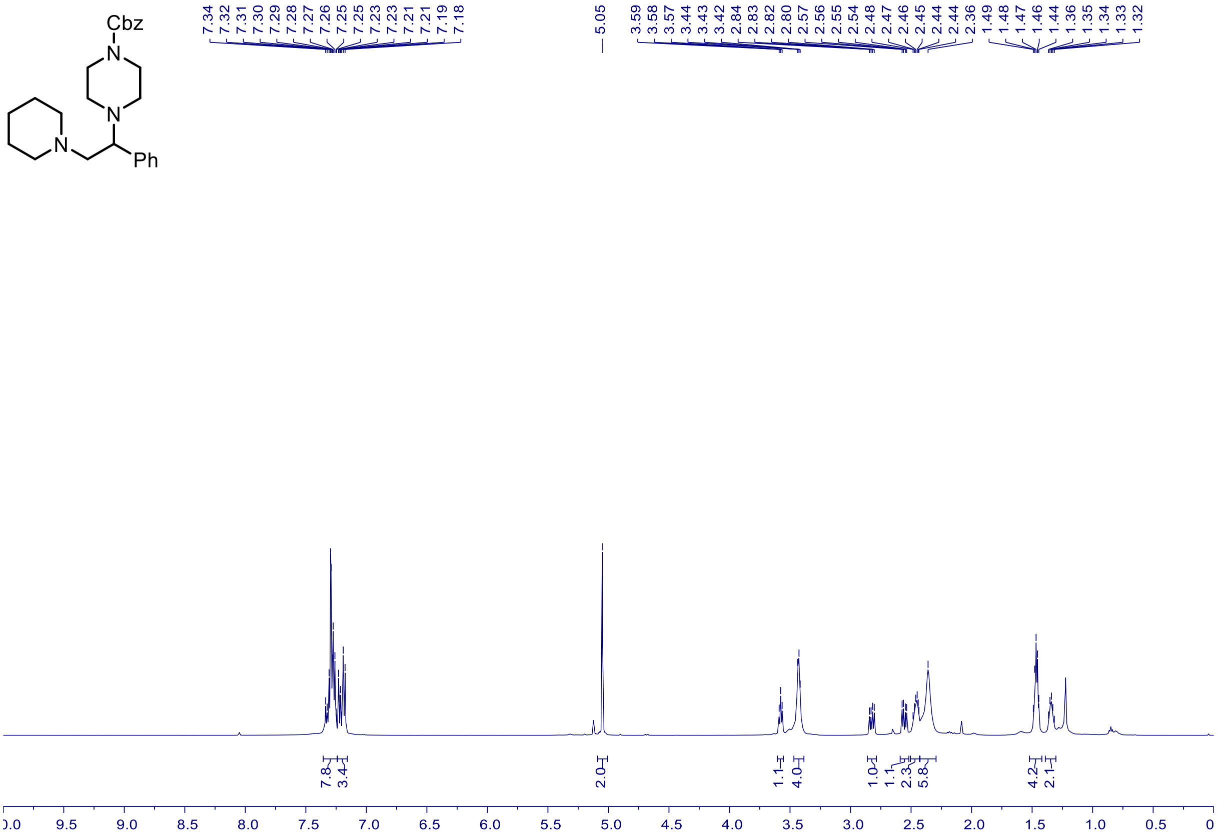

81 – ^{13}C NMR (126 MHz, $CDCl_3$)

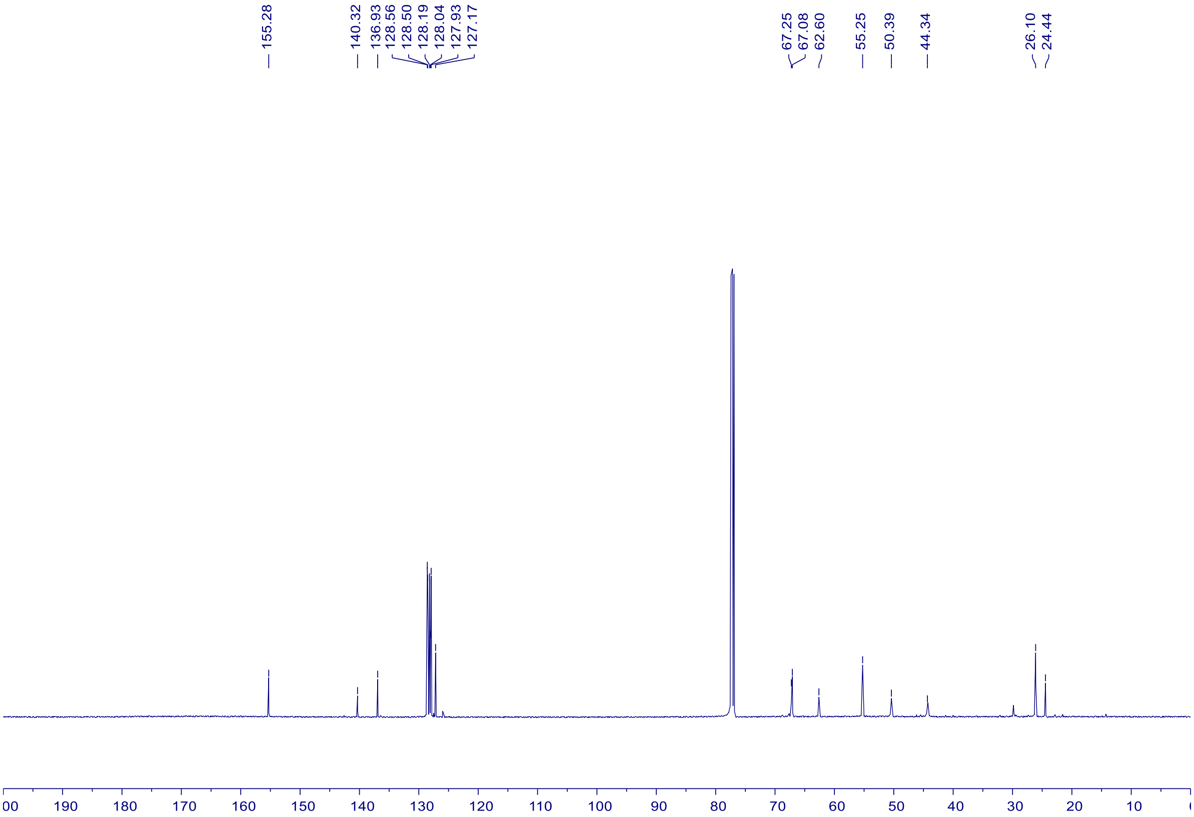

82 – ^{1}H NMR (500 MHz, $CDCl_3$)

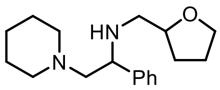

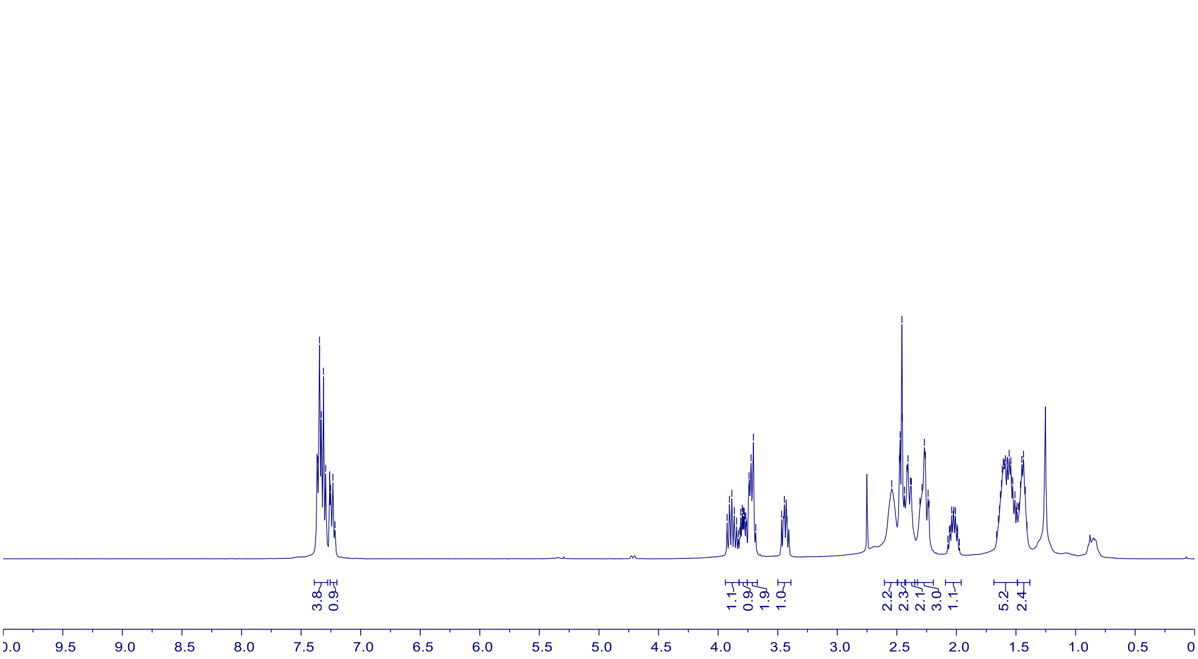

82 – ^{13}C NMR (126 MHz, $CDCl_3$)

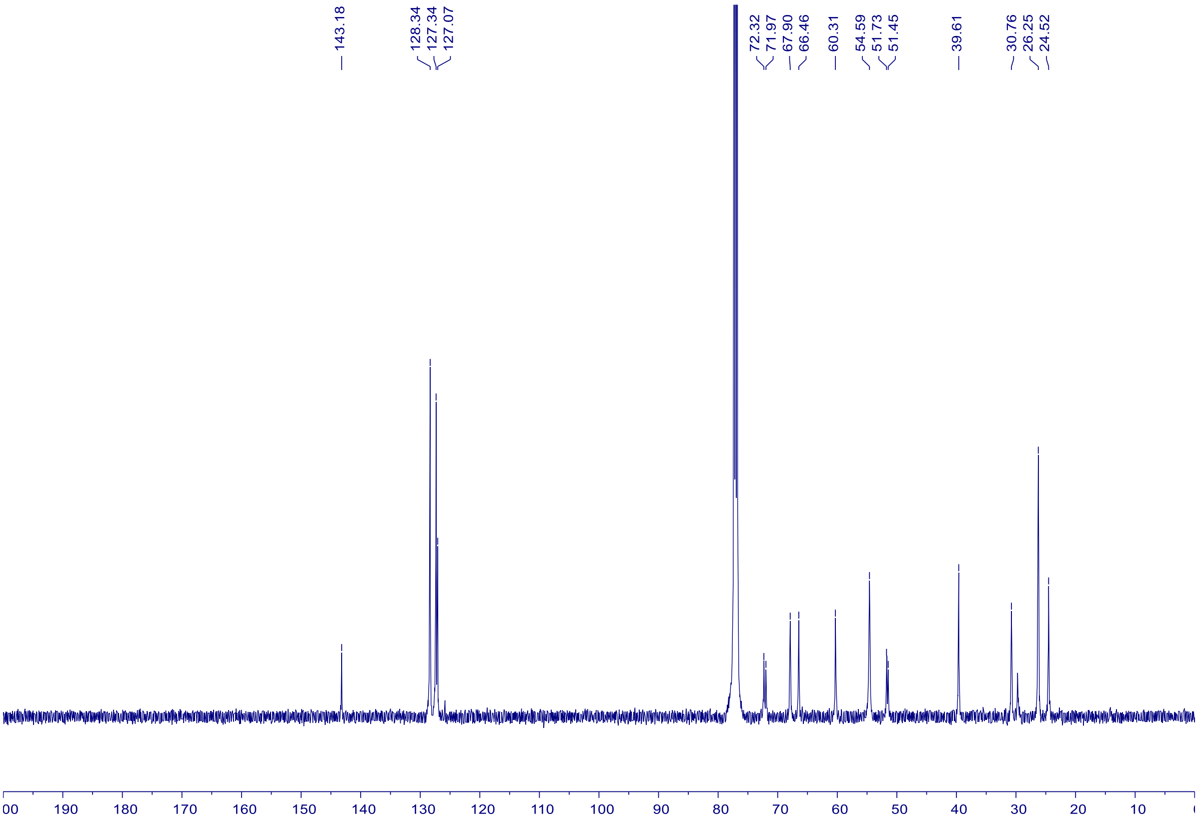

83 – ^{1}H NMR (500 MHz, $CDCl_3$)

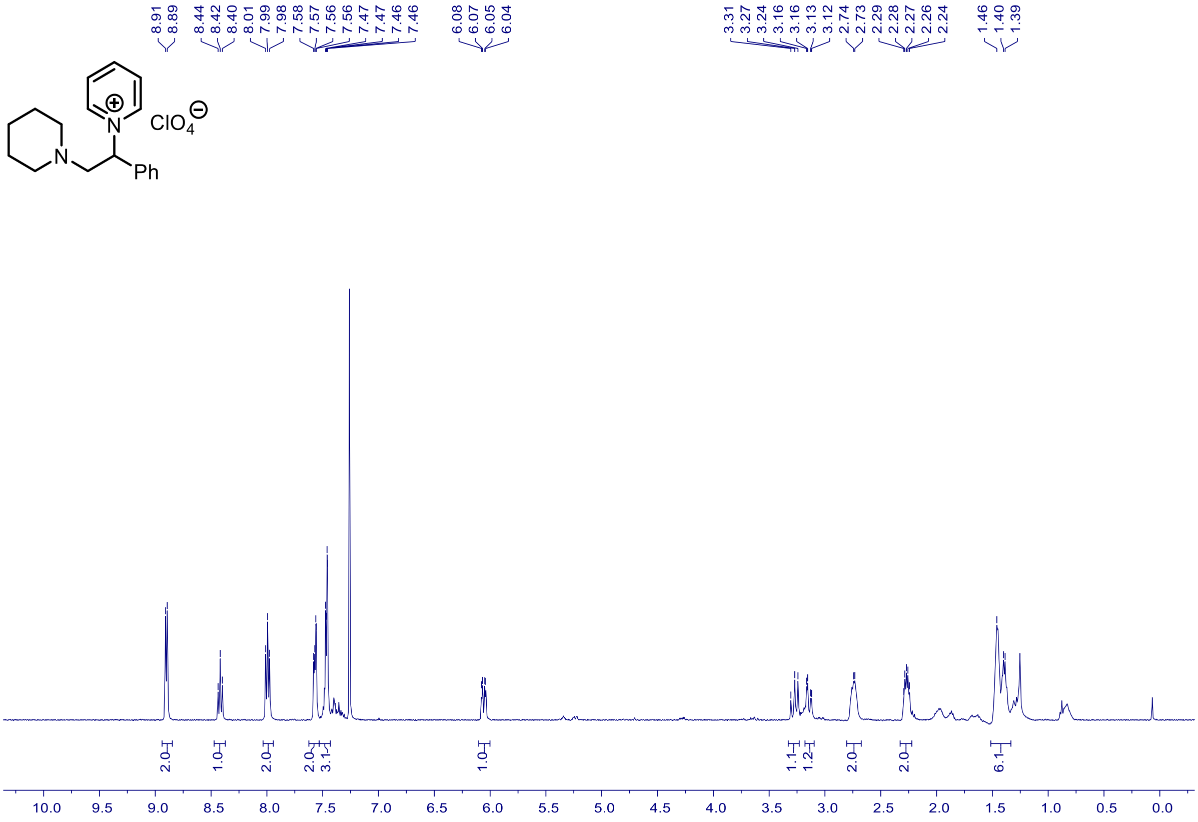

83 – ^{13}C NMR (126 MHz, $CDCl_3$)

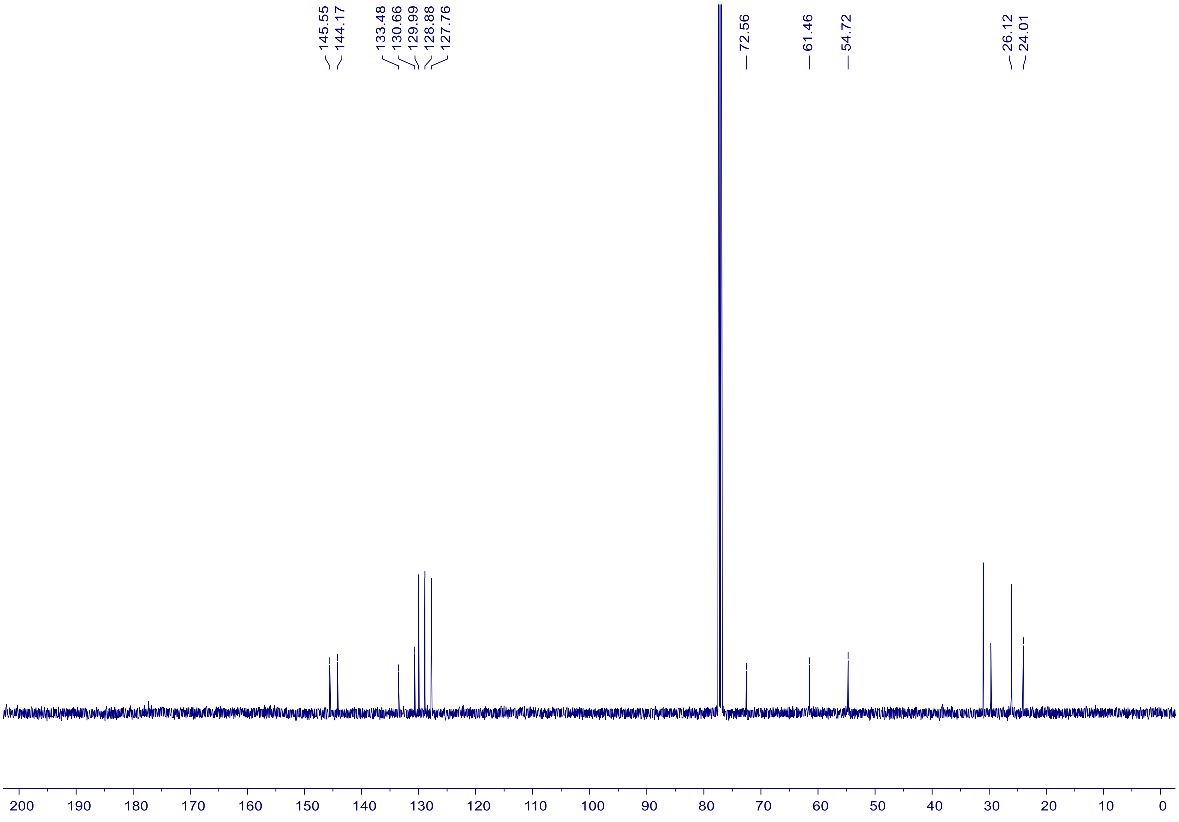

84 – ^{1}H NMR (500 MHz, $CDCl_3$)

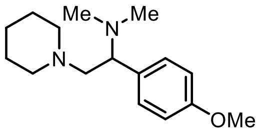

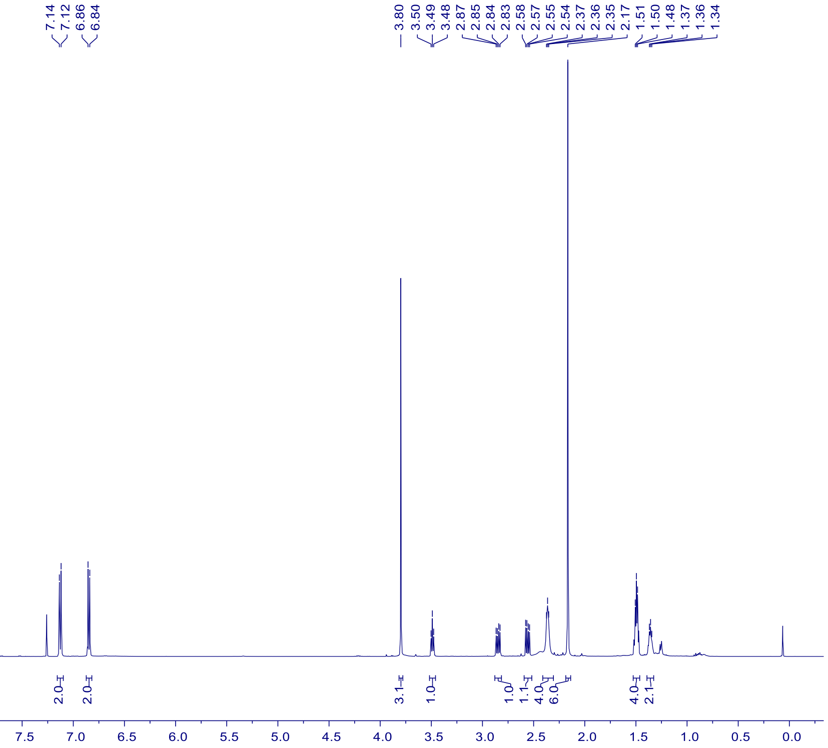

84 – ^{13}C NMR (126 MHz, $CDCl_3$)

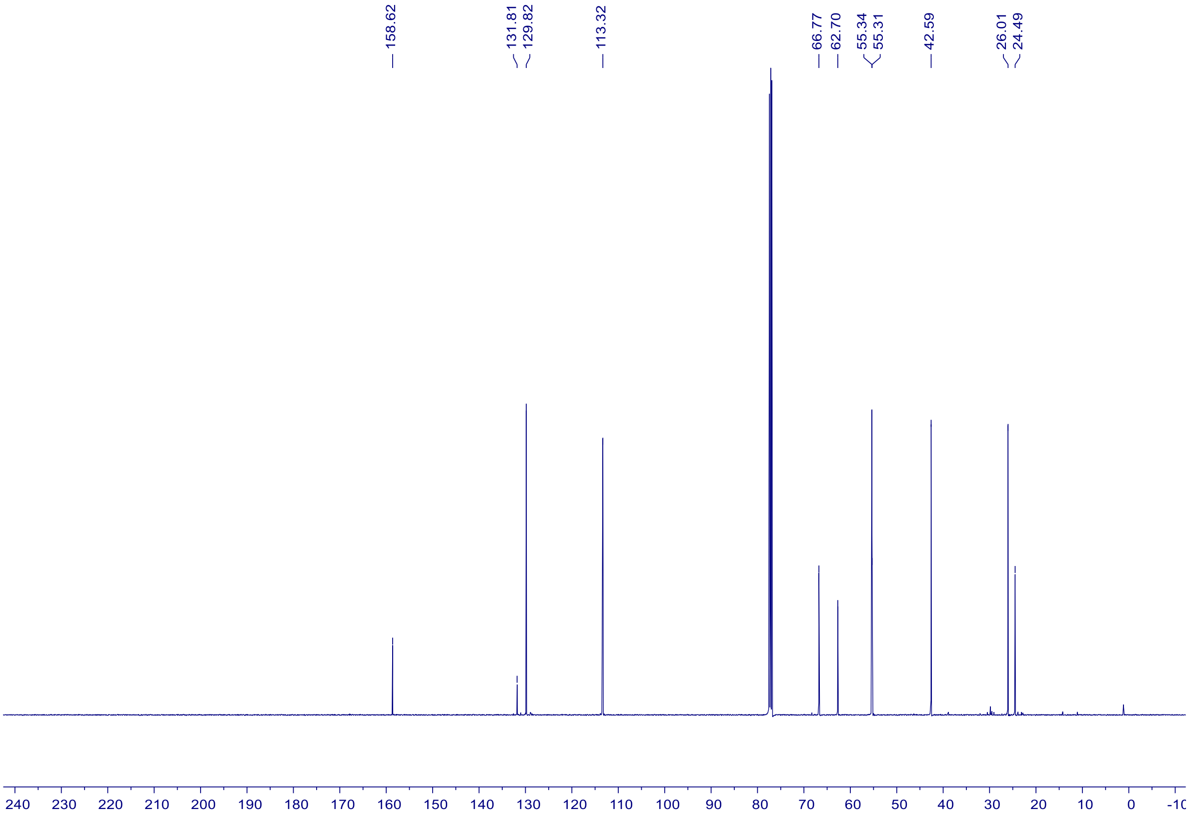

85 – ^{1}H NMR (500 MHz, CDCl$_3$)

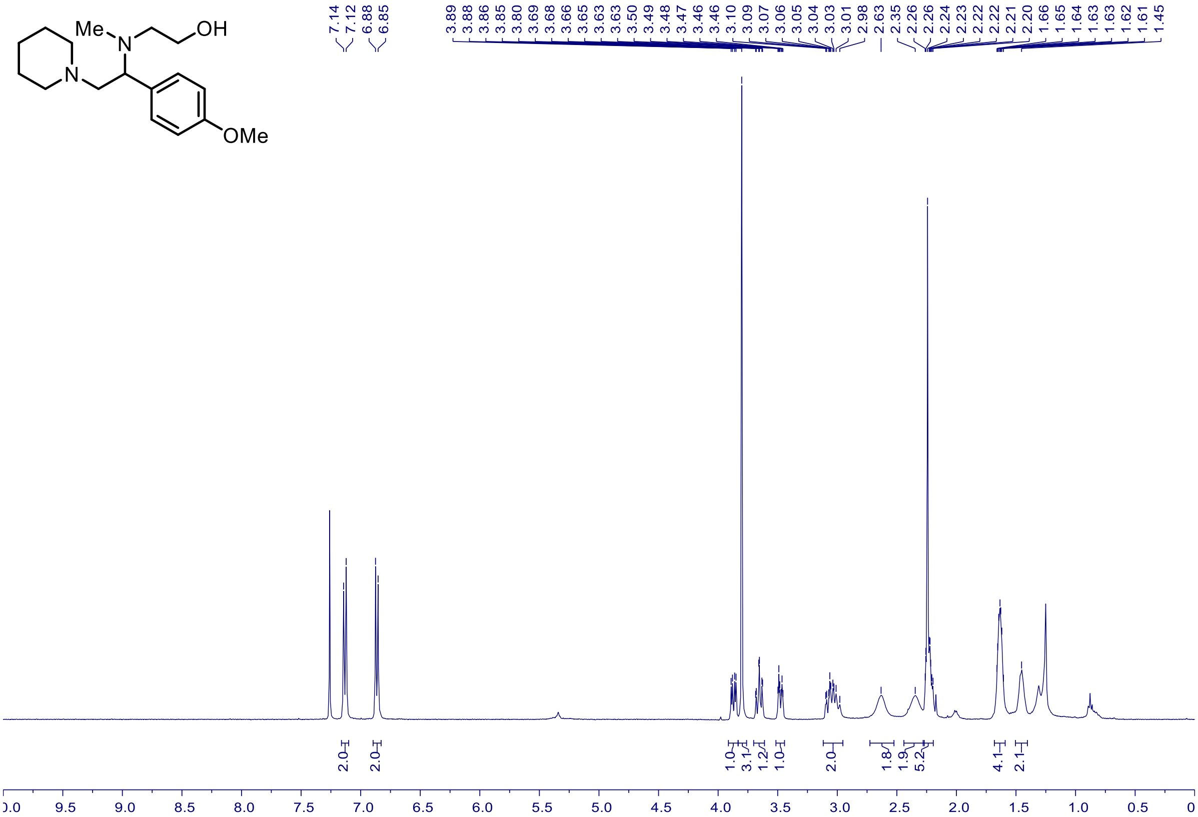

85 – ^{13}C NMR (126 MHz, CDCl$_3$)

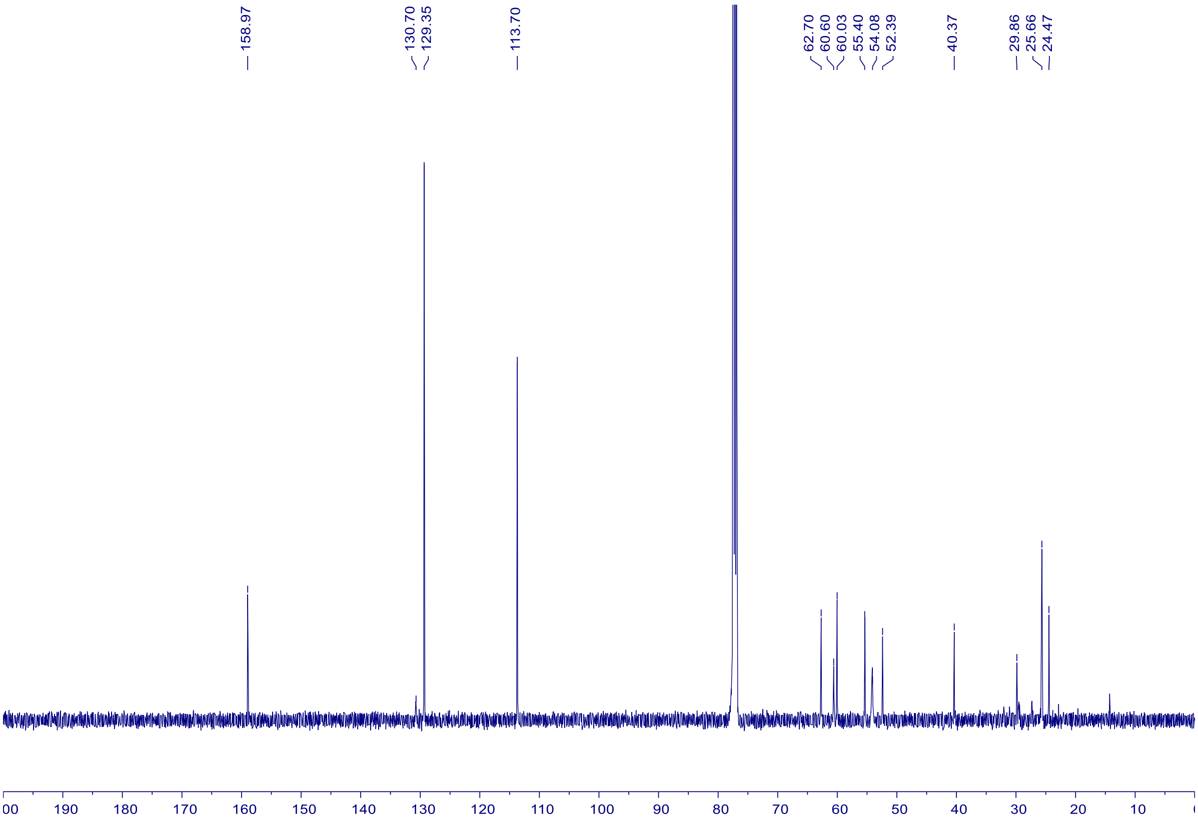

86 – ^{1}H NMR (500 MHz, $CDCl_3$)

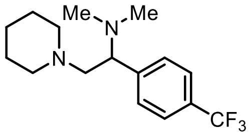

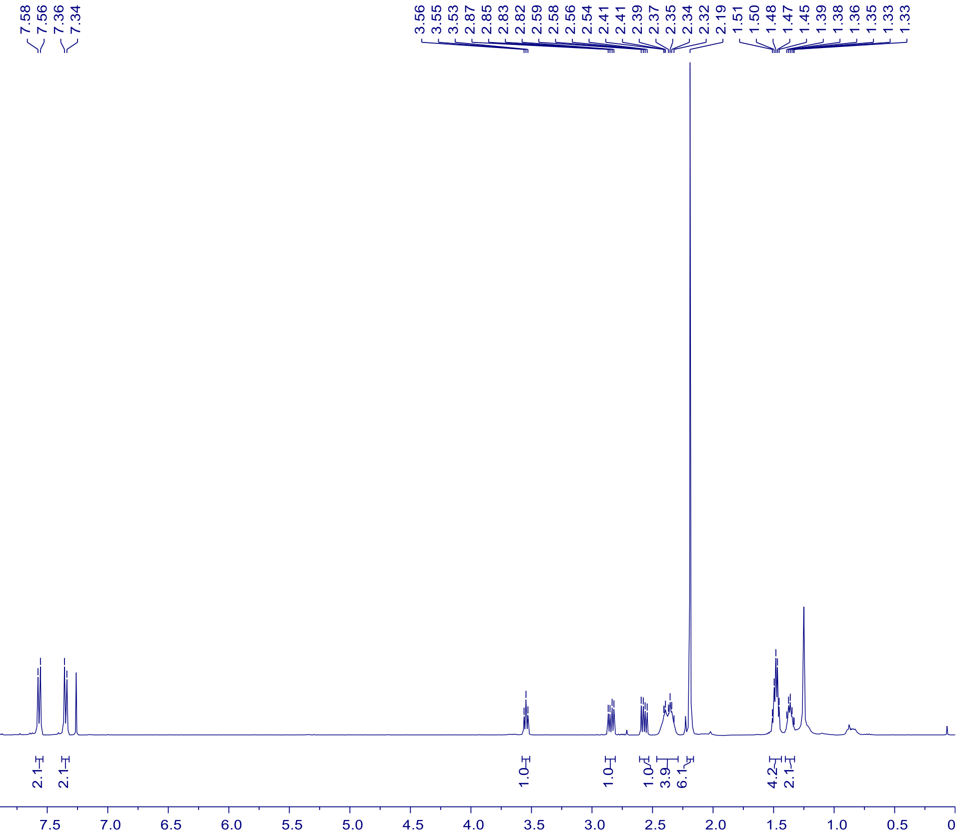

86 – ^{13}C NMR (126 MHz, $CDCl_3$)

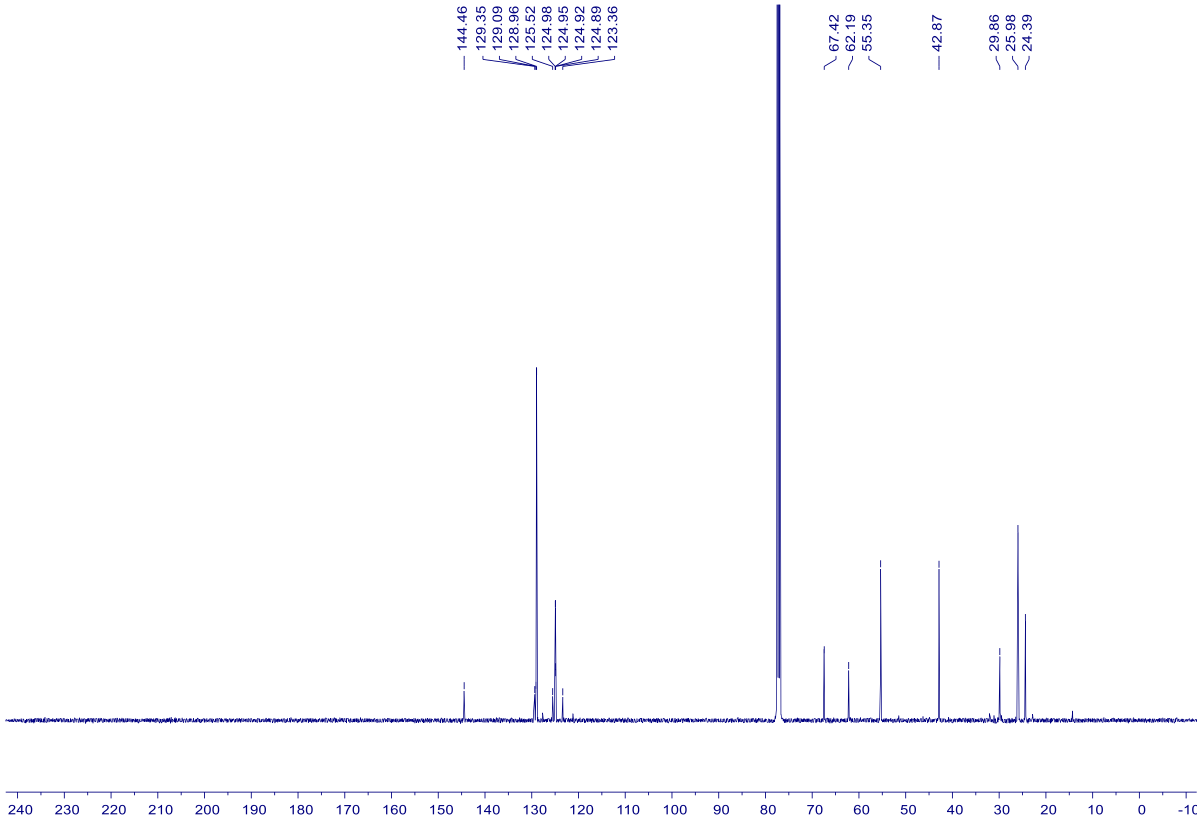

86 – ^{19}F NMR (471 MHz, $CDCl_3$)

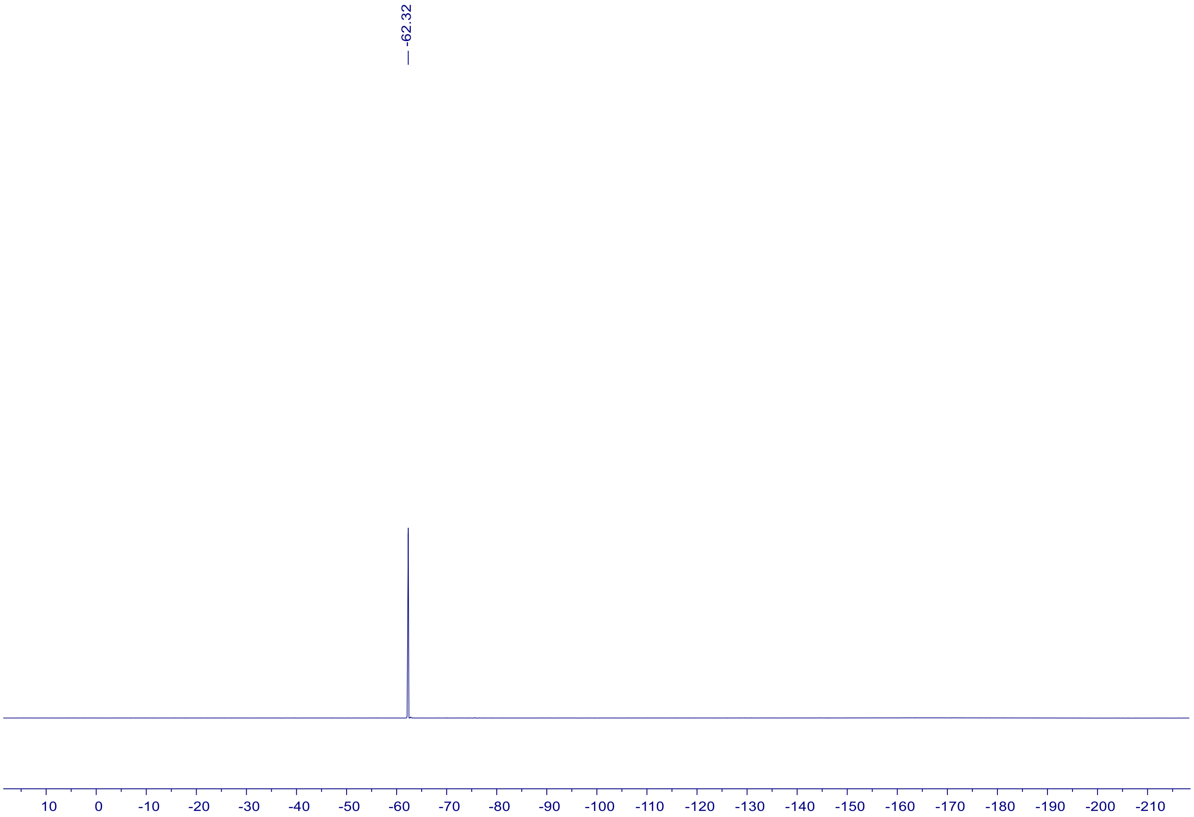

87 – ^{1}H NMR (500 MHz, $CDCl_3$)

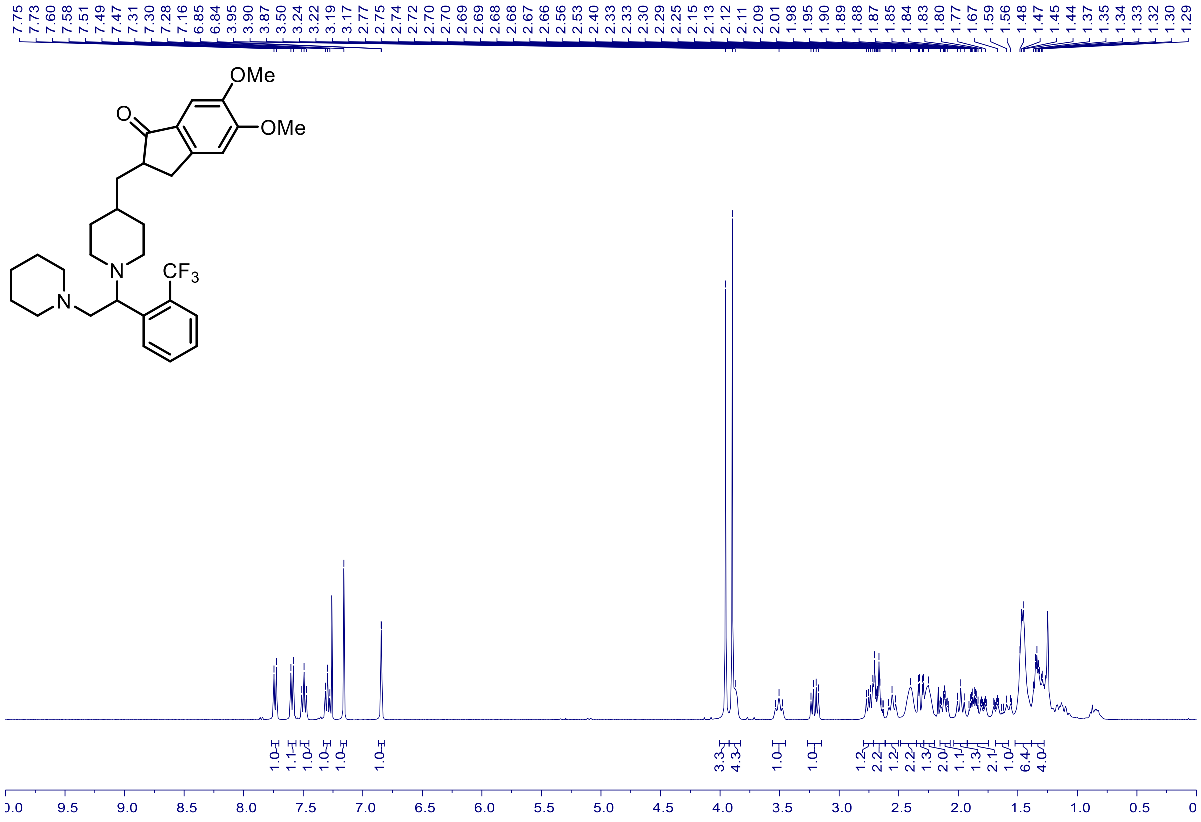

87 – ^{13}C NMR (126 MHz, $CDCl_3$)

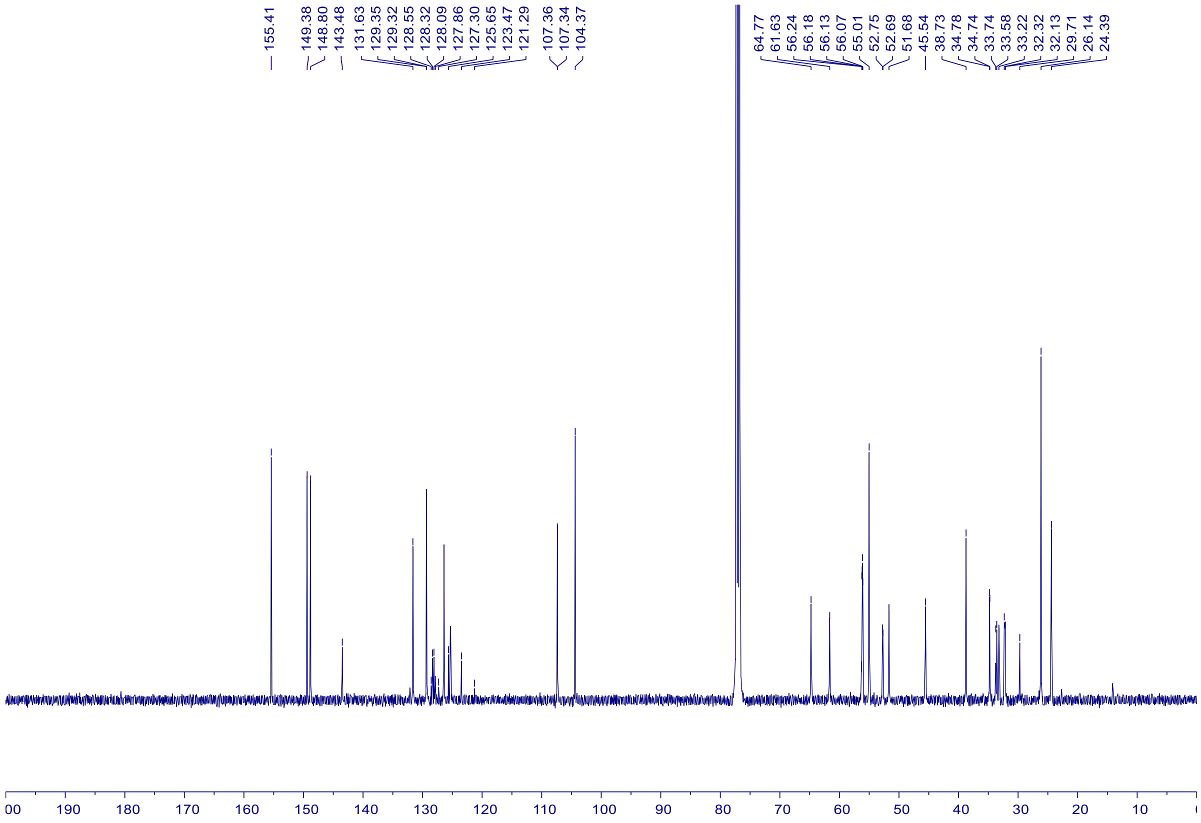

87 – ^{19}F NMR (471 MHz, $CDCl_3$)

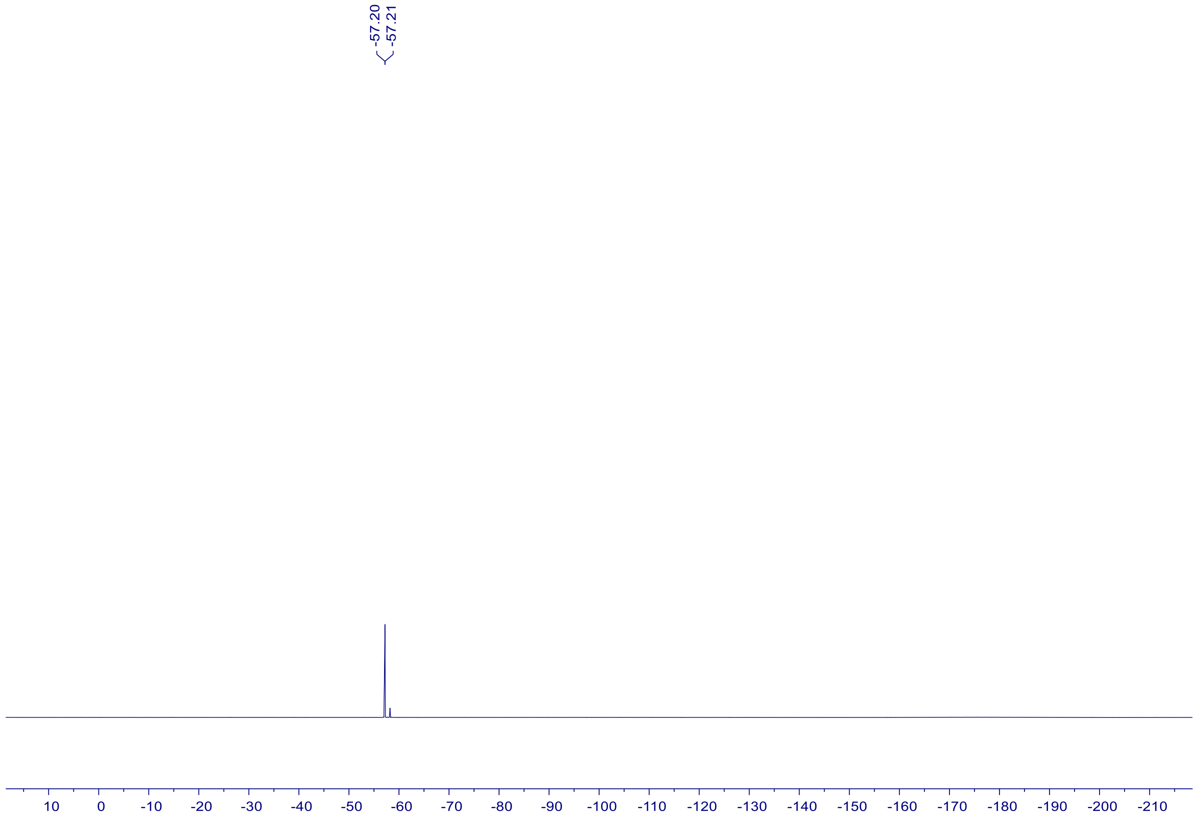

88 – ^{1}H NMR (500 MHz, CDCl$_3$)

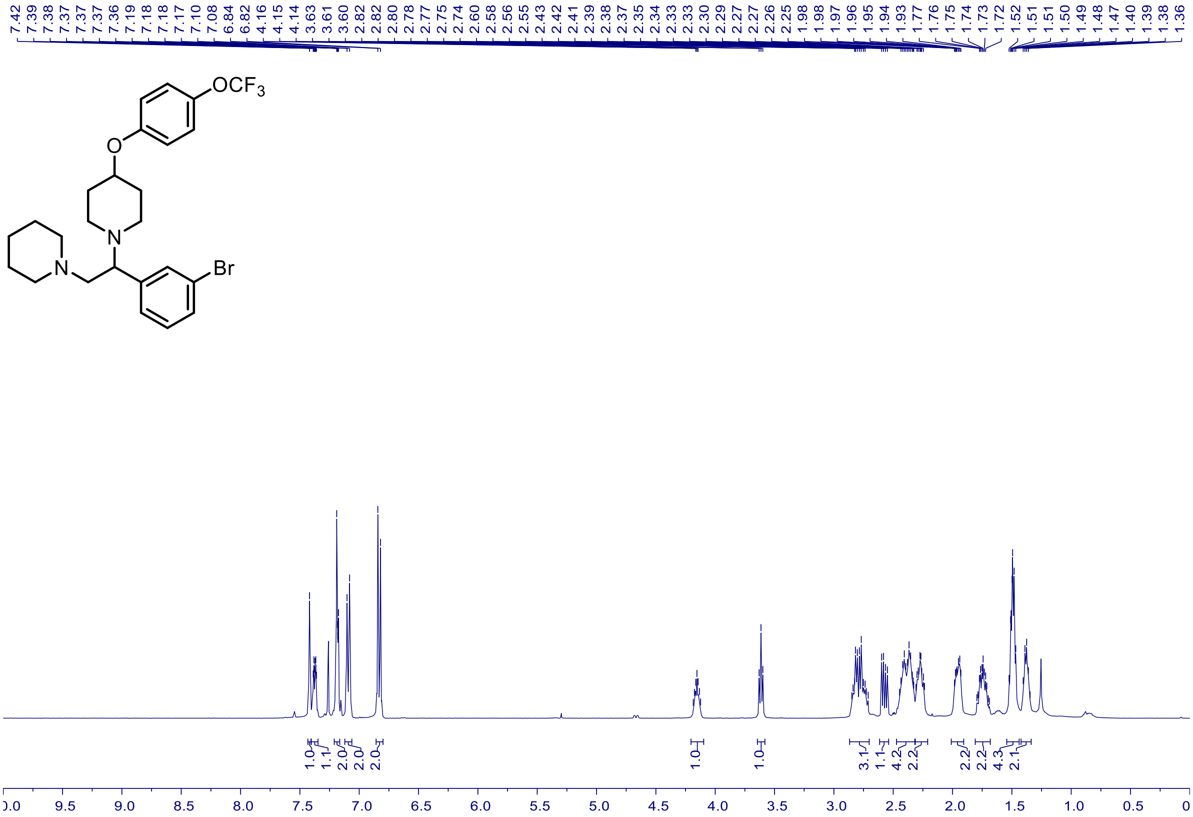

88 – ^{13}C NMR (126 MHz, CDCl$_3$)

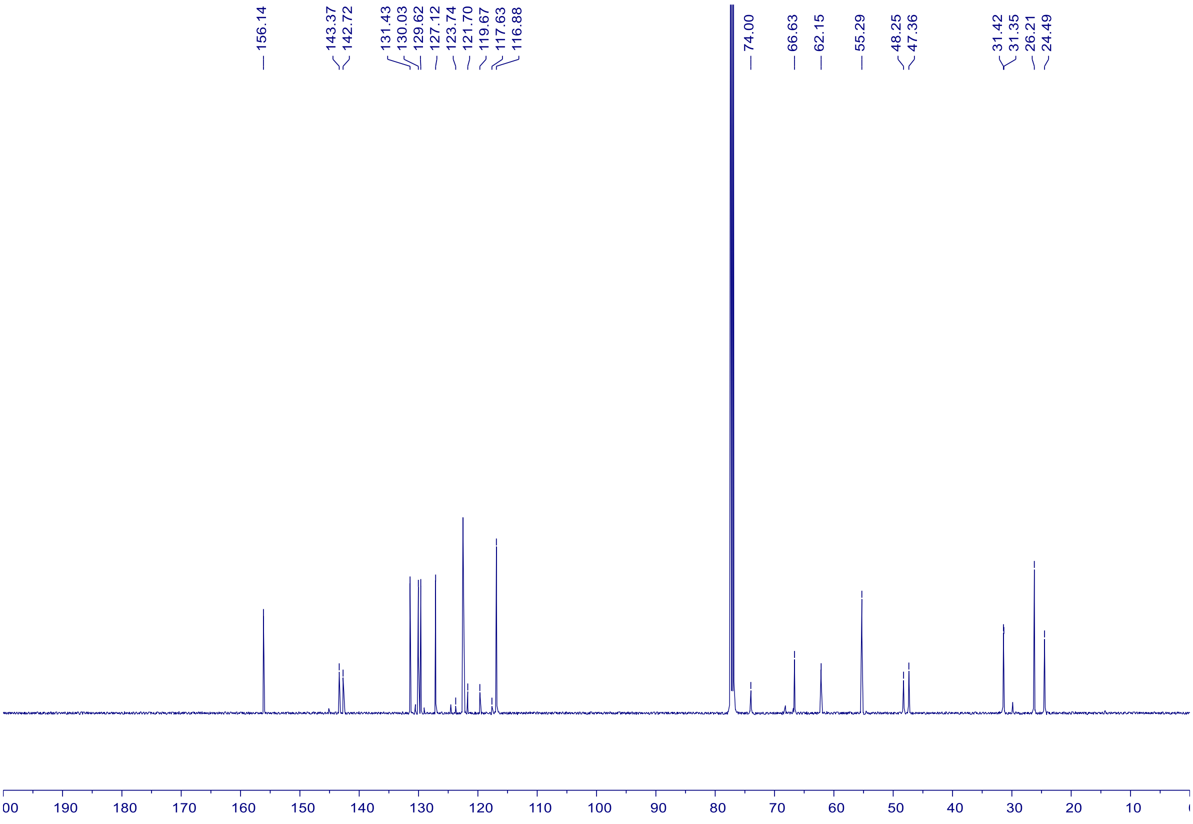

88 – ^{19}F NMR (471 MHz, $CDCl_3$)

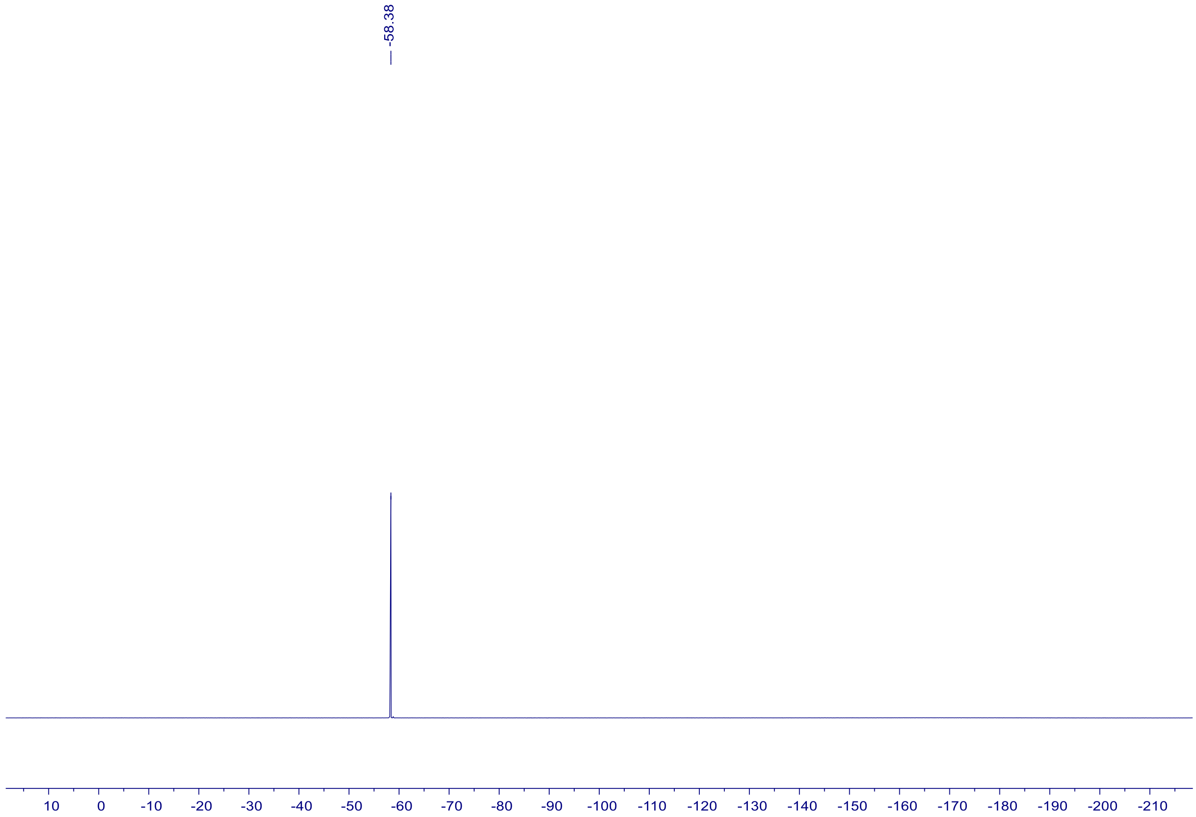

89 – ^{1}H NMR (500 MHz, $CDCl_3$–CD_3OD)

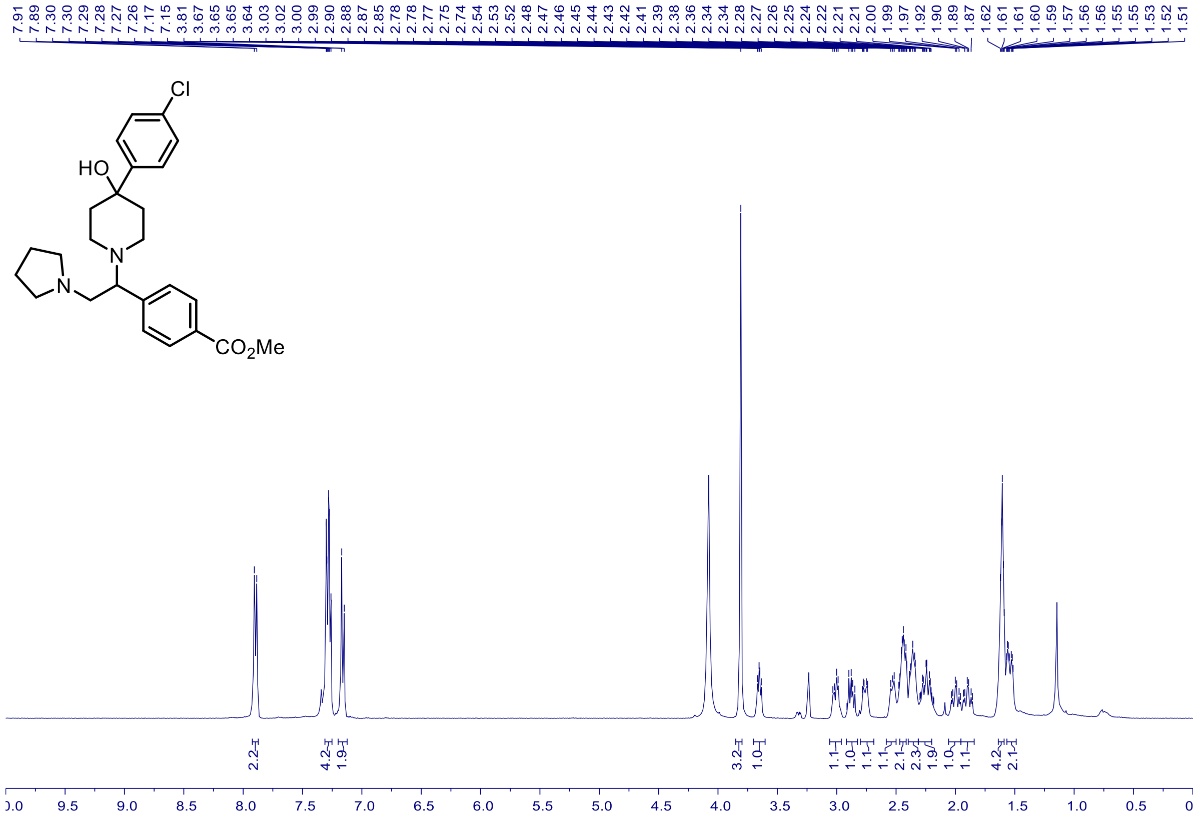

89 – ^{13}C NMR (126 MHz, $CDCl_3$–CD_3OD)

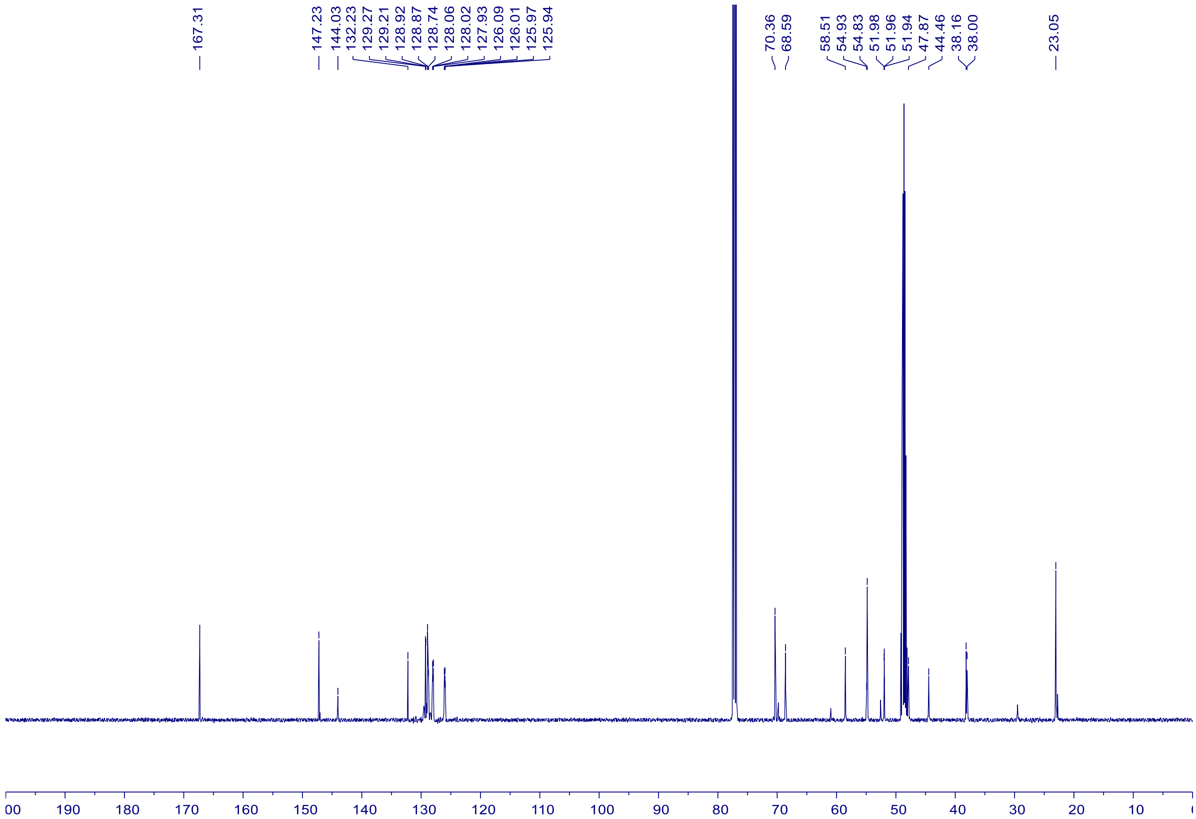

90 – ^{1}H NMR (500 MHz, CDCl$_3$)

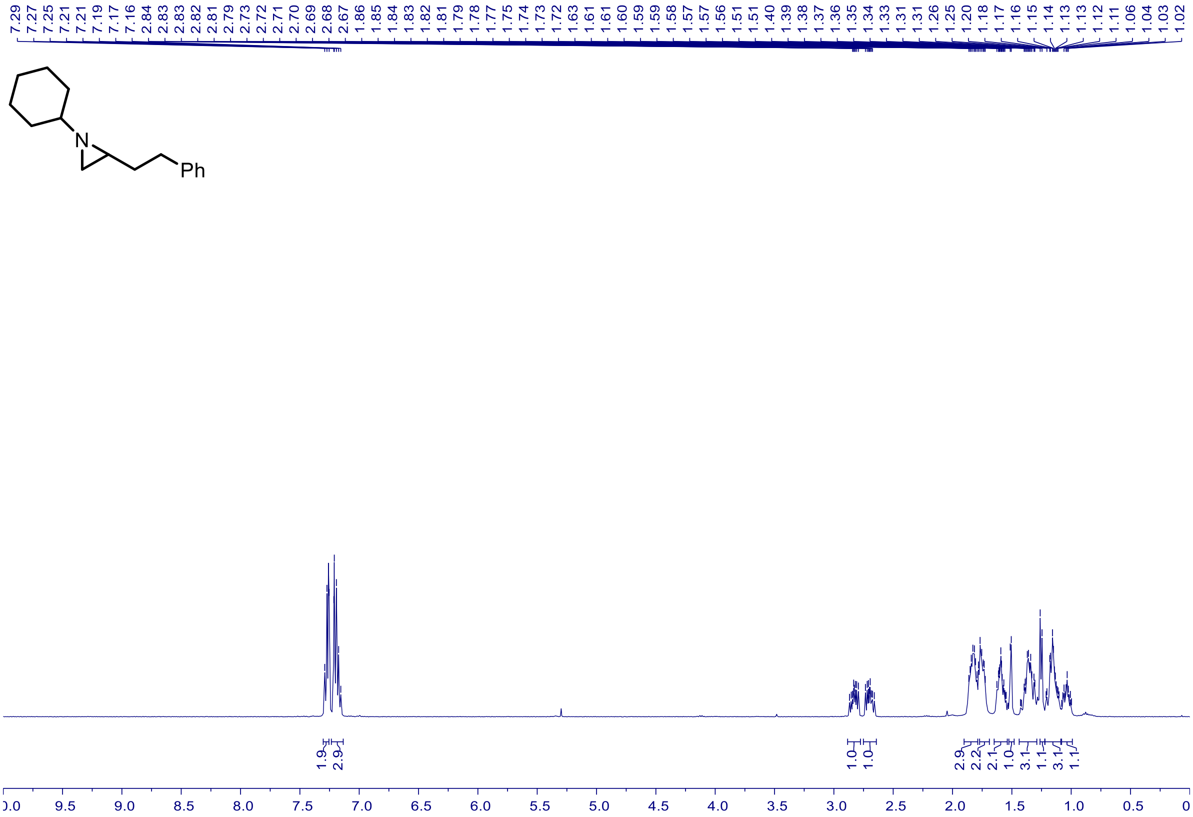

90 – ^{13}C NMR (126 MHz, CDCl$_3$)

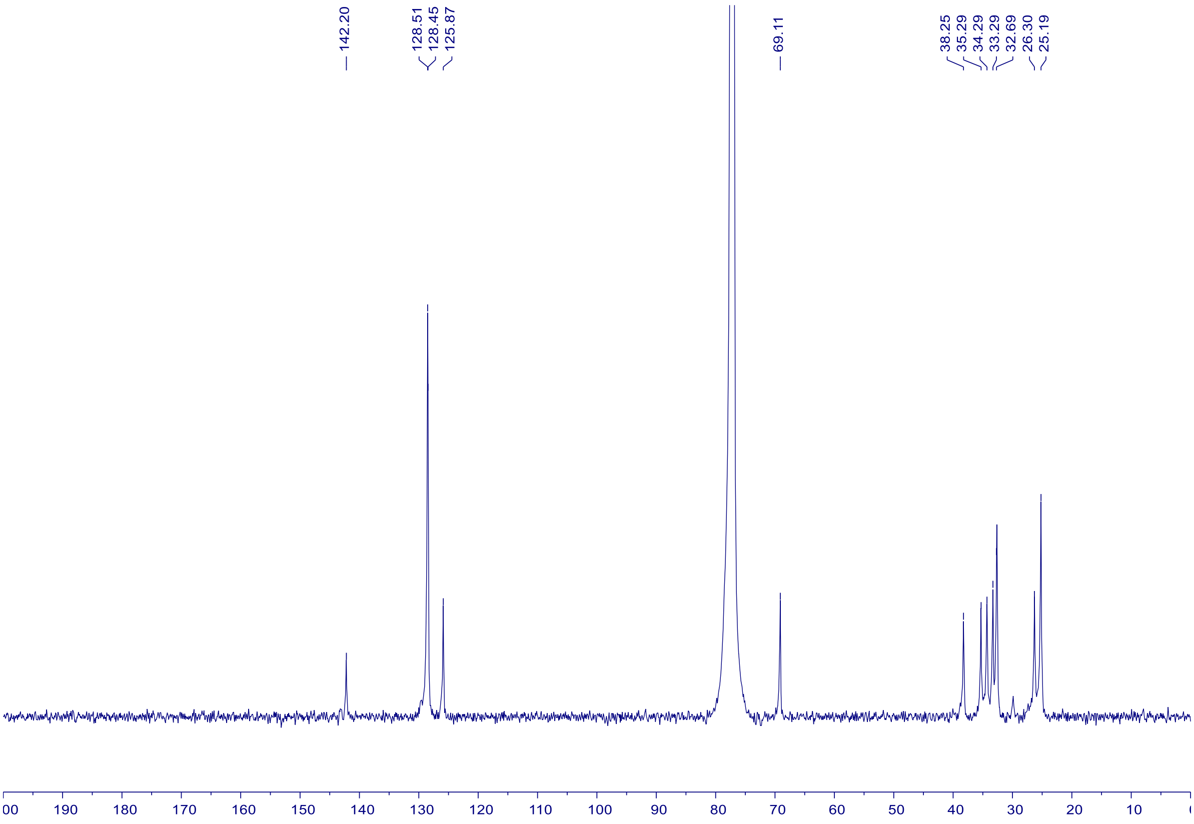

91 – ^{1}H NMR (500 MHz, $CDCl_3$)

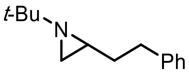

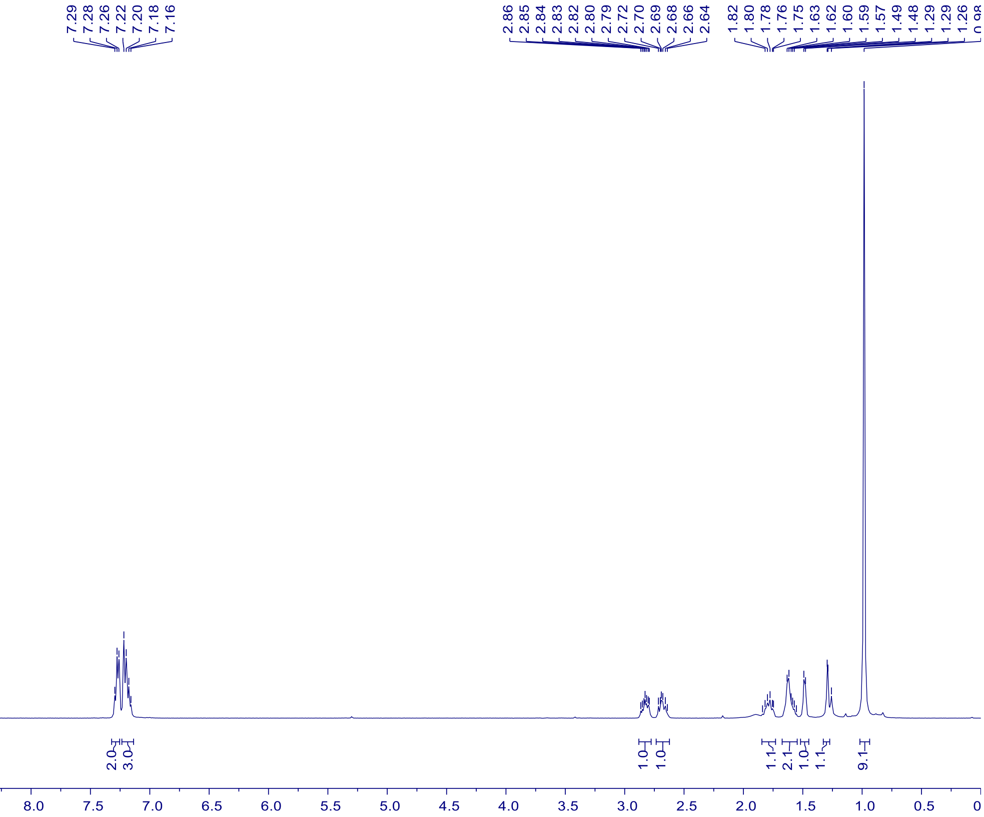

91 – ^{13}C NMR (126 MHz, $CDCl_3$)

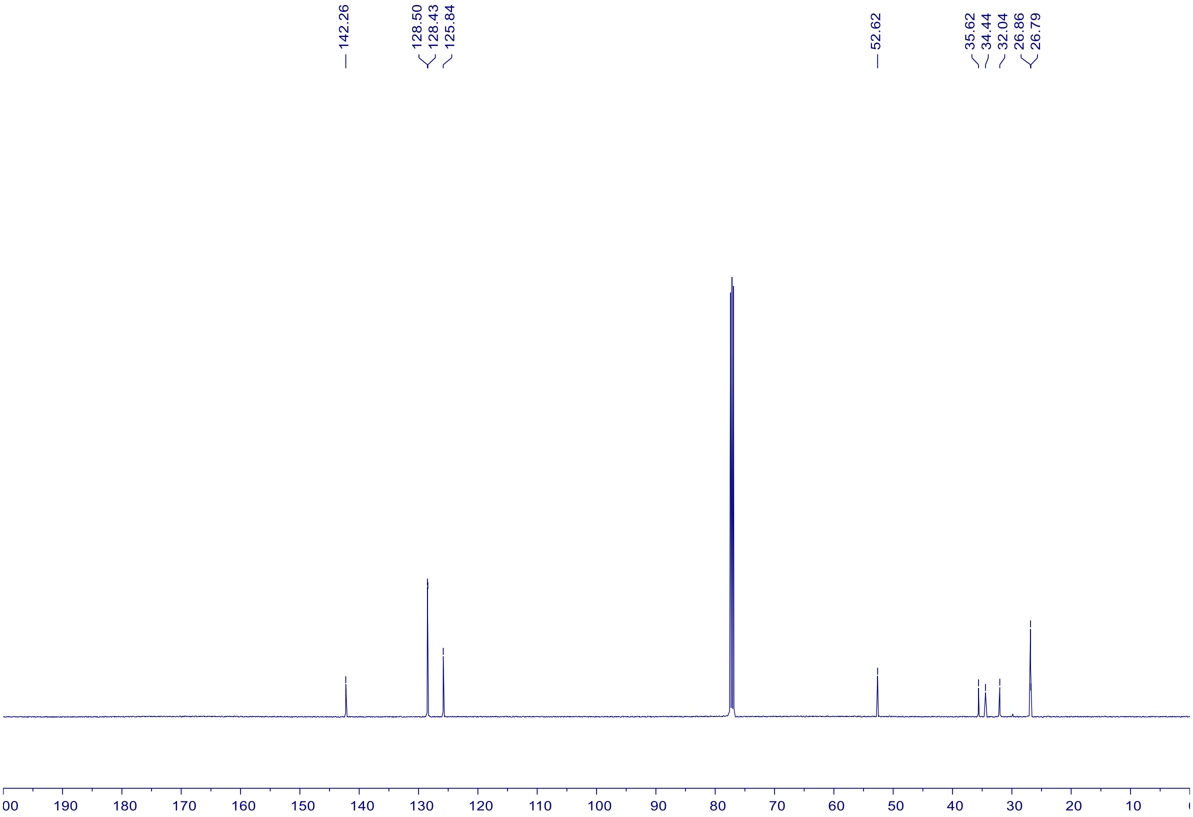

92 – ^{1}H NMR (500 MHz, $CDCl_3$)

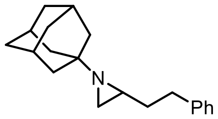

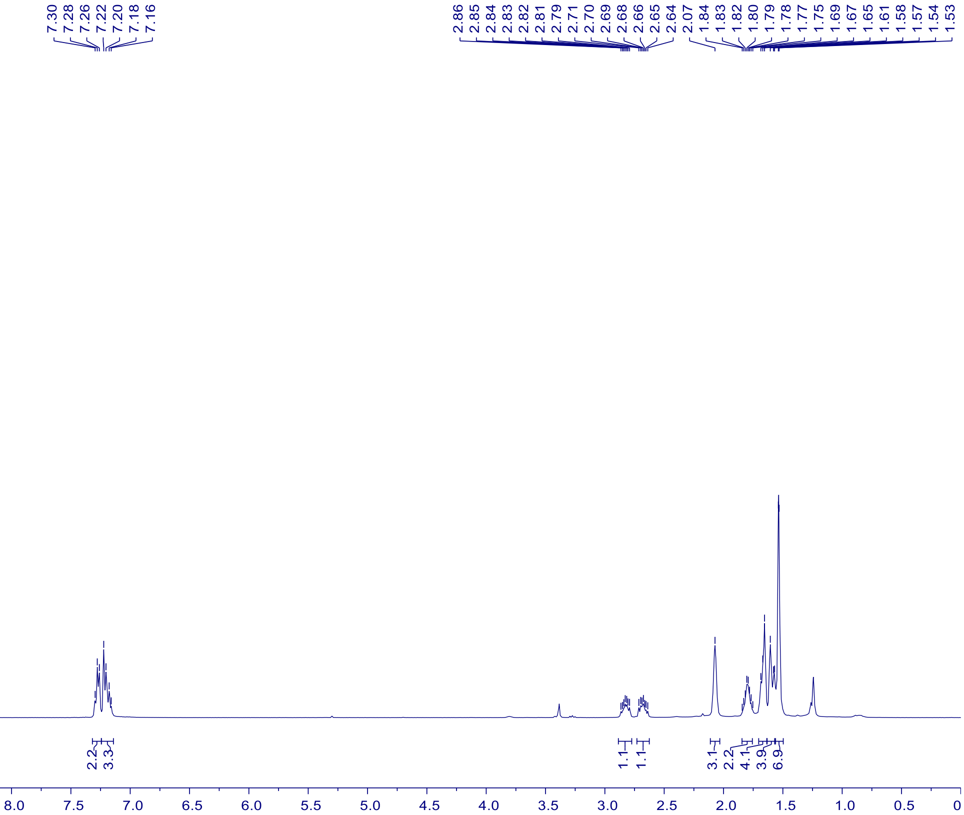

92 – ^{13}C NMR (126 MHz, $CDCl_3$)

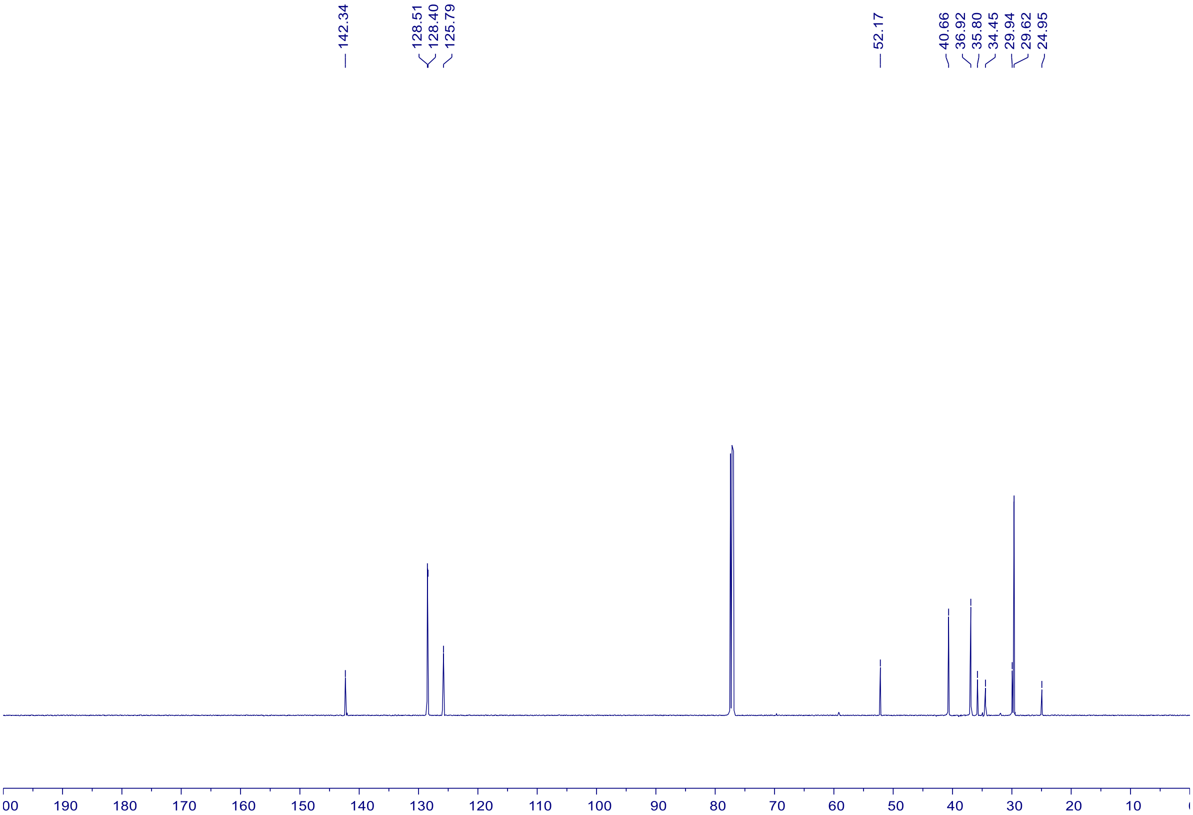

93 – ^{1}H NMR (500 MHz, CDCl$_3$)

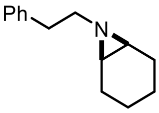

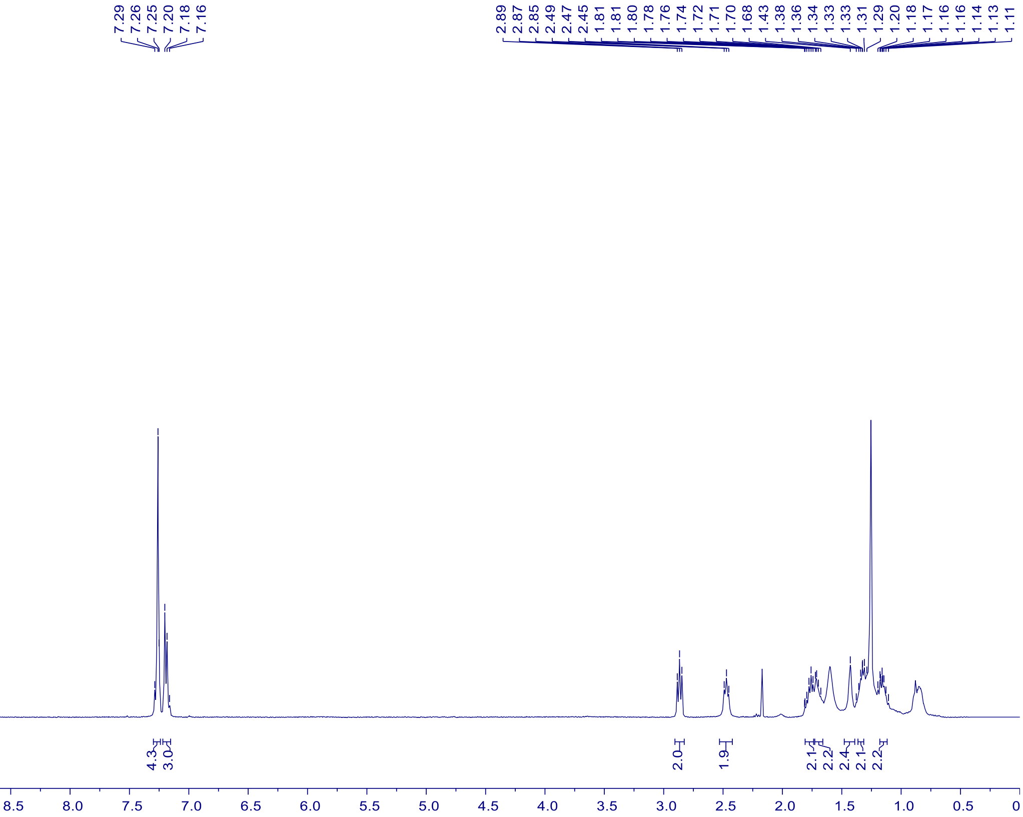

93 – ^{13}C NMR (126 MHz, CDCl$_3$)

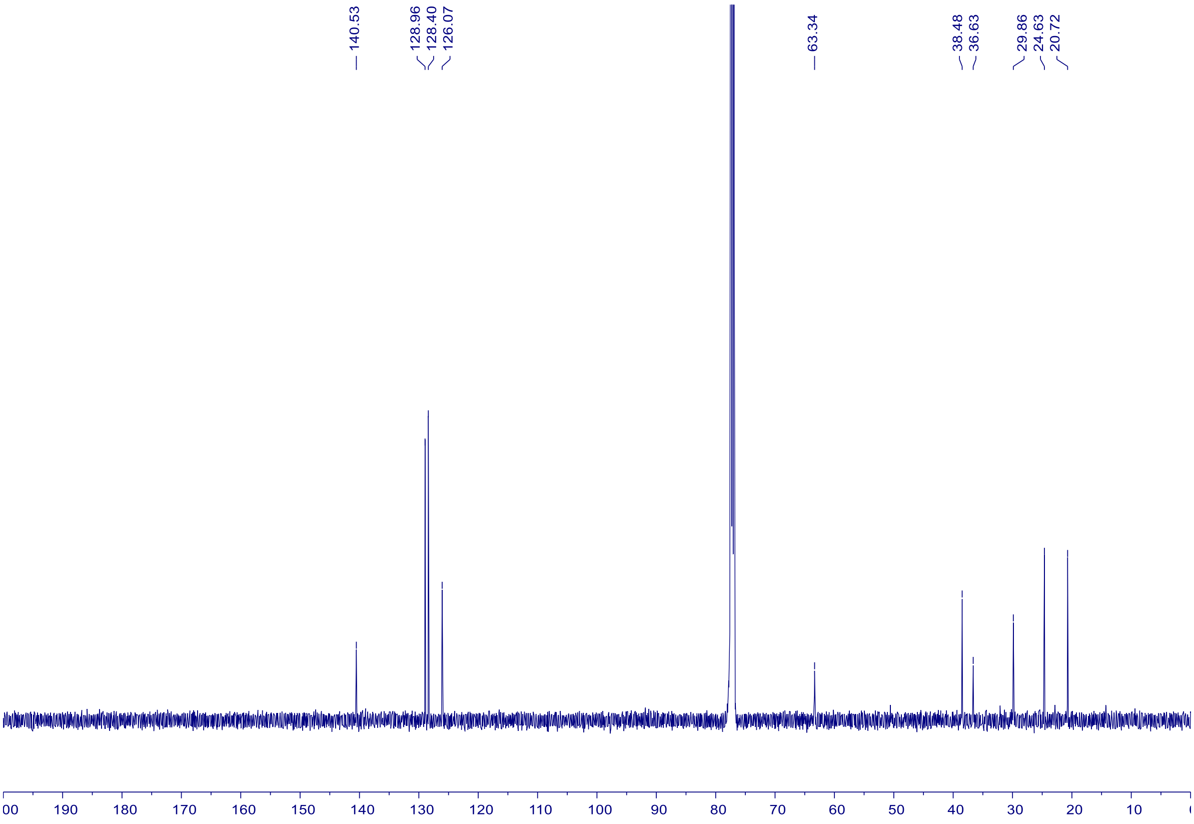

94 – ^{1}H NMR (500 MHz, $CDCl_3$)

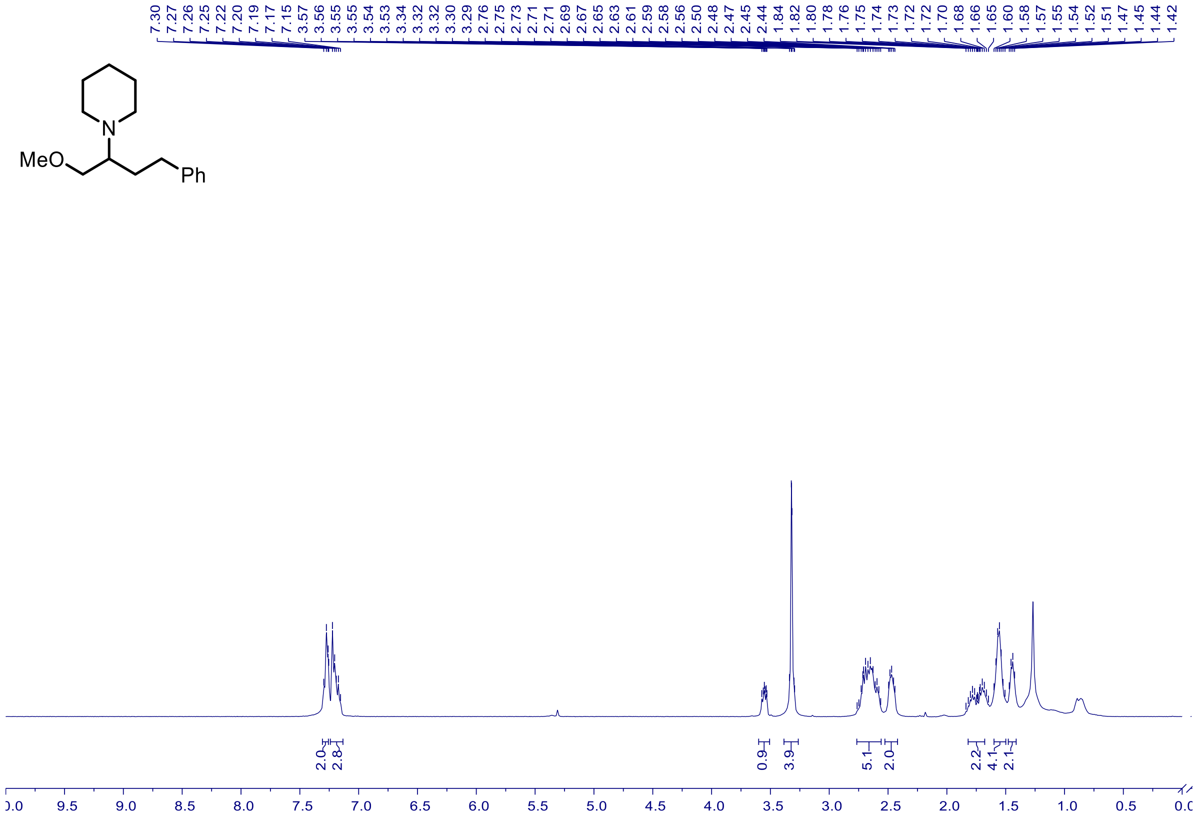

94 – ^{13}C NMR (126 MHz, $CDCl_3$)

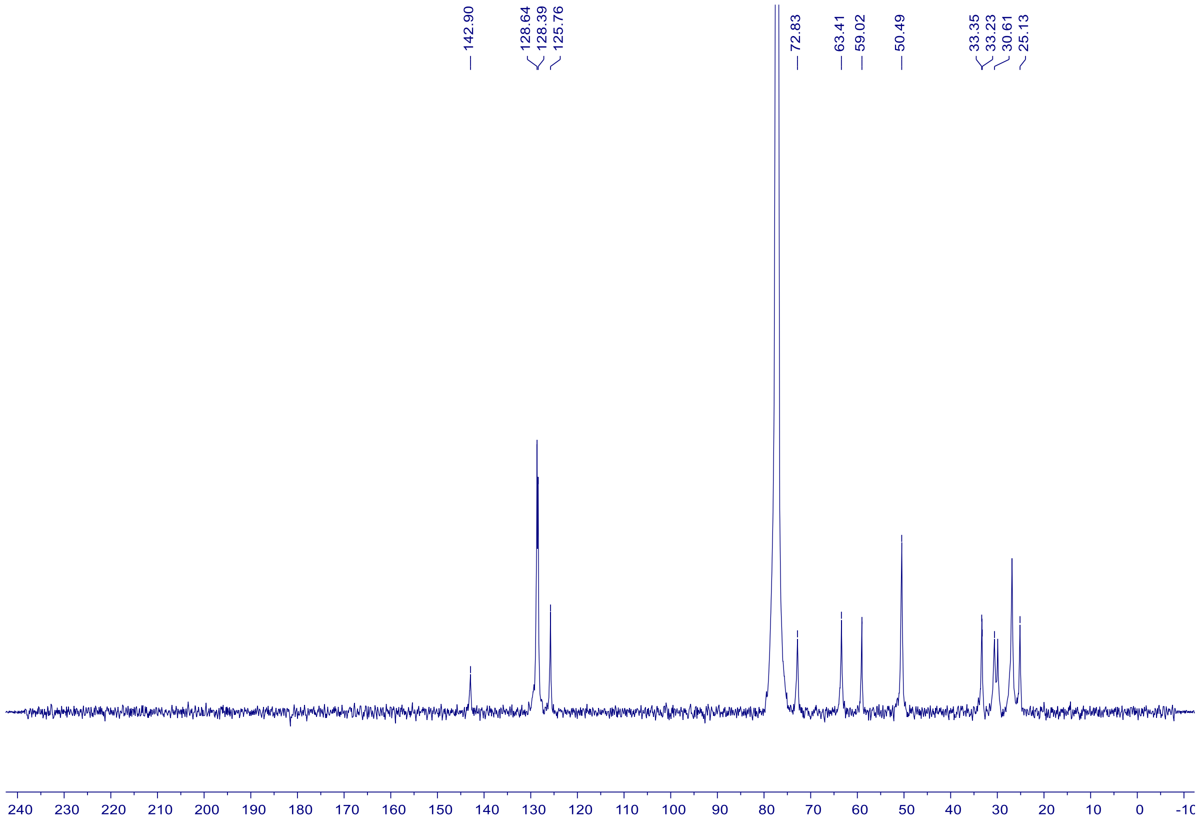

95 – ^1H NMR (400 MHz, $CDCl_3$)

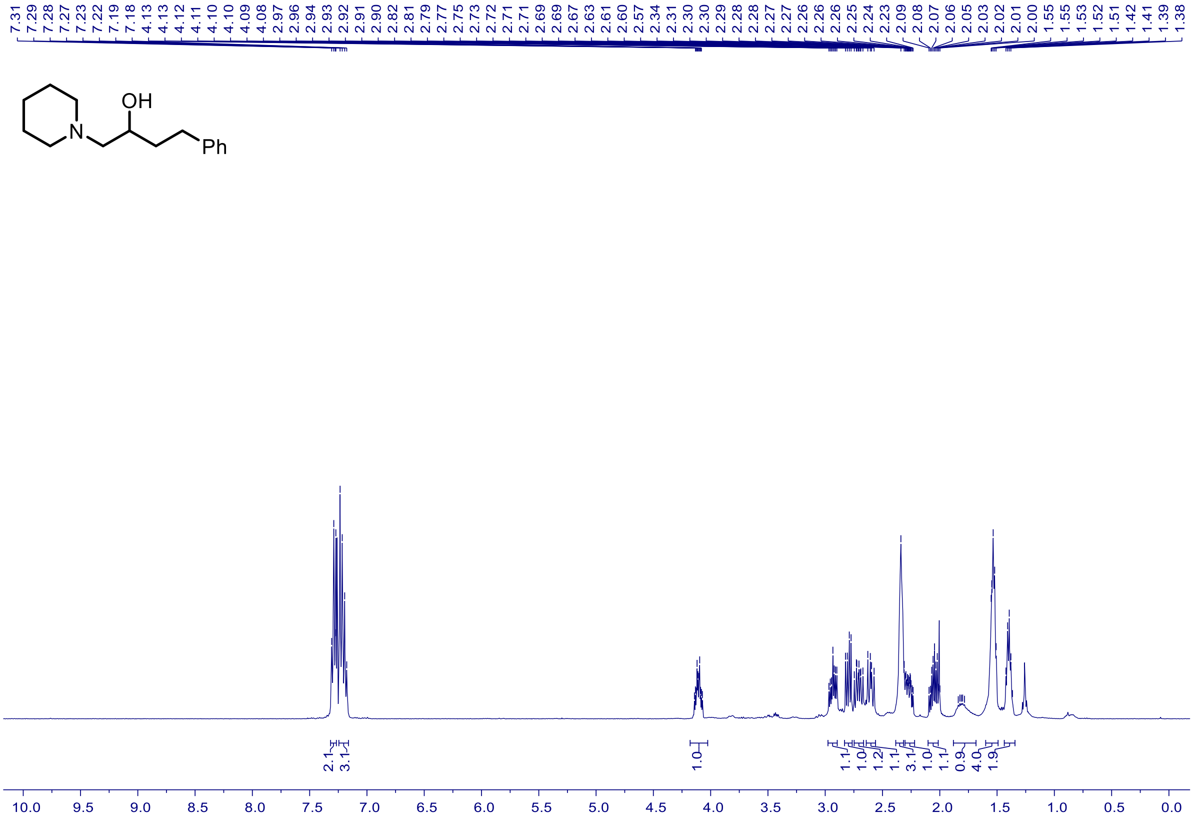

95 – ^13C NMR (126 MHz, $CDCl_3$)

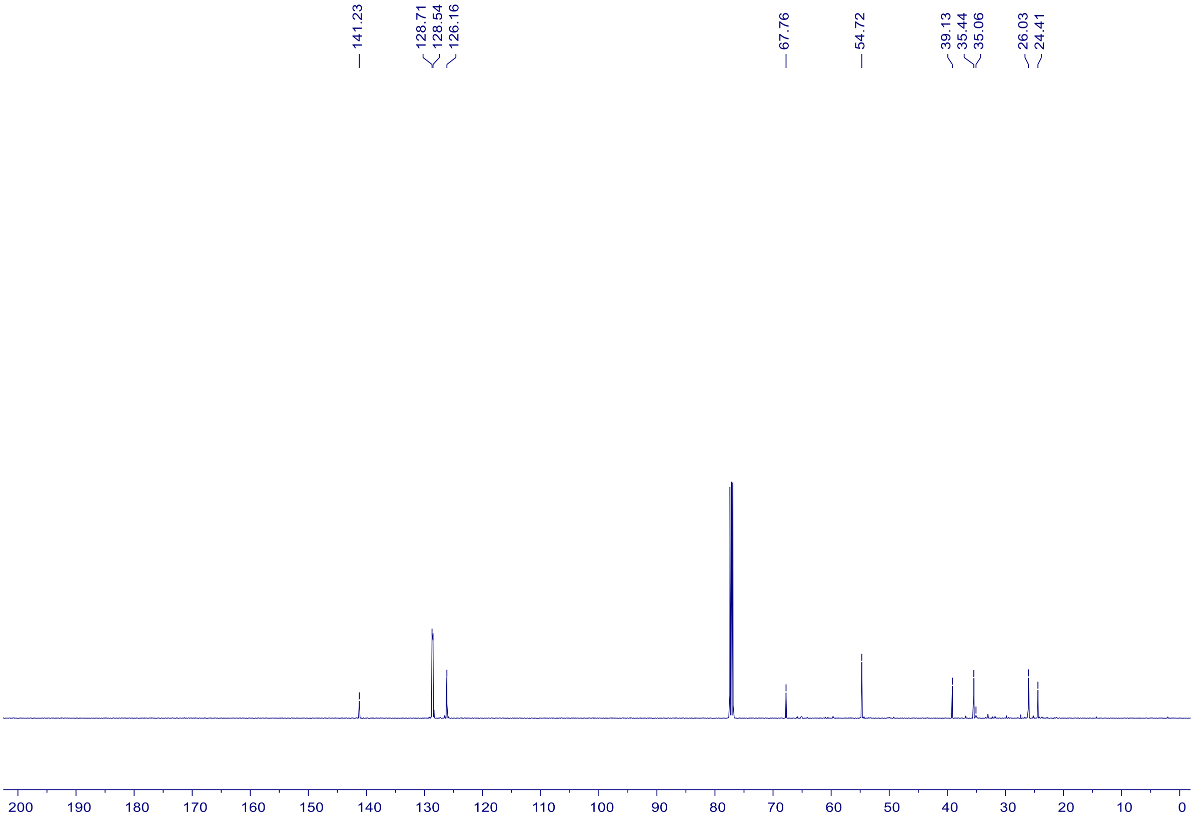

95 – ^{1}H–^{13}C HSQC (500–125 MHz, $CDCl_3$)

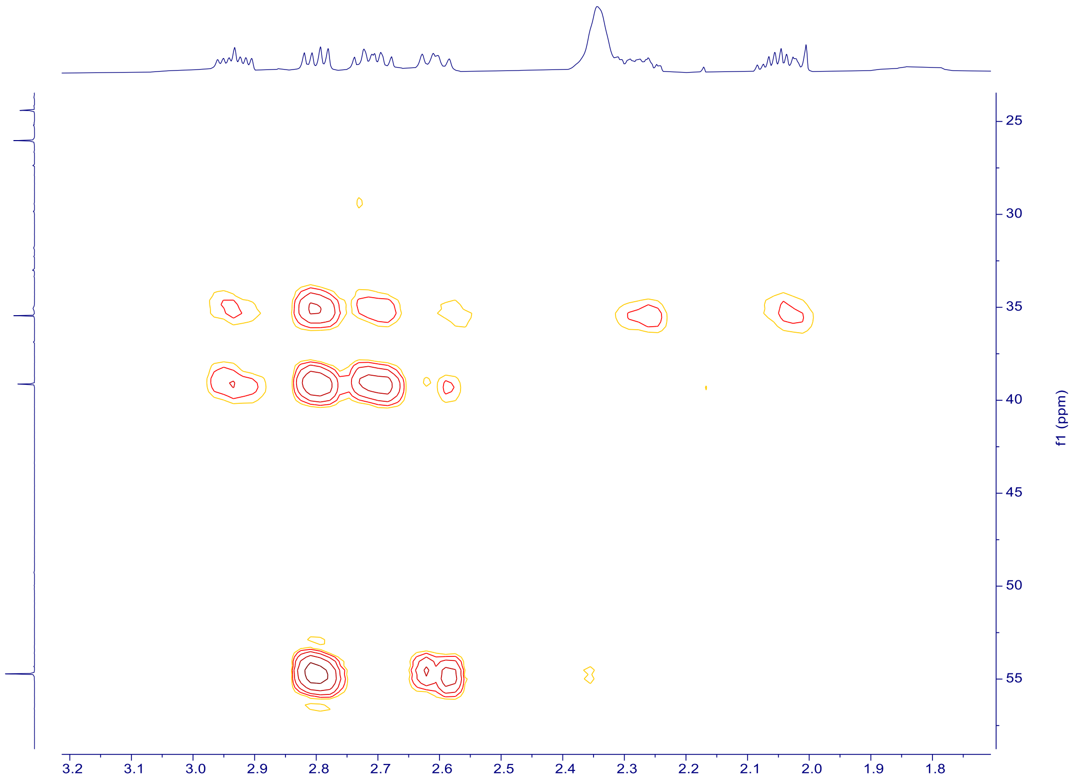

96 – ^{1}H NMR (500 MHz, $CDCl_3$)

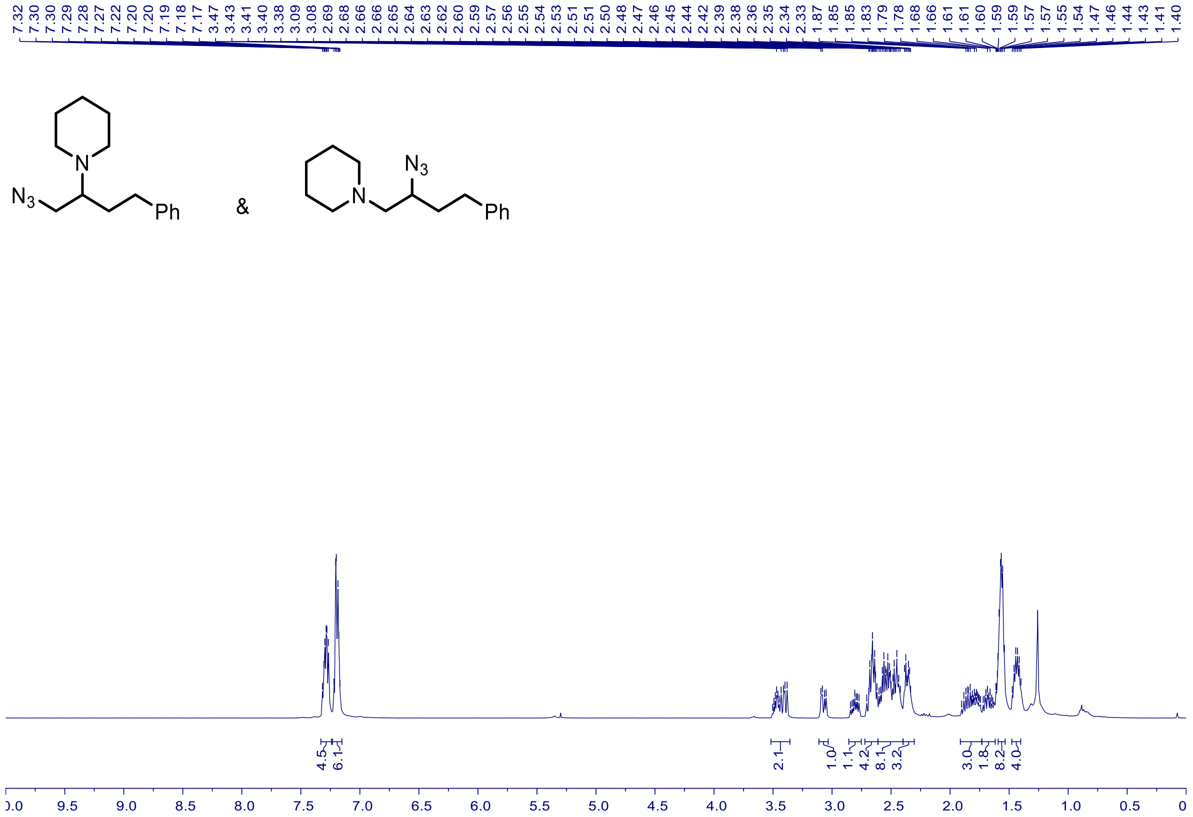

96 – ^{13}C NMR (126 MHz, $CDCl_3$)

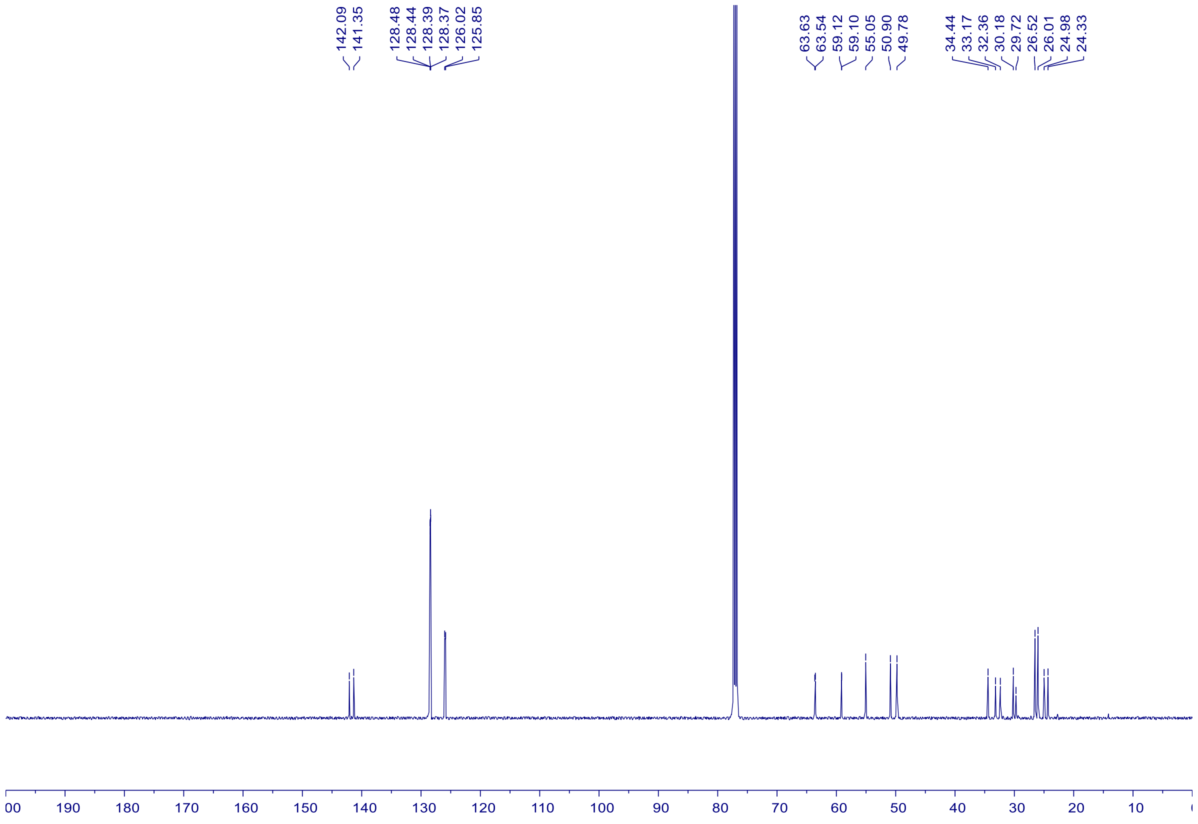

97 – ^{1}H NMR (500 MHz, $CDCl_3$)

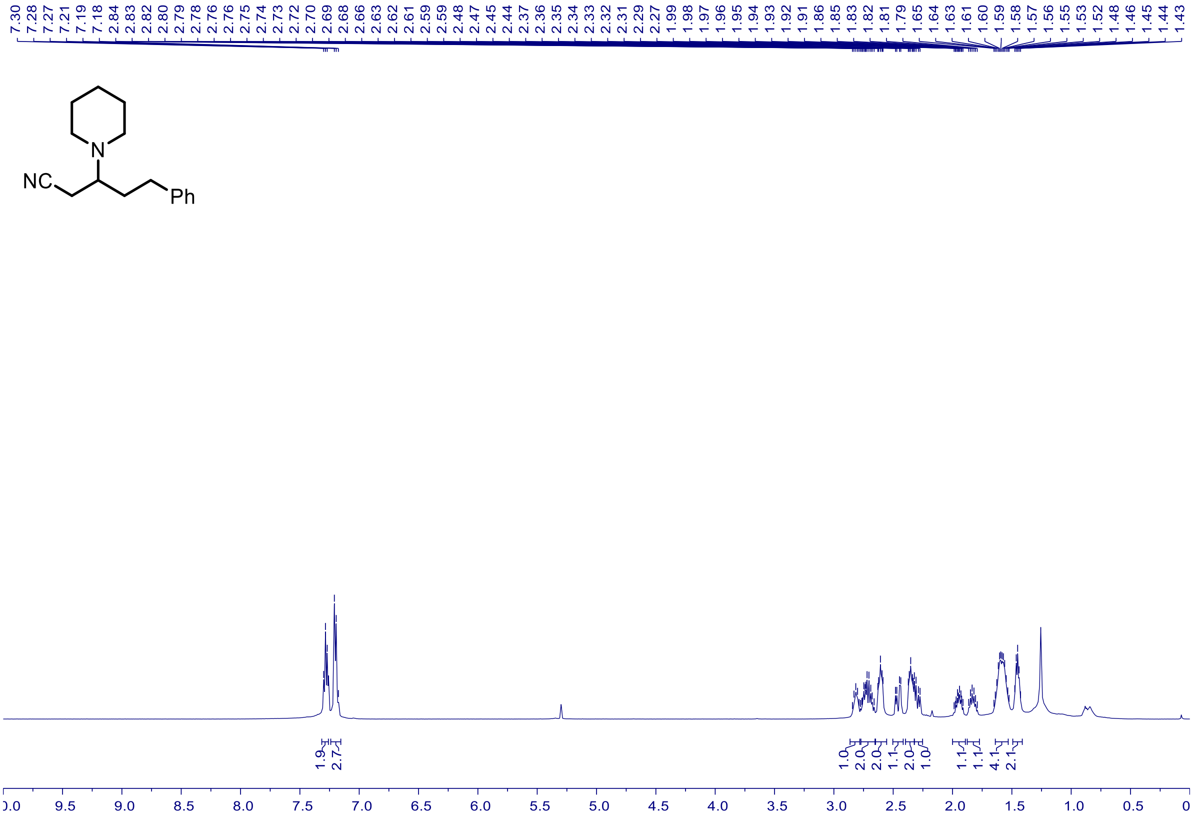

97 – ^{13}C NMR (126 MHz, $CDCl_3$)

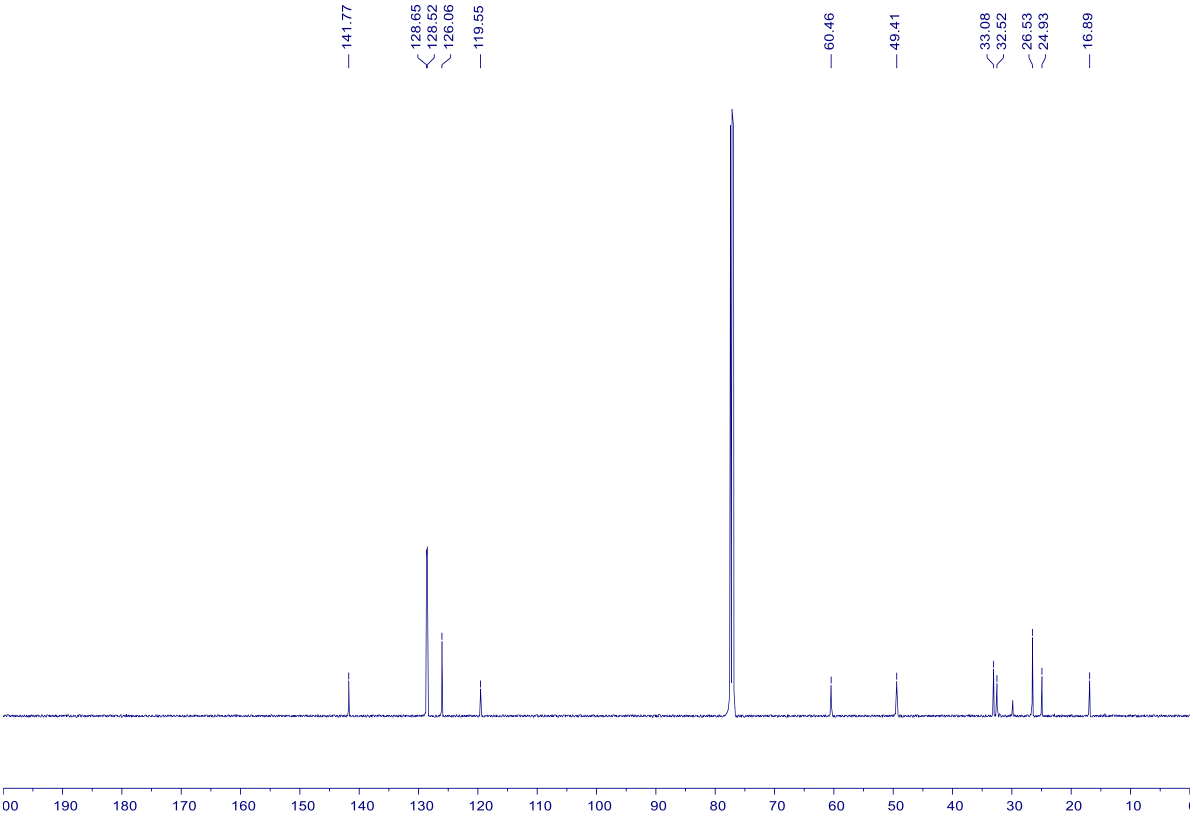

98 – ^{1}H NMR (500 MHz, $CDCl_3$)

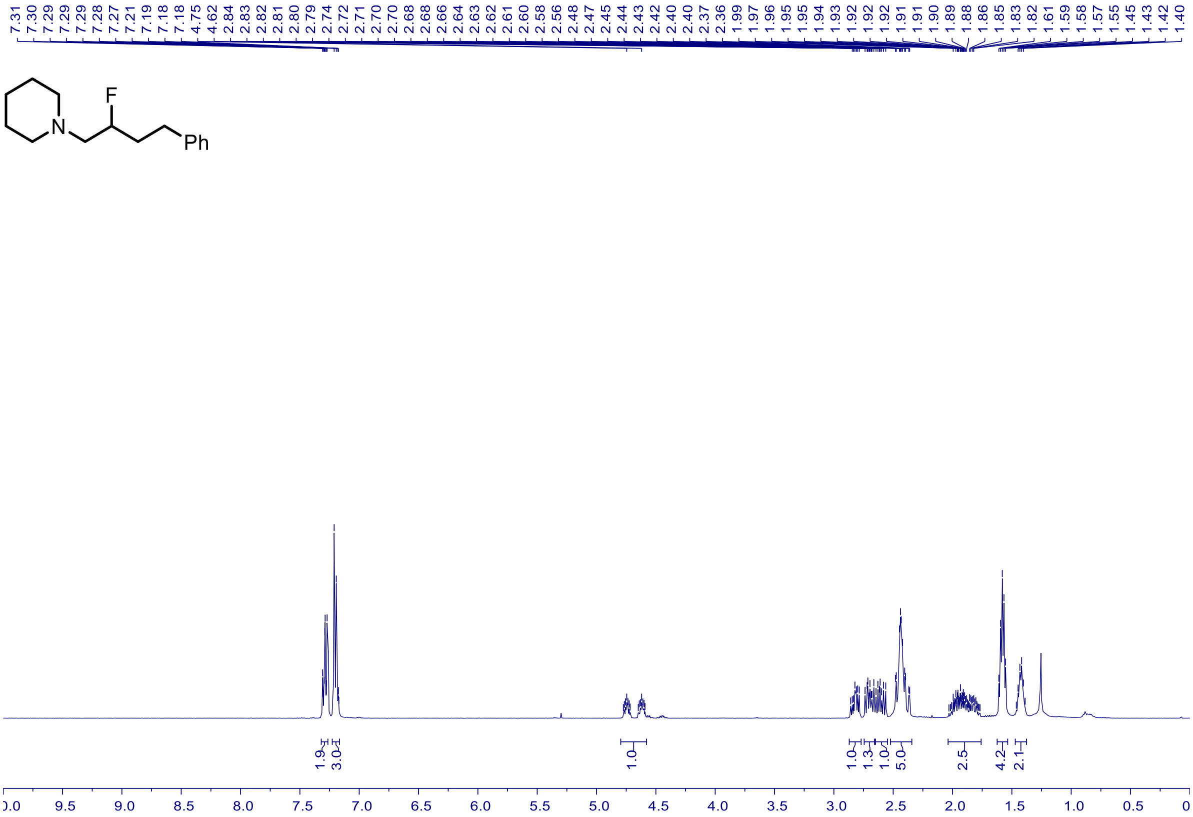

98 – ^{13}C NMR (126 MHz, $CDCl_3$)

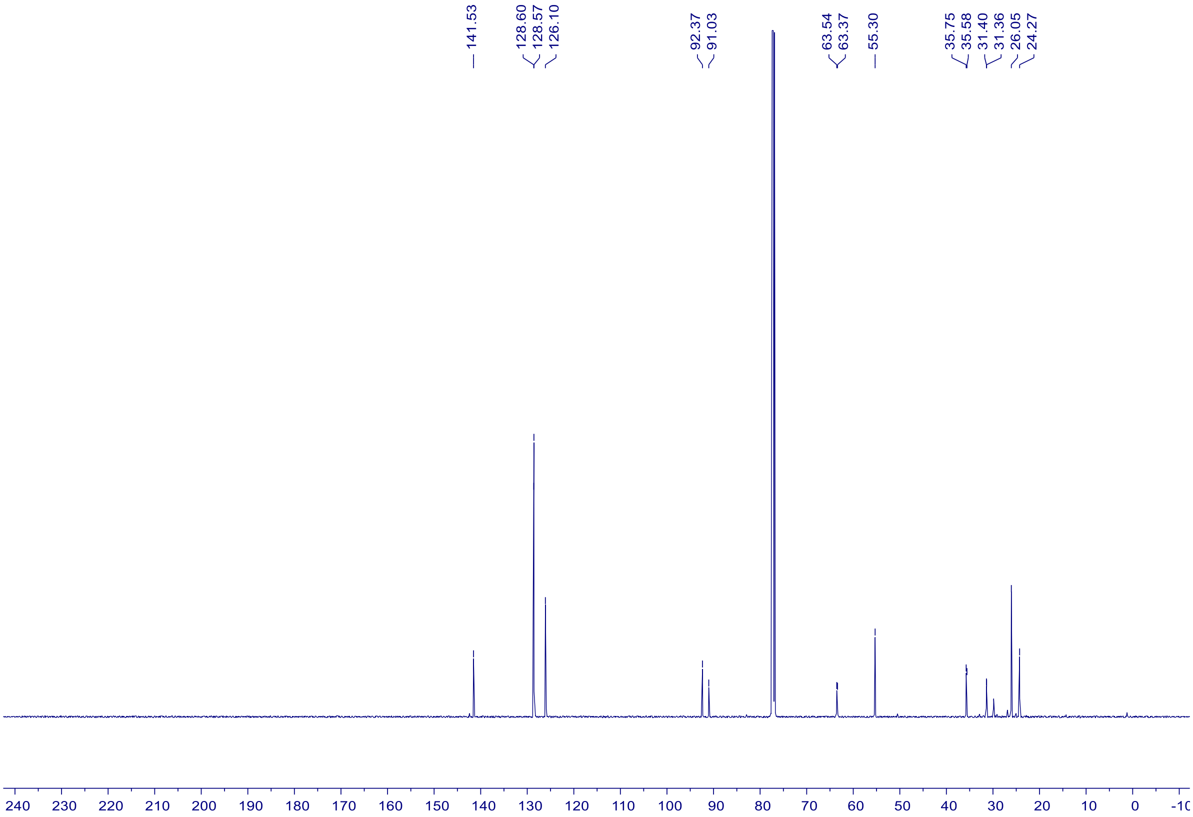

98 – ^{19}F NMR (471 MHz, $CDCl_3$)

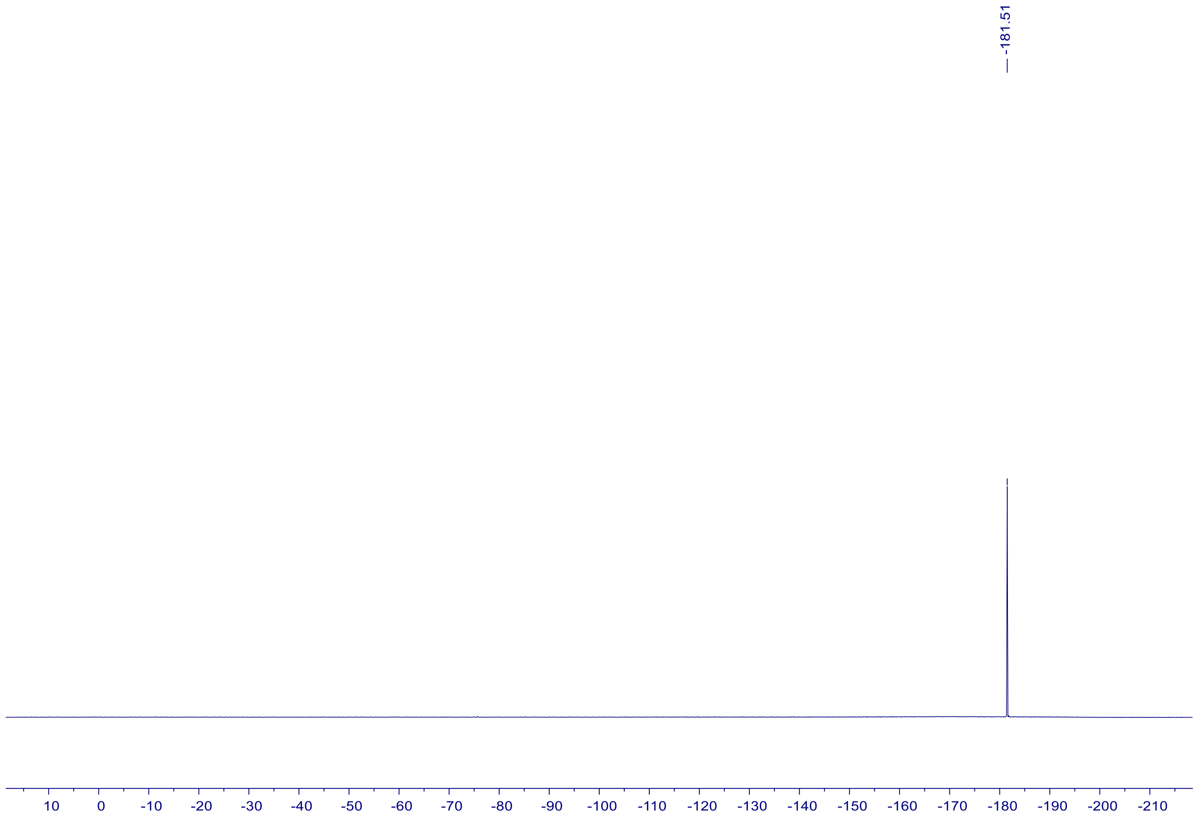

99 – ^{1}H NMR (500 MHz, CDCl$_3$)

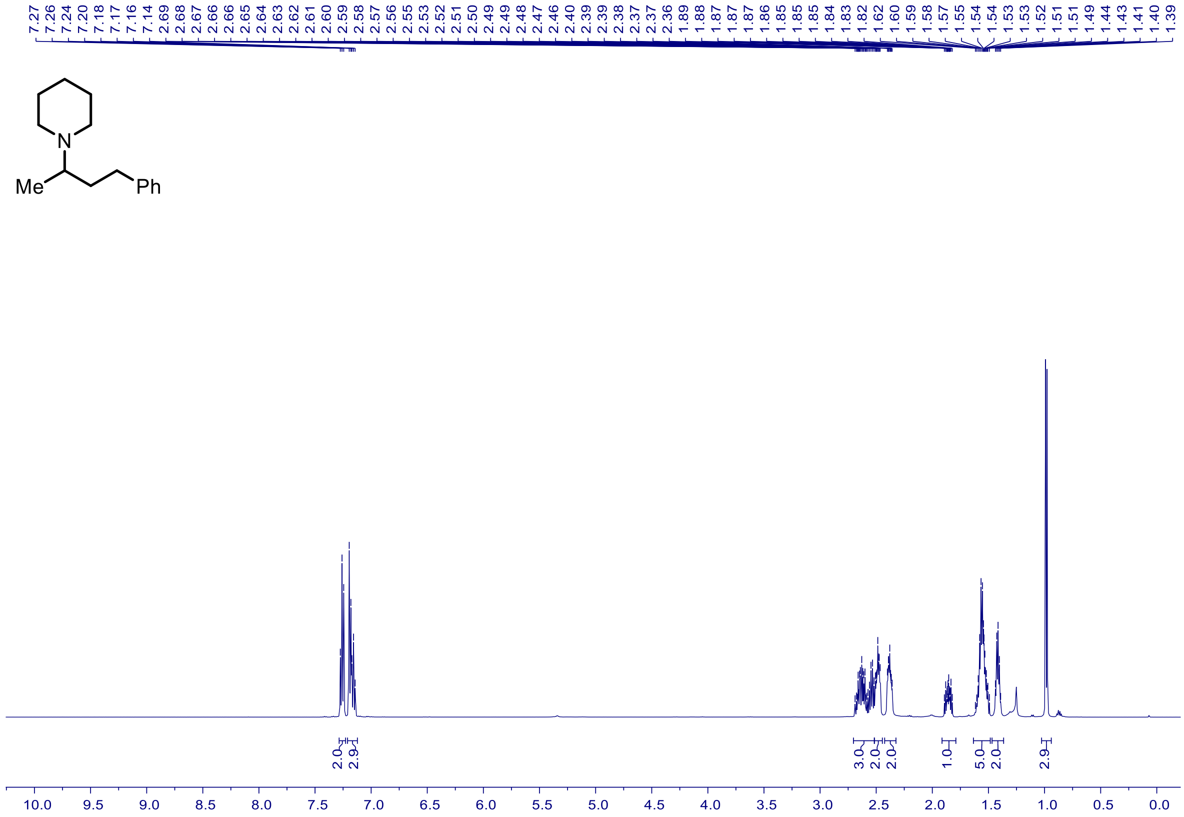

99 – ^{13}C NMR (500 MHz, CDCl$_3$)

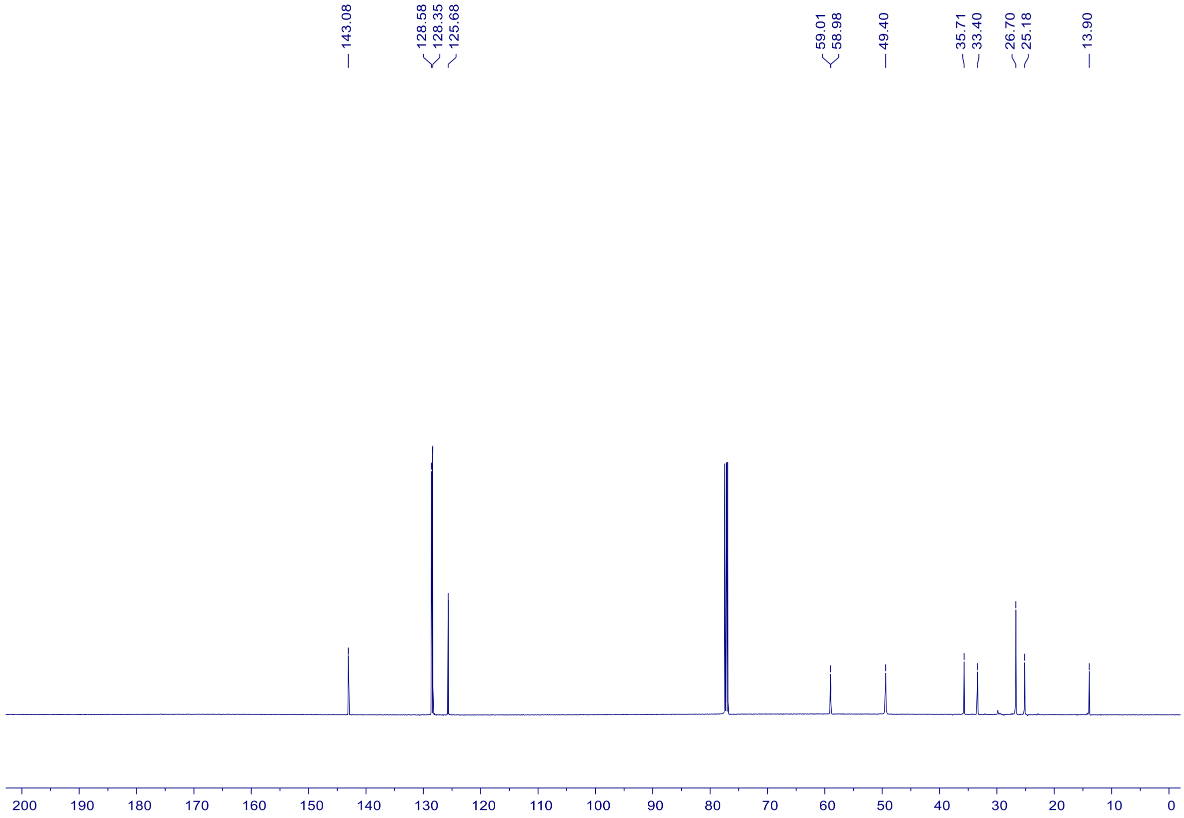

12 References

[1] B. M. Trost, K. Lehr, D. J. Michaelis, J. Xu, A. K. Buckl, *J. Am. Chem. Soc.* **2010**, *132*, 8915-8917.

[2] C. A. Urbina ‐ Blanco, A. Leitgeb, C. Slugovc, X. Bantreil, H. Clavier, A. M. Slawin, S. P. Nolan, *Chem. Eur. J.* **2011**, *17*, 5045-5053.

[3] N. Cabrera-Lobera, M. T. Quirós, W. W. Brennessel, M. L. Neidig, E. Buñuel, D. J. Cárdenas, *Org. Lett.* **2019**, *21*, 6552-6556.

[4] D. Marchand, J. Martinez, F. Cavelier, *Eur. J. Org. Chem.* **2008**, *2008*, 3107-3112.

[5] S. A. Green, S. Vásquez-Céspedes, R. A. Shenvi, *J. Am. Chem. Soc.* **2018**, *140*, 11317-11324.

[6] J.-x. Wang, X. Jia, T. Meng, L. Xin, *Synthesis* **2005**, *2005*, 2838-2844.

[7] S. Munnuri, A. M. Adebesin, M. P. Paudyal, M. Yousufuddin, A. Dalipe, J. R. Falck, *J. Am. Chem. Soc.* **2017**, *139*, 18288-18294.

[8] B. Bertrand, L. Stefan, M. Pirrotta, D. Monchaud, E. Bodio, P. Richard, P. Le Gendre, E. Warmerdam, M. H. de Jager, G. M. M. Groothuis, M. Picquet, A. Casini, *Inorg. Chem.* **2014**, *53*, 2296-2303.

[9] X. Li, Y. Feng, L. Lin, G. Zou, *J. Org. Chem.* **2012**, *77*, 10991-10995.

[10] D. Bernier, A. J. Blake, S. Woodward, *J. Org. Chem.* **2008**, *73*, 4229-4232.

[11] M. A. Cismesia, T. P. Yoon, *Chem. Sci.* **2015**, *6*, 5426-5434.

[12] A. Kumar, R. Parella, S. A. Babu, *Synlett* **2014**, *25*, 835-842.

[13] M. Chini, P. Crotti, F. Macchia, *J. Org. Chem.* **1991**, *56*, 5939-5942.

[14] P. Saravanan, A. Bisai, S. Baktharaman, M. Chandrasekhar, V. K. Singh, *Tetrahedron* **2002**, *58*, 4693-4706.

[15] B. Colman, S. E. de Sousa, P. O'Brien, T. D. Towers, W. Watson, *Tetrahedron: Asymmetry* **1999**, *10*, 4175-4182.

[16] M. E. Piotti, H. Alper, *J. Am. Chem. Soc.* **1996**, *118*, 111-116.

[17] V. B. Kharitonov, E. Podyacheva, Y. V. Nelyubina, D. V. Muratov, A. S. Peregudov, G. Denisov, D. Chusov, D. A. Loginov, *Organometallics* **2019**, *38*, 3151-3158.

[18] B. M. H. Kwon, Dong Cho; Lee, Sang Ku; Lee, Yu-Jin; Jeon, Yoon Jung; Ha, Ah-Rum; Han, Young Min;, (Ed.: U. S. P. Office), USA, **April 7, 2016**.
